# Synthesis and Conformational Analysis of Hydantoin-Based Universal Peptidomimetics

**DOI:** 10.1021/acs.joc.2c01903

**Published:** 2022-10-13

**Authors:** Alessio M. Caramiello, Maria Cristina Bellucci, Gaetano Cristina, Carlo Castellano, Fiorella Meneghetti, Matteo Mori, Francesco Secundo, Fiorenza Viani, Alessandro Sacchetti, Alessandro Volonterio

**Affiliations:** † Department of Chemistry, Materials and Chemical Engineering “Giulio Natta”, Politecnico di Milano, via Mancinelli 7, 20131Milano, Italy; ‡ Department of Food, Environmental and Nutritional Sciences, Università degli Studi di Milano, via Celoria 2, 20133Milano, Italy; § Department of Chemistry, Università degli Studi di Milano, via Golgi 19, 20133Milano, Italy; ∥ Department of Pharmaceutical Sciences, Università degli Studi di Milano, via Mangiagalli 25, 20133Milano, Italy; ⊥ Consiglio Nazionale delle Ricerche, Istituto di Scienze e Tecnologie Chimiche “G. Natta” (SCITEC), via Mario Bianco 9, 20131Milan, Italy

## Abstract

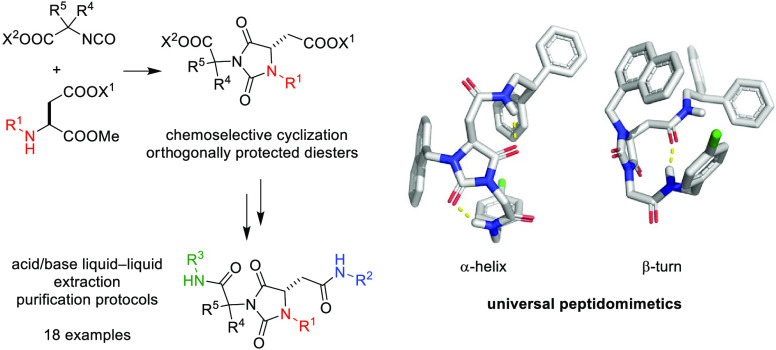

The synthesis of a collection of enantiomerically pure, systematically substituted hydantoins as structural privileged universal mimetic scaffolds is presented. It relies on a chemoselective condensation/cyclization domino process between isocyanates of quaternary or unsubstituted α-amino esters and *N*-alkyl aspartic acid diesters followed by standard hydrolysis/coupling reactions with amines, using liquid–liquid acid/base extraction protocols for the purification of the intermediates. Besides the nature of the α carbon on the isocyanate moiety, either a quaternary carbon or a more flexible methylene group, conformational studies *in silico* (molecular modeling), in solution (NMR, circular dichroism (CD), Fourier transform infrared (FTIR)), and in solid state (X-ray) showed that the presented hydantoin-based peptidomimetics are able to project their substituents in positions superimposable to the side chains of common protein secondary structures such as α-helix and β-turn, being the open α-helix conformation slightly favorable according to molecular modeling, while the closed β-turn conformation preferred in solution and in solid state.

## Introduction

“The most fruitful basis for the discovery of a new drug is to start with an old drug”. This is a sentence stated in 1988 by Nobel Laureate Sir James Whyte Black.^1^ Although almost 35 years have passed, the statement is still as relevant today. Indeed, there are highly favorable scaffolds, referred to as privileged scaffold, that are found in many synthetic drugs and can serve as templates to generate structurally diverse bioactive molecules for targeting more than one receptor type, including traditionally “undruggable” targets.^2^ The judicious decoration of these scaffolds, mostly heterocycles, has provided, and still provides, a useful strategy in medicinal chemistry. Indeed, from early “lock and key” theory and combinatorial chemistry to the modern drug repositioning,^3^ privileged substructure-based diversity-oriented synthesis (pDOS),^4^ biology-oriented synthesis (BIOS),^5^ and complexity-to-diversity (CtD) strategy,^6^ privileged scaffolds serve as “chemical navigators” to design targeted libraries first, and then to cover unexplored chemical space.^7^

Privileged scaffolds have been exploited also for the design of structural peptidomimetics, encompassing minimalist and universal mimetics, which are a particular class of peptidomimetics where a privileged scaffold that does not possess a peptide character is used as starting point to build structures able to mimic the secondary structures of the proteins, such as α-helix and β-turn.^8^ Since the pioneering works by Smith and Hirschmann, who used sugar, steroid, and catechol scaffolds for the design of β-turn mimetic,^9^ and Hamilton, who developed terphenyl and related scaffold α-helix mimetics,^10^ many other templates have been used to mimic one preferred peptide secondary conformation, such as pyrrolidine,^11^ pyridazine,^12^ pyrrolopyrimidines,^13^ oligooxopiperazine,^14^ bicyclic lactam,^15^ benzodiazepine,^16^ among others. These mimetics, *i.e.*, minimalist mimetics, possess conformations with energies similar to the global minima where their substituents overlap with some of the key *i* + *n* positions of α-helices or β-turn. More recently, Burgess and co-workers have introduced the concept of universal peptidomimetics, which are a particular class of structural mimetics designed on scaffolds that being not too rigid are able to mimic different, if not all, secondary structures through rotation around a few of significant degrees of freedom.^17^ Being easy to be functionalized with substituents corresponding to many of the side chains of the protein-derived amino acids, universal peptidomimetics could be very useful for the design of libraries for high-throughput screening programs against diverse targets, including conventionally “undruggable” targets, *i.e.*, those proteins that are not yet being targeted.^18^ Heterocycles as omegatides,^19^ piperidine-piperidinones,^20^ and pyrrolinone-pyrrolidine oligomers^21^ have been used as core skeletons for the construction of universal peptidomimetics able to target local pairs of amino acids in ideally any secondary structure. Thanks to their relative flexibility, universal peptidomimetics are very intriguing because, when the binding is mostly governed by the side chains (for example, in protein–protein interactions), they can interact selectively their targets displaying their side chains in the appropriate conformations, overall when the exact binding conformations of the target is unknown. For this reason, there is a great interest in (1) finding new scaffolds for universal peptidomimetics and (2) developing convenient synthetic strategies for the preparation of libraries to supply high-throughput screening programs.

**Scheme 1:**
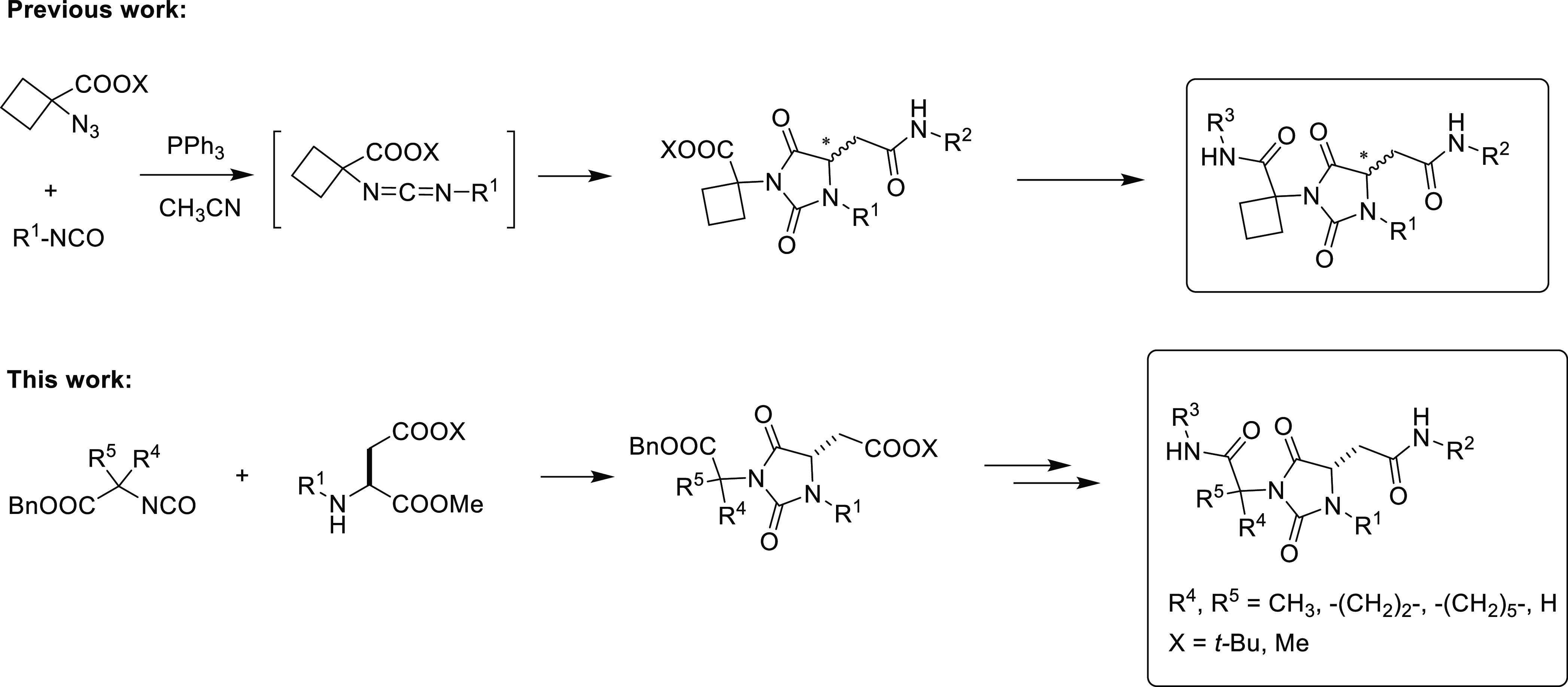
Synthetic Strategies for the Synthesis of Hydantoin-Based Universal Peptidomimetics

Hydantoin, being a small heterocycle having four derivatizable positions to generate diversity and a maximum of four hydrogen-bond donors/acceptors, are a class of very intriguing, privileged scaffolds exhibiting a wide spectrum of biological activities as evidenced by the number of marketed drugs as anticonvulsants, postsynaptic muscle relaxants, and androgen receptor agonists, and clinical candidates in the treatment of psoriasis, LFA-1 antagonist, and in the treatment of Duchenne muscular dystrophy.^22^ Indeed, even if hydantoin heterocycle could be considered an “old” privileged scaffold, over the past years, the synthetic and pharmaceutical interest in these heterocycle-based compounds, encompassing more elaborated fused- and spiro-hydantoins, has not experienced a decrease in interest as evidenced by the number of publications and patents appeared in the literature dealing with both methodological and medicinal chemistry. The hydantoin core has been also used also as privileged skeleton for the design of minimalist and universal peptidomimetics.^23^ In this context, we have described the design and the conformational analysis of racemic, universal peptidomimetic 3-*cyclo*-butylcarbamoyl hydantoins which were synthesized through a regioselective multicomponent (MC) domino process starting from α-azido-*cyclo*-butyl carboxylic ester and isocyanates (Scheme 1).^24^ Modeling experiments have shown that these compounds can exist at room temperature (rt) in kinetically accessible α-helix and β-turn conformations which can exchange with no significant entropic penalties, presenting the substituents R^1^, R^2^, and R^3^ to the classical *i* + *n* side-chain positions of secondary structures. To clarify the importance of the exo-quaternary carbon on the stabilization of the conformations and to widen the synthetic scope of this class of universal peptidomimetics, we present herein the synthesis and conformation analysis of (1) analogues having different quaternary substituents, such a *gem*-dimethyl, *cyclo*-hexyl, and *cyclo*-propyl, and (2) analogues lacking the substituent in that position (glycine derivatives) (Scheme 1). Although the straightforward MC process reported earlier has been the first choice for the synthesis of these derivatives, it failed to give a regioselective process in some cases and high yields in others (see below). For this reason, we exploited a chemoselective process involving only liquid–liquid acid/base extractions for the isolation and purification of all intermediates, thus suitable for combinatorial synthesis purposes. In addition, this synthetic pathway allowed us to prepare a selected collection of enantiomerically pure derivatives instead of racemic mixtures that we would have obtained with the MC process.

## Results and Discussion

### Synthesis

**Scheme 2:**
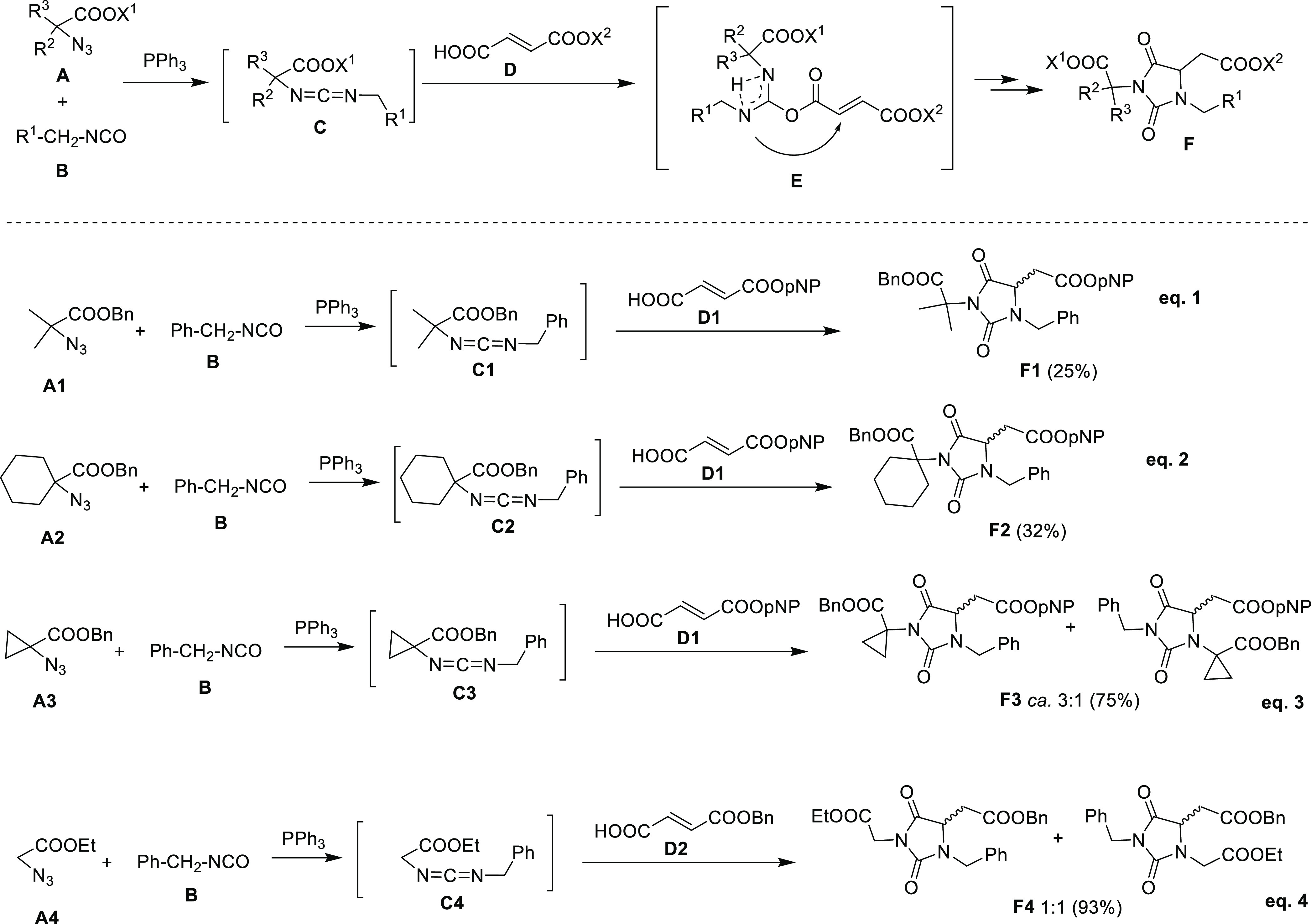
MC Process Leading to Hydantoin **F**

The MC process exploited for the synthesis of racemic 3-*cyclo*-butylcarbamoyl hydantoin universal peptidomimetics **F** relies on a regioselective intermolecular aza-Michael addition of the *O*-acyl isourea intermediate **E** formed by a reaction between *in situ* generated carbodiimides **C** and α,β-unsaturated fumaric acid mono esters **D** (Scheme 2).^25^ The regiocontrol of the reaction is dictated by the difference between the steric hindrance of the two nucleophilic amino moieties, *i.e.*, one bearing the bulky *cyclo*-butyl (R^2^, R^3^ = −(CH_2_)_3_−) *versus* the other bearing a methylene group. Due to the efficiency, we attempted to exploit the same process for the synthesis of derivatives having different substituents on the azide **A**. In particular, we sought that other azides linked to a tertiary carbon could have given the same regiochemical control leading to the formation of only one isomer. Indeed, the process was regioselective with *gem*-dimethyl glycine azide (R^2^ = R^3^ = Me, eq 1, Scheme 2) and 1-azidocyclohexane carboxylic acid benzyl ester (R^2^, R^3^ = −(CH_2_)_5_–, eq 2, Scheme 2) leading to the formation of hydantoins **F1** and **F2**, respectively, but in low yields. This is probably due to the steric hindrance in the reaction between the starting azide and Ph_3_P during the first step of the process. Starting with 1-azidocyclopropane carboxylic acid benzyl ester (R^2^, R^3^ = −(CH_2_)_2_–, eq 3, Scheme 2) the process occurred with high yield but low regiocontrol (3:1 mixture of regioisomers **F3**), probably due to the constrain in the cycle that renders the azido group more accessible in the Staudinger reaction but, at the same time, the resulting amino moiety not much more sterically congested than the amino group bearing a primary carbon in the *O*-acyl isourea intermediate **E**. Finally, we tried the reaction with azido glycine benzyl ester (R^2^, R^3^ = H, eq 4, Scheme 2) hoping that the higher nucleophilicity of an alkyl amine compared to the amino group belonging to the glycine moiety would lead certain stereocontrol. However, although the yield was high, the process occurred with no regiocontrol, and an equimolecular mixture of hydantoins **F4** was obtained.

With these results in hand, we sought an alternative synthetic pathway that could be exploited for the combinatorial synthesis of libraries of hydantoin-based universal peptidomimetics adopting a simple, technically not demanding multistep protocol that would allow us to avoid tedious chromatographic purifications of the intermediates by employing simple solution-phase protocols based on acid/base liquid/liquid extractions.^26^ Actually, a convenient method to synthesize *N*,*N*′-disubstituted hydantoins under mild conditions consists in the reaction between isocyanates and *N*-alkyl-α-amino esters which occurs through a condensation/cyclization domino process (Scheme 1).^27^ However, to the best of our knowledge, this protocol has never been used starting with *N*-alkyl aspartic acid esters and α-aminoester isocyanates, which could give a non-regiospecific process yielding a mixture of the desired hydantoin scaffold **5** along with the six-membered ring **6** arising from the attack of the urea-NH to the ester moiety on the aspartic acid side chain during the cyclization step (see scheme in Table 1).^28^ It is worth noting that if successful, this procedure would allow us to prepare enantiomerically pure hydantoin universal peptidomimetics in contrast to the reported MC domino process through which we synthesized 3-*cyclo*-butylcarbamoyl hydantoins in racemic form (Scheme 1).

**Table 1:**
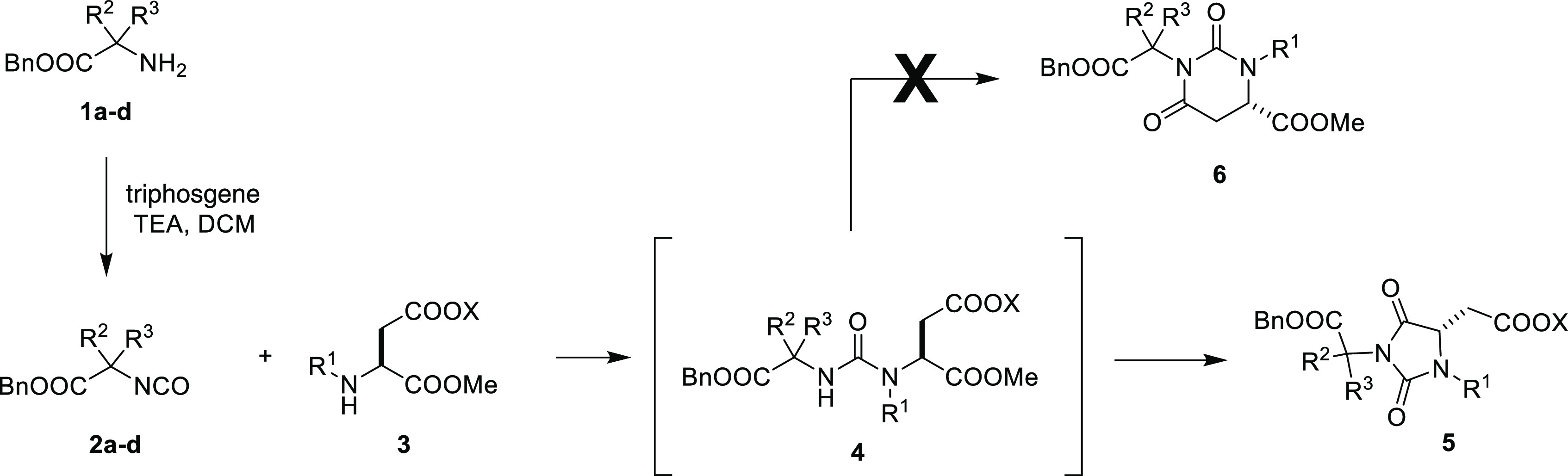

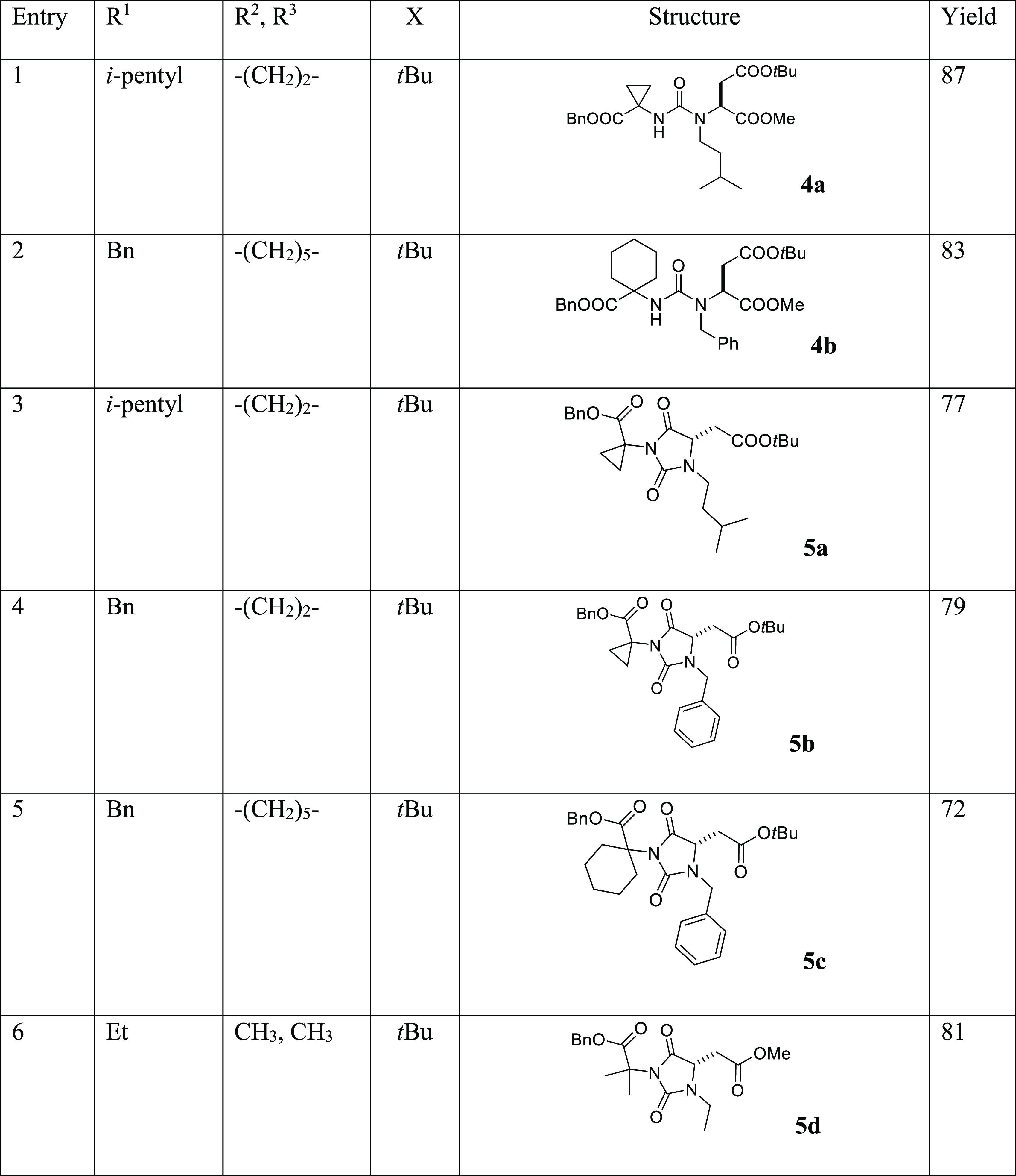

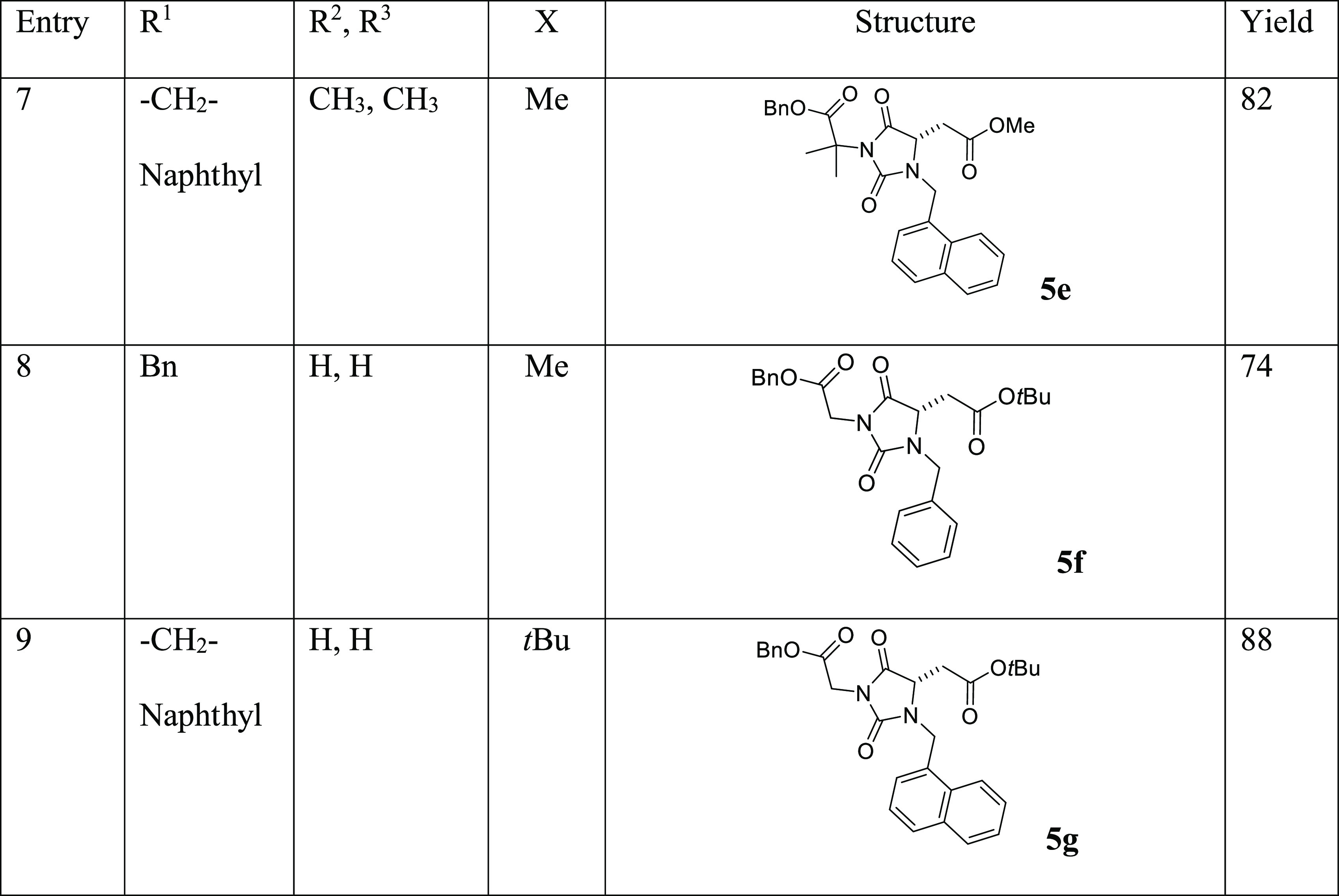
Regioselective Synthesis of Hydantoin Intermediates **5**

To check the regioselectivity of the cyclization, we synthesized the urea derivatives **4a** and **4b** by treating isocyanate **2a** and **2b** with *N*-*iso*-pentyl and *N*-benzyl aspartic esters **3a** and **3b**, respectively, in dichloromethane (DCM) at 0 °C for 1 h (entries 1 and 2, Table 1). To our delight, either cyclization triggered by the treatment of **4a** and **4b** with a strong base (typically 1.0 M NaOH in Schotten–Baumann conditions) in a very short time (less than 5 min) or by leaving overnight **4a** and **4b** in the presence of a weaker organic base (typically triethylamine (TEA) in DCM solution) yielded the formation of the corresponding hydantoin **5a** and **5c** with total regiocontrol and very good yields (data not shown).^29^

**Scheme 3:**
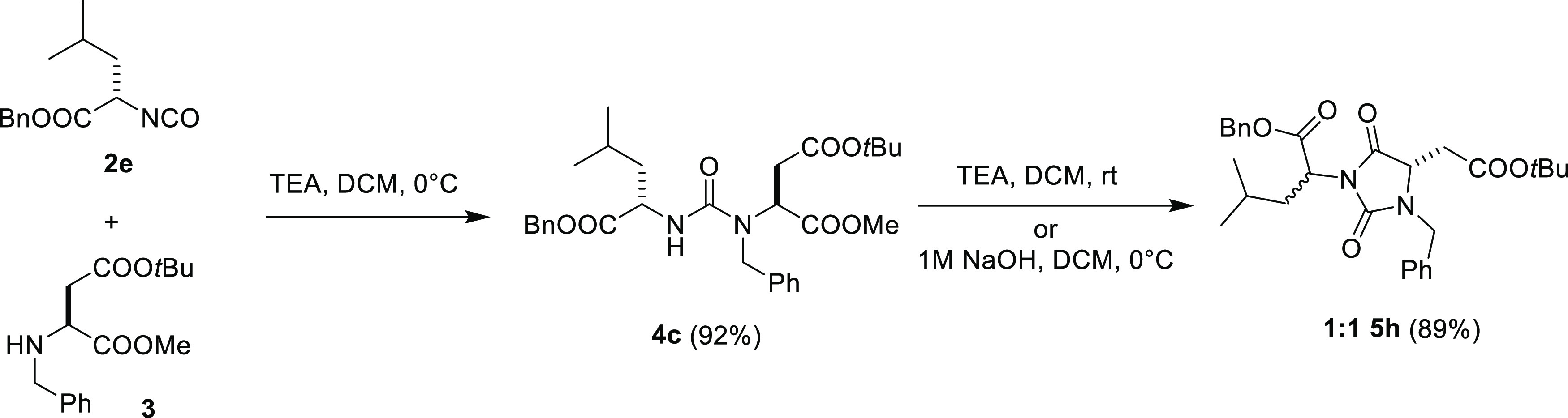
Synthesis of Hydantoin Intermediate **5h**

With this result in hand, we decided to synthesize a collection of seven systematically modified hydantoin derivatives **5a**–**h** by performing the addition/cyclization process one-pot, *i.e.*, by leaving the reaction with an excess of TEA overnight or, when the cyclization process is not complete in the former conditions, by adding a 1 M aqueous NaOH solution at the end of the condensation step (Table 1). To our delight, the process worked efficiently also with glycine ester isocyanate (entries 8 and 9, Table 1) demonstrating that the regioselectivity achieved in the cyclization step is not driven by the presence of a quaternary carbon which could stabilize a particular reactive conformation. In this way, we obtained hydantoin scaffolds having (1) different exo-quaternary carbon on the N-3 substituent, such as *cyclo*-propyl (entries 3 and 4, Table 1), *cyclo*-hexyl (entry 5, Table 1), *gem*-dimethyl (entries 6 and 7, Table 1), or unsubstituted glycine derivative (entries 8 and 9, Table 1); (2) different substituents on the N-1 position, such as *iso*-pentyl (entry 3, Table 1), benzyl (entries 4, 5, and 8, Table 1), ethyl (entry 6, Table 1), naphthyl (entries 7 and 9, Table 1); and (3) two carboxyl groups orthogonally protected as benzyl and *tert*-butyl esters (entries 3–5 and 8–9, Table 1) or benzyl and methyl esters (entries 6 and 7, Table 1) ready to be functionalized after selective hydrolysis. It is worth noting that all of these intermediates have been recovered in high yields and purities after simple acid/base extraction procedures and used in the following reactions without any further purification. We applied the process also starting from enantiomerically pure α-aminoester isocyanates such as leucine benzyl ester isocyanate **2e** to have another point of diversity in the final hydantoin-based peptidomimetics (Scheme 3). However, while the addition step worked nicely producing enantiomerically pure intermediate **4c** in good yields, all of the attempts made for the cyclization step yielded an almost equimolar mixture of two diastereoisomeric hydantoins **5h**, due to epimerization of the stereocenter of the reacting isocyanate **2e**. Moreover, we obtained the same epimerization also during the following coupling steps (data not shown), demonstrating the extreme stereochemical instability of this stereocenter.

**Scheme 4:**
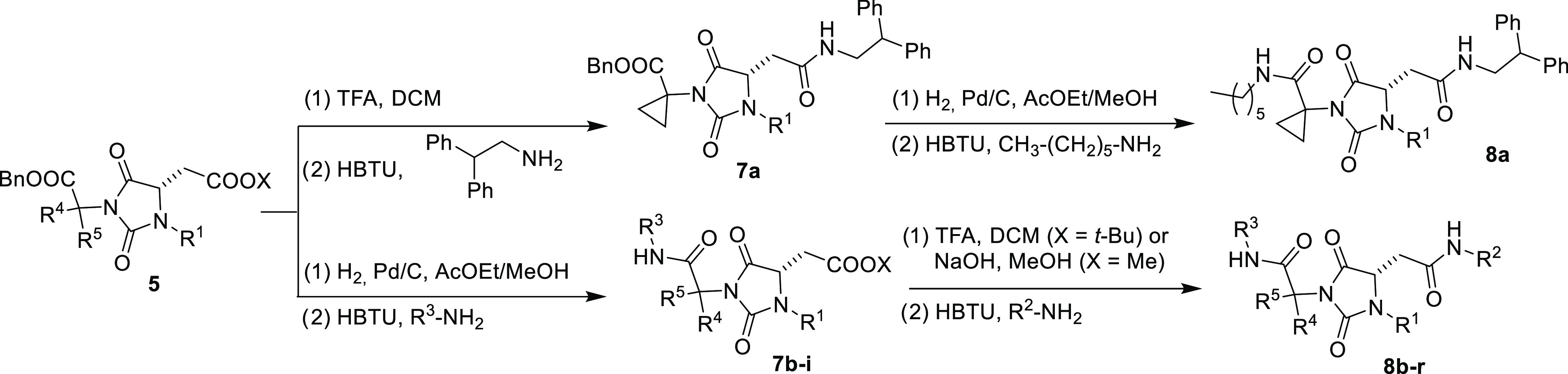
Synthesis of Hydantoin Universal Peptidomimetics **8**

Starting from intermediates **5a**–**g** the following steps were performed using standard hydrolysis/condensation protocols (Scheme 4), *i.e.*, trifluoroacetic acid (TFA)-promoted hydrolysis of the *tert*-butyl ester of intermediate **5b** and coupling with 2,2-diphenylethylamine leading to hydantoin monoamide **7a** followed by hydrogenolysis of the benzyl ester and coupling with hexylamine yielding **8a**, or hydrogenolysis of the benzyl ester of **5a**, **5c**–**g** catalyzed by Pd/C and coupling promoted by 2-(1*H*-benzotriazol-1-yl)-1,1,3,3-tetramethyluronium hexafluorophosphate (HBTU) of the resulting free carboxylic acid with a first amine yielding compounds **7b**–**i**, followed by acid hydrolysis of the *tert*-butyl ester or NaOH promoted hydrolysis of the methyl ester and coupling in the same conditions with a second amine producing final peptidomimetics **8b**–**r**.

**Chart 1:**
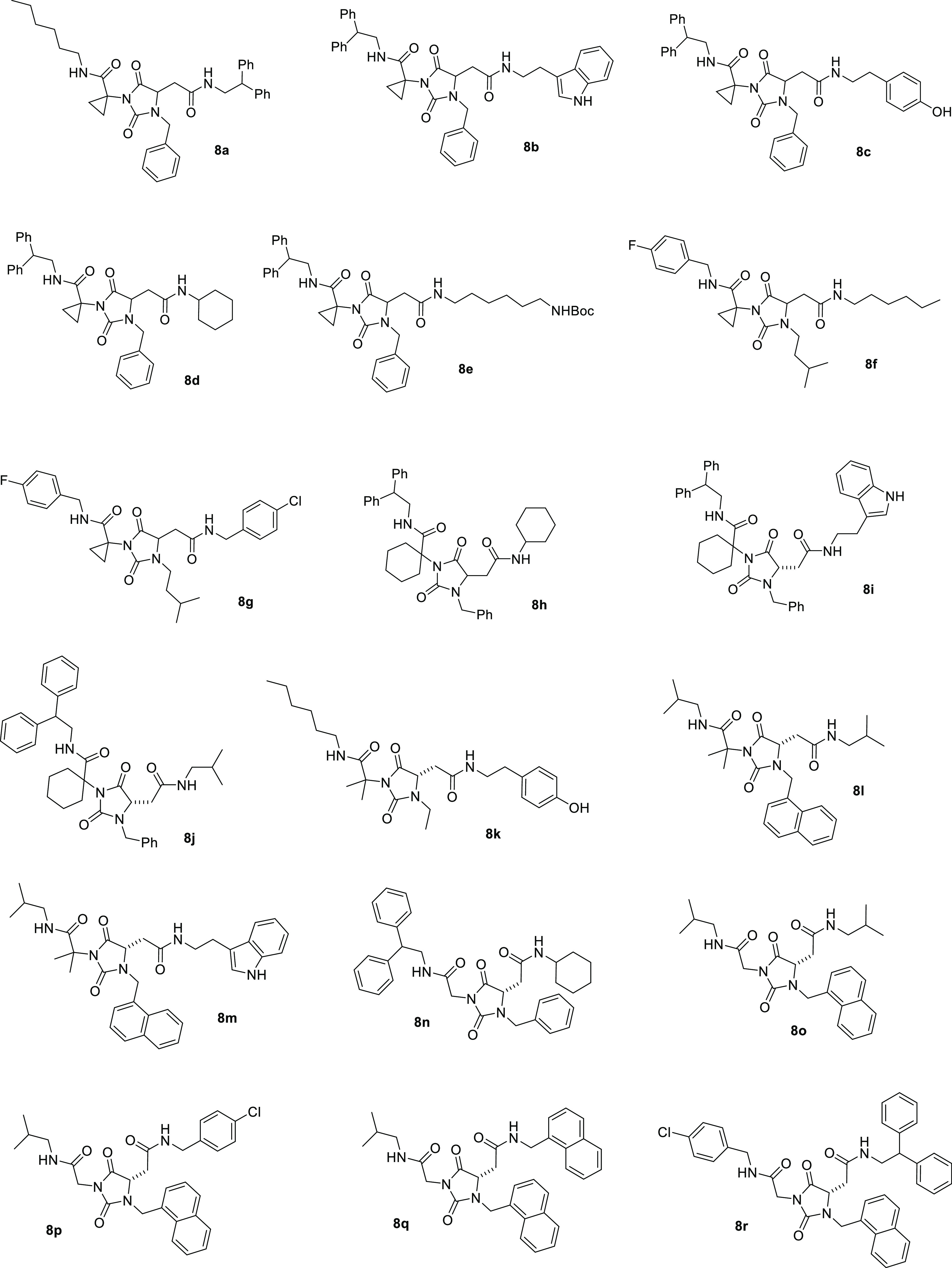
Structures of Hydantoin Universal Peptidomimetics **8**

Accordingly, we recovered in good yield and excellent purities a collection of 18 hydantoin-based universal peptidomimetics **8a**–**r** (Chart 1) having different types of substituents, from highly hydrophobic to polar, with a synthetic protocol based in operationally simple and time-saving liquid–liquid acid/base extractions which did not require further chromatographic purification, thus suitable for combinatorial synthesis/high-throughput screening programs. It is worth noting that the key substituents R^1^, R^2^, and R^3^ have been introduced using simple, commercially available aldehydes (R^1^) and amines (R^2^, R^3^) and easy conventional synthetic protocols (reductive amination and coupling reactions, respectively), which render the new synthetic pathway designed herein hypothetically conform to introduce all of the natural (and unnatural) amino acid side chains in the key *i* + *n* positions of α-helices or β-turn.

### Conformational Analysis *In Silico*: Computation

**Figure 1:**
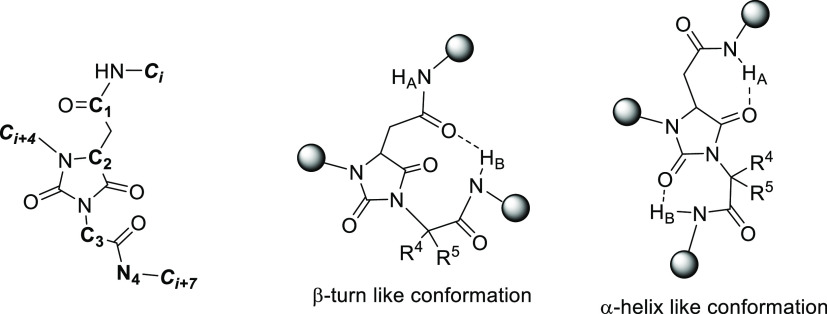
β-Turn- and β-helix-like conformations of universal peptidomimetics **8**.

The propensity of molecules **8a**–**r** to adopt a defined secondary structure was investigated by computational tools. The structures were first submitted to a conformational search by a combined Monte Carlo–Molecular Mechanics (MM) approach. For each structure, the conformers within 10 kcal/mol from the minimum were considered. Different parameters were measured to establish whether an α-helix or β-turn conformation was present, according to the literature (Figure 1).^17^ The three atoms C*i*, C*i* + 4 and C*i* + 7 were related to the *i*, *i* + 4 and *i* + 7 residues of an ideal α-helix, and the ideal interatomic distances *i* – *i* + 4 = 6.2 Å, *i* – *i* + 7 = 10.3 Å and *i* + 4 – *i* + 7 = 5.8 Å were used as reference. For the β-turn conformation, the interatomic distance *d*α < 7 Å and the absolute value of the dihedral angle C1–C2–C3–N4 β < 60° were considered as a condition. The presence of the intramolecular 10-membered ring H-bond was also evaluated. We also considered the less common 3_10_ helix motif, and we found that the compounds **8a**–**r** can efficiently mimic this structure by placing the substituents in coincidence with the *i*, *i* + 2 and *i* + 4 residues of the helix. Results are reported as percentage of conformers meeting the requirements (Table 2 and Figure S1).

**Table 2:** Results from Monte Carlo/MM Conformational Analysis^a^

compound	β-turn (%)	α-helix (%)	3_10_-helix	global minimum
**8a**	23	52	11	α-helix
**8b**	7	66	19	α-helix
**8c**	9	63	11	α-helix
**8d**	18	50	19	α-helix
**8e**	44	54	35	β-turn
**8f**	10	51	10	α-helix
**8g**	23	0	0	n.d.
**8h**	14	52	5	α-helix
**8i**	15	48	7	n.d.
**8j**	14	61	10	α-helix
**8k**	1	60	1	α-helix
**8l**	46	30	4	β-turn
**8m**	23	46	10	α-helix
**8n**	11	43	15	α-helix
**8o**	18	34	9	β-turn
**8p**	39	23	5	β-turn
**8q**	21	34	13	β-turn
**8r**	12	44	11	α-helix

a Results are reported as a percentage of conformers meeting the geometrical requirements for β-turn, α-helix, and 3_10_-helix.

**Figure 2:**
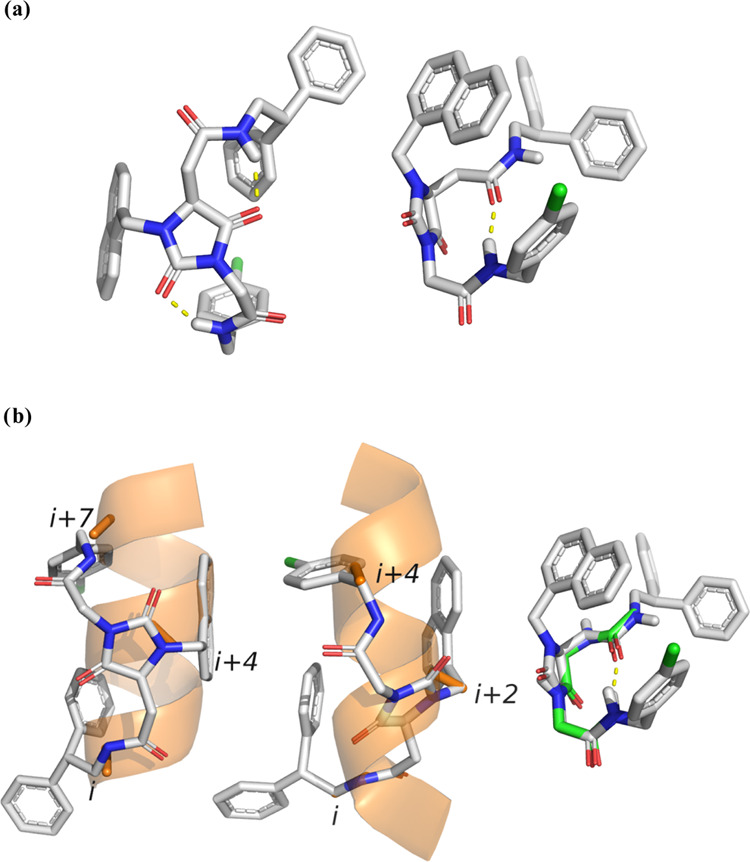
(a) α-Helix (left)- and β-turn (right)-like conformations for representative compound **8r**; (b) superimposition of **8r** conformers with an α-helix model in orange (left), 3_10_-helix model in orange (center), and β-turn in green (right). For the α-helix, the relevant *i*, *i* + 4, and *i* + 7 positions are highlighted.

β-Turn is described by the presence of the distinctive intramolecular hydrogen bond involving the hydrogen NH_B_ and forming a 10-membered ring, whereas the α-helix structure is established by the presence of a two consecutive γ-turn type hydrogen bonding (both NH_A_ and NH_B_ are involved) around the hydantoin central core (Figure 1). In general, the α-helix conformation is preferred because of the higher percentage of conformers adopting this structure for each compound. Looking at the global minimum conformers, the β-turn geometry is the most favored in particular for compounds **8o**–**q** having two hydrogen atoms on C3 carbon (R_4_ = R_5_ = H, Figure 1), while for the *gem*-dimethyl **8k**–**m** and cycloalkyl derivatives **8a**–**j**, the α-helix is preferred. This last result is in line with that obtained in the previous work for 3-*cyclo*-butylcarbamoyl hydantoins.^23^ For selected molecules **8a**, **8m**, **8o**–**r**, a density functional theory (DFT) study was performed at the B3LYP6-311G(d,p) level. For each structure, the first lower-energy α-helix and β-turn conformers were optimized in vacuo. All of the energies were corrected for the zero-point energy (ZPE). Unlike that obtained from MM force field, results from DFT showed that the most stable secondary structure is the α-helix. This difference can be ascribed to the more accurate calculation of the energy from DFT with respect to molecular mechanics. The energy gaps between the α-helix and the β-turn conformers varied from 0.17 kcal/mol (**8r**) to 5.05 kcal/mol (**8m**). It can be then assumed that in most cases the herein proposed scaffolds can access both the α-helix and the β-turn secondary structures according to the environment. For compound **8r** the energy of the β-turn structure found by X-ray single-crystal analysis (*vide infra*) was also calculated without further optimization, resulting to be 2.85 kcal/mol higher than the β-turn conformer as obtained from conformational analysis and DFT optimization, indicating a decisive role of the crystal packing in determining the conformation of the residues around the β-turn core. Superimposition of the β-turn conformation with theoretical β-turn models revealed the ability to mimic preferentially the type II β-turn (see root-mean-square deviation (rmsd) values of the backbone in Table 3 and Figure 2). Acceptable rmsd values have been measured also for the type I′ β-turn. The type I and II turns mostly differ in the orientation of the central amide bond. The presence of the hydantoin core allows for the good superimposition of the two orientations thanks to the presence of the two carbonyls of the imide moiety (Figure 2). A comparison within the investigated molecules seems to indicate that the presence of the two hydrogen atoms on C3 carbon is favorable in stabilizing the type II β-turn, with compounds **8o**–**r** having the lowest rmsds.

**Table 3:** Results from DFT Studies

	relative energy (kcal/mol)		rmsd (Å) from superimposition with β-turn models			
	α-helix	β-turn	type I	type II	type I′	type II′
**8a**	0.00	1.81	0.775	0.425	0.585	0.263
**8m**	0.00	5.05	0.657	0.268	0.518	0.442
**8o**	0.00	2.58	0.591	0.172	0.490	0.381
**8p**	0.00	2.48	0.665	0.183	0.405	0.451
**8q**	0.00	4.14	0.634	0.119	0.518	0.433
**8r**	0.00	0.18	0.701	0.169	0.412	0.567
**8r**^a^	0.00	3.03	0.621	0.221	0.380	0.570

a Single point energy of the structure obtained from single-crystal X-ray analysis.

### Conformational Analysis in Solution: NMR, Circular Dichroism (CD), Fourier Transform Infrared (FTIR)

To investigate the forces that stabilize the secondary structure, we relied on the behavior of the intramolecular hydrogen-bond network of our molecules in solution. Two patterns were identified in our previous work: (1) the α-helix is stabilized by two H-bonds involving both carbonyls on the hydantoin ring and both H_A_ and H_B_, while (2) the β-turn conformation is stabilized by another H-bond not involving any hydantoin-ring carbonyl and only amide NH_B_.^24^ An important parameter to assess the presence of hydrogens involved in hydrogen bonds is the chemical shift in a relatively nonpolar solvent such as CDCl_3_, according to which higher values, typically around 8 ppm, are more related to an internal hydrogen bond. The value of the chemical shifts of the amidic protons H_A_ and H_B_ of those compounds for which it was possible to record the ^1^H NMR spectrum in deuterated chloroform (2.5 mM solutions)^30^ are reported in Table 4.

**Table 4:** ^1^H NMR Data for Hydantoin-Based Universal Mimetics **8**^a^

compound	δ N–H_B_ (ppm)^b^	δ N–H_A_ (ppm)^b^	Δδ/Δ*T*N–H_B_(ppb/K)^c^ CDCl_3_	Δδ/Δ*T*N–H_A_(ppb/K)^c^ CDCl_3_	Δδ/Δ*T*N–H_B_(ppb/K)^c^ DMSO-*d*_6_	Δδ/Δ*T*N–H_A_(ppb/K)^c^ DMSO-*d*_6_
**8a**	8.03	5.34				
**8d**	8.12	5.03				
**8h**	7.76	5.78				
**8i**	8.16	5.41				
**8j**	7.71	5.89				
**8n**	7.72	5.42				
**8o**	7.95	4.99	22.2	9.1		
**8p**	8.05	6.39	40.1	21.3	4.2	3.8
**8q**	8.57	5.41				
**8r**	8.40	4.84	20.6	10.6	3.5	6.1

a NMR experiments performed in 2.5 mM solutions.

b NMR spectra recorded in CDCl_3_.

c Absolute values.

H_B_ protons resonate at lower field than H_A_ protons in all cases, most of them at ppm higher than 8, indicating their involvement in the formation of hydrogen bonds. Moreover, the rates of H_B_/D exchange in ^1^H NMR spectra recorded in CD_3_OD were very slow for compound **8k**, **8l**, **8o**. Indeed, the spectra of **8k** and **8l** showed the presence of the amide proton H_B_ at 8.01 and 8.10 ppm, respectively, integrating for one hydrogen, thus indicating that no exchange occurred, while for **8o** there is a peak at 8.16 ppm integrating for 0.25 meaning that only a 75% of hydrogens H_B_ underwent H/D exchange (see spectra in the Supporting Information). In all spectra recorded in CD_3_OD, we did not detect the presence of a peak for hydrogen H_A_ which exchanges quickly with deuterium.

To study more in-depth the presence of these interactions for the more flexible peptidomimetics, namely, the glycyl derivatives having two hydrogen atoms on C3 carbon, we performed variable-temperature (VT) ^1^H NMR analysis on compounds **8o**, **8p**, **8r** in CDCl_3_ and compounds **8p**, **8r** in dimethyl sulfoxide (DMSO)-*d*_6_ 2.0 mM solutions. In the relatively nonpolar solvent CDCl_3_, low-temperature coefficient values, typically lower than 2.4 ppb/K, are not always very indicative since they can be attributed either to shielded protons or to accessible ones. On the contrary, values significantly larger than 2.4 ppb/K can be assigned to NH protons involved in intramolecular hydrogen bonds which become accessible to the solvent upon increasing temperature.^31^ The Δδ/Δ*T* values obtained for H_B_ of peptidomimetics **8o**, **8p**, **8r** are in all cases very high (22.2, 40.1, and 20.6 ppb/K, respectively, Table 4 and Figures S2–S4) suggesting the involvement of the amide proton in intramolecular hydrogen bonding that is disrupted with the temperature. Interestingly, also the values for H_A_ are high, although to a lesser extent (Table 4 and Figures S2–S4). This could be explained by the possible involvement of the latter amide proton in less strong hydrogen bond as expected in an α-helix conformation. These results corroborate the possibility for the more flexible glycine peptidomimetics to exist in solution as an equilibrium between β-turn conformation triggered by an intramolecular hydrogen bond involving H_B_ and α-helix conformation stabilized by two intramolecular hydrogen bonds involving both H_B_ and H_A_, being the first the preferred one. In very polar solvents, such as DMSO, typically, solvent-accessible protons exhibit a Δδ/Δ*T* > 5 ppb/K, whereas Δδ/Δ*T* < 5 ppb/K denotes protons involved in H-bonds.^32^ The value found in **8r** for NH_B_ is smaller than 5 ppb/K and smaller than that found for NH_A_ (Table 4 and Figure S6), indicating a preference of NH_B_ to be involved in intramolecular H-bonding. Interestingly, for molecule **8p**, both values are below the 5 ppb/K Δδ/Δ*T* threshold (Table 4 and Figure S5), the value for H_A_ being slightly lower than that of H_B_, suggesting that both amide hydrogens are involved in H-bonds. These results suggest a stronger preference to adopt a folded conformation (β-turn, only NH_B_ H-bond is present) for **8r** and a slight preference for **8p** to an open conformation (α-helix, both NH_A_ and NH_B_ are hydrogen-bonded).^33^ The extent of H-bonding can be evaluated also by performing DMSO titration experiments. DMSO is added in small aliquots (5 μL) gradually to diluted CDCl_3_ solutions (2.0 mM) of the compounds of interest. A ^1^H NMR spectrum is then recorded after each addition, and the chemical shift of the H-bonded protons is plotted against the DMSO aliquots. H-bonding generally results in deshielding, and an increasingly downfield-shifted proton resonance indicates increased H-bond strength.^34^ As the concentration of DMSO increases, the resonance line shifts downfield as a result of increasing H-bonding interactions with DMSO. In contrast, only small changes are seen for the protons involved in intramolecular H-bonding, thus indicating that DMSO cannot compete with the proposed intramolecular H-bond.

**Figure 3:**
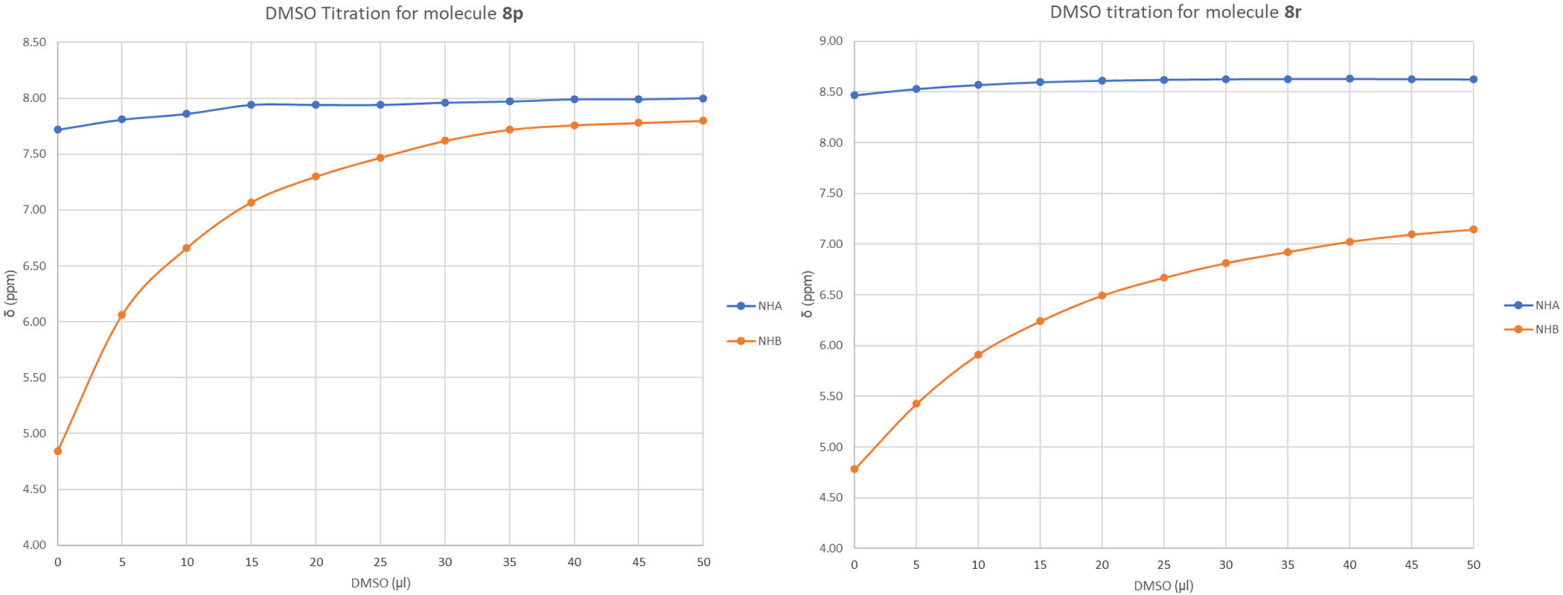
DMSO titration experiments on substrates **8p**, **8r**.

We performed DMSO titration experiments on compounds **8p**, **8r**, and the results are shown in Figure 3 (see also Figures S7 and S8, respectively). As can be seen in Figure 3, the chemical shifts of H_B_ in compounds **8p**, **8r** essentially did not change upon dilution with DMSO, whereas we observed a clear increase of the chemical shifts of amidic hydrogens H_A_, indicating the presence of a strong intramolecular hydrogen bond involving only the amide proton H_B_ in all compounds. All of the observations obtained with the NMR experiments seem to underline a preference of the β-turn conformation in solution. However, two-dimensional (2D) nuclear Overhauser enhancement spectroscopy (NOESY) experiment on glycine derivatives **8o**–**r** did not show evident long-range contacts supporting the possibility that no preferential conformation is adopted.^14^

**Figure 4:**
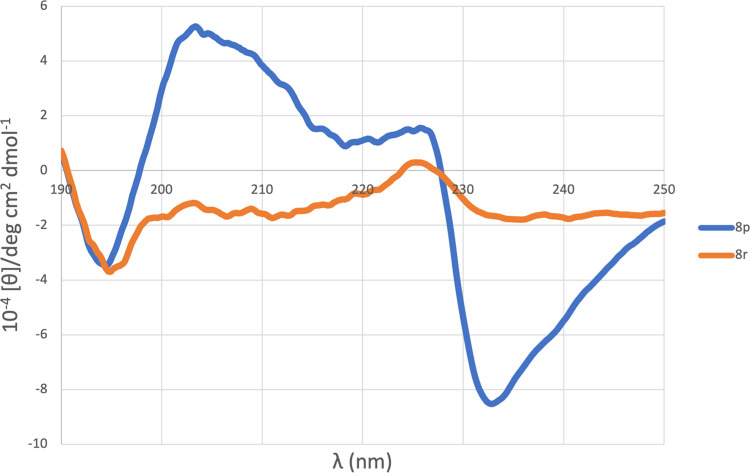
CD spectra for peptidomimetics **8p**, **8r**.

The ability of universal peptidomimetic **8p**, **8r** to adopt secondary structure in solution was assessed also by CD spectroscopy. Figure 4 shows the spectra recorded in methanol (10^–5^ M solutions)

The CD profile of compound **8r** has a negative absorption band at 194.9 nm and a second positive band centered at 225.4. This profile can be addressed to a right β-turn in accordance with those reported in the literature.^36^ The results for molecule **8p** are even more interesting. In addition to having the typical characteristics of a right β-turn as described above (negative band at 194.4 nm, positive band at 225.7 nm) the spectrum has two additional absorption bands, a positive one at 203.4 nm and a negative one at 232.8 nm. This phenomenon reflects a possible contribution from aromatic residues π-stacking interactions,^37^ further stabilizing our proposed β-turn conformation.

**Figure 5:**
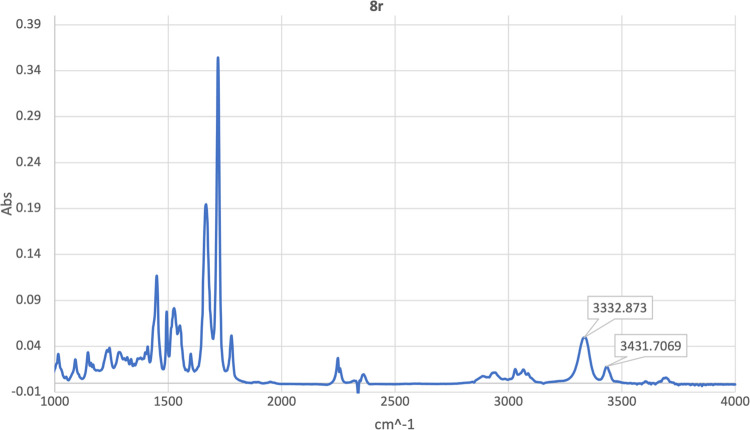
ATR-FTIR spectrum of **8r**.

Finally, we registered the attenuated total reflection (ATR)-FTIR spectrum of peptidomimetic **8r**, which was the most soluble in chloroform, as model compound for the flexible glycine hydantoin-based universal peptidomimetics (Figure 5). The spectrum was run by depositing a thin layer of **8r** in diluted chloroform solution (20 mg/mL) between two quartz plates. Based on literature data, the band at 3431 cm^–1^ was assigned to the free NH groups, and a second one, more intense, appearing at 3332 cm^–1^ to NH groups involved in an intramolecular hydrogen bonding.^38^ These findings show that, in solvents of low polarity such as chloroform, secondary structures promoted by intramolecular hydrogen bonds significantly populate the conformational equilibrium of the molecule in solution.

### Conformational Analysis in Solid State: X-ray

Single crystals of **8r** were obtained by the slow evaporation of a 1:1 water/acetone solution, after 1 week. Crystallographic data and refinement details are given in Table 5.

**Table 5:** Crystal Data and Structural Parameters of Compound **8r**

Crystal Data	
chemical formula	C_39_H_35_ClN_4_O_4_
*M*_r_	659.16
crystal system, space group	monoclinic, *C*2
*a*, *b*, *c* (Å)	33.471(7), 9.5152(19), 10.877(2)
β (deg)	99.48(3)
*V* (Å^3^)	3416.9(12)
*Z*	4
*F*(000)	1384
density (g/cm^3^)	1.281
temperature (K)	298(2)
radiation type	Mo Kα (λ = 0.71073 Å)
μ (mm^–1^)	0.159
crystal size (mm)	0.06 × 0.05 × 0.03

Data Collection	
diffractometer	Bruker Apex II CCD
no. of measured, independent, and observed [*I* > 2σ(*I*)] reflections	6841, 3544, 3043
*R*_int_	0.0682

Structure Refinement	
*R*, w**R**^2^, *S*	0.0637 [*I* > 2σ(*I*)] and 0.0772 [all], 0.1557 [*I* > 2σ(*I*)] and 0.1653 [all], 1.032 [all]
no. of parameters	433
no. of restraints	1
Δρ_max_, Δρ_min_(e/Å^3^)	0.235, −0.214

**Figure 6:**
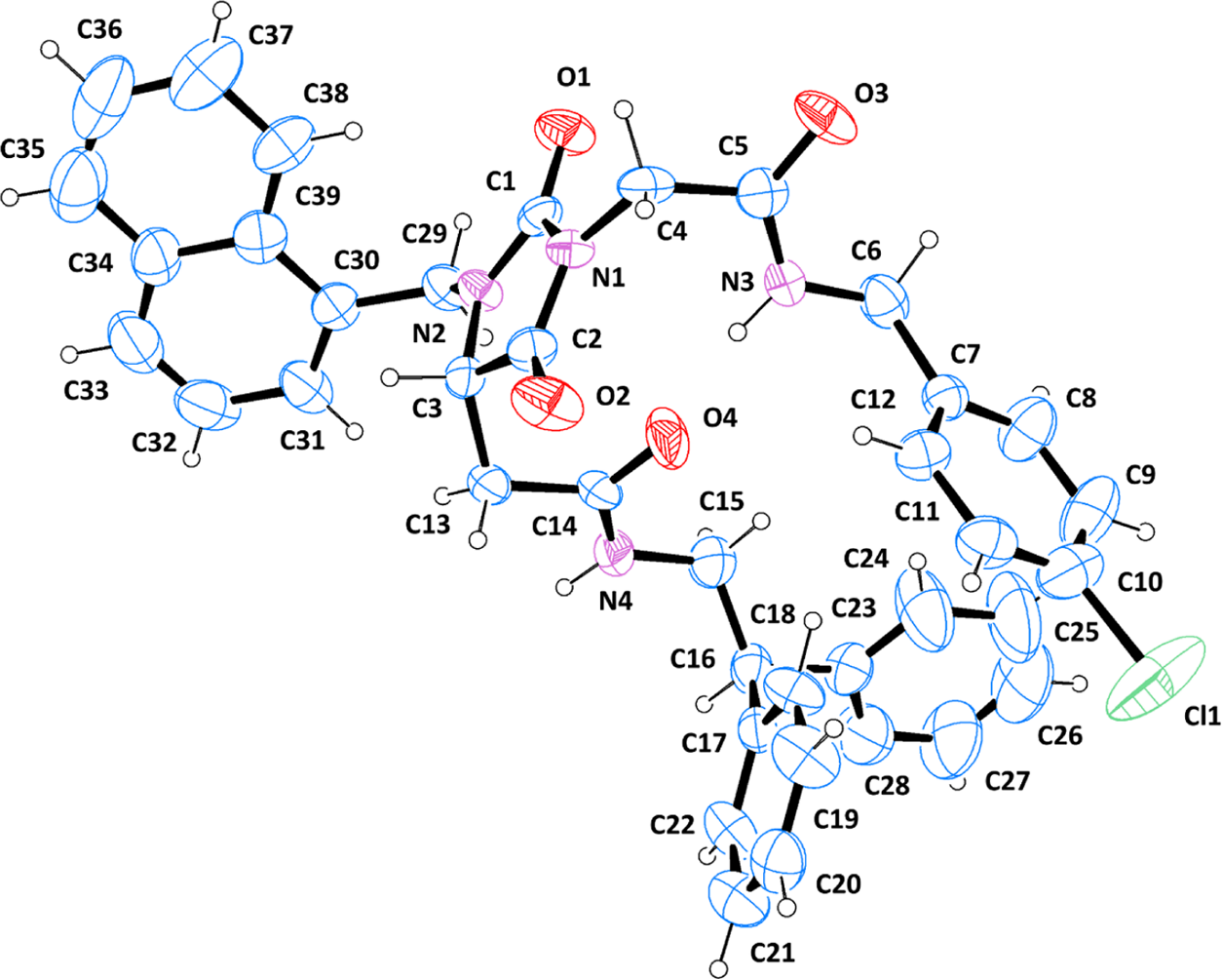
ORTEP diagram of **8r**, with the arbitrary atom-numbering scheme. Thermal ellipsoids are drawn at the 40% probability level.

Compound **8r** crystallized in the monoclinic space group *C*2; its structure is shown in Figure 6 as an Oak Ridge thermal ellipsoid plot (ORTEP) diagram,^39^ indicating the arbitrary atom-numbering scheme used in the following discussion.

Notably, in solid state, the molecule adopts a β-turn conformation stabilized by an intramolecular hydrogen bond that persisted during the crystal formation even under the presence of a highly competitive protic solvent (H_2_O) in the crystallization solution.

The molecular structure of **8r** is characterized by a hydantoin nucleus, substituted at the two nitrogen atoms and at the C3 carbon. The hydantoin ring is planar, with a maximum deviation of 0.032(4) Å (C2) from the best mean plane. The substituents at N1 and C3 extend toward the same direction, with torsion angles of −61.2(9) and 97.2(9)° for C2–C3–C13–C14 and C2–N1–C4–C5, respectively. The divergence between the two values is motivated by the different position of the methylene group bridging the hydantoin nucleus and the amide. While C13 is linked to an asymmetrical carbon (in the *S* configuration), C4 is directly attached to N1 and, thus, remains coplanar with the central ring. The same can be said for C29, which connects N2 to the naphthalene; in this case, the aromatic moiety is oriented in the opposite direction with respect to the other two substituents, with a torsion angle of −131.6(7)° for C1–N2–C29–C30. Both the naphthalene ring and the *p*-chlorophenyl moiety are almost perpendicular to the hydantoin nucleus; in detail, the former forms an angle of 80.3° with the central heterocycle, while the latter is inclined at 86.6°.

**Figure 7:**
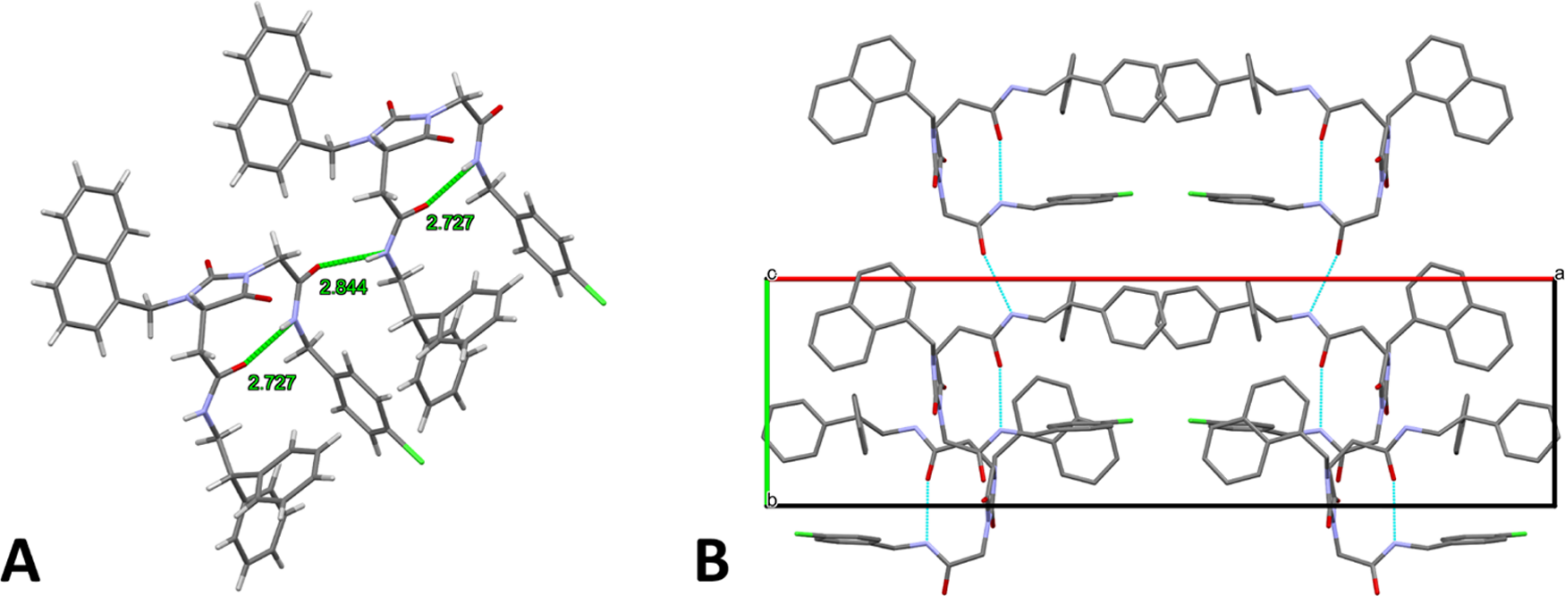
(A) Stick model of **8r** in an arbitrary orientation, evidencing the main H-bonds. (B) Stick model showing the crystal packing along the *c* axis. Hydrogen atoms are omitted for the sake of clarity.

The crystal packing (Figure 7) is mainly ensured by two strong H-bonds, one of them, being intramolecular and involving amidic NH_B_ (N3–H3 in the ORTEP diagram), responsible for the adopted β-turn conformation. In detail, the former intramolecular contact (N3–H3N···O4) links the two elongated hydantoin substituents (D···A: 2.727(9) Å; D–H···A: 1.965(6) Å; D–H···A: 147.2(5)°), while the other, which is intermolecular (N4–H4N···O3^I^, ^I^ at *x*, *y* – 1, *z*), connects the **8r** molecules (D···A: 2.843(9) Å; D–H···A: 2.001(6) Å; D–H···A: 166.1(5)°) forming a chain. Weak-to-very-weak intramolecular and intermolecular C–H···O contacts and a C–H···Cl interaction contribute to stabilize the network. A complete account of the H-bonds is provided in the Supporting Information (Table S1). Despite the considerable number of aromatic moieties, the packing is not significantly influenced by stacking interactions; the only exception is a very weak, almost parallel π–π contact between two phenyl groups of the benzhydryl moieties of two adjacent molecules (centroid–centroid distance: 4.079 Å; angle between planes: 8.0°). However, numerous C–H···π interactions are established between the various aromatic rings.

The Hirshfeld surface (HS) of the **8r** was mapped over the normalized contact distance (*d*_norm_), according to the following equation
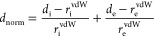
where *d*_i_ is the distance between the HS and the nearest nucleus inside the surface, *d*_e_ is the distance between the HS and the nearest nucleus outside the surface, and *r*^vdW^ represents the van der Waals radius of the atom. Details of the HS are provided in Table 6.

**Table 6:** Characteristics of the HS Generated for **8r**

**8r**	*V* (Å^3^)	*A* (Å^2^)	*G*	Ω
HS	845.05	641.95	0.673	0.191

**Figure 8:**
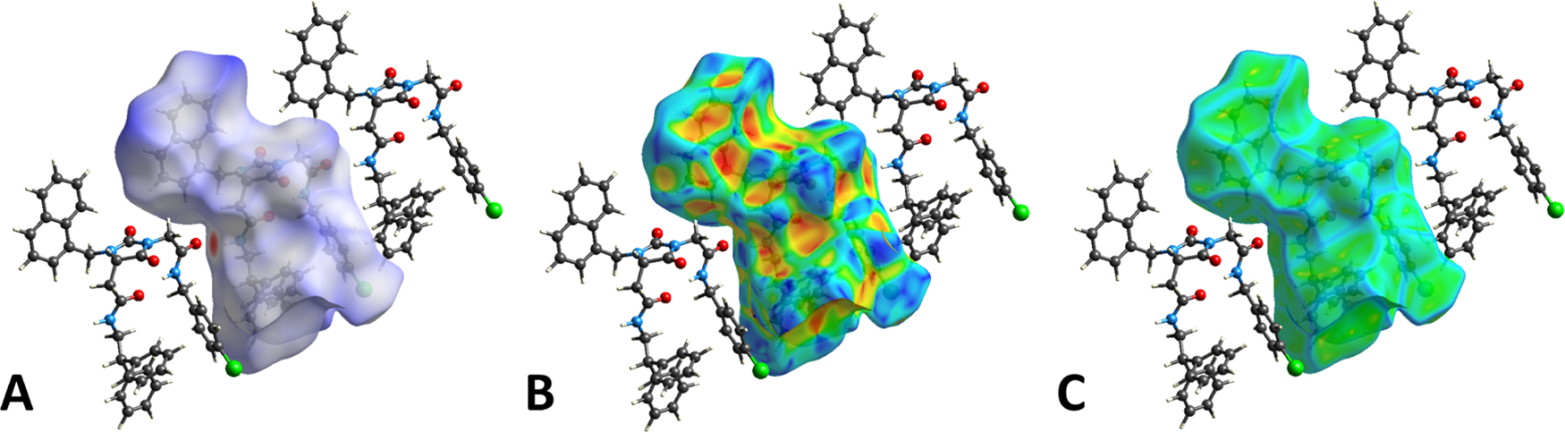
(A) HS mapped over *d*_norm_ with a fixed color scale in the range −0.5808 au (red) to 2.9223 au (blue), based on the length of the intermolecular contacts with respect to the sum of the van der Waals radii (red: shorter; blue: longer; white: same). (B) HS mapped over the shape index (color scale: −0.9973 to 0.9977 au). Blue areas represent bumps, and red regions indicate hollows. (C) HS mapped over the curvedness (color scale: −4.4169 to 0.8187 au). Green represents flat regions, and blue indicates edges.

The *d*_norm_ property was visualized with a red-blue-white color scheme, based on the length of the intermolecular contact with respect to the sum of the van der Waals radii (Figure 8A). The inspection revealed a rather unperturbed surface, with only two major red spots corresponding to the short-range H-bond connecting the amide groups of adjacent molecules. Additional small and very feeble spots indicate the presence of minor C–H···O interactions and a weak C–H···Cl contact. The surface mapped over the shape index (Figure 8B) confirmed the absence of strong π–π stacking interactions; the only notable feature is the presence of deep hollow regions, indicated by the red color, especially in the vicinity of the benzhydryl substituent. Finally, the curvedness plot (Figure 8C) showed the absence of large flat areas, despite the abundance of aromatic moieties, further confirming the marginal contribution of stacking interactions to the overall packing.

**Figure 9:**
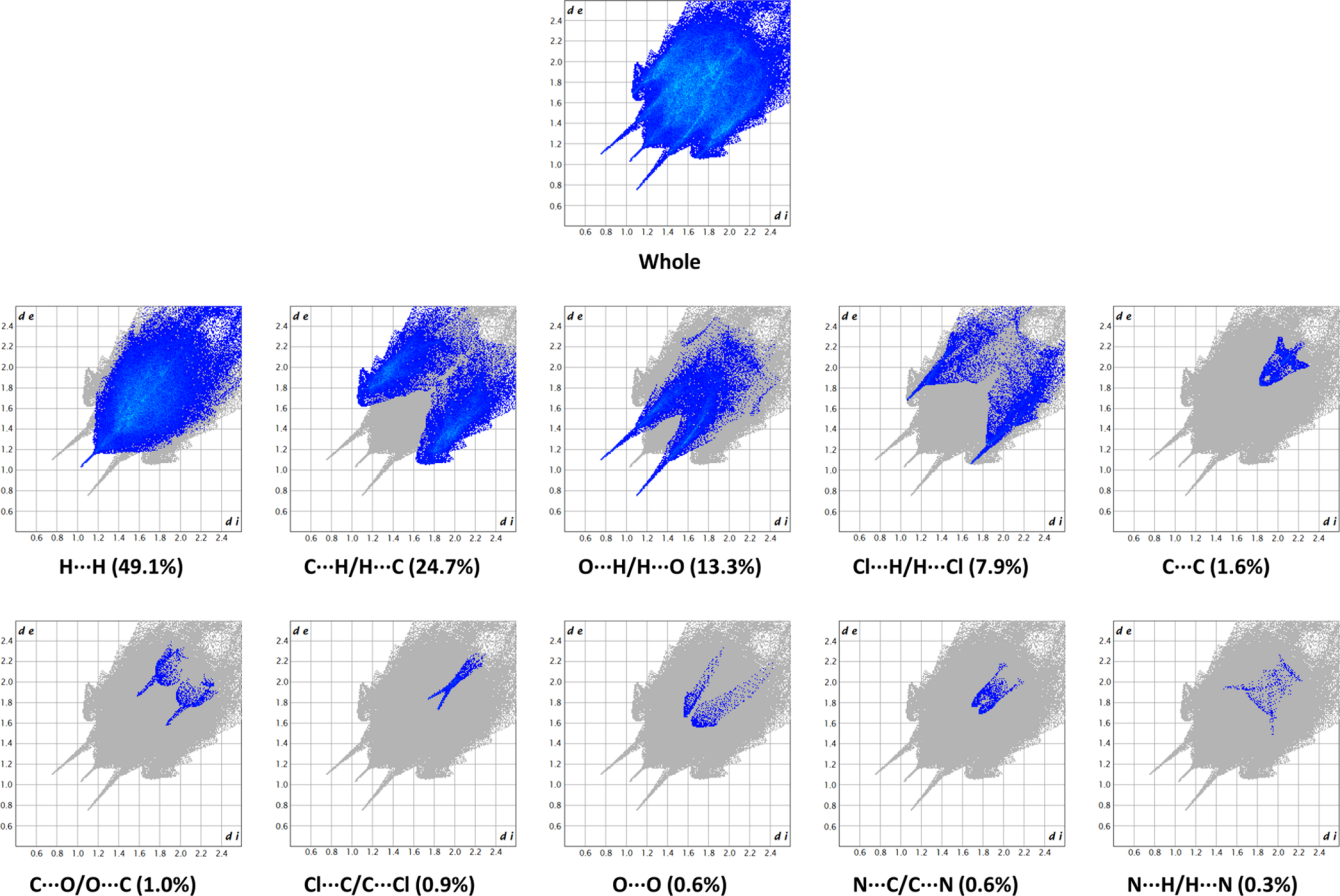
Two-dimensional Fingerprint plots of HS, providing a visual summary of the frequency of each combination of *d*_e_ and *d*_i_ across the HS. Points with a contribution to the surface are colored blue for a small contribution to green for a great contribution.

The two-dimensional (2D) fingerprint of the HS (Figure 9), providing a visual summary of the contribution of each contact type and the relative area of the surface corresponding to it, revealed a prominence of nonspecific van der Waals H···H contacts (49.1%). The wings of the plot are occupied by C···H/H···C interactions (24.7%), which include the numerous, weak C–H···π H-bonds. O···H/H···O contacts (13.3%) also offer a significant contribution to the surface, representing the strong H-bonds that interconnect the molecules. Cl···H/H···Cl interactions (7.9%) occupy an appreciable portion of the surface, without being particularly relevant for the crystal network. Finally, C···C contacts (1.6%), forming the characteristic arrow-shaped region at the center of the plot, only marginally contribute to the surface. The remaining interactions are negligible and are indicated in Figure 8. These observations were supported by the analysis of the contact enrichments (Table 7).^39^ The calculations showed that Cl···H/H···Cl, O···H/H···O, and C···H/H···C interactions are enriched (*E_XY_* ≥ 1) with respect to the corresponding random contacts.

**Table 7:** Analysis of the Intermolecular Contacts on the HS of **8r**, According to Jelsch et al.^40^^,^^a^

atoms	H	C	N	O	Cl
surface (%)	72.2	15.2	0.5	7.8	4.4
contacts (%)					
H	49.1				
C	24.7	1.6			
N	0.3	0.6	0.0		
O	13.3	1.0	0.0	0.6	
Cl	7.9	0.9	0.0	0.0	0.0
enrichments					
H	0.9				
C	**1.1**	0.7			
N					
O	**1.2**	0.4			
Cl	**1.2**	0.7			

a The first part of the table gives the surface contribution *S*_*X*_ of each chemical type *X* to the Hirshfeld surface. The second part shows the proportions of the actual contacts (*C*_*XY*_), and the third part indicates the enrichment ratios (*E*_*XY*_) of the various contact types. Reciprocal contacts X···Y and Y···X are merged. *E*_*XY*_ were not computed when the random contacts (*R*_*XY*_) were lower than 0.9%. *E*_*XY*_ ratios larger than unity indicate enriched contacts (in bold), while those lower than unity are impoverished. The percentages of actual contacts were calculated using CrystalExplorer21.5.

## Conclusions

In conclusion, we have conceived a new scaffold for universal peptidomimetics based on hydantoin ring. The synthetic strategy for these compounds is based on a chemoselective domino condensation/cyclization process between α-aminoester isocyanates and *N*-alkyl aspartic acid diesters which occurs in mild condition (room temperature) followed by conventional deprotection/coupling reactions. All of the intermediates were recovered through time-saving liquid–liquid acid/base extraction procedures in pure form, thus not needing any further chromatographic purification. We synthesized in this way a collection of 18 enantiomerically pure, systematically substituted hydantoins having either different quaternary carbons at the exo-C_3_ position (*cyclo*-hexyl, *cyclo*-propyl, *gem*-dimethyl) or a more flexible methylene group. It is worth noting that a wide range of natural or unnatural amino acid side chains could be incorporated since they come from easily accessible aldehydes (R^1^) and amines (R^2^ and R^3^). All of these characteristics render the synthetic strategy presented particularly suitable for combinatorial synthesis/high-throughput screening programs. The conformational behavior of the peptidomimetics was studied *in silico*, by molecular modeling, in solution, by NMR, CD, and IR experiments, and in solid state through X-ray analysis. Molecular modeling showed that these scaffolds can adopt kinetically and thermodynamically accessible, intramolecular hydrogen-bond-driven conformations where the key substituents are projected in positions superimposable to some of the key *i*, *i* + *n* side chains of protein secondary structures such as α-helix and β-turn, the first one being the favored for most of the scaffolds, either for those having a quaternary exo-carbon or for the more flexible glycine derivatives. On the contrary, VT-NMR experiments recorded in nonpolar CDCl_3_ and polar DMSO-*d*_6_ and DMSO titration NMR experiments, both performed on the more flexible molecules having two hydrogens bonded to the C-3 carbon (glycine derivatives **8o**–**r**) evidenced the presence in solution of a stronger intramolecular hydrogen bond engaging amide NH_B_, which is responsible for both α-helix and β-turn conformations, compared to that involving NH_A_ (responsible for the only α-helix conformation), suggesting the presence of a more populated β-turn conformation. Analogously, X-ray analysis showed that glycine derivative **8r** adopts in solid state a well-defined β-turn conformation with the presence of the characteristic intramolecular hydrogen bond between NH_B_ and the carbonyl not belonging to the hydantoin ring. All of these features demonstrate that hydantoin scaffolds **8** can be considered a novel class of universal peptidomimetics able to adopt common protein secondary structures with favorable enthalpic and entropic profiles.

## Materials and Methods

### Materials

Commercially available reagent-grade solvents were employed without purification. Thin-layer chromatography (TLC) was run on silica gel 60 F254 Merck. Visualization of the developed chromatogram was achieved with UV light and ceric ammonium molybdate (CAM) or ninhydrin stains. Flash chromatography (FC) was performed with silica gel 60 (60–200 μm, Merck). ^1^H, and ^13^C NMR spectra were run at 400 or 500 MHz. Chemical shifts are expressed in ppm (δ), using tetramethylsilane (TMS) as internal standard for ^1^H and ^13^C nuclei (δH and δC = 0.00). Electrospray ionization (ESI) mass spectra were performed by a Bruker Esquire 3000+ instrument equipped with an MS detector composed of an ESI ionization source and a single-quadrupole mass selective detector or by an Agilent Technologies 1200 series high-performance liquid chromatography (HPLC) system equipped with a DAD and a 6120 MS detector composed by an ESI ionization source and a single-quadrupole mass selective detector. Optical rotations were measured on a Propol Digital Polarimeter with a sodium lamp. Isocyanates **2a**–**d** were synthesized according to the procedure described in ref (41) and suddenly used in the next step, while *N*-alkyl α-aminoester **3** was synthesized according to ref (42). CD spectra were recorded on a Jasco J-1000 CD spectrometer at 25 °C, *V*_scan_ = 10 nm/min, DIT = 4 ms, three repetitions per sample, within a range of wavelength between 190 and 250 nm. The blank was preliminarily recorded and subtracted from the sample at each repetition. ATR-FTIR spectrum was run on a Jasco FT/IR-610. X-ray diffraction data for **8r** were collected on a Bruker Apex II CCD three-circle diffractometer working at room temperature with graphite-monochromatized Mo Kα radiation (λ = 0.71073 Å). X-ray data were acquired in the θ range of 2–21° recording four sets of 360 bidimensional CCD frames with the following operative conditions: omega rotation axis, scan width 0.5°, acquisition time 50 s, sample-to-detector distance 50 mm, ϕ angle fixed at four different values (0, 90, 180, and 270°) for the four different sets. Omega rotation frames were processed using the SAINT software^43^ for data reduction (intensity integration, background, Lorentz, and polarization corrections) and for the determination of accurate unit-cell dimensions, obtained by least-squares refinement of the positions of 3623 independent reflections with *I* > 10σ(*I*). Absorption effects were empirically evaluated by the SADABS software,^44^ and an absorption correction was applied to the data. The structure was solved by direct methods with SIR-2019^45^ and completed by iterative cycles of full-matrix least-squares refinement on *F*_o_^2^ and Δ*F* synthesis using the SHELXL-2018/3 program in the WinGX v2021.3 suite.^46^ The hydrogen atoms were included at geometrically calculated positions and refined using a riding model. Uiso(H) was defined as 1.2Ueq of the parent atoms for phenyl, methylene, and methine residues. The structure was analyzed using PARST^47^ and Mercury 2021.3.^48^ Graphical representations were generated with ORTEP-3 2020.1,^39^ Mercury,^48^ and CrystalExplorer 21.^49^

### Computational Details

Conformational analysis was performed with the software Spartan’08^50^ by means of the “conformer distribution” function, using the Monte Carlo search method. For each compound, a variable number of 900–400 conformers were generated, according to the structure. The MMFF force field in vacuo was used for the energy minimization of the found structures. The structures were then clustered according to the default setting of the software (which consists in pruning out higher energy conformers and keeping a diverse set of the low-energy conformers using the RMS-torsion definition of nearness). Full geometry optimization of selected lowest-energy conformations was then performed with DFT at the B3LYP 6-311G (d,p) level in vacuo with the software Gaussian’09.^51^ All energies were corrected by adding the ZPE as obtained by frequency calculation at the same level.

#### General Procedure for the Synthesis of Urea Intermediates **4**

To a solution of isocyanate **2** (0.5 mmol, 1 equiv) in DCM (0.3 M solution), a solution of TEA (2 equiv) and *N*-alkyl α-aminoester **3** (1.2 equiv) in DCM (0.3 M solution) was added at 0 °C. After 1 h, the reaction was quenched with a 1 N HCl aqueous solution, the temperature raised to rt, and the mixture was extracted with DCM. The collected organic phases were washed with a 1 N HCl aqueous solution (once), brine (once), a saturated aqueous solution of NaHCO_3_ (twice), and brine (once). The organic phase was dried over Na_2_SO_4_, filtered, and the solvent evaporated. The urea intermediates **4** were used without any further purification.

##### 4-(*tert*-Butyl)-1-methyl *N*-((1-((Benzyloxy)carbonyl)cyclopropyl)carbamoyl)-*N*-isopentyl-l-aspartate (**4a**)

Yellow oil. Yield 87% (129 mg). *R*_*f*_ (hexane/AcOEt, 70:30) = 0.35; [α]_D_^20^ −15.2 (*c* = 1.0, CHCl_3_); ^1^H NMR (400 MHz, CDCl_3_), δ (ppm) = 5.32–5.30 (m, 5H), 5.41 (br s, 1H), 5.14 (d, *J* = 12.4 Hz, 1H), 5.05 (d, *J* = 12.4 Hz, 1H), 4.52 (t, *J* = 6.8 Hz, 1H), 3.64 (s, 3H), 3.27–3.22 (m, 1H), 3.12–3.07 (m, 2H), 2.56 (dd, *J* = 16.8 and 6.8 Hz, 1H), 1.56–1.44 (m, 5H), 1.44 (s, 9H), 1.67–1.14 (m, 2H), 0.90 (d, *J* = 6.0 Hz, 6H); ^13^C{^1^H} NMR (101 MHz, CDCl_3_), δ (ppm) = 173.0, 171.2, 170.6, 157.6, 135.9, 128.4, 128.0, 127.8, 81.0, 66.8, 57.4, 52.2, 46.4, 37.7, 36.8, 34.8, 28.0, 26.0, 22.4, 17.8, 17.7; ESI-MS: *m*/*z* (%) calcd 490.3 [M]^+^. Found 491.6 [M + H]^+^ (100); anal. calcd for C_26_H_38_N_2_O_7_: C, 63.65; H, 7.81; N, 5.71. Found: C, 63.66; H, 7.81; N, 5.73

##### 4-(*tert*-Butyl)-1-methyl *N*-Benzyl-*N*-((1-((benzyloxy)carbonyl)cyclohexyl)carbamoyl)-l-aspartate (**4b**)

Yellow oil. Yield 83% (112 mg). *R*_*f*_ (hexane/AcOEt, 70:30) = 0.38; [α]_D_^20^ −12.7 (*c* = 0.9, CHCl_3_); ^1^H NMR (400 MHz, CDCl_3_), δ (ppm) = 7.26–7.18 (m, 10H), 5.01 (s, 2H), 4.89 (t, *J* = 6.8 Hz, 1H), 4.80 (br s, 1H), 4.47 (d, *J* = 17.2 Hz, 1H), 4.33 (d, *J* = 17.2 Hz, 1H), 3.55 (s, 3H), 2.95 (dd, *J* = 16.4 and 6.8 Hz, 1H), 2.56 (dd, *J* = 16.4 and 7.2 Hz, 1H), 1.82–1.80 (m, 2H), 1.64–1.62 (m, 2H), 1.40–1.33 (m, 4H), 1.33 (s, 9H), 1.04–1.02 (m, 2H); ^13^C{^1^H} NMR (101 MHz, CDCl_3_), δ (ppm) = 174.6, 171.4, 170.4, 157.2, 137.3, 136.4, 128.9, 128.4, 128.0, 127.9, 127.7, 126.7, 81.2, 66.5, 58.9, 57.5, 52.2, 36.8, 32.8, 32.3, 28.0, 25.1, 21.2, 21.1; ESI-MS: *m*/*z* (%) calcd 552.3 [M]^+^. Found 553.8 [M + H]^+^ (100); anal. calcd for C_31_H_40_N_2_O_7_: C, 67.37; H, 7.30; N, 5.07. Found: C, 67.35; H, 7.31; N, 5.09.

##### 4-(*tert*-Butyl)-1-methyl *N*-Benzyl-*N*-(((*S*)-1-(benzyloxy)-4-methyl-1-oxopentan-2-yl)carbamoyl)-l-aspartate (**4c**)

Yellow oil. Yield 92% (104 mg). *R*_*f*_ (hexane/AcOEt, 70:30) = 0.35; [α]_D_^20^ −15.8 (*c* = 1.0, CHCl_3_); ^1^H NMR (400 MHz, CDCl_3_), δ (ppm) = 7.25–7.23 (m, 10H), 5.04 (d, *J* = 12.4 Hz, 1H), 5.01 (d, *J* = 12.4 Hz, 1H), 4.93–4.91 (m, 2H), 4.47 (d, *J* = 17.2 Hz, 1H), 4.39 (br s, 1H), 4.36 (d, *J* = 17.2 Hz, 1H), 3.59 (s, 3H), 2.97 (dd, *J* = 16.8 and 7.2 Hz, 1H), 2.62 (dd, *J* = 16.8 and 6.8 Hz, 1H), 1.35–1.33 (m, 1H), 1.32–1.30 (m, 2H), 1.32 (s, 9H), 0.75 (d, *J* = 6.4 Hz, 3H), 0.73 (d, *J* = 6.4 Hz, 3H); ^13^C{^1^H} NMR (101 MHz, CDCl_3_), δ (ppm) = 173.6, 171.7, 170.5, 158.1, 137.4, 136.0, 129.2, 128.8, 128.5, 128.4, 128.0, 127.0, 81.5, 67.0, 57.6, 52.8, 52.6, 51.4, 41.7, 37.0, 28.3, 25.0, 23.0, 22.2; ESI-MS: *m*/*z* (%) calcd 540.3 [M]^+^. Found 563.4 [M + Na]^+^ (100); anal. calcd for C_30_H_40_N_2_O_7_: C, 66.65; H, 7.46; N, 5.18. Found: C, 66.66; H, 7.46; N, 5.19.

#### General Procedure for the Synthesis of Hydantoin Intermediates **5**

To a solution of the isocyanate 2 (2.5 mmol, 1 equiv) in DCM (0.3 M solution), a solution of TEA (2 equiv) and *N*-alkyl α-aminoester 3 (1.2 equiv) in DCM (0.3 M solution) was added at rt. The solution was left to stir for 18 h. In case the urea derivative (TLC monitoring) is left, a 1 N NaOH aqueous solution (10% in volume) was added and the mixture was vigorously stirred for 5 min. The reaction was quenched with a 1 N HCl aqueous solution and extracted with DCM. The collected organic phases were washed with a 1 N HCl aqueous solution (once), brine (once), a saturated aqueous solution of NaHCO_3_ (twice), and brine (once). The organic phase was dried over Na_2_SO_4_, filtered, and the solvent evaporated. The hydantoin intermediates **4** were used without any further purification.

##### Benzyl 1-(4-(2-(*tert*-Butoxy)-2-oxoethyl)-3-isopentyl-2,5-dioxoimidazolidin-1-yl)cyclopropane-1-carboxylate (**5a**)

Orange gum. Yield 77% (523 mg). *R*_*f*_ (hexane/AcOEt, 70:30) = 0.46; [α]_D_^20^ −31.2 (*c* = 1.0, CHCl_3_); ^1^H NMR (400 MHz, CDCl_3_), δ (ppm) = 7.31–7.26 (m, 5H), 5.11 (d, 1H, *J* = 12.2 Hz, 1H), 5.07 (d, *J* = 12.2 Hz, 1H), 4.18 (t, *J* = 4.8 Hz, 1H), 3.65–3.55 (m, 1H), 3.08–3.06 (m, 1H,), 2.75 (dd, *J* = 16.8 and 4.3 Hz, 1H), 2.61 (dd, *J* = 16.8 and 5.5 Hz, 1H), 1.78–1.74 (m, 2H), 1.44–1.42 (m, 5H), 1.43 (s, 9H), 0.91 (d, *J* = 2.4 Hz, 3H), 0.89 (d, *J* = 2.4 Hz, 3H); ^13^C{^1^H} NMR (101 MHz, CDCl_3_), δ (ppm) = 172.6, 170.4, 168.5, 155.6, 135.4, 128.5, 128.2, 127.8, 81.9, 67.4, 55.7, 39.7, 36.6, 32.5, 27.9, 25.8, 22.5, 22.2, 16.5, 16.2; ESI-MS: *m*/*z* (%) calcd 458.2 [M]^+^. Found 481.3 [M + Na]^+^ (100); anal. calcd for C_25_H_34_N_2_O_6_: C, 65.48; H, 7.47; N, 6.11. Found: C, 65.50; H, 7.48; N, 6.10.

##### Benzyl 1-(3-Benzyl-4-(2-(*tert*-butoxy)-2-oxoethyl)-2,5-dioxoimidazolidin-1-yl)cyclopropane-1-carboxylate (**5b**)

Dark orange gum. Yield 79% (712 mg). *R*_*f*_ (hexane/AcOEt, 70:30) = 0.32; [α]_D_^20^ −20.9 (*c* = 1.0, CHCl_3_); ^1^H NMR (400 MHz, CDCl_3_), δ (ppm) = 7.35–7.28 (m, 10H), 5.18 (d, *J* = 12.4 Hz, 1H), 5.13 (d, *J* = 12.4 Hz, 1H), 4.88 (d, *J* = 15.6 Hz, 1H), 4.25 (d, *J* = 15.6 Hz, 1H), 4.05 (t, *J* = 4.8 Hz, 1H), 2.65–2.63 (m, 2H), 1.84–1.81 (m, 2H), 1.58–1.56 (m, 2H), 1.39 (s, 9H); ^13^C{^1^H} NMR (101 MHz, CDCl_3_), δ (ppm) = 172.3, 170.4, 168.3, 156.3, 136.7, 135.4, 128.9, 128.6, 128.2, 127.9, 127.8, 81.9, 67.5, 55.6, 45.1, 32.6, 27.9, 16.7, 16.3; ESI-MS: *m*/*z* (%) calcd 478.2 [M]^+^. Found 517.3 [M + K]^+^ (100), 501.3 [M + Na]^+^ (87); anal. calcd for C_27_H_30_N_2_O_6_: C, 67.77; H, 6.32; N, 5.85. Found: C, 67.78; H, 6.30; N, 5.83.

##### Benzyl (*S*)-1-(3-Benzyl-4-(2-(*tert*-butoxy)-2-oxoethyl)-2,5-dioxoimidazolidin-1-yl)cyclohexane-1-carboxylate (**5c**)

Orange gum. Yield 72% (578 mg). *R*_*f*_ (hexane/AcOEt, 70:30) = 0.36; [α]_D_^20^ −21.3 (*c* = 0.8, CHCl_3_); ^1^H NMR (400 MHz, CDCl_3_), δ (ppm) = 7.25–7.14 (m, 10H), 5.11 (s, 2H), 4.76 (d, *J* = 15.4 Hz, 1H), 4.12 (d, 1H, *J* = 15.4 Hz, 1H), 3.91 (t, 1H, *J* = 5.2 Hz, 1H), 2.79–2.73 (m, 1H), 2.67–2.61 (m, 1H), 2.57 (dd, *J* = 16.6, 4.5 Hz, 1H), 2.50 (dd, *J* = 16.6, 4.5 Hz, 1H), 2.12–1.96 (m, 2H), 1.5 −1.40 (m, 6H), 1.31 (s, 9H); ^13^C{^1^H} NMR (101 MHz, CDCl_3_), δ (ppm) = 172.5, 171.6, 168.4, 157.0, 135.9, 135.8, 128.9, 128.5, 128.2, 128.1, 127.9, 127.8, 81.8, 67.2, 65.5, 55.7, 45.2, 35.7, 31.7, 31.5, 28.0, 24.9, 22.6, 22.4; ESI-MS: *m*/*z* (%) calcd 520.3 [M]^+^. Found 521.4 [M + H]^+^; anal. calcd for C_30_H_36_N_2_O_6_: C, 69.21; H, 6.97; N, 5.38. Found: C, 69.21; H, 6.98; N, 5.40.

##### Benzyl (*S*)-2-(3-Ethyl-4-(2-methoxy-2-oxoethyl)-2,5-dioxoimidazolidin-1-yl)-2-methylpropanoate (**5d**)

Yellow gum. Yield 81% (641 mg). *R*_*f*_ (hexane/AcOEt, 70:30) = 0.28; [α]_D_^20^ +11.4 (*c* = 1.1, CHCl_3_); ^1^H NMR (400 MHz, DMSO-*d*_6_), δ (ppm) = 7.36–7.33 (m 5H), 5.12 (d, *J* = 12.8 Hz, 1H), 5.07 (d, *J* = 12.8 Hz, 1H), 4.35 (t, *J* = 4.4 Hz, 1H), 3.56 (s, 3H), 3.44–3.42 (m, 1H), 3.08–3.06 (m, 1H), 2.95 (dd, *J* = 17.2 and 4.4 Hz, 1H), 2.88 (dd, *J* = 17.2 and 4.4 Hz, 1H), 1.66 (s, 3H), 1.62 (s, 3H), 1.02 (t, *J* = 7.2 Hz, 3H); ^13^C{^1^H} NMR (101 MHz, CDCl_3_), δ (ppm) = 172.4, 171.7, 169.6, 155.7, 135.7, 128.5, 128.24, 128.22, 67.3, 60.7, 55.4, 52.2, 36.0, 34.2, 24.0, 23.8, 13.1; ESI-MS: *m*/*z* (%) calcd 376.2 [M]^+^. Found 399.3 [M + Na]^+^ (100); anal. calcd for C_19_H_24_N_2_O_6_: C, 60.63; H, 6.43; N, 7.44. Found: C, 60.61; H, 6.43; N, 7.45.

##### Benzyl (*S*)-2-(4-(2-Methoxy-2-oxoethyl)-3-(naphthalen-1-ylmethyl)-2,5-dioxoimidazolidin-1-yl)-2-methylpropanoate (**5e**)

Orange gum. Yield 82% (589 mg). *R*_*f*_ (hexane/AcOEt, 70:30) = 0.36; [α]_D_^20^ −18.2 (*c* = 1.0, CHCl_3_); ^1^H NMR (400 MHz, CDCl_3_), δ (ppm) = 7.96–7.93 (m, 1H), 7.77–7.72 (m, 2H), 7.41–7.36 (m 2H), 7.25–7.22 (m, 7H), 5.15 (d, *J* = 12.4 Hz, 1H), 5.09 (d, *J* = 12.4 Hz, 1H), 5.07 (d, *J* = 15.6 Hz, 1H), 4.68 (d, *J* = 15.6 Hz, 1H), 3.66 (dd, *J* = 4.8 and 3.6 Hz, 1H), 3.25 (s, 3H), 2.63 (dd, *J* = 16.8 and 3.6 Hz, 1H), 2.47 (dd, *J* = 16.8 and 4.8 Hz, 1H), 1.73 (s, 6H); ^13^C{^1^H} NMR (101 MHz, CDCl_3_), δ (ppm) = 172.5, 171.7, 168.9, 156.3, 135.8, 134.9, 131.3, 130.9, 129.4, 128.8, 128.5, 128.2, 128.1, 127.2, 127.0, 126.3, 125.1, 123.4, 67.3, 60.9, 55.8, 51.9, 43.8, 33.5, 24.0, 23.8; ESI-MS: *m*/*z* (%) calcd 488.2 [M]^+^. Found 511.2 [M + Na]^+^ (100); anal. calcd for C_28_H_28_N_2_O_6_: C, 68.84; H, 5.78; N, 5.73. Found: C, 68.84; H, 5.79; N, 5.72.

##### Benzyl (*S*)-2-(3-Benzyl-4-(2-(*tert*-butoxy)-2-oxoethyl)-2,5-dioxoimidazolidin-1-yl)acetate (**5f**)

Yellowish oil. Yield 74% (612 mg). *R*_*f*_ (hexane/AcOEt, 70:30) = 0.32; [α]_D_^20^ −10.7 (*c* = 1.0, CHCl_3_); ^1^H NMR (400 MHz, CDCl_3_), δ (ppm) = 7.31–7.28 (m, 10H), 5.18 (d, *J* = 12.4 Hz, 1H), 5.14 (d, *J* = 12.4 Hz, 1H), 4.86 (d, *J* = 15.6 Hz, 1H), 4.32 (s, 2H), 4.22 (d, *J* = 15.6 Hz, 1H), 4.16 (t, *J* = 4.8 Hz, 1H), 4.68–4.65 (m, 2H), 1.34 (s, 9H); ^13^C{^1^H} NMR (101 MHz, CDCl_3_), δ (ppm) = 173.2, 169.6, 168.5, 157.5, 137.1, 136.5, 130.5, 130.1, 130.09, 130.07, 129.9, 129.6, 129.4, 83.6, 69.2, 57.8, 46.8, 41.5, 37.0, 29.5; ESI-MS: *m*/*z* (%) calcd 452.2 [M]^+^. Found 475.3 [M + Na]^+^ (100); anal. calcd for C_25_H_28_N_2_O_6_: C, 66.36; H, 6.24; N, 6.19. Found: C, 66.37; H, 6.23; N, 6.20.

##### Benzyl (*S*)-2-(4-(2-(*tert*-Butoxy)-2-oxoethyl)-3-(naphthalen-1-ylmethyl)-2,5-dioxoimidazolidin-1-yl)acetate (**5g**)

Yellowish oil. Yield 88% (582 mg). *R*_*f*_ (hexane/AcOEt, 70:30) = 0.28; [α]_D_^20^ +8.2 (*c* = 1.0, CHCl_3_); ^1^H NMR (400 MHz, CDCl_3_), δ (ppm) = 7.99–7.96 (m, 1H), 7.80–7.74 (m, 2H), 7.44–7.42 (m, 2H), 7.31–7.23 (m, 7H), 5.32 (d, *J* = 15.6 Hz, 1H), 5.12 (s, 2H), 4.65 (d, *J* = 15.6 Hz, 1H), 4.30 (s, 2H), 3.89 (dd, *J* = 5.2 and 4.0 Hz, 1H), 2.68 (dd, *J* = 16.8 and 4.0 Hz, 1H), 2.56 (dd, *J* = 16.8 and 5.2 Hz, 1H), 1.20 (s, 9H); ^13^C{^1^H} NMR (101 MHz, CDCl_3_), δ (ppm) = 171.6, 167.7, 166.9, 155.8, 135.0, 134.0, 131.2, 130.7, 129.2, 128.8, 128.5, 128.3, 127.1, 126.6, 126.3, 125.2, 123.2, 82.0, 67.6, 56.5, 43.7, 40.0, 35.2, 27.8; ESI-MS: *m*/*z* (%) calcd 502.2 [M]^+^. Found 525.3 [M + Na]^+^ (100); anal. calcd for C_29_H_30_N_2_O_6_: C, 69.31; H, 6.02; N, 5.57. Found: C, 69.33; H, 6.02; N, 5.58.

##### Benzyl 2-((*S*)-3-Benzyl-4-(2-(*tert*-butoxy)-2-oxoethyl)-2,5-dioxoimidazolidin-1-yl)-4-methylpentanoate (**5h**)

Yellow gum. Yield 89% (217 mg). *R*_*f*_ (hexane/AcOEt, 70:30) = 0.32; ^1^H NMR (400 MHz, CDCl_3_), δ (ppm), mixture of diastereoisomers = 7.34–7.27 (m, 20H), 5.21–5.17 (m, 4H), 4.90–4.83 (m, 4H), 4.29–4.26 (m, 2H), 4.15–4.09 (m, 2H), 2.68–2.63 (m, 4H), 2.34–2.30 (m, 2H), 1.96–1.93 (m, 2H), 1.66–1.64 (m, 2H), 1.41 (s, 9H), 1.40 (s, 9H), 0.98–0.95 (m, 12H); ^13^C{^1^H} NMR (101 MHz, CDCl_3_), δ (ppm), mixture of diastereoisomers = 172.2, 172.1, 169.76, 169.73, 168.6, 156.7, 156.4, 136.1, 135.67, 135.62, 129.2, 129.1, 128.88, 128.85, 128.6, 128.58, 128.51, 128.3, 128.2, 128.17, 128.12, 128.0, 127.9, 127.3, 82.2, 67.8, 67.7, 56.2, 56.1, 52.4, 52.0, 45.6, 45.5, 37.2, 37.0, 36.0, 35.7, 30.0, 28.2, 25.2, 23.4, 23.3, 21.4, 21.3; ESI-MS: *m*/*z* (%) calcd 508.3 [M]^+^. Found 531.3 [M + Na]^+^ (100); anal. calcd for C_29_H_36_N_2_O_6_: C, 68.48; H, 7.13; N, 5.51. Found: C, 68.46; H, 7.14; N, 5.52.

#### General Procedure for the Synthesis of Hydantoin Monoamides **7**

To a solution of hydantoin derivative **5** (1.2 mmol, 1 equiv) in a 1:1 mixture AcOEt/MeOH (0.1 M solution), a catalytic amount of Pd/C was added at rt. The mixture was vigorously stirred under hydrogen atmosphere. When the starting material is totally consumed (around 3 h, TLC monitoring), the mixture is filtered on a Celite pad, washed with MeOH, and the solvent evaporated. The crude was dissolved in DMF (0.1 M solution), and HBTU (1.1 equiv) followed by TEA (1.1 equiv) and the amine (1.1 equiv) were added at rt. The reaction was left to stir for 12 h. The solution was diluted with AcOEt and washed with brine (three times), a 1 N HCl aqueous solution (twice), brine (once), a saturated NaHCO_3_ aqueous solution (twice), and brine (twice). The organic phase was dried over Na_2_SO_4_, filtered, and the solvent evaporated. Hydantoin monoamides **7** were used without any further purification.

##### Benzyl 1-(3-Benzyl-4-(2-((2,2-diphenylethyl)amino)-2-oxoethyl)-2,5-dioxoimidazolidin-1-yl)cyclopropane-1-carboxylate (**7a**)

Yellow gum. Yield 75% (230 mg). *R*_*f*_ (hexane/AcOEt, 50:50) = 0.36; [α]_D_^20^ −24.2 (*c* = 1.0, CHCl_3_); ^1^H NMR (400 MHz, CDCl_3_), δ (ppm) = 7.32–7.19 (m, 20H), 5.17 (br s, 1H), 5.14 (d, *J* = 10.0 Hz, 1H), 5.06 (d, *J* = 10.0 Hz, 1H), 4.62 (d, *J* = 12.0 Hz, 1H), 4.26 (d, *J* = 12.0 Hz, 1H), 4.16–4.14 (m, 1H), 4.06 (t, *J* = 6.4 Hz, 1H), 3.86–3.84 (m, 1H), 3.69–3.67 (m, 1H), 3.45 (dd, *J* = 12.8 and 3.2 Hz, 1H), 2.14–2.11 (m, 1H), 1.83–1.81 (m, 4H); ^13^C{^1^H} NMR (101 MHz, CDCl_3_), δ (ppm) = 173.0, 170.5, 167.8, 156.3, 141.7, 141.6, 136.4, 135.5, 128.94, 128.92, 128.8, 128.7, 128.6, 128.5, 128.3, 128.2, 128.1, 128.0, 127.9, 127.1, 67.7, 56.0, 50.5, 45.5, 44.0, 36.8, 32.8, 16.8, 16.3; ESI-MS: *m*/*z* (%) calcd 601.3 [M]^+^. Found 624.9 [M + Na]^+^ (100), 602.0 [M + H]^+^ (12); anal. calcd for C_37_H_35_N_3_O_5_: C, 73.86; H, 5.86; N, 6.98. Found: C, 73.86; H, 5.87; N, 7.00

##### *tert*-Butyl 2-(3-Benzyl-1-(1-((2,2-diphenylethyl)carbamoyl)cyclopropyl)-2,5-dioxoimidazolidin-4-yl)acetate (**7b**)

Amorphous white solid. Yield 80% (421 mg). *R*_*f*_ (hexane/AcOEt, 50:50) = 0.31; [α]_D_^20^ −15.4 (*c* = 1.0, CHCl_3_); ^1^H NMR (400 MHz, CDCl_3_), δ (ppm) = 7.19–7.12 (m, 16H), 4.53 (d, *J* = 15.2 Hz, 1H), 4.24 (d, *J* = 15.2 Hz, 1H), 4.24 (s, 1H), 3.92–3.90 (m, 1H), 3.65–3.61 (m, 2H), 2.80 (dd, *J* = 18.0 and 3.2 Hz, 1H), 2.52 (dd, *J* = 4.4 Hz, 1H), 1.54–1.51 (m, 4H), 1.29 (s, 9H); ^13^C{^1^H} NMR (101 MHz, CDCl_3_), δ (ppm) = 172.0, 169.4, 166.3, 156.0, 142.9, 142.6, 136.1, 128.9, 128.5, 128.3, 128.25, 128.23, 128.05, 128.02, 126.2, 126.1, 77.2, 56.1, 49.9, 48.9, 45.9, 45.2, 32.8, 25.4, 16.0, 15.5; ESI-MS: *m*/*z* (%) calcd 567.3 [M]^+^. Found 590.5 [M + Na]^+^ (100); anal. calcd for C_34_H_37_N_3_O_5_: C, 71.94; H, 6.57; N, 7.40. Found: C, 71.95; H, 6.58; N, 7.39.

##### *tert*-Butyl 2-(1-(1-((4-Fluorobenzyl)carbamoyl)cyclopropyl)-3-isopentyl-2,5-dioxoimidazolidin-4-yl)acetate (**7c**)

Amorphous white solid. Yield 86% (256 mg). *R*_*f*_ (hexane/AcOEt, 50:50) = 0.15; [α]_D_^20^ +9.4 (*c* = 1.0, CHCl_3_); ^1^H NMR (400 MHz, CDCl_3_), δ (ppm) = 7.94 (br s, 1H), 7.13–7.11 (m, 2H), 6.89–6.85 (m, 2H), 4.45 (dd, *J* = 15.6 and 6.4 Hz, 1H), 4.27 (dd, *J* = 15.6 and 5.6 Hz, 1H), 3.84 (dd, *J* = 4.4 and 2.4 Hz, 1H), 3.46–3.42 (m, 1H), 3.09–3.05 (m, 1H), 3.02 (dd, *J* = 18.0 and 2.4 Hz, 1H), 2.73 (dd, 18.0 and 4.4 Hz, 1H), 1.83–1.81 (m, 1H), 1.65–1.63 (m, 1H), 1.48 (septet, *J* = 6.4 Hz, 1H), 1.38–1.14 (m, 4H), 1.13 (s, 9H), 0.86 (d, *J* = 6.4 Hz, 3H), 0.84 (d, *J* = 6.4 Hz, 3H); ^13^C{^1^H} NMR (101 MHz, CDCl_3_), δ (ppm) = 172.1, 169.7, 169.2, 161.8 (d, *J* = 244.2 Hz), 155.5, 134.5 (d, *J* = 3.0 Hz), 128.6 (d, *J* = 8.1 Hz), 115.1 (d, *J* = 22.2 Hz), 83.2, 55.6, 42.8, 39.6, 37.0, 33.6, 33.5, 27.7, 25.9, 22.4, 22.3, 16.3, 15.9; ESI-MS: *m*/*z* (%) calcd 475.2 [M]^+^. Found 498.3 [M + Na]^+^ (100); anal. calcd for C_25_H_34_FN_3_O_5_: C, 63.14; H, 7.21; N, 8.84. Found: C, 63.16; H, 7.21; N, 8.85.

##### *tert*-Butyl 2-(3-Benzyl-1-(1-((2,2-diphenylethyl)carbamoyl)cyclohexyl)-2,5-dioxoimidazolidin-4-yl)acetate (**7d**)

Yellow gum. Yield 81% (518 mg). *R*_*f*_ (hexane/AcOEt, 50:50) = 0.41; [α]_D_^20^ +13.8 (*c* = 1.0, CHCl_3_); ^1^H NMR (400 MHz, CDCl_3_), δ (ppm) = 7.35–7.24 (m, 15H), 7.00 (t, *J* = 5.6 Hz, 1H), 4.75 (d, *J* = 15.2 Hz, 1H), 4.38 (t, *J* = 8.0 Hz, 1H), 4.20 (d, *J* = 15.2 Hz, 1H), 4.10–4.07(m, 1H), 8.81–3.73 (m, 2H), 2.85 (dd, *J* = 17.6 and 3.6 Hz, 1H), 2.69–2.62 (m 1H), 2.09–2.07 (m, 1H), 1.78–1.28 (m, 9H), 1.45 (s, 9H); ^13^C{^1^H} NMR (101 MHz, CDCl_3_), δ (ppm) = 172.5, 172.3, 169.2, 156.7, 142.3, 142.2, 135.8, 129.1, 128.5, 128.4, 128.2, 128.12, 128.10, 127.9, 126.5, 126.4, 82.5, 70.0, 55.3, 50.4, 45.3, 44.3, 34.0, 32.1, 28.1, 25.1, 22.8, 22.2; ESI-MS: *m*/*z* (%) calcd 609.3 [M]^+^. Found 632.4 [M + Na]^+^ (100); anal. calcd for C_37_H_43_N_3_O_5_: C, 72.88; H, 7.11; N, 6.89. Found: C, 72.89; H, 7.13; N, 6.90.

##### Methyl (*S*)-2-(3-Ethyl-1-(1-(hexylamino)-2-methyl-1-oxopropan-2-yl)-2,5-dioxoimidazolidin-4-yl)acetate (**7e**)

Amorphous white solid. Yield 79% (267 mg). *R*_*f*_ (hexane/AcOEt, 50:50) = 0.35; [α]_D_^20^ +22.3 (*c* = 1.0, CHCl_3_); ^1^H NMR (400 MHz, CDCl_3_), δ (ppm) = 6.70 (br s, 1H), 3.99 (dd, *J* = 4.4 and 3.2 Hz, 1H), 3.70 (s, 3H), 3.56 (sextet, *J* = 7.2 Hz, 1H), 3.24–3.19 (m, 3H), 3.08 (dd, *J* = 17.6 and 3,2 Hz, 1H), 2.91 (dd, *J* = 17.6 and 4.4 Hz, 1H), 1.80 (s, 3H), 1.73 (s, 3H), 1.49–1.47 (m, 2H), 1.27–1.23 (m, 6H), 1.35 (t, *J* = 7.2 Hz, 3H), 0.86 (t, *J* = 6.8 Hz, 3H); ^13^C{^1^H} NMR (101 MHz, CDCl_3_), δ (ppm) = 172.9, 171.9, 170.2, 155.8, 62.3, 55.2, 52.3, 39.9, 35.9, 32.9, 31.5, 29.1, 26.5, 25.2, 24.6, 22.5, 14.0, 13.2; ESI-MS: *m*/*z* (%) calcd 369.2 [M]^+^. Found 392.4 [M + Na]^+^ (100); anal. calcd for C_18_H_31_N_3_O_5_: C, 58.52; H, 8.46; N, 11.37. Found: C, 58.51; H, 8.48; N, 11.38.

##### Methyl (*S*)-2-(1-(1-(Isobutylamino)-2-methyl-1-oxopropan-2-yl)-3-(naphthalen-1-ylmethyl)-2,5-dioxoimidazolidin-4-yl)acetate (**7f**)

Yellow gum. Yield 91% (208 mg). *R*_*f*_ (hexane/AcOEt, 50:50) = 0.37; [α]_D_^20^ −10.0 (*c* = 1.0, CHCl_3_); ^1^H NMR (400 MHz, CDCl_3_), δ (ppm) = 8.02–8.00 (m, 1H), 7.80–7.78 (m, 2H), 7.45–7.43 (m, 2H), 7.34–7.32 (m, 2H), 6.92 (br t, *J* = 4.8 Hz, 1H), 4.97 (d, *J* = 14.8 Hz, 1H), 4.85 (d, *J* = 14.8 Hz, 1H), 3.61 (dd, *J* = 4.4 and 2.8 Hz, 1H), 3.14 (s, 3H), 3.10–3.08 (m, 1H), 3.02–3.00 (m, 1H), 2.71 (dd, *J* = 18.0 Hz and 2.8 Hz, 1H), 2.38 (dd, *J* = 18.0 and 4.4 Hz, 1H), 1.76 (s, 3H), 1.75–1.73 (m, 1H), 1.71 (s, 3H), 0.85 (d, *J* = 4.0 Hz, 3H), 0.83 (d, *J* = 4.0 Hz. 3H); ^13^C{^1^H} NMR (101 MHz, CDCl_3_), δ (ppm) = 173.5, 172.2, 170.4, 156.7, 134.4, 131.7, 131.6, 130.0, 129.1, 128.1, 127.2, 126.6, 125.4, 124.0, 62.9, 56.4, 52.2, 47.8, 45.1, 33.3, 28.7, 25.8, 24.9, 20.6; ESI-MS: *m*/*z* (%) calcd 453.2 [M]^+^. Found 476.2 [M + Na]^+^ (100); anal. calcd for C_25_H_31_N_3_O_5_: C, 66.21; H, 6.89; N, 9.27. Found: C, 66.19; H, 6.88; N, 9.28.

##### Methyl (*S*)-2-(3-Benzyl-1-(2-((2,2-diphenylethyl)amino)-2-oxoethyl)-2,5-dioxoimidazolidin-4-yl)acetate (**7g**)

Orange gum. Yield 82% (174 mg). *R*_*f*_ (hexane/AcOEt, 50:50) = 0.43; [α]_D_^20^ −6.0 (*c* = 1.1, CHCl_3_); ^1^H NMR (400 MHz, CDCl_3_), δ (ppm) = 7.17–7.02 (m, 15H), 7.00 (br s, 1H), 4.49 (d, *J* = 15.2 Hz, 1H), 4.31 (d, *J* = 15.2 Hz, 1H), 4.17 (t, *J* = 8.0 Hz, 1H), 4.12 (s, 2H), 3.86–3.77 (m, 3H), 3.39 (s, 3H), 2.75 (dd, *J* = 17.6 and 3.6 Hz, 1H), 2.58 (dd, *J* = 17.6 and 4.4 Hz, 1H); ^13^C{^1^H} NMR (101 MHz, CDCl_3_), δ (ppm) = 171.2, 169.9, 166.8, 155.7, 142.1, 142.0, 135.5, 129.0, 128.5, 128.4, 128.3, 128.23, 128.20, 128.1, 126.6, 56.1, 52.3, 50.1, 45.6, 44.6, 41.8, 32.8, 29.7; ESI-MS: *m*/*z* (%) calcd 499.2 [M]^+^. Found 522.2 [M + Na]^+^ (100); anal. calcd for C_29_H_29_N_3_O_5_: C, 69.72; H, 5.85; N, 8.41. Found: C, 69.74; H, 5.85; N, 8.40.

##### *tert*-Butyl (*S*)-2-(1-(2-(Isobutylamino)-2-oxoethyl)-3-(naphthalen-1-ylmethyl)-2,5-dioxoimidazolidin-4-yl)acetate (**7h**)

Yellow gum. Yield 93% (278 mg). *R*_*f*_ (hexane/AcOEt, 50:50) = 0.24; [α]_D_^20^ −23.1 (*c* = 1.0, CHCl_3_); ^1^H NMR (400 MHz, CDCl_3_), δ (ppm) = 8.06–8.04 (m, 1H), 7.83–7.80 (m, 2H), 7.49–7.35 (m, 4H), 7.23 (br t, *J* = 5.6 Hz, 1H), 5.18 (d, *J* = 14.8 Hz, 1H), 4.75 (d, *J* = 14.8 Hz, 1H), 4.23 (d, *J* = 16.8 Hz, 1H), 4.18 (d, *J* = 16.8 Hz, 1H), 3.67 (dd, *J* = 4.4 and 2.8 Hz, 1H), 3.10–3.07 (m, 1H), 2.93–2.90 (m, 1H), 2.83 (dd, *J* = 17.6 and 2.8 Hz, 1H), 2.60 (dd, *J* = 17.6 and 4.4 Hz, 1H), 1.77 (septet, *J* = 6.8 Hz, 1H), 1.19 (s, 9H), 0.82 (d, *J* = 6.8 Hz, 6H); ^13^C{^1^H} NMR (101 MHz, CDCl_3_), δ (ppm) = 171.5, 169.1, 166.4, 155.8, 134.1, 131.3, 130.7, 129.7, 128.9, 127.6, 127.3, 126.5, 125.1, 123.3, 82.9, 56.4, 47.2, 44.4, 42.1, 33.9, 28.4, 27.7, 20.2, 20.1; ESI-MS: *m*/*z* (%) calcd 467.2 [M]^+^. Found 490.1 [M + Na]^+^ (100); anal. calcd for C_26_H_33_N_3_O_5_: C, 66.79; H, 7.11; N, 8.99. Found: C, 66.80; H, 7.11; N, 9.00.

##### *tert*-Butyl (*S*)-2-(1-(2-((4-Chlorobenzyl)amino)-2-oxoethyl)-3-(naphthalen-1-ylmethyl)-2,5-dioxoimidazolidin-4-yl)acetate (**7i**)

Amorphous white solid. Yield 90% (185 mg). *R*_*f*_ (hexane/AcOEt, 50:50) = 0.28; [α]_D_^20^ −12.9 (*c* = 1.0, CHCl_3_); ^1^H NMR (400 MHz, CDCl_3_), δ (ppm) = 8.11–8.09 (m, 1H), 7.90–7.88 (m, 2H), 7.78 (br t, *J* = 6.0 Hz, 1H), 7.57–7.54 (m, 2H), 7.46–7.43 (m, 2H), 7.27 (d, *J* = 8.4 Hz, 2H), 7.20 (d, *J* = 8.4 Hz, 2H), 5.29 (d, *J* = 14.8 Hz, 1H), 4.78 (d, *J* = 14.8 Hz, 1H), 4.56 (dd, *J* = 15.6 and 6.4 Hz, 1H), 4.38 (dd, *J* = 15.6 and 5.6 Hz, 1H), 4.37–4.36 (m, 2H), 3.74 (dd, *J* = 4.4 and 2.4 Hz, 1H), 2.89 (dd, *J* = 18.0 and 2.4 Hz, 1H), 2.67 (dd, *J* = 18.0 and 4.4 Hz, 1H), 1.10 (s, 9H); ^13^C{^1^H} NMR (101 MHz, CDCl_3_), δ (ppm) = 171.5, 169.1, 166.6, 136.7, 134.1, 132.8, 131.3, 130.6, 129.7, 128.9, 128.65, 128.61, 127.6, 127.3, 126.5, 125.1, 123.2, 83.1, 56.3, 44.2, 42.5, 42.1, 33.9, 27.6; ESI-MS: *m*/*z* (%) calcd 535.2 [M]^+^. Found 558.4 [M + Na]^+^ (68); anal. calcd for C_29_H_30_ClN_3_O_5_: C, 64.98; H, 5.64; N, 7.84. Found: C, 64.96; H, 5.65; N, 7.85.

#### General Procedure for the Hydrolysis of the *tert*-Butyl Ester of Hydantoin Monoamides **7**

To a solution of hydantoin monoamide 7 (1.0 mmol, 1 equiv) in DCM (0.1 M solution), TFA (30% in volume) was added at rt. The mixture was stirred until the starting material is totally consumed (around 12 h, TLC monitoring). The solvents were evaporated and co-evaporated twice with cyclohexane. The obtained acids were used in the coupling reaction without any further purification.

#### General Procedure for the Hydrolysis of the Methyl Ester of Hydantoin Monoamides **7**

To a solution of hydantoin monoamide **7** (1.0 mmol, 1 equiv) in MeOH (0.1 M solution), a 1 M NaOH aqueous solution (10% in volume) was added at rt. The reaction was stirred until complete disappearance of the starting material (around 3 h, TLC monitoring). The organic solvent was evaporated, the mixture was diluted with AcOEt, and a 1 M HCl aqueous solution was added until acidic pH was reached. The phases were separated, and the aqueous phase was washed three times with AcOEt. The collected organic phases were dried over Na_2_SO_4_, filtered, and the solvent evaporated. The obtained acids were used in the coupling reaction without any further purification.

#### General Procedure for the Synthesis of Hydantoin Universal Peptidomimetics **8**

The acid obtained was dissolved in DMF (1.0 mmol, 0.1 M solution), and HBTU (1.1 equiv) followed by TEA (1.1 equiv) and the amine (1.1 equiv) were added at rt. The reaction was left to stir for 12 h. The solution was diluted with AcOEt and washed with brine (three times), a 1 N HCl aqueous solution (twice), brine (once), a saturated NaHCO_3_ aqueous solution (twice), and brine (twice). The organic phase was dried over Na_2_SO_4_, filtered, and the solvent evaporated. Hydantoin universal peptidomimetics **8** were purified by flash chromatography (from AcOEt/hexane = 80:20 to AcOEt 100%).

##### (*S*)-1-(3-Benzyl-4-(2-((2,2-diphenylethyl)amino)-2-oxoethyl)-2,5-dioxoimidazolidin-1-yl)-*N*-hexylcyclopropane-1-carboxamide (**8a**)

Yellowish gum. Yield 82% (134 mg). *R*_*f*_ (AcOEt/hexane, 80/20) = 0.29; [α]_D_^20^ −31.2 (*c* = 1.0, CHCl_3_); ^1^H NMR (400 MHz, CDCl_3_), δ (ppm) = 8.03 (br s, 1H), 7.24–7.05 (m, 15H), 5.34 (br s, 1H), 4.57 (d, *J* = 14.8 Hz, 1H), 4.04–4.02 (m, 2H), 3.69–3.63 (m, 3H), 3.24 (m, 1H), 3.14 (m, 1H), 2.82 (br s, 1H), 2.30 (br s, 1H), 1.85–1.82 (m, 1H), 1.63–1.60 (m, 2H), 1.50–1.46 (m, 2H), 1.16–1.10 (m, 7H), 0.73 (t, *J* = 6.8 Hz, 3H); ^13^C{^1^H} NMR (400 MHz, CDCl_3_), δ (ppm) = 172.1, 169.3, 167.6, 156.1, 141.3, 135.8, 129.0, 128.9, 128.2, 128.1, 127.9, 127.8, 127.15, 127.11, 55.5, 50.3, 45.4, 43.9, 40.6, 34.1, 33.7, 31.5, 29.1, 26.6, 22.6, 16.1, 15.7, 14.0; ESI-MS: *m*/*z* (%) calcd 594.3 [M]^+^. Found 617.4 [M + Na]^+^ (100), 595.4 [M + H]^+^ (5); anal. calcd for C_36_H_42_N_4_O_4_: C, 72.70; H, 7.12; N, 9.42. Found: C, 72.69; H, 7.12; N, 9.40

##### (*S*)-1-(4-(2-((2-(1*H*-Indol-3-yl)ethyl)amino)-2-oxoethyl)-3-benzyl-2,5-dioxoimidazolidin-1-yl)-*N*-(2,2-diphenylethyl)cyclopropane-1-carboxamide (**8b**)

Amorphous white solid. Yield 82% (101 mg). *R*_*f*_ (AcOEt) = 0.35; [α]_D_^20^ −10.1 (*c* = 1.0, CHCl_3_); ^1^H NMR (400 MHz, DMSO-*d*_6_), δ (ppm) = 10.88 (s, 1H), 8.52 (brs, 1H), 8.25 (s, 1H), 7.56 (d, *J* = 7.8 Hz, 1H), 7.45–7.05 (m, 18H), 7.00 (t, *J* = 7.2 Hz, 1H), 4.57 (d, *J* = 15.9 Hz, 1H), 4.33 (t, *J* = 8.05 Hz, 1H), 4.30 (d, *J* = 15.9 Hz, 1H), 4.02 (t, *J* = 3.4 Hz, 1H), 3.78– 3.75 (m, 1H), 3.63–3.61 (m, 1H), 3.35–3.27 (m, 3H), 2.82–2.80 (m, 2H), 2.73–2.71 (m, 1H), 1.47–1.45 (m, 1H), 1.37–1.35 (m, 1H), 1.16–1.13 (m, 2H); ^13^C{^1^H} NMR (101 MHz, CDCl_3_), δ (ppm) = 171.4, 168.8, 165.7, 155.4, 142.2, 142.0, 135.5, 128.3, 127.9, 127.7, 127.64, 127.62, 127.43, 127.41, 125.6, 125.5, 76.5, 55.4, 49.3, 48.2, 45.3, 44.6, 32.1, 24.8, 15.4, 14.8; ESI-MS: *m*/*z* (%) calcd 653.3 [M]^+^. Found 676.5 [M + Na]^+^ (100); anal. calcd for C_40_H_39_N_5_O_4_: C, 73.49; H, 6.01; N, 10.71. Found: C, 73.50; H, 6.00; N, 10.72.

##### (*S*)-1-(3-Benzyl-4-(2-((4-hydroxyphenethyl)amino)-2-oxoethyl)-2,5-dioxoimidazolidin-1-yl)-*N*-(2,2-diphenylethyl)cyclopropane-1-carboxamide (**8c**)

Amorphous white solid. Yield 85% (84 mg). *R*_*f*_ (AcOEt) = 0.27; [α]_D_^20^ +29.5 (*c* = 1.1, CHCl_3_); ^1^H NMR (400 MHz, DMSO-*d*_6_), δ (ppm) = 9.17 (s, 1H), 8.47 (s, 1H), 8.16 (s, 1H), 7.24–7.14 (m, 15H,), 6.97 (d, *J* = 8.4 Hz, 2H), 6.66 (d, *J* = 8.4 Hz, 2H), 4.51 (d, *J* = 15.9 Hz, 1H), 4.31 (t, *J* = 7.0 Hz, 1H), 4.24 (d, *J* = 15.9 Hz, 1H), 3.96 (s, 1H), 3.80–3.78 (m, 1H), 3.65–3.63 (m, 1H), 3.26–3.24 (m, 2H), 3.17–3.13 (m, 2H), 2.70 (br s, 2H), 1.48–1.46 (m, 1H), 1.31–1.29 (m, 1H), 1.15–1.13 (m, 2H); ^13^C{^1^H} NMR (101 MHz, DMSO-*d*_6_), δ (ppm) = 173.0, 169.8, 168.7, 156.2, 143.3, 143.2, 137.2, 129.8, 129.6, 128.9, 128.7, 128.4, 128.0, 127.8, 126.6, 115.7, 56.3, 49.8, 44.9, 44.8, 41.2, 34.4, 33.8, 33.4, 15.8, 15.2; ESI-MS: *m*/*z* (%) calcd 630.3 [M]^+^. Found 631.5 [M + H]^+^ (100); anal. calcd for C_38_H_38_N_4_O_5_: C, 72.36; H, 6.07; N, 8.88. Found: C, 72.36; H, 6.08; N, 8.90.

##### (*S*)-1-(3-Benzyl-4-(2-(cyclohexylamino)-2-oxoethyl)-2,5-dioxoimidazolidin-1-yl)-*N*-(2,2-diphenylethyl)cyclopropane-1-carboxamide (**8d**)

Amorphous white solid. Yield 92% (163 mg). *R*_*f*_ (AcOEt/hexane, 80:20) = 0.22; [α]_D_^20^ −37.5 (*c* = 1.0, CHCl_3_); ^1^H NMR (400 MHz, CDCl_3_), δ (ppm) = 8.12 (br s, 1H), 7.26–7.20 (m, 15H), 5.03 (d, *J* = 7.6 Hz, 1H), 4.48 (d, *J* = 15.6 Hz, 1H), 4.42 (d, *J* = 15.6 Hz, 1H), 4.37 (t, *J* = 7.6 Hz, 1H), 3.98–3.94 (m, 1H), 3.77–3.70 (m, 2H), 3.54–3.48 (m, 1H), 2.85 (dd, *J* = 16.8 and 2.4 Hz, 1H), 2.33 (dd, *J* = 16.8 and 4.4 Hz, 1H), 1.87–1.56 (m, 10H), 1.19–1.02 (m, 4H); ^13^C{^1^H} NMR (101 MHz, CDCl_3_), δ (ppm) = 172.0, 169.5, 166.4, 156.0, 142.9, 142.6, 136.1, 129.0, 128.5, 128.3, 128.26, 128.24, 128.0, 126.2, 126.1, 56.1, 49.9, 48.9, 45.9, 45.2, 34.1, 33.7, 32.8, 25.4, 24.8, 24.7, 16.0, 15.5; ESI-MS: *m*/*z* (%) calcd 592.3 [M]^+^. Found 615.5 [M + Na]^+^ (100); anal. calcd for C_36_H_40_N_4_O_4_: C, 72.95; H, 6.80; N, 9.45. Found: C, 72.97; H, 6.81; N, 9.44.

##### *tert*-Butyl (*S*)-(6-(2-(3-Benzyl-1-(1-((2,2-diphenylethyl)carbamoyl)cyclopropyl)-2,5-dioxoimidazolidin-4-yl)acetamido)hexyl)carbamate (**8e**)

Amorphous white solid. Yield 78% (78 mg). *R*_*f*_ (AcOEt/hexane, 90:10) = 0.27; [α]_D_^20^ −7.9 (*c* = 1.0, MeOH); ^1^H NMR (400 MHz, CD_3_OD), δ (ppm) = 8.67 (br s, 0.5 H), 7.16–7.10 (m, 15H), 4.40 (d, *J* = 15.6 Hz, 1H), 4.35 (d, *J* = 15.6 Hz, 1H), 4.30 (t, *J* = 7.6 Hz, 1H), 3.85–3.80 (m, 2H), 3.64–3.62 (m, 1H), 3.00–2.80 (m, 4H), 2.74 (dd, *J* = 17.2 and 2.8 Hz, 1H), 2.55 (dd, *J* = 17.2 and 4.8 Hz, 1H), 1.57–1.55 (m, 1H), 1.38–1.30 (m, 3H), 1.32 (s, 9H), 1.19–1.15 (m, 8H). ^13^C{^1^H} NMR (101 MHz, CD_3_OD), δ (ppm) = 173.3, 170.9, 170.8, 168.3, 156.6, 142.7, 142.5, 136.5, 128.4, 128.0, 127.99, 127.92, 127.7, 127.5, 126.0, 78.4, 60.1, 56.6, 49.7, 45.1, 44.9, 39.8, 39.2, 33.2, 29.6, 28.9, 27.4, 26.4, 26.2, 15.4, 14.8; ESI-MS: *m*/*z* (%) calcd 709.4 [M]^+^. Found 710.5 [M + H]^+^ (100); anal. calcd for C_41_H_51_N_5_O_6_: C, 69.37; H, 7.24; N, 9.87. Found: C, 69.38; H, 7.22; N, 9.90.

##### (*S*)-*N*-(4-Fluorobenzyl)-1-(4-(2-(hexylamino)-2-oxoethyl)-3-isopentyl-2,5-dioxoimidazolidin-1-yl)cyclopropane-1-carboxamide (**8f**)

Amorphous white solid. Yield 85% (152 mg). *R*_*f*_ (AcOEt/hexane, 80:20) = 0.22; [α]_D_^20^ −17.3 (*c* = 1.0, CHCl_3_); ^1^H NMR (400 MHz, CD_3_OD), δ (ppm) = 7.23–7.18 (m, 2H), 6.93–6.88 (m, 2H), 4.40 (d, *J* = 15.2 Hz, 1H), 4.25 (d, *J* = 15.2 Hz, 1H), 4.06 (dd, *J* = 4.8 and 2.8 Hz, 1H), 3.46–3.44 (m, 1H), 3.11–3.08 (m, 1H), 2.90–2.87 (m, 2H), 2.83–2.78 (m, 2H), 1.69–1.66 (m, 1H), 1.50–1.11 (m, 14H), 0.88–0.86 (m, 6H), 0.81 (t, *J* = 7.2 Hz, 3H); ^13^C{^1^H} NMR (101 MHz, CD_3_OD), δ (ppm) = 173.7, 171.0, 168.5, 161.9 (d, *J* = 244.4 Hz), 156.3, 134.6 (d, *J* = 4.0 Hz), 128.6 (d, *J* = 8.1 Hz), 114.5 (d, *J* = 21.2 Hz), 56.2, 42.3, 39.3, 38.5, 36.6, 33.3, 33.1, 31.4, 28.9, 26.3, 25.6, 22.3, 22.2, 21.4, 15.4, 15.0, 12.9; ESI-MS: *m*/*z* (%) calcd 502.3 [M]^+^. Found 525.3 [M + Na]^+^ (100); anal. calcd for C_27_H_39_FN_4_O_4_: C, 64.52; H, 7.82; N, 11.15. Found: C, 64.52; H, 7.81; N, 11.15.

##### (*S*)-1-(4-(2-((4-Chlorobenzyl)amino)-2-oxoethyl)-3-isopentyl-2,5-dioxoimidazolidin-1-yl)-*N*-(4-fluorobenzyl)cyclopropane-1-carboxamide (**8g**)

Yellow gum. Yield 85% (117 mg). *R*_*f*_ (AcOEt/hexane, 80:20) = 0.28; [α]_D_^20^ −22.4 (*c* = 1.0, CHCl_3_); ^1^H NMR (400 MHz, CD_3_OD), δ (ppm) = 7.13–7.08 (m, 4H), 6.97–6.95 (m, 2H), 8.82–6.80 (m, 2H), 4.33 (d, *J* = 15.2 Hz, 1H), 4.16 (d, *J* = 15.2 Hz, 1H), 4.08–4.05 (m, 2H), 4.91 (d, *J* = 14.8 Hz, 1H), 3.35–3.32 (m, 1H), 3.01–2.99 (m, 1H), 2.95–2.92 (m, 1H), 2.84–2.80 (m, 1H), 1.64–1.62 (m, 1H), 1.45–1.18 (m, 6H), 0.79 (d, *J* = 4.8 Hz, 3H), 0.77 (d, *J* = 4.8 Hz, 3H); ^13^C{^1^H} NMR (101 MHz, CD_3_OD), δ (ppm) = 172.2, 169.5, 167.2, 160.5 (d, *J* = 244.4 Hz), 154.8, 135.5, 132.9, 131.5, 127.4 (d, *J* = 8.1 Hz), 127.3, 127.0, 113.3 (d, *J* = 22.2 Hz), 54.7, 41.2, 40.8, 38.1, 35.2, 31.9, 24.3, 20.3, 20.2, 14.4, 14.0; ESI-MS: *m*/*z* (%) calcd 542.2 [M]^+^. Found 565.3 [M + Na]^+^ (100); anal. calcd for C_28_H_32_ClFN_4_O_4_: C, 61.93; H, 5.94; N, 10.32. Found: C, 61.94; H, 5.96; N, 10.32.

##### 1-(3-Benzyl-4-(2-(cyclohexylamino)-2-oxoethyl)-2,5-dioxoimidazolidin-1-yl)-*N*-(2,2-diphenylethyl)cyclohexane-1-carboxamide (**8h**)

Amorphous white solid. Yield 91% (201 mg). *R*_*f*_ (AcOEt/hexane, 80:20) = 0.21; [α]_D_^20^ −6.7 (*c* = 1.0, CHCl_3_); ^1^H NMR (400 MHz, CDCl_3_), δ (ppm) = 7.76 (t, *J* = 5.2 Hz, 1H), 7.18–7.14 (m, 15H), 5.78 (d, *J* = 7.6 Hz, 1H), 4.63 (d, *J* = 15.2 Hz, 1H), 4.33 (t, *J* = 8.0 Hz, 1H), 4.10 (d, *J* = 15.2 Hz, 1H), 3.99–3.95 (m, 1H), 3.71–3.68 (m, 1H), 3.60–3.58 (m, 2H), 2.76 (dd, *J* = 16.8 and 2.8 Hz, 1H), 2.60–2.57 (m, 2H), 2.48 (dd, *J* = 16.8 and 4.0 Hz, 1H), 1.99–1.02 (m, 18H); ^13^C{^1^H} NMR (101 MHz, CDCl_3_), δ (ppm) = 172.9, 172.8, 156.9, 142.6, 142.5, 136.1, 128.9, 128.4, 128.34, 128.32, 128.2, 128.0, 127.9, 126.4, 126.3, 67.0, 55.5, 50.2, 48.9, 45.1, 44.5, 34.2, 32.8, 32.7, 32.3, 25.4, 25.1, 24.9, 23.0, 22.1; ESI-MS: *m*/*z* (%) calcd 634.4 [M]^+^. Found 657.5 [M + Na]^+^ (100); anal. calcd for C_39_H_46_N_4_O_4_: C, 73.79; H, 7.30; N, 8.83. Found: C, 73.81; H, 7.31; N, 8.82.

##### 1-(4-(2-((2-(1*H*-Indol-3-yl)ethyl)amino)-2-oxoethyl)-3-benzyl-2,5-dioxoimidazolidin-1-yl)-*N*-(2,2-diphenylethyl)cyclohexane-1-carboxamide (**8i**)

Amorphous white solid. Yield 72% (72 mg). *R*_*f*_ (AcOEt) = 0.23; [α]_D_^20^ +16.5 (*c* = 1.0, CHCl_3_); ^1^H NMR (400 MHz, CDCl_3_), δ (ppm) = 8.16 (s, 1H), 7.57 (br s, 1H), 7.48 (d, *J* = 7.6 Hz, 1H), 7.26 (d, *J* = 7.6 Hz, 1H), 7.20–7.00 (m, 17H), 6.88 (s, 1H), 5.41 (br s, 1H), 4.51 (d, *J* = 15.2 Hz, 1H), 4.34 (t, *J* = 7.8 Hz, 1H), 3.97–3.95 (m, 1H), 3.95 (d, *J* = 15.2 Hz, 1H), 3.67–3.65 (m, 2H), 2.88–2.86 (m, 2H), 2.64 (d, *J* = 15.4 Hz, 1H), 2.61–2.59 (m, 2H), 2.26 (d, *J* = 15.4 Hz, 1H), 2.04–1.56 (m, 10H); ^13^C{^1^H} NMR (101 MHz, CDCl_3_), δ (ppm) = 172.9, 172.7, 167.7, 156.8, 142.6, 142.5, 136.5, 136.0, 128.9, 128.4, 128.36, 128.33, 128.2, 128.0, 127.9, 126.3, 122.4, 112.3, 111.5, 100.0, 67.1, 55.4, 50.2, 45.1, 44.5, 39.8, 34.4, 32.3, 31.3, 29.7, 25.07, 25.00, 23.0, 22.2; ESI-MS: *m*/*z* (%) calcd 695.3 [M]^+^. Found 718.4 [M + Na]^+^ (100); anal. calcd for C_43_H_45_N_5_O_4_: C, 74.22; H, 6.52; N, 10.06. Found: C, 74.23; H, 6.51; N, 10.04.

##### (*S*)-1-(3-Benzyl-4-(2-(isobutylamino)-2-oxoethyl)-2,5-dioxoimidazolidin-1-yl)-*N*-(2,2-diphenylethyl)cyclohexane-1-carboxamide (**8j**)

Amorphous white solid. Yield 83% (111 mg). *R*_*f*_ (AcOEt/hexane, 80:20) = 0.28; [α]_D_^20^ +32.6 (*c* = 1.0, CHCl_3_); ^1^H NMR (400 MHz, CDCl_3_), δ (ppm) = 7.71 (t, *J* = 5.2 Hz, 1H), 7.19–7.14 (m, 15H), 5.89 (br s, 1H), 4.61 (d, *J* = 15.2 Hz, 1H), 4.33 (t, *J* = 8.0 Hz, 1H), 4.13 (d, *J* = 15.2 Hz, 1H), 3.98–3.95 (m, 1H), 3.71 (s, 1H), 3.68–3.66 (m, 1H), 2.97–2.95 (m, 1H), 2.85–2.83 (m, 2H), 2.56–2.54 (m, 2H), 1.99–1.97 (m, 1H), 1.67–1.64 (m, 2H), 1.43–1.40 (m, 7H), 0.80 (d, *J* = 6.4 Hz, 6H); ^13^C{^1^H} NMR (101 MHz, CDCl_3_), δ (ppm) = 173.0, 172.7, 168.0, 156.8, 142.6, 142.5, 136.2, 128.9, 128.4, 128.3, 128.2, 128.0, 126.4, 126.3, 67.0, 55.6, 50.2, 47.3, 45.1, 44.5, 34.3, 32.3, 31.3, 28.3, 25.1, 23.0, 22.1, 20.24, 20.21; ESI-MS: *m*/*z* (%) calcd 608.3 [M]^+^. Found 609.3 [M + H]^+^ (100); anal. calcd for C_37_H_44_N_4_O_4_: C, 73.00; H, 7.29; N, 9.20. Found: C, 73.01; H, 7.28; N, 9.20.

##### (*S*)-2-(3-Ethyl-4-(2-((4-hydroxyphenethyl)amino)-2-oxoethyl)-2,5-dioxoimidazolidin-1-yl)-*N*-hexyl-2-methylpropanamide (**8k**)

Amorphous white solid. Yield 93% (228 mg). *R*_*f*_ (AcOEt/hexane, 80:20) = 0.12; [α]_D_^20^ −14.6 (*c* = 1.0, CHCl_3_); ^1^H NMR (400 MHz, CD_3_OD), δ (ppm) = 8.00 (br s, 1H), 7.00 (d, *J* = 6.8 Hz, 2H), 6.70 (d, *J* = 6.8 Hz, 2H), 4.11 (t, *J* = 3.2 Hz, 1H), 3.49–3.47 (m, 1H), 3.36–3.33 (m, 2H), 3.19–3.14 (m, 2H), 3.10–3.06 (m, 1H), 2.87 (dd, *J* = 13.2 and 2.4 Hz, 1H), 2.82 (dd, *J* = 13.2 and 3.6 Hz, 1H), 2.68 (t, *J* = 6.0 Hz, 2H), 1.77 (s, 3H), 1.67 (s, 3H), 1.51–1.49 (m, 2H), 1.27–1.15 (m, 6H), 1.11 (t, *J* = 5.6 Hz, 3H), 0.86 (t, *J* = 4.8 Hz, 3H); ^13^C{^1^H} NMR (101 MHz, CD_3_OD), δ (ppm) = 173.6, 172.2, 167.9, 155.3, 154.5, 128.5, 128.1, 113.8, 60.6, 54.8, 39.6, 38.6, 34.4, 33.0, 32.8, 30.2, 27.6, 25.2, 23.3, 22.5, 21.1, 11.0; ESI-MS: *m*/*z* (%) calcd 474.3 [M]^+^. Found 475.5 [M + H]^+^ (37), 475.5 [M + Na]^+^ (100), 513.5 [M + K]^+^ (22); anal. calcd for C_25_H_38_N_4_O_5_: C, 63.27; H, 8.07; N, 11.81. Found: C, 63.26; H, 8.07; N, 11.80.

##### (*S*)-*N*-Isobutyl-2-(4-(2-(isobutylamino)-2-oxoethyl)-3-(naphthalen-1-ylmethyl)-2,5-dioxoimidazolidin-1-yl)-2-methylpropanamide (**8l**)

Amorphous white solid. Yield 91% (169 mg). *R*_*f*_ (AcOEt/hexane, 80:20) = 0.30; [α]_D_^20^ −21.4 (*c* = 0.9, CHCl_3_); ^1^H NMR (400 MHz, CD_3_OD), δ (ppm) = 8.09 (t, *J* = 5.2 Hz, 1H), 8.04–8.02 (m, 1H), 7.77–7.74 (m, 2H), 7.42–7.35 (m, 4H), 4.94 (d, *J* = 15.2 Hz, 1H), 4.82 (d, *J* = 15.2 Hz, 1H), 3.72 (dd, *J* = 4.4 and 2.8 Hz, 1H), 3.00–2.98 (m, 1H), 2.90–2.88 (m, 1H), 2.64–2.62 (m, 2H), 2.51 (dd, *J* = 16.4 and 4.4 Hz, 1H), 2.45 (dd, *J* = 13.2 and 6.8 Hz, 1H), 1.77–1.72 (m, 1H), 1.70 (s, 3H), 1.62 (s, 3H), 1.43–1.41 (m, 1H), 0.83 (d, *J* = 4.4 Hz, 3H), 0.81 (d, *J* = 4.4 Hz, 3H), 0.68 (t, *J* = 6.4 Hz, 6H); ^13^C{^1^H} NMR (101 MHz, CD_3_OD), δ (ppm) = 175.2, 173.6, 169.0, 157.2, 134.4, 132.0, 131.7, 129.1, 128.8, 127.3, 126.6, 126.1, 125.3, 123.5, 78.3, 62.3, 57.1, 46.8, 44.0, 34.2, 28.4, 28.2, 25.0, 24.0, 19.7, 19.4; ESI-MS: *m*/*z* (%) calcd 494.3 [M]^+^. Found 517.4 [M + Na]^+^ (100); anal. calcd for C_28_H_38_N_4_O_4_: C, 67.99; H, 7.74; N, 11.33. Found: C, 68.00; H, 7.73; N, 11.33.

##### (*S*)-2-(4-(2-((2-(1*H*-Indol-3-yl)ethyl)amino)-2-oxoethyl)-3-(naphthalen-1-ylmethyl)-2,5-dioxoimidazolidin-1-yl)-*N*-isobutyl-2-methylpropanamide (**8m**)

Amorphous white solid. Yield 69% (59 mg). *R*_*f*_ (AcOEt) = 0.28; [α]_D_^20^ −36.9 (*c* = 0.9, CHCl_3_); ^1^H NMR (400 MHz, CD_3_OD), δ (ppm) = 7.97–7.94 (m, 1H), 7.73–7.69 (m, 2H), 7.38–7.36 (m, 3H), 7.32–7.30 (m, 2H), 7.23–7.21 (m, 1H), 6.99–6.97 (m, 1H), 7.91–7.86 (m, 2H), 4.85 (d, *J* = 15.2 Hz, 1H), 4.68 (d, *J* = 15.2 Hz, 1H), 3.69 (dd, *J* = 4.8 and 2.8 Hz, 1H), 3.12–3.10 (m, 1H), 2.99–2.95 (m, 2H), 2.88 (dd, *J* = 8.4 and 6.8 Hz, 1H), 2.63–2.58 (m, 3H), 2.42 (dd, *J* = 16.4 and 4.8 Hz, 1H), 1.74–1.71 (m, 1H), 1.71 (s, 3H), 1.63 (s, 3H), 0.78 (d, *J* = 2.8 Hz, 3H), 0.76 (d, *J* = 2.8 Hz, 3H); ^13^C{^1^H} NMR (101 MHz, CD_3_OD), δ (ppm) = 174.8, 173.3, 168.6, 156.9, 136.8, 134.0, 131.5, 131.4, 128.8, 128.5, 127.3, 127.0, 126.3, 125.8, 124.9, 123.2, 121.8, 118.3, 117.9, 111.7, 110.9, 78.0, 61.9, 56.8, 43.6, 39.6, 34.1, 28.2, 24.7, 24.5, 23.7, 19.4; ESI-MS: *m*/*z* (%) calcd 581.3 [M]^+^. Found 604.3 [M + Na]^+^ (100); anal. calcd for C_34_H_39_N_5_O_4_: C, 70.20; H, 6.76; N, 12.04. Found: C, 70.19; H, 6.77; N, 12.05.

##### (*S*)-2-(3-Benzyl-1-(2-((2,2-d iphenylethyl)amino)-2-oxoethyl)-2,5-dioxoimidazolidin-4-yl)-*N*-cyclohexylacetamide (**8n**)

Amorphous white solid. Yield 88% (152 mg). *R*_*f*_ (AcOEt/hexane, 80:20) = 0.18; [α]_D_^20^ −12.9 (*c* = 1.0, CHCl_3_); ^1^H NMR (400 MHz, CDCl_3_), δ (ppm) = 7.72 (br s, 1H), 7.19–7.11 (m, 15H), 5.42 (br s, 1H), 5.54 (d, *J* = 15.2 Hz, 1H), 4.32 (d, *J* = 15.2 Hz, 1H), 4.27 (t, *J* = 7.6 Hz, 1H), 4.13–4.02 (m, 2H), 3.89–3.86 (m, 1H), 3.80 (br s, 1H), 3.75–3.72 (m, 1H), 3.50–3.48 (m, 1H), 2.73 (d, *J* = 14.4 Hz, 1H), 2.38 (d, *J* = 14.4 Hz, 1H), 1.80–1.52 (m, 5H), 1.24–0.97 (m, 5H); ^13^C{^1^H} NMR (101 MHz, CDCl_3_), δ (ppm) = 171.8, 166.6, 155.9, 142.5, 142.3, 135.9, 129.0, 128.4, 128.3, 128.2, 128.1, 128.0, 126.5, 126.4, 56.5, 49.9, 48.9, 45.7, 44.5, 41.9, 34.6, 32.8, 32.7, 25.4, 24.8, 24.7; ESI-MS: *m*/*z* (%) calcd 566.3 [M]^+^. Found 589.4 [M + Na]^+^ (100); anal. calcd for C_34_H_38_N_4_O_4_: C, 72.06; H, 6.76; N, 9.89. Found: C, 72.04; H, 6.77; N, 9.90.

##### (*S*)-2,2′-(3-(Naphthalen-1-ylmethyl)-2,5-dioxoimidazolidine-1,4-diyl)bis(*N*-isobutylacetamide) (**8o**)

Amorphous white solid. Yield 85% (97 mg). *R*_*f*_ (AcOEt/hexane, 80:20) = 0.30; [α]_D_^20^ −39.0 (*c* = 1.0, CHCl_3_); ^1^H NMR (400 MHz, CD_3_OD), δ (ppm) = 8.51 (br s, 0.3H), 8.16–8.14 (m, 1H), 7.92–7.87 (m, 2H), 7.55–7.44 (m, 4H), 5.10 (d, *J* = 15.6 Hz, 1H), 5.02 (d, *J* = 15.6 Hz, 1H), 4.26 (d, *J* = 17.2 Hz, 1H), 4.16 (d, *J* = 17.2 Hz, 1H), 4.07 (dd, *J* = 4.8 and 3.2 Hz, 1H), 3.16–3.14 (m, 1H), 3.12–3.10 (m, 1H), 2.81 (dd, *J* = 16.4 and 2.8 Hz, 1H), 2.74–2.66 (m, 2H), 2.57–2.51 (m, 1H), 1.89–1.85 (m, 1H), 1.55–1.50 (m, 1H), 0.93 (d, *J* = 1.6 Hz, 3H), 0.91 (d, *J* = 1.6 Hz, 3H), 0.79 (d, *J* = 6.0 Hz, 3H), 0.77 (d, *J* = 6.0 Hz, 3H); ^13^C{^1^H} NMR (101 MHz, CD_3_OD), δ (ppm) = 173.0, 168.4, 167.9, 167.8, 156.5, 134.1, 131.4, 128.8, 128.4, 126.8, 126.4, 125.8, 124.9, 123.1, 78.1, 57.32, 57.31, 43.7, 41.0, 33.7, 28.0, 27.9, 19.2, 19.1, 19.0; ESI-MS: *m*/*z* (%) calcd 466.3 [M]^+^. Found 489.2 [M + Na]^+^ (100); anal. calcd for C_26_H_34_N_4_O_4_: C, 66.93; H, 7.35; N, 12.01. Found: C, 66.93; H, 7.37; N, 12.00.

##### (*S*)-*N*-(4-Chlorobenzyl)-2-(1-(2-(isobutylamino)-2-oxoethyl)-3-(naphthalen-1-ylmethyl)-2,5-dioxoimidazolidin-4-yl)acetamide (**8p**)

Amorphous white solid. Yield 81% (131 mg). *R*_*f*_ (AcOEt/hexane, 80:20) = 0.27; [α]_D_^20^ −16.0 (*c* = 1.0, CHCl_3_); ^1^H NMR (400 MHz, CDCl_3_), δ (ppm) = 8.06–8.03 (m, 2H), 7.76–7.74 (m, 2H), 7.45–7.33 (m, 4H), 7.15 (d, *J* = 8.4 Hz, 2H), 6.93 (d, *J* = 8.4 Hz, 2H), 6.39 (br t, *J* = 5.6 Hz, 1H), 5.01 (d, *J* = 14.8 Hz, 1H), 4.86 (d, *J* = 14.8 Hz, 1H), 4.15 (d, *J* = 17.2 Hz, 1H), 4.04 (d, *J* = 17.2 Hz, 1H), 4.02 (dd, *J* = 14.8 and 6.4 Hz, 1H), 3.73–3.72 (m, 1H), 3.61 (dd, *J* = 14.8 and 5.2 Hz, 1H), 2.99–2.97 (m, 1H), 2.85–2.72 (m, 2H), 2.57 (dd, *J* = 16.4 and 4.8 Hz, 1H), 1.67 (septet, *J* = 6.8 Hz, 1H), 0.72 (d, *J* = 6.8 Hz, 6H); ^13^C{^1^H} NMR (101 MHz, CDCl_3_), δ (ppm) = 171.9, 167.9, 167.2, 155.8, 136.0, 133.9, 133.4, 131.5, 131.2, 129.6, 128.8, 128.7, 127.8, 127.2, 126.5, 125.2, 123.6, 56.8, 47.4, 44.4, 42.6, 41.8, 34.5, 28.0, 20.22, 20.20; ESI-MS: *m*/*z* (%) calcd 534.2 [M]^+^. Found 557.2 [M + Na]^+^ (100); anal. calcd for C_29_H_31_ClN_4_O_4_: C, 65.10; H, 5.84; N, 10.47. Found: C, 65.09; H, 5.85; N, 10.46.

##### (*S*)-*N*-Isobutyl-2-(3-(naphthalen-1-ylmethyl)-4-(2-((naphthalen-1-ylmethyl)amino)-2-oxoethyl)-2,5-dioxoimidazolidin-1-yl)acetamide (**8q**)

Amorphous white solid. Yield 81% (153 mg). *R*_*f*_ (AcOEt) = 0.43; [α]_D_^20^ −28.5 (*c* = 1.0, CHCl_3_); ^1^H NMR (400 MHz, CD_3_OD), δ (ppm) = 8.01–7.95 (m, 2H), 7.75–7.68 (m, 4H), 7.37–7.33 (m, 8H), 5.00 (d, *J* = 15.2 Hz, 1H), 4.86 (d, *J* = 15.2 Hz, 1H), 4.82 (d, *J* = 15.2 Hz, 1H), 4.73 (d, *J* = 15.2 Hz, 1H), 4.24 (d, *J* = 17.2 Hz, 1H), 4.15 (d, *J* = 17.2 Hz, 1H), 3.95 (dd, *J* = 4.8 and 3.2 Hz, 1H), 2.64 (dd, *J* = 16.8 and 3.2 Hz, 1H), 2.53 (dd, *J* = 16.8 and 4.8 Hz, 1H), 2.34 (dd, *J* = 13.2 and 6.8 Hz, 1H), 2.18 (dd, *J* = 13.2 and 6.4 Hz, 1H), 1.22 (septet, *J* = 6.8 Hz, 1H), 0.50 (d, *J* = 6.8 Hz, 3H), 0.48 (d, *J* = 6.8 Hz, 3H); ^13^C{^1^H} NMR (101 MHz, CD_3_OD), δ (ppm) = 173.0, 168.3, 167.9, 156.5, 134.0, 133.8, 133.1, 131.3, 131.2, 128.7, 128.4, 128.3, 127.7, 126.6, 126.4, 125.9, 125.4, 125.3, 125.0, 124.9, 123.0, 122.9, 57.2, 46.2, 43.6, 41.1, 40.8, 33.7, 27.8, 18.94, 18.92; ESI-MS: *m*/*z* (%) calcd 550.3 [M]^+^. Found 573.3 [M + Na]^+^ (100); anal. calcd for C_33_H_34_N_4_O_4_: C, 71.98; H, 6.22; N, 10.17. Found: C, 71.98; H, 6.22; N, 10.15.

##### (*S*)-*N*-(4-Chlorobenzyl)-2-(4-(2-((2,2-diphenylethyl)amino)-2-oxoethyl)-3-(naphthalen-1-ylmethyl)-2,5-dioxoimidazolidin-1-yl)acetamide (**8r**)

Crystalline white solid. Yield 79% (127 mg). *R*_*f*_ (AcOEt) = 0.38; mp 132–134 °C; [α]_D_^20^ +11.5 (*c* = 1.0, CHCl_3_); ^1^H NMR (400 MHz, CDCl_3_), δ (ppm) = 8.40 (br t, *J* = 6.0 Hz, 1H), 7.91 (d, *J* = 8.4 Hz, 1H), 7.82–7.80 (m 1H), 7.76–7.73 (m, 1H), 7.46–7.34 (m, 2H), 7.28–7.20 (m, 5H), 7.17–7.13 (m, 4H), 7.05–6.93 (m, 7H), 4.87 (d, *J* = 15.2 Hz, 1H), 4.84 (br s, 1H), 4.70 (d, *J* = 15.2 Hz, 1H), 4.50 (dd, *J* = 15.2 and 6.4 Hz, 1H), 4.29 (d, *J* = 17.2 Hz, 1H), 4.24 (d, *J* = 17.2 Hz, 1H), 4.20 (dd, *J* = 11.2 and 5.2 Hz, 1H), 3.71 (t, *J* = 8.0 Hz, 1H), 3.65–3.64 (m, 1H), 3.16–3.14 (m, 1H), 2.89–2.87 (m, 1H), 2.65 (dd, *J* = 16.4 and 2.4 Hz, 1H), 2.10 (dd, *J* = 16.4 and 4.8 Hz, 1H); ^13^C{^1^H} NMR (101 MHz, CDCl_3_), δ (ppm) = 171.7, 167.3, 166.6, 155.8, 141.23, 141.21, 137.1, 133.8, 132.6, 131.5, 131.2, 129.5, 128.9, 128.87, 128.85, 128.7, 128.4, 128.3, 127.8, 127.7, 127.3, 127.1, 127.0, 126.6, 125.0, 123.5, 56.8, 50.0, 44.5, 43.2, 42.5, 42.1, 34.6; ESI-MS: *m*/*z* (%) calcd 658.2 [M]^+^. Found 681.5 [M + Na]^+^ (100); anal. calcd for C_39_H_35_ClN_4_O_4_: C, 71.06; H, 5.35; N, 8.50. Found: C, 71.04; H, 5.36; N, 8.51.

## Supporting Information Available

Copies of the ^1^H, ^13^C NMR, and MS spectra for all new compounds and copies of the VT ^1^H NMR and DMSO titration ^1^H NMR experiments (PDF)
