# Supplementary material for: Synthesis and Conformational Analysis of Hydantoin-Based Universal Peptidomimetics
Source: J Org Chem. 2022 Oct 13;88(15):10381–402. doi: 10.1021/acs.joc.2c01903 (PMC10407853; doi:10.1021/acs.joc.2c01903)
Supplement: Supplementary file 1 — jo2c01903_si_001.pdf [file jo2c01903_si_001.pdf]

SUPPORTING INFORMATION

Synthesis and Conformational Analysis of Hydantoin-Based Universal Peptidomimetics

Alessio M. Caramiello,[†] Maria Cristina Bellucci,[§] Gaetano Cristina,[†] Carlo Castellano,[ç] Fiorella Meneghetti,[¥] Matteo Mori,[¥] Francesco Secundo,[‡] Fiorenza Viani,[‡] Alessandro Sacchetti,[†,*] Alessandro Volonterio[†,‡,*]

[†] Department of Chemistry, Material and Chemical Engineering "Giulio Natta", Politecnico di Milano, via mancinelli 7, 20311 Milano, ITALY.

[§] Department of Food, Environmental and Nutritional Sciences, Università degli Studi di Milano, via Celoria 2, 20133 Milano, Italy .

[¥] Department of Pharmaceutical Sciences, Università degli Studi di Milano, via Mangiagalli 25, 20133 Milano, Italy.

[ç] Department of Chemistry, Università degli Studi di Milano, via Golgi 19, 20133 Milano.

[‡] Consiglio Nazionale delle Ricerche, Istituto di Scienze e Tecnologie Chimiche "G. Natta" (SCITEC), via Mario Bianco 9, 20131 Milan, Italy

alessandro.volonterio@polimi.it; alessandro.sacchetti@polimi.it

Table of contents

Pages S2-S117	Copies of the ^{1}H NMR, ^{13}C NMR and ESI MS spectra of all new compounds
Page S118	Figure S1. Results from Monte Carlo/MM conformational analysis.
Pages S119-S123	Figures S2-S6. Copies of the VT ^{1}H NMR experiments
Pages S124-S125	Figures S7-S8. Copies of the ^{1}H NMR DMSO titrations experiments
Page S126	Table S1. Selected H-bond distances and angles.

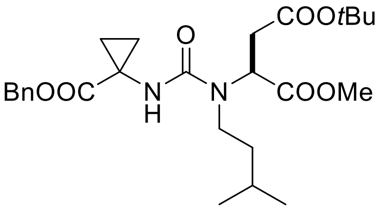

4a

^{1}H-NMR (400 MHz, $CDCl_3$)

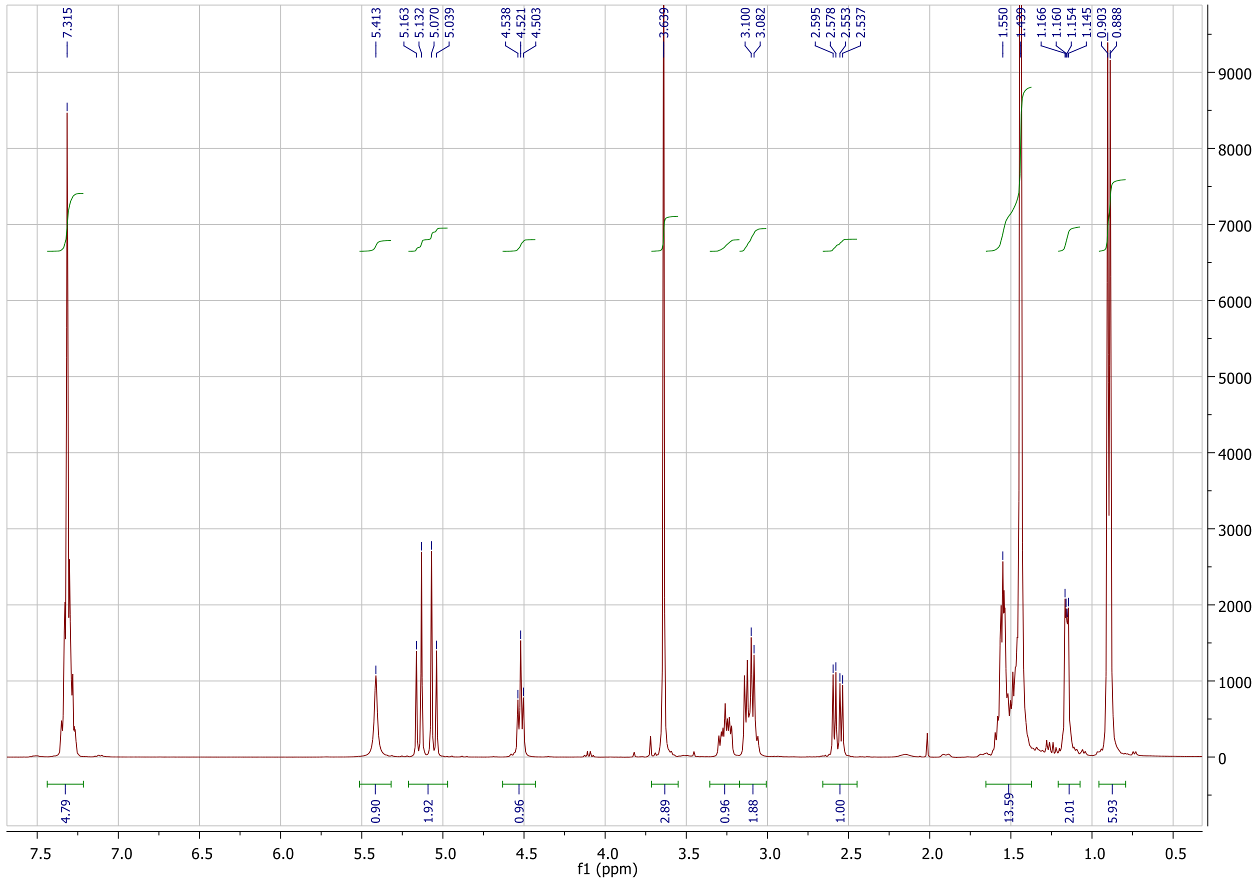

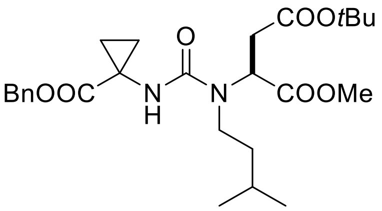

4a

$^{13}C\{^{1}H\}$-NMR (101 MHz, $CDCl_3$)

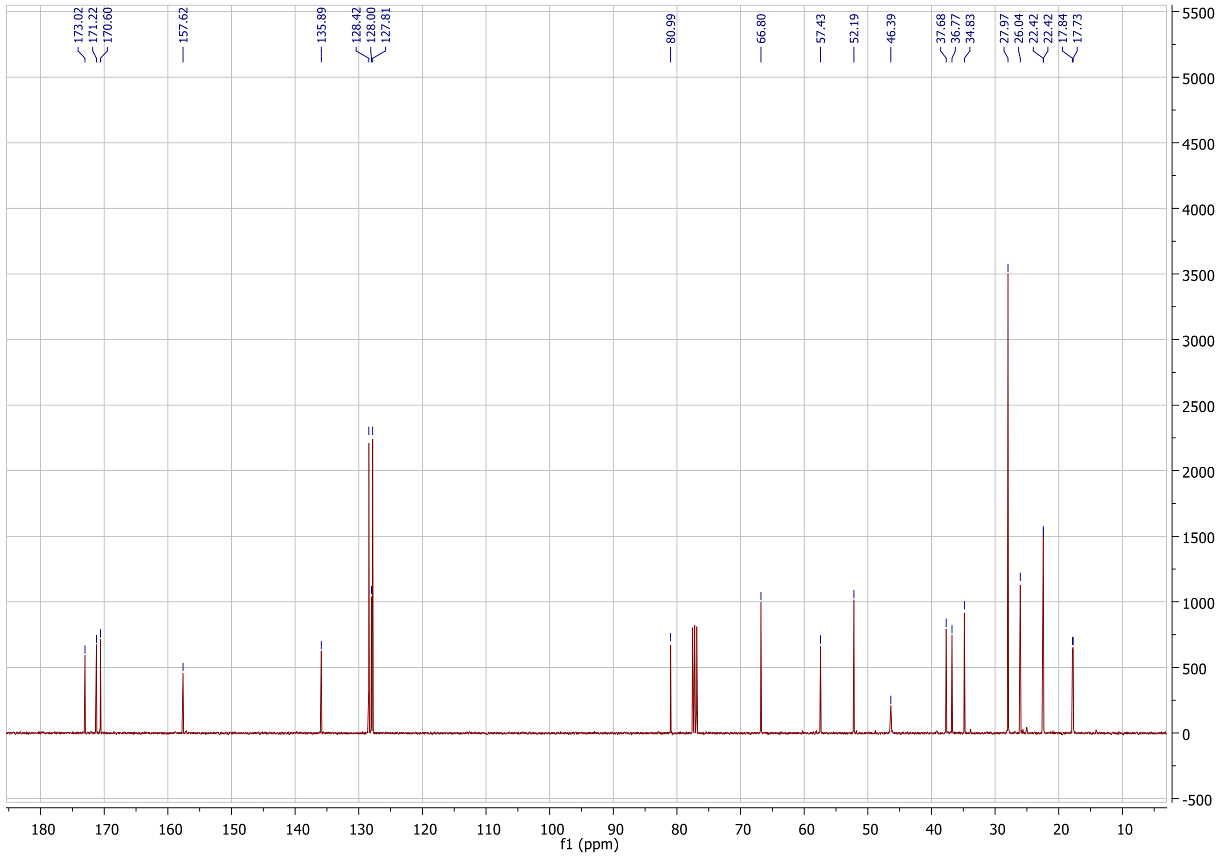

-L.G.S. - Laboratorio Grandi Strumenti - Display Report

Analysis Name	av gc22b.d	Acquisition Date	02/08/18 14:19:50	Operator	Walter Panzeri
Sample Name		Method	Copy of _01tmix_posneg lm.MS	Instrument	esquire3000plus
Comment	1 mg/ml dil 1:100 CH3CN Richiedente: Cristina				

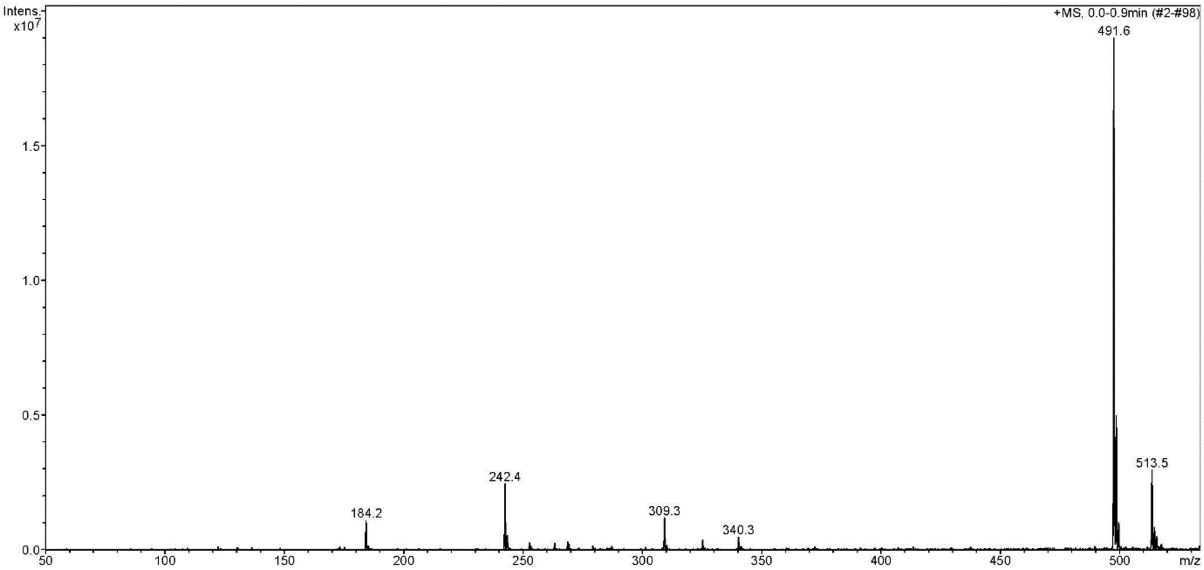

Bruker Daltonics DataAnalysis 3.1 printed: 02/08/18 14:21:50 Page 1 of 1

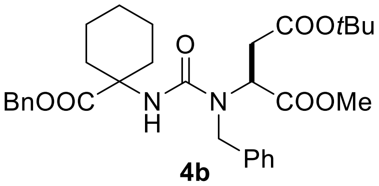

4b

^{1}H-NMR (400 MHz, CDCl$_3$)

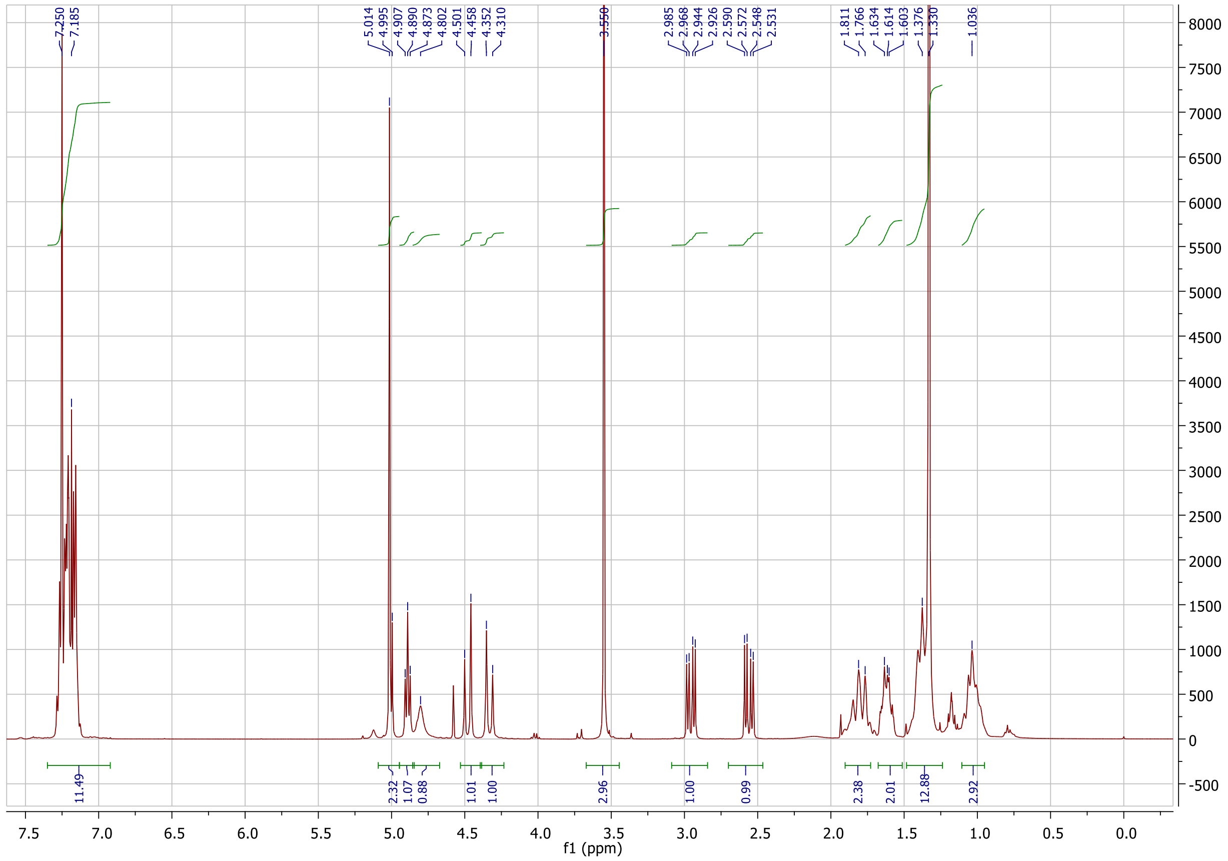

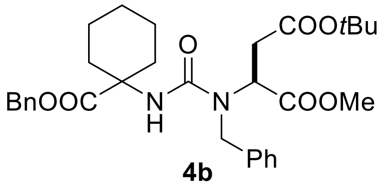

4b

$^{13}C\{^{1}H\}$-NMR (101 MHz, $CDCl_3$)

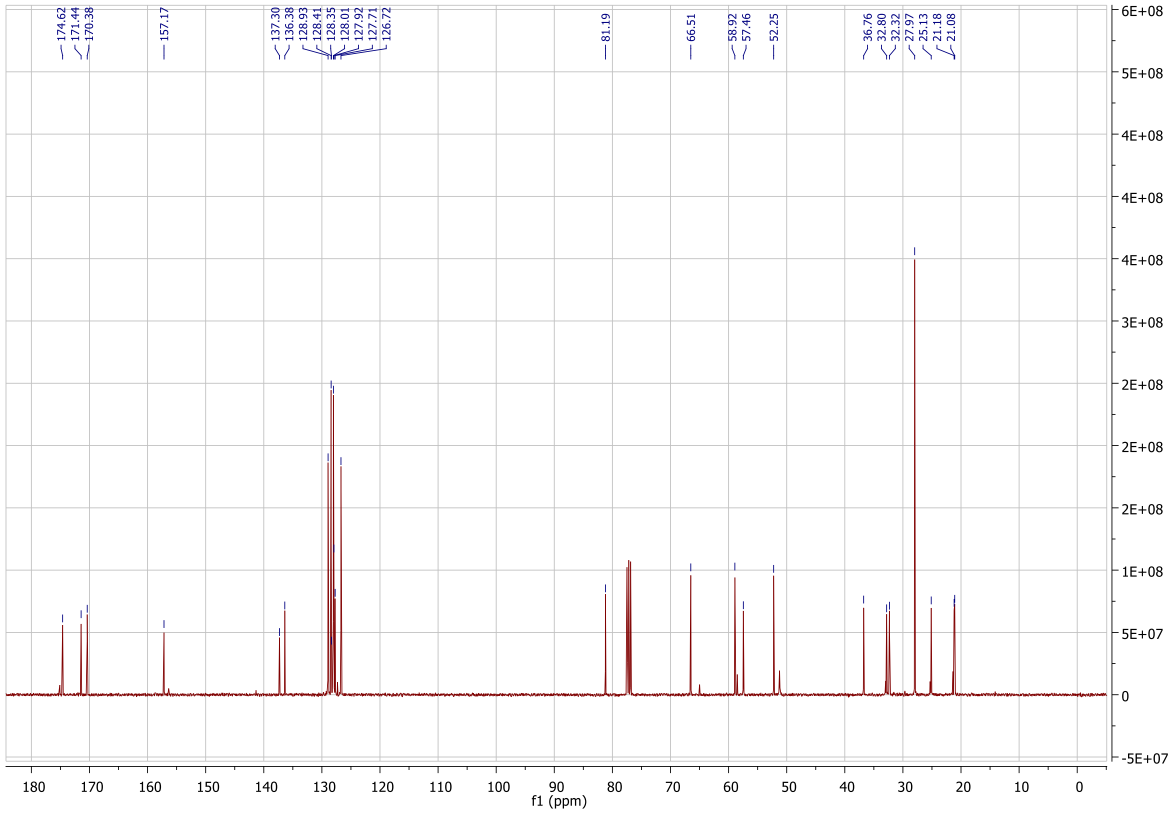

-L.G.S. - Laboratorio Grandi Strumenti - Display Report

Analysis Name	av gc83.d	Acquisition Date	09/24/18 15:47:02	Operator	Walter Panzeri
Sample Name		Method	Copy of _01tmix_posneg lm.MS	Instrument	esquire3000plus
Comment	1 mg/ml dil 1:100 MeOH<br>Richiedente: Cristina				

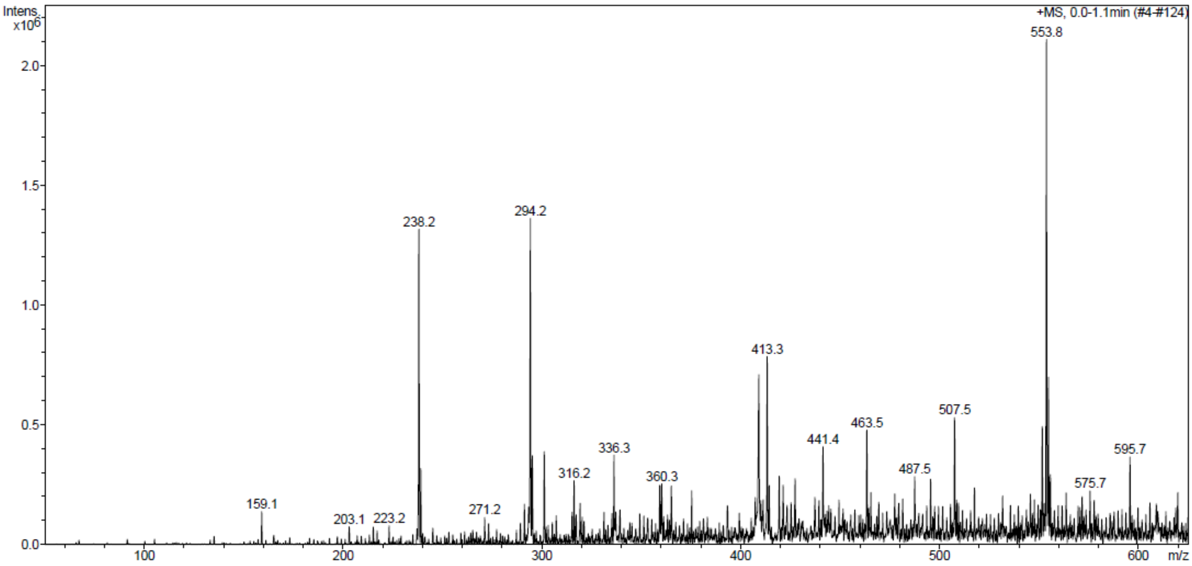

Bruker Daltonics DataAnalysis 3.1 printed: 09/24/18 15:50:40 Page 1 of 1

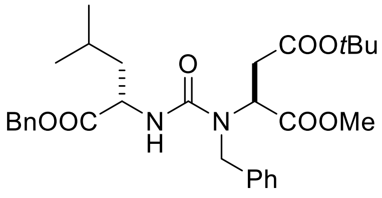

4c

^{1}H-NMR (400 MHz, $CDCl_3$)

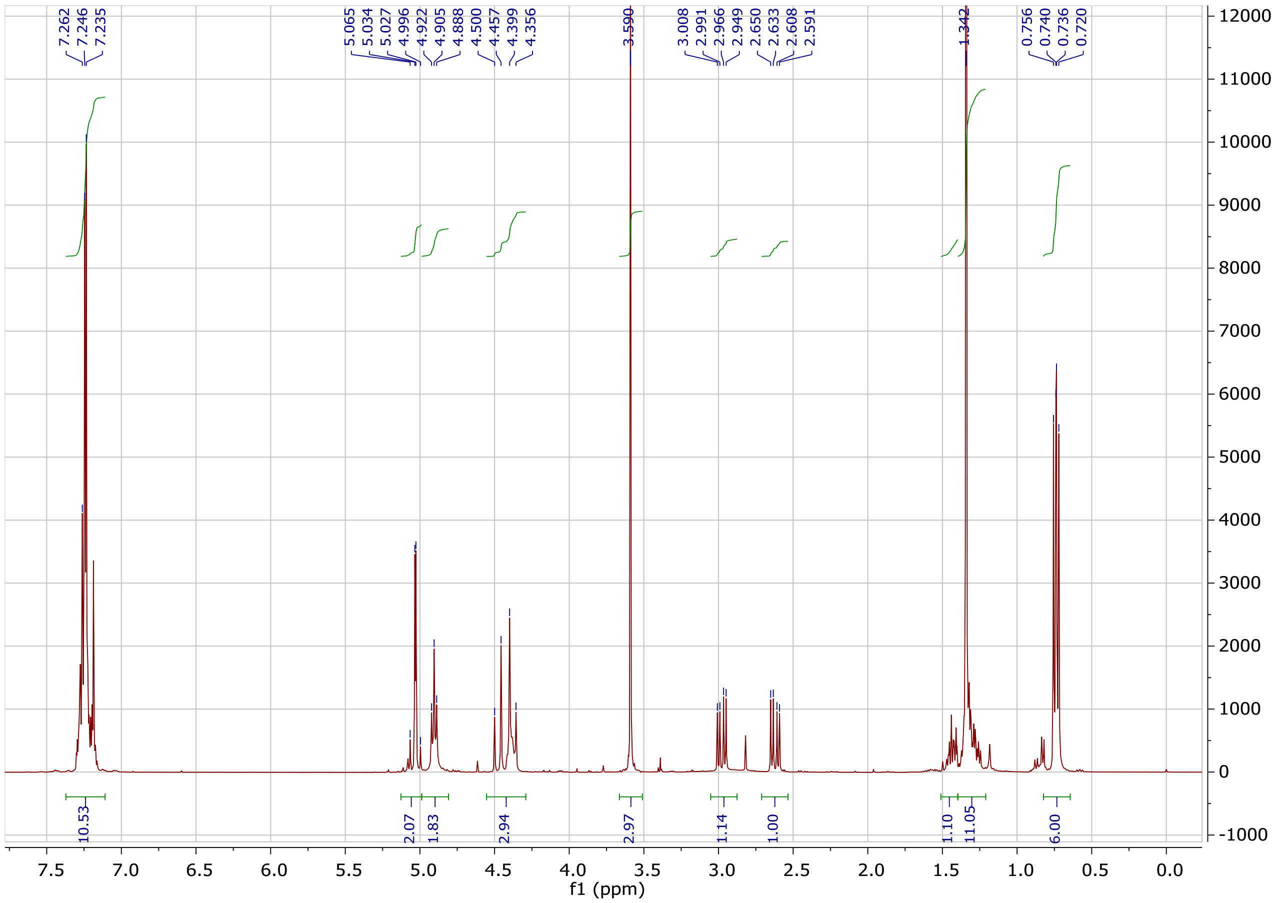

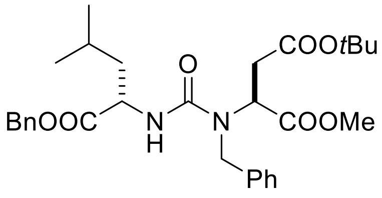

4c

$^{13}C\{^1H\}$-NMR (101 MHz, $CDCl_3$)

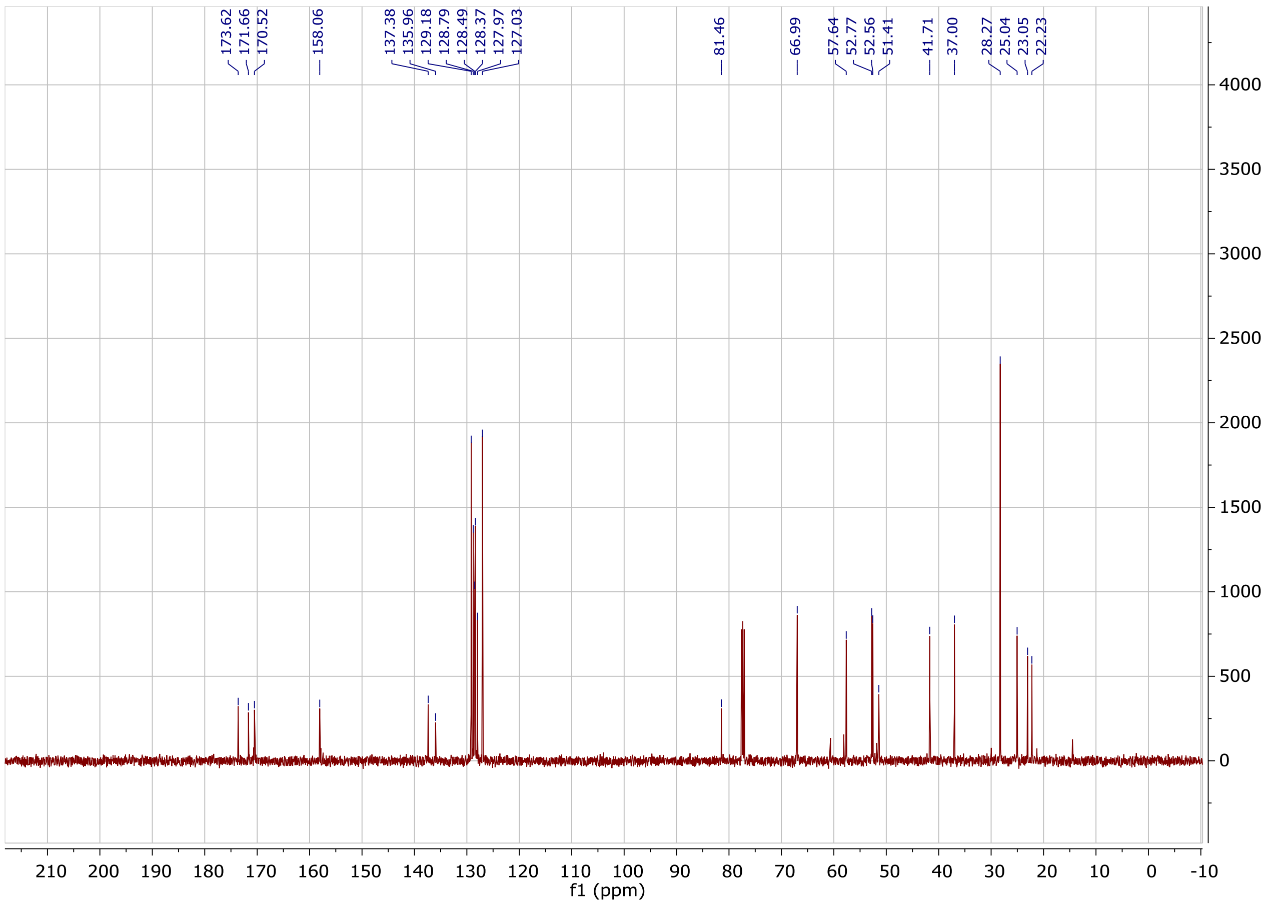

-L.G.S. - Laboratorio Grandi Strumenti - Display Report

Analysis Name	av gc85a.d	Acquisition Date	10/02/18 13:53:06	Operator	Walter Panzeri
Sample Name		Method	Copy of _01tmix_posneg lm.MS	Instrument	esquire3000plus
Comment	1 mg/ml dil 1:100 MeOH Richiedente Cristina				

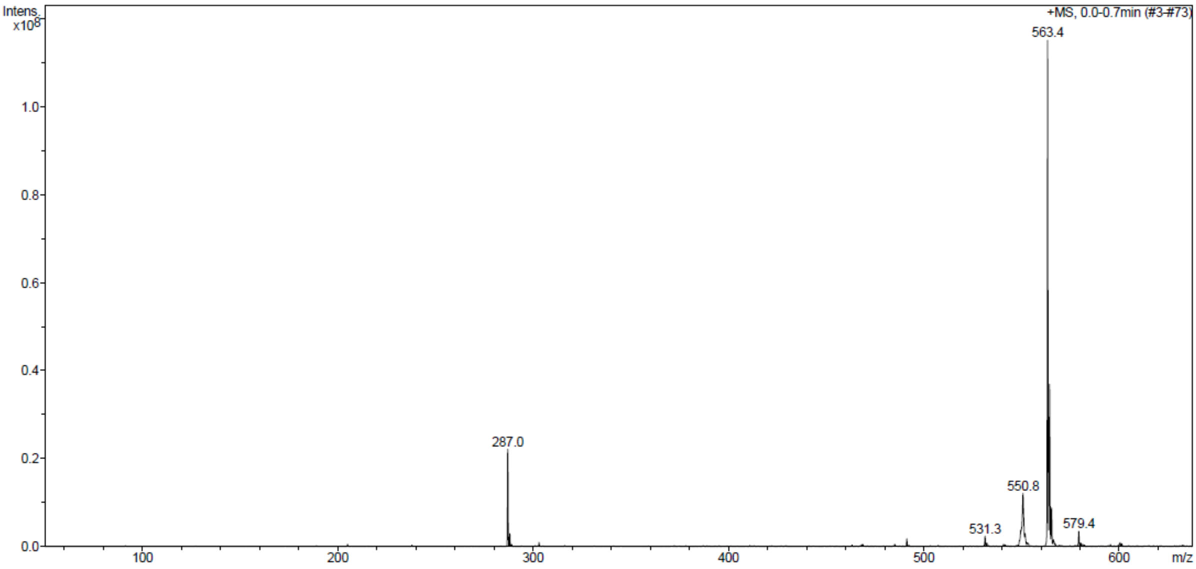

Bruker Daltonics DataAnalysis 3.1 printed: 10/02/18 13:54:50 Page 1 of 1

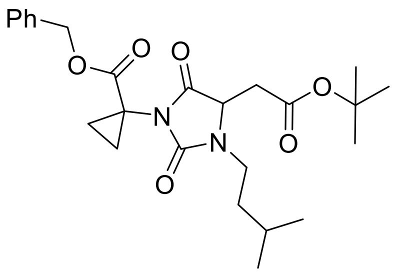

5a

^{1}H-NMR (400 MHz, $CDCl_3$)

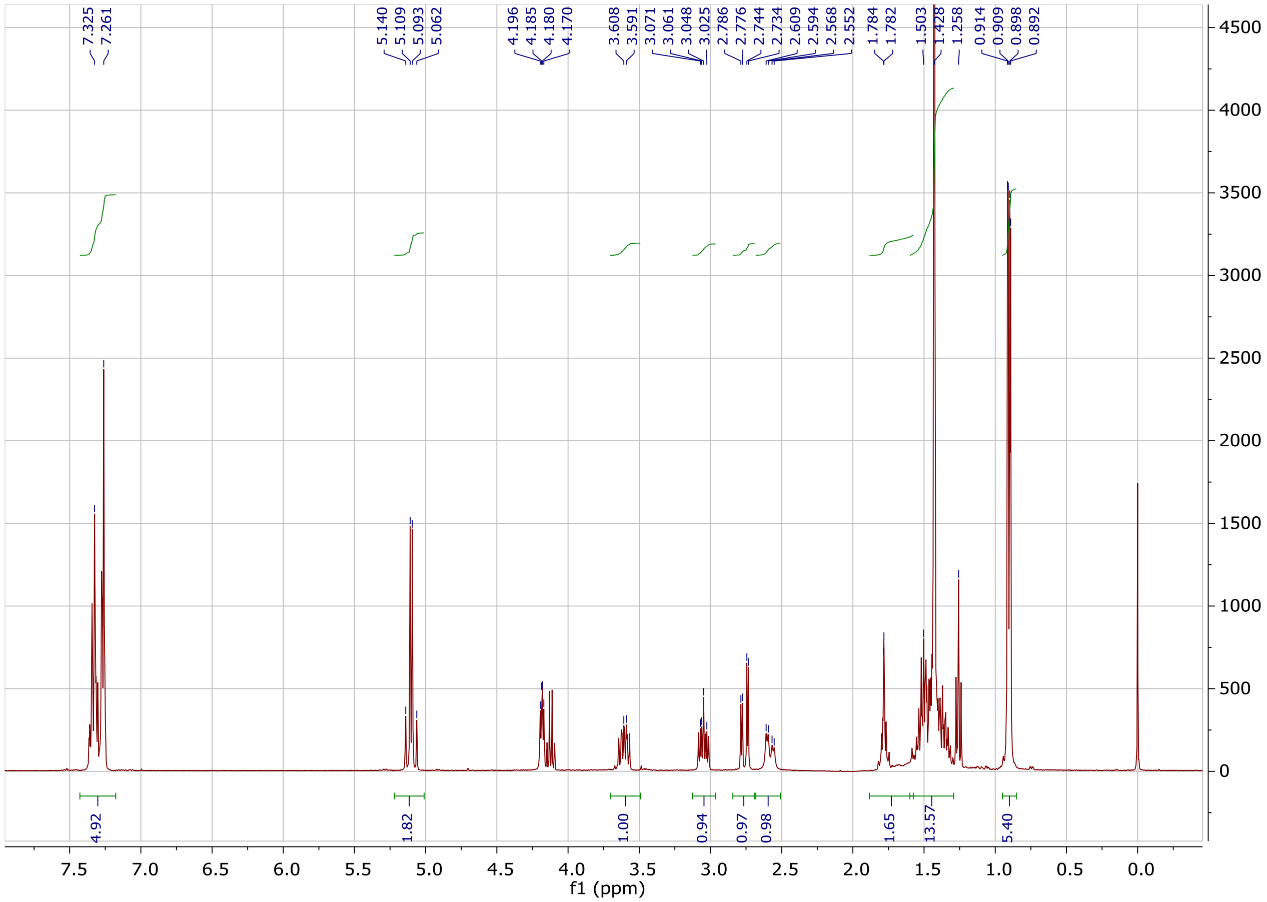

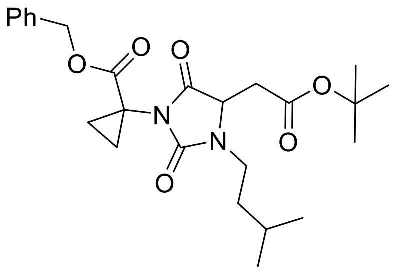

5a

$^{13}C\{^{1}H\}$-NMR (101 MHz, $CDCl_3$)

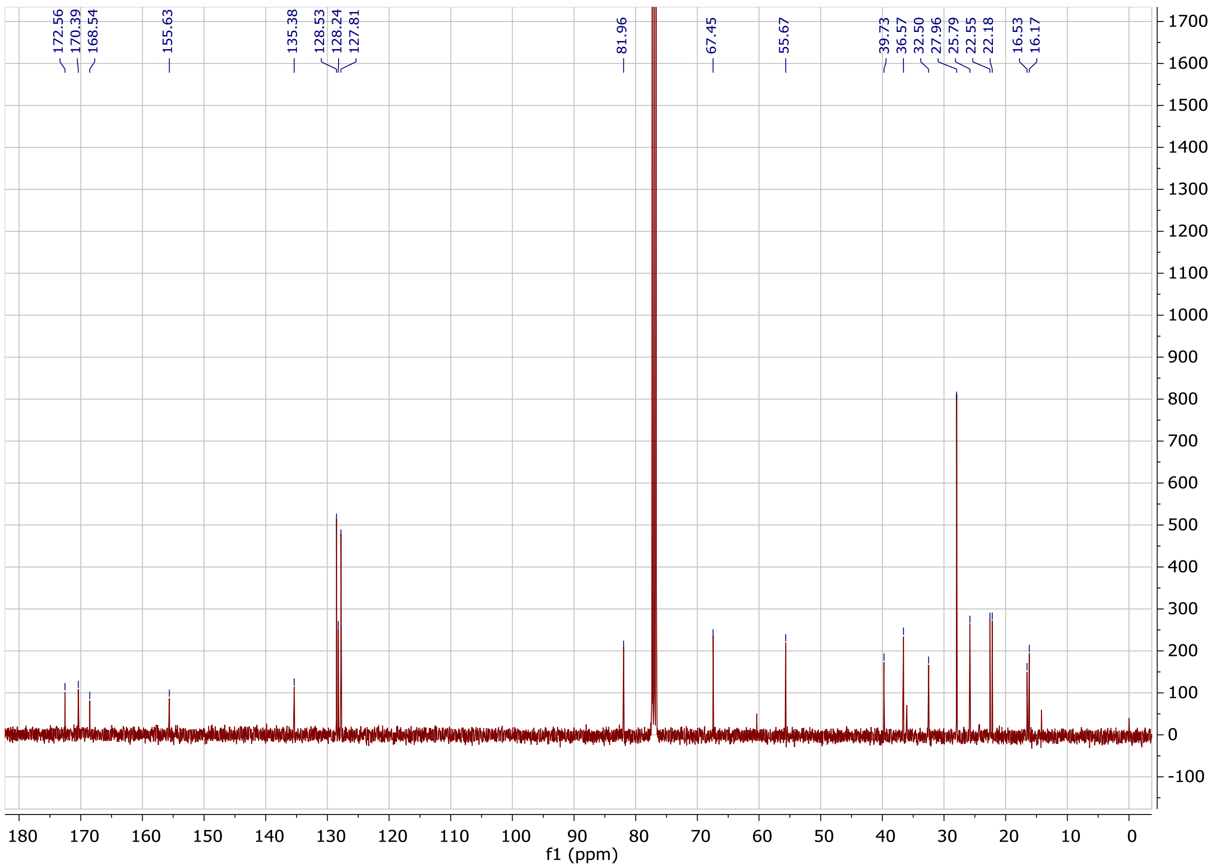

-L.G.S. - Laboratorio Grandi Strumenti - Display Report

Analysis Name	av gc118.d	Acquisition Date	03/26/19 13:57:44	Operator	Walter Panzeri
Sample Name		Method	Copy of _01tmix_posneg lm.MS	Instrument	esquire3000plus
Comment	1 mg/ml dil 1:100 MeOH<br>Richiedente: Cristina				

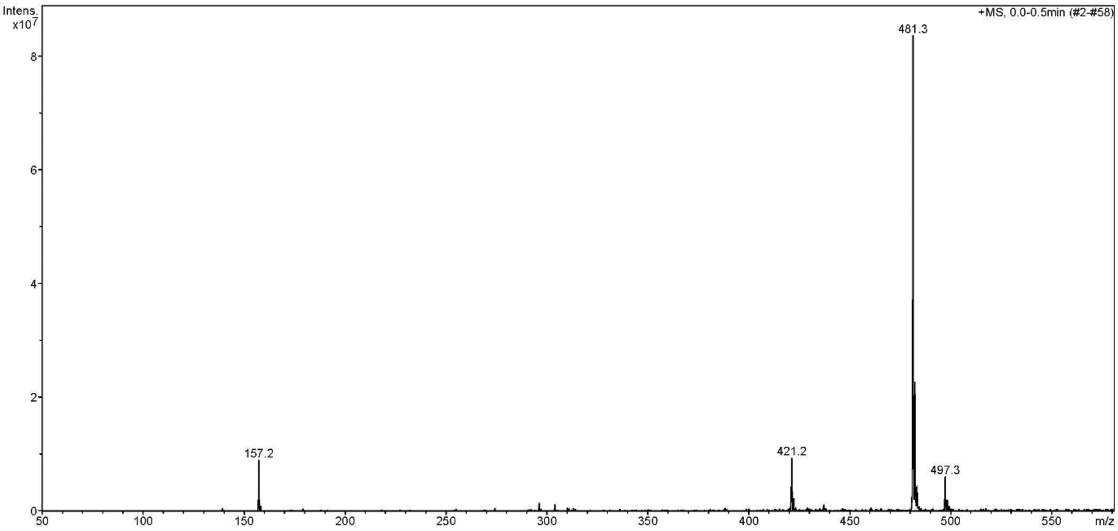

Bruker Daltonics DataAnalysis 3.1 printed: 03/26/19 13:58:54 Page 1 of 1

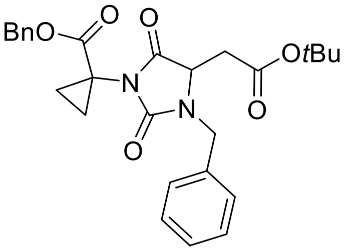

5b

^{1}H-NMR (400 MHz, $CDCl_3$)

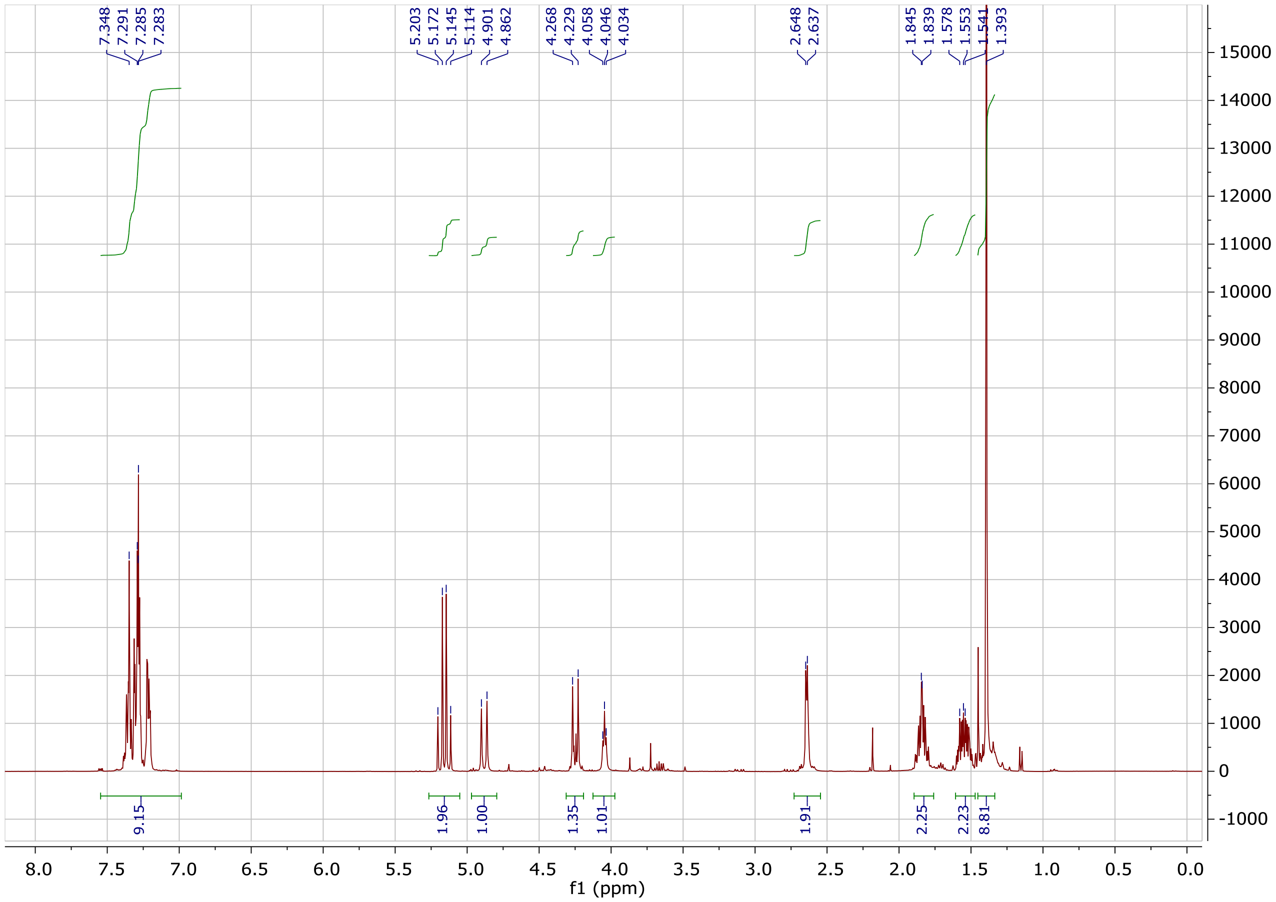

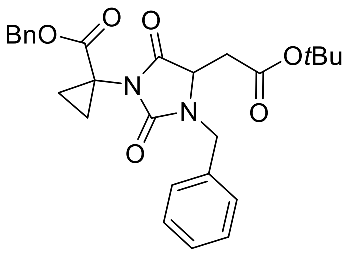

5b

$^{13}C\{^{1}H\}$-NMR (101 MHz, $CDCl_3$)

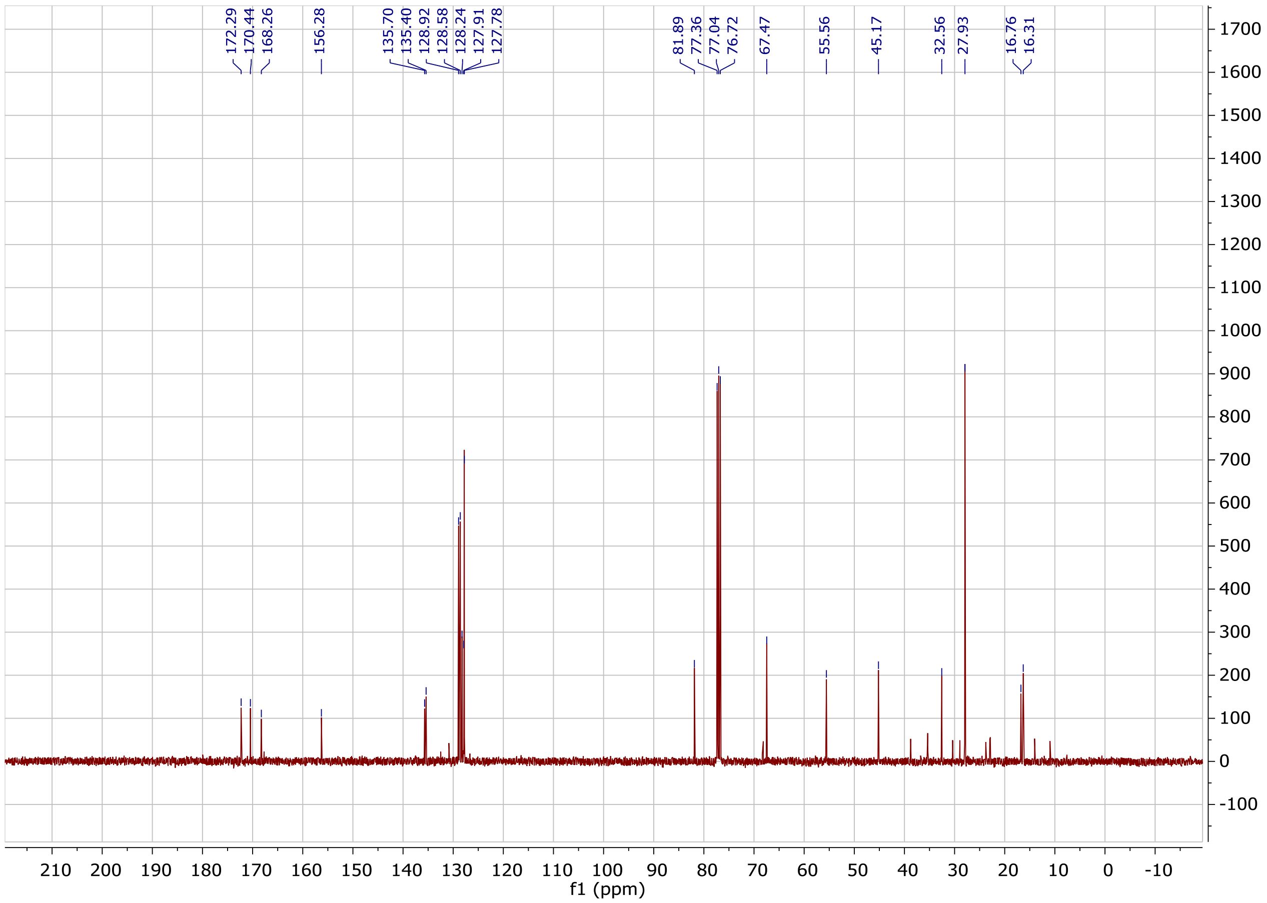

-L.G.S. - Laboratorio Grandi Strumenti - Display Report

Analysis Name	av gc103.d	Acquisition Date	12/12/18 15:30:33	Operator	Walter Panzeri
Sample Name		Method	Copy of _01tmix_posneg lm.MS	Instrument	esquire3000plus
Comment	1 mg/ml dil 1:100 MeOH<br>Richiedente: Cristina				

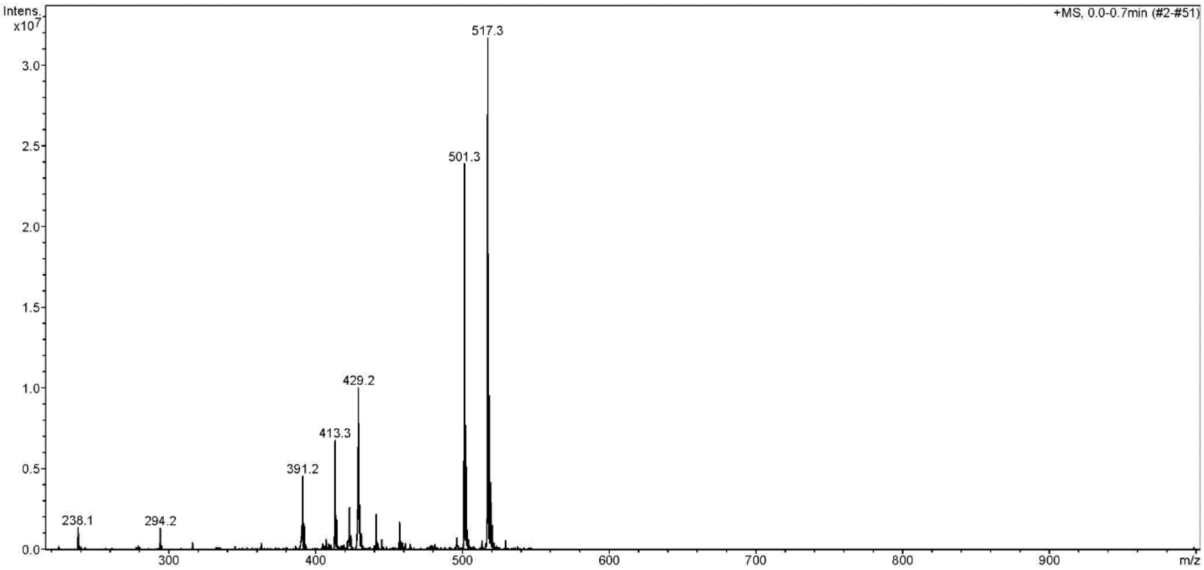

Bruker Daltonics DataAnalysis 3.1 printed: 12/12/18 15:32:18 Page 1 of 1

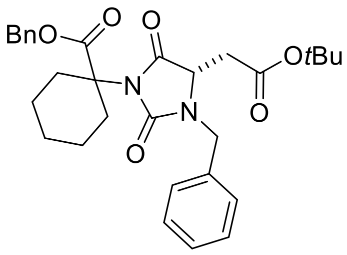

5c

^{1}H-NMR (400 MHz, $CDCl_3$)

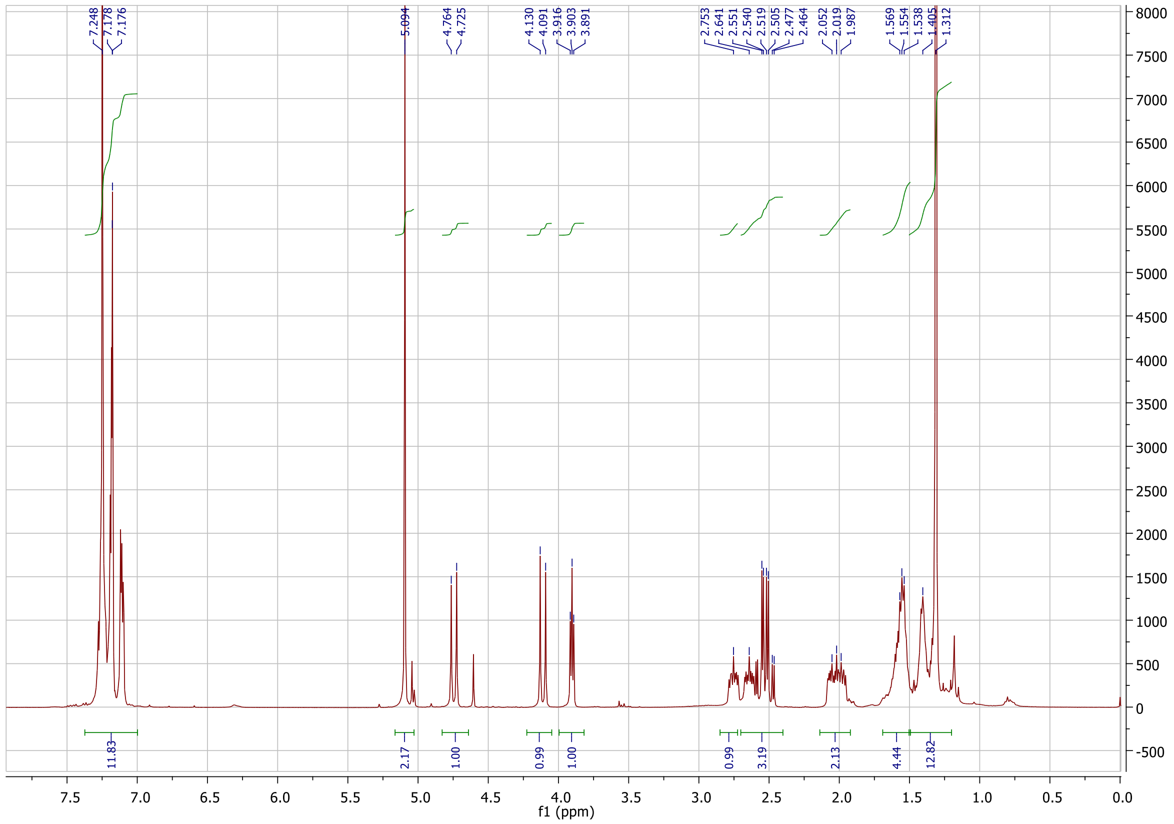

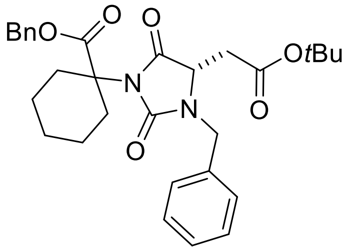

5c

$^{13}C\{^{1}H\}$-NMR (101 MHz, $CDCl_3$)

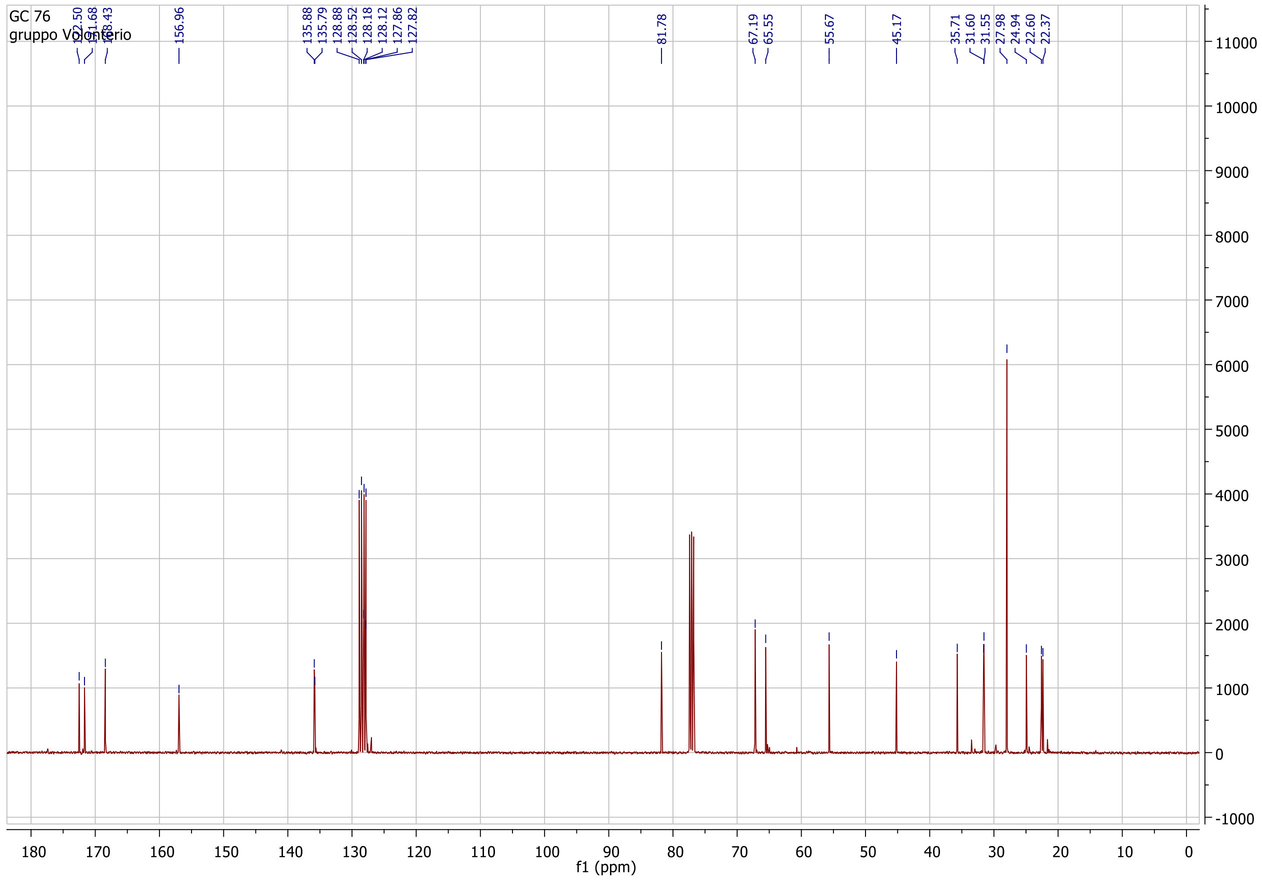

-L.G.S. - Laboratorio Grandi Strumenti - Display Report

Analysis Name	av gc86.d	Acquisition Date	10/04/18 15:39:51	Operator	Walter Panzeri
Sample Name		Method	Copy of _01tmix_posneg lm.MS	Instrument	esquire3000plus
Comment	1 mg/ml dil 1:100 MeOH Richiedente: Cristina				

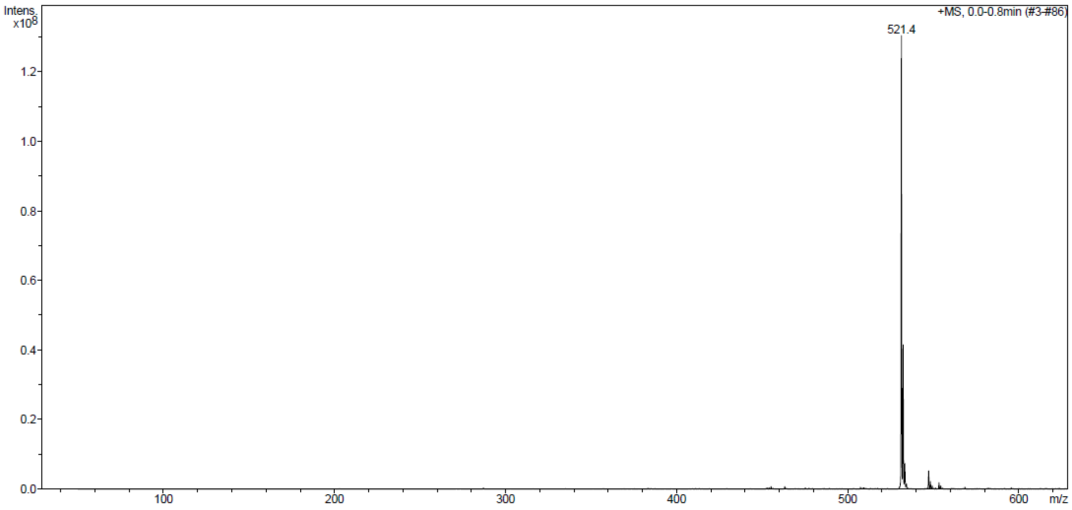

Bruker Daltonics DataAnalysis 3.1 printed: 10/04/18 15:58:57 Page 1 of 1

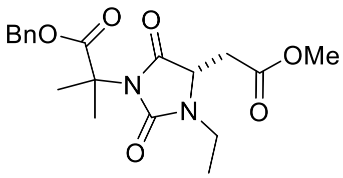

5d

^{1}H-NMR (400 MHz, dmso-d_6)

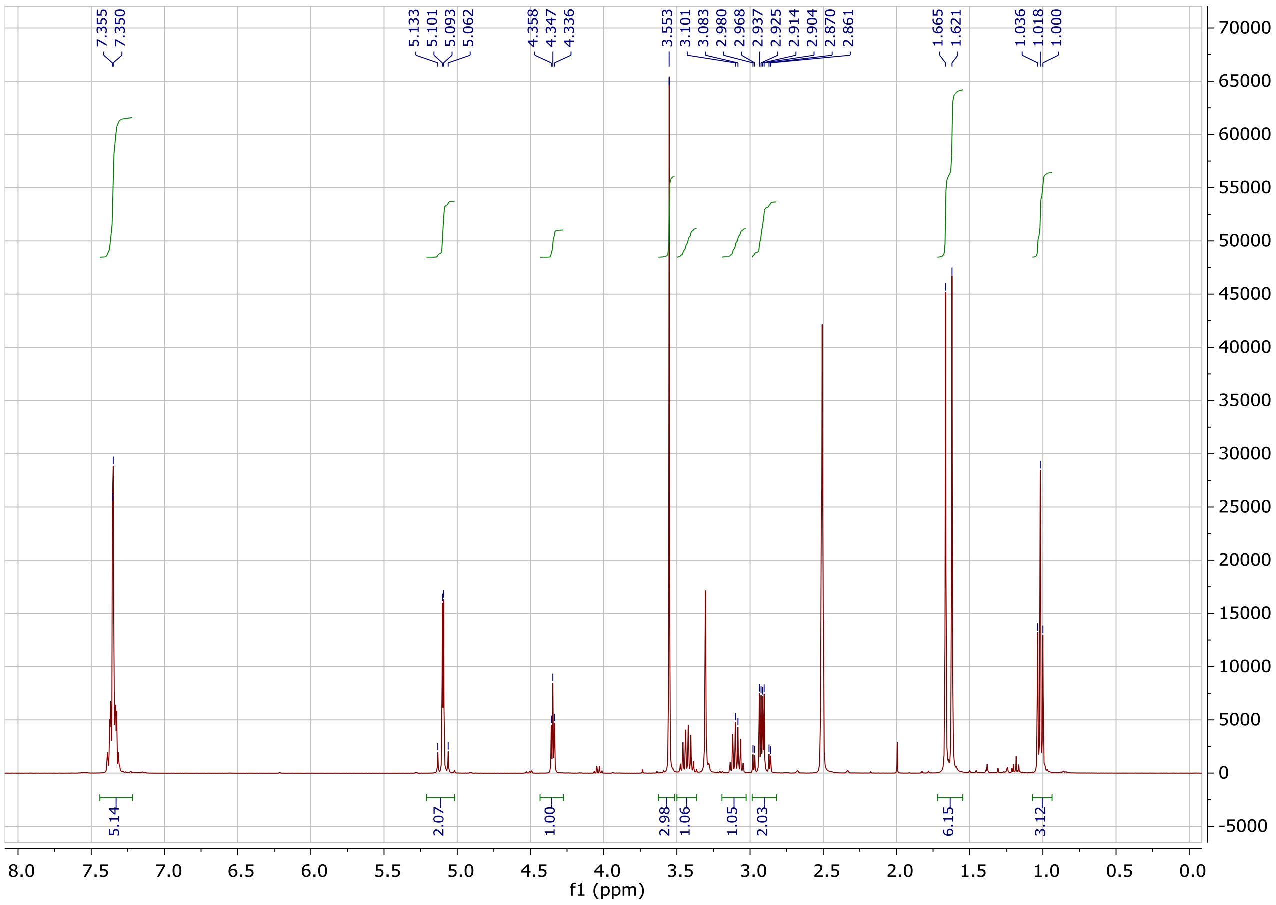

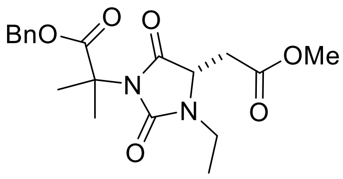

5d

$^{13}C\{^{1}H\}$-NMR (101 MHz, $CDCl_3$)

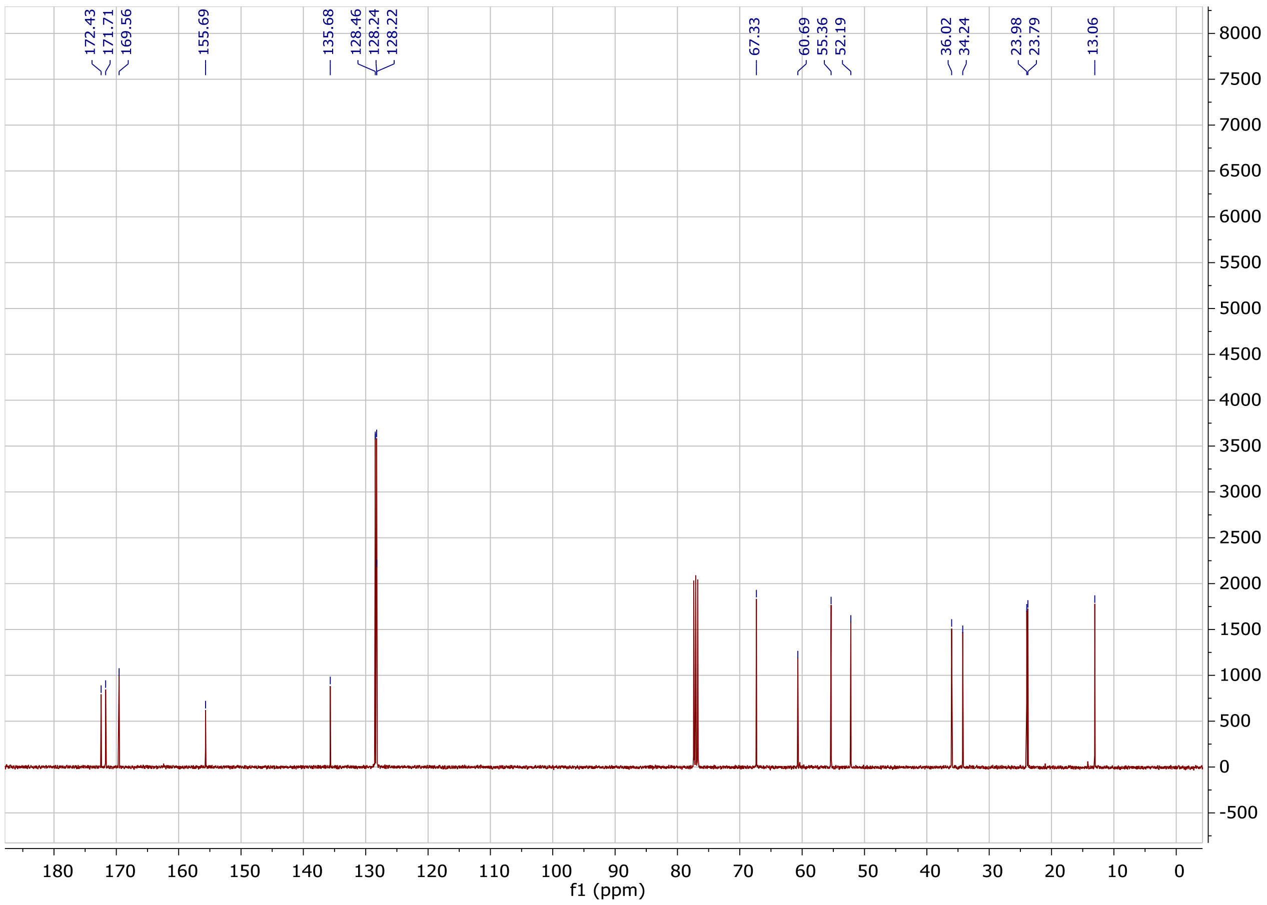

-L.G.S. - Laboratorio Grandi Strumenti - Display Report

Analysis Name	av gc18.d	Acquisition Date	01/17/18 14:38:06	Operator	Administrator
Sample Name		Method	Copy of _01tmix_posneg lm.MS	Instrument	esquire3000plus
Comment	1 mg/ml dil:1:100 MeOH Richiedente: Cristina Gaetano				

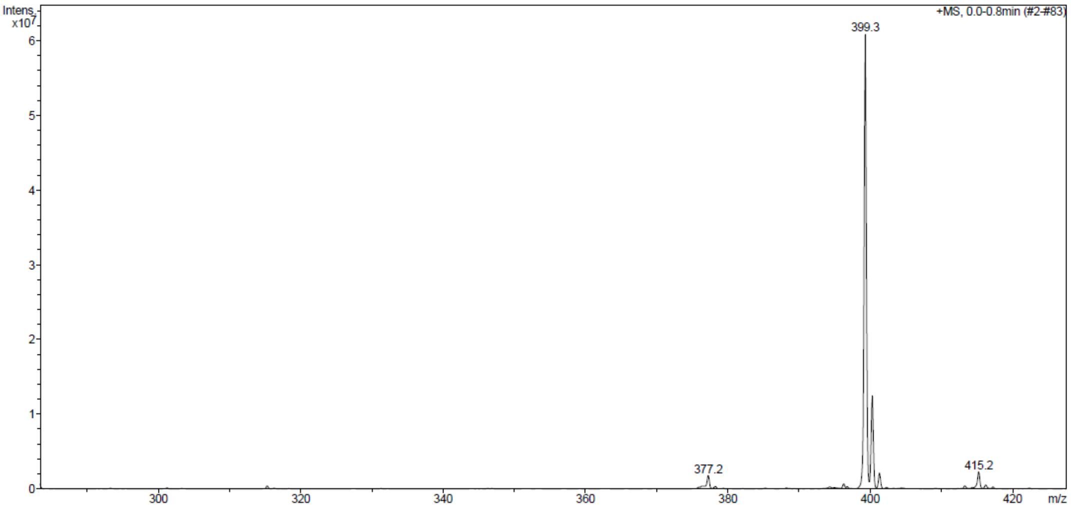

Bruker Daltonics DataAnalysis 3.1 printed: 01/17/18 14:39:24 Page 1 of 1

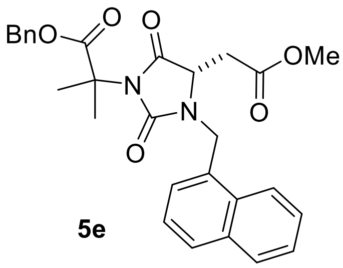

5e

^{1}H-NMR (400 MHz, $CDCl_3$)

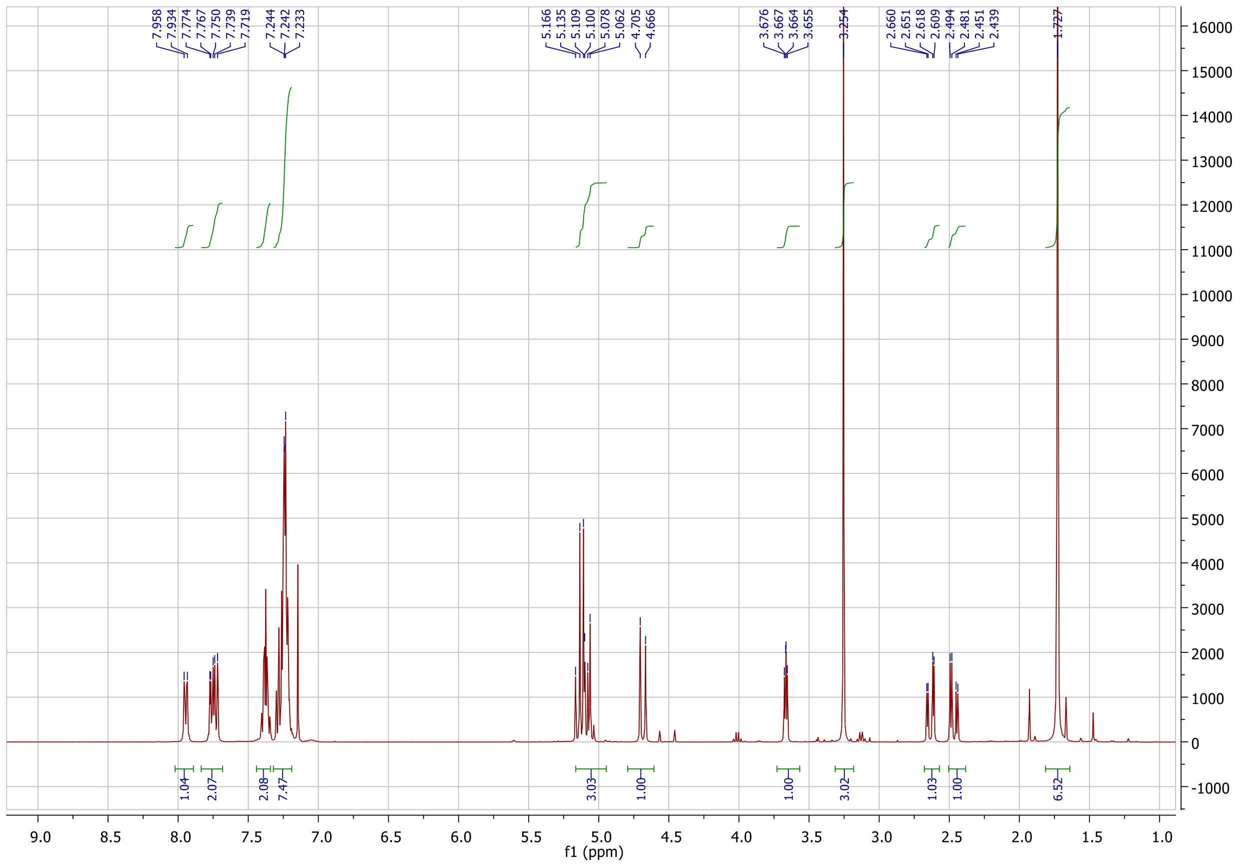

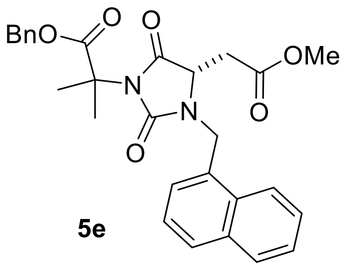

5e

$^{13}C\{^1H\}$-NMR (101 MHz, $CDCl_3$)

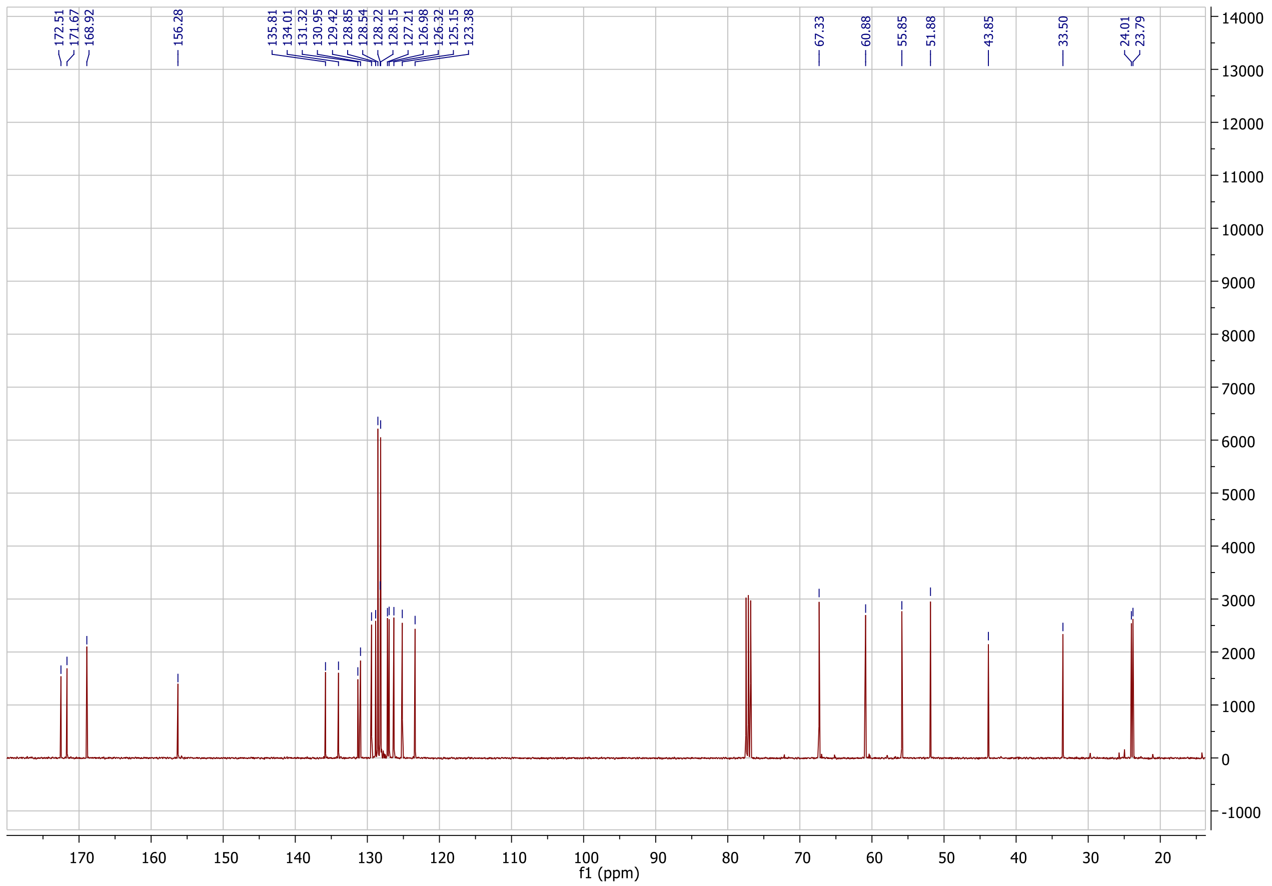

-L.G.S. - Laboratorio Grandi Strumenti - Display Report

Analysis Name	av gc48.d	Acquisition Date	05/18/18 10:45:14	Operator	Walter Panzeri
Sample Name		Method	Copy of _01tmix_posneg lm.MS	Instrument	esquire3000plus
Comment	1 mg/ml dil 1:100 MeOH<br>Richiedente: Cristina				

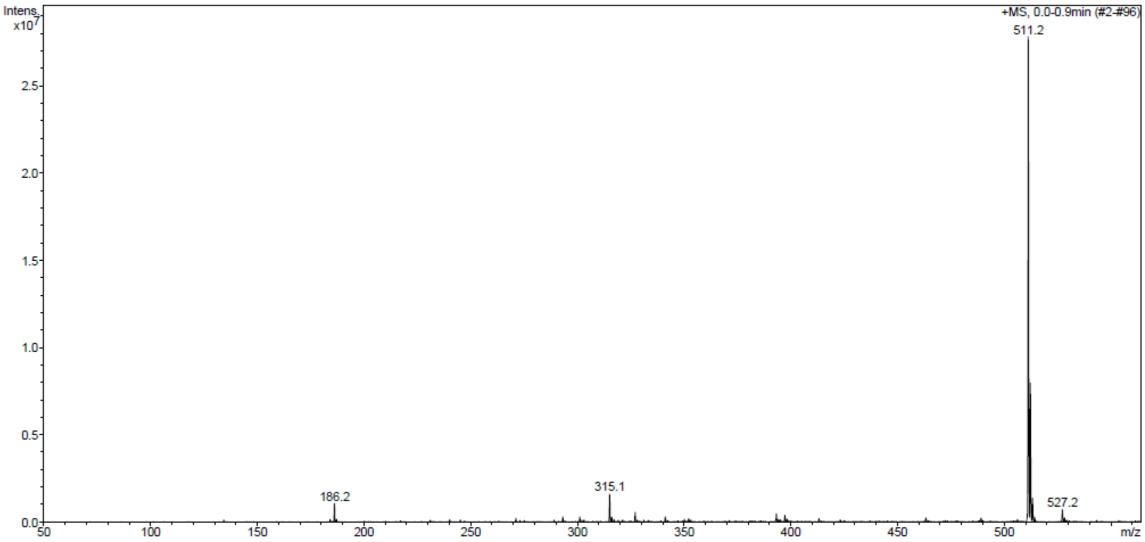

Bruker Daltonics DataAnalysis 3.1 printed: 05/18/18 10:47:00 Page 1 of 1

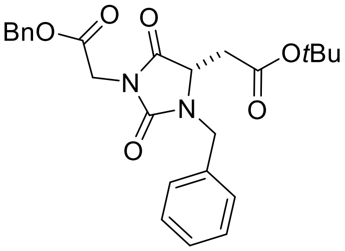

5f

^{1}H-NMR (400 MHz, $CDCl_3$)

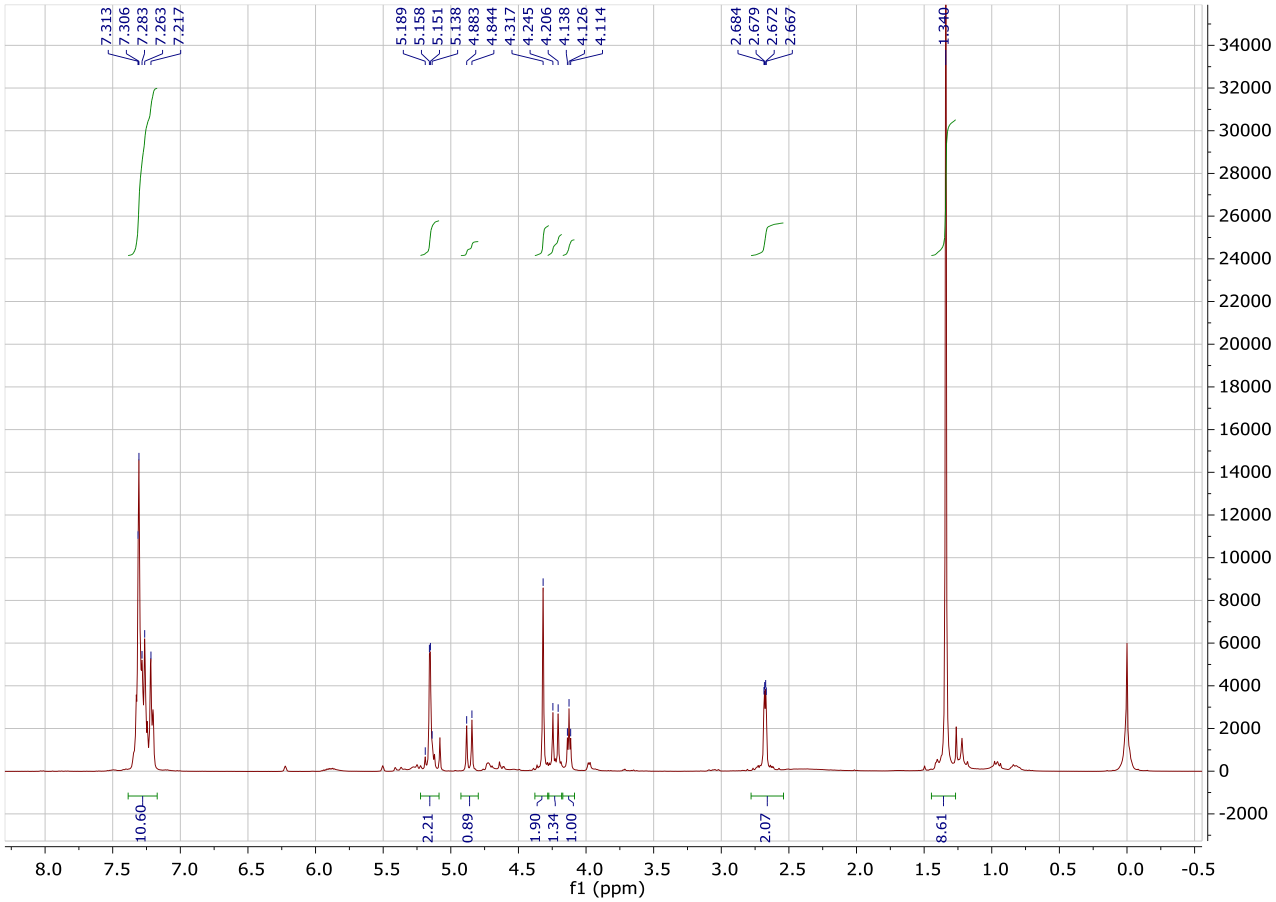

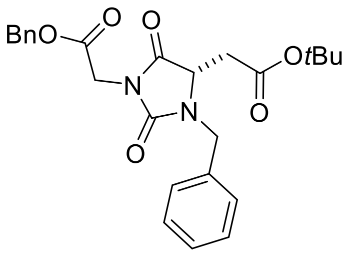

5f

$^{13}C\{^{1}H\}$-NMR (101 MHz, $CDCl_3$)

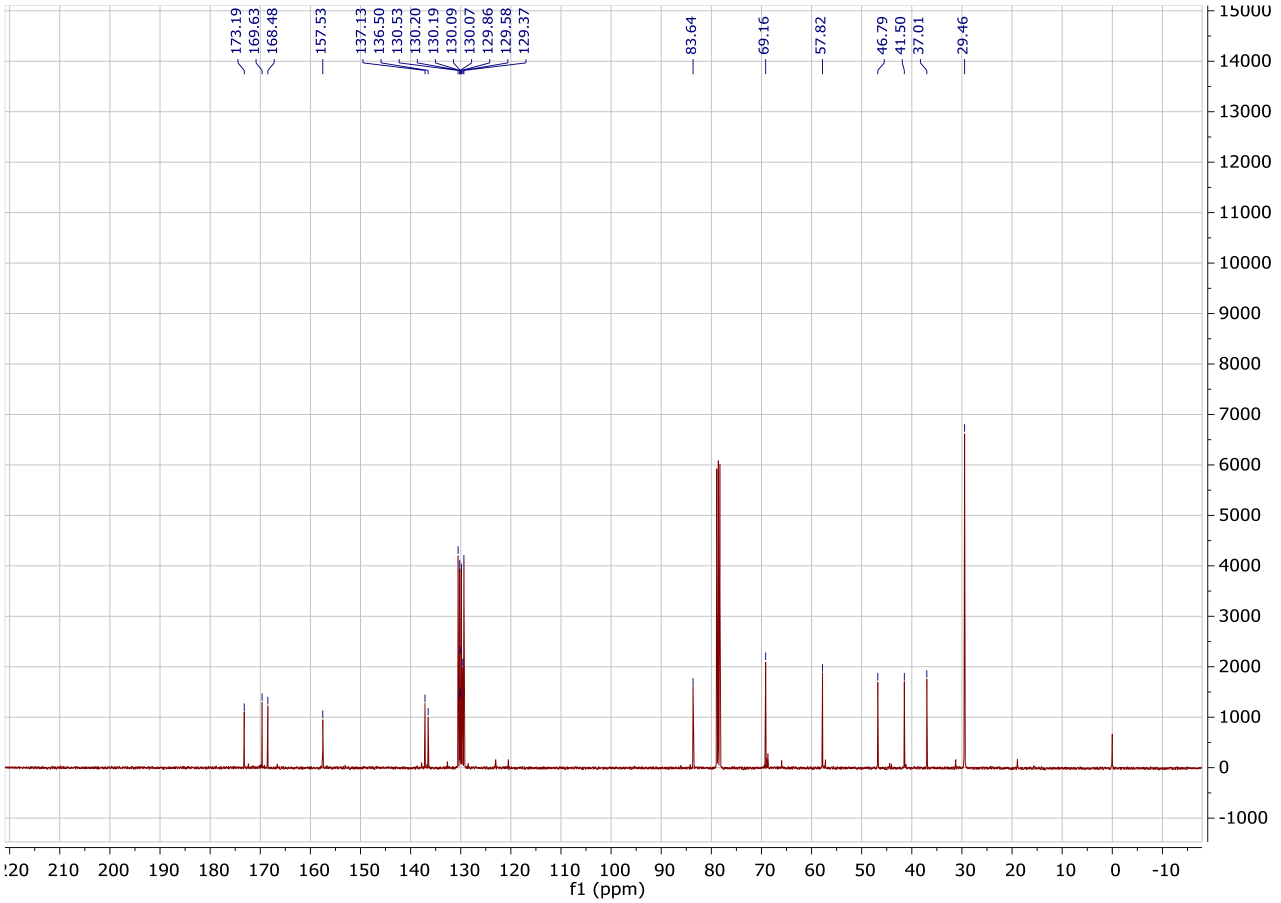

-L.G.S. - Laboratorio Grandi Strumenti - Display Report

Analysis Name	av ac050p.d	Acquisition Date	07/12/22 13:16:09	Operator	Walter Panzeri
Sample Name		Method	Copy of $opt0622.MS	Instrument	esquire3000plus
Comment	1 mg/mL dil 1:100 MeOH<br>Richiedente: Caramiello				

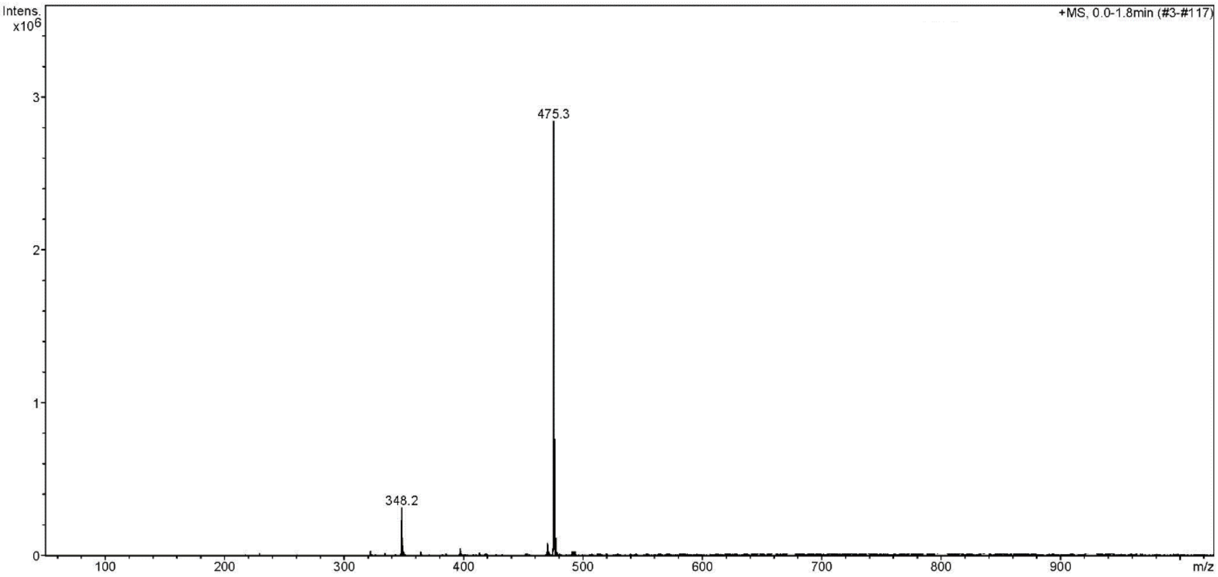

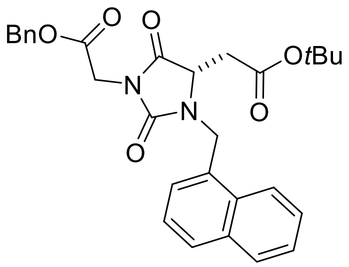

5g

^{1}H-NMR (400 MHz, $CDCl_3$)

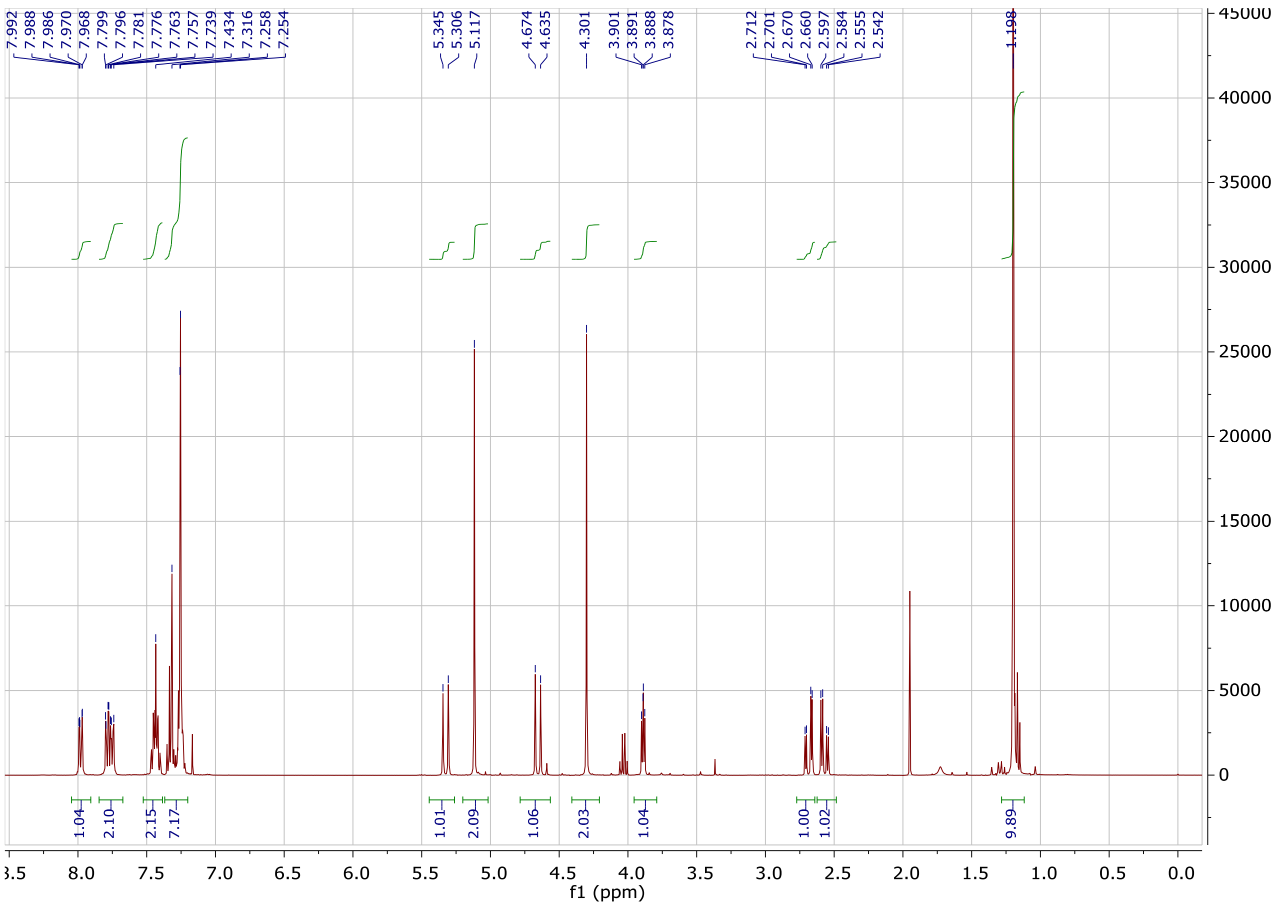

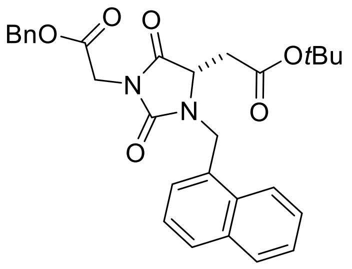

5g

$^{13}C\{^{1}H\}$-NMR (101 MHz, $CDCl_3$)

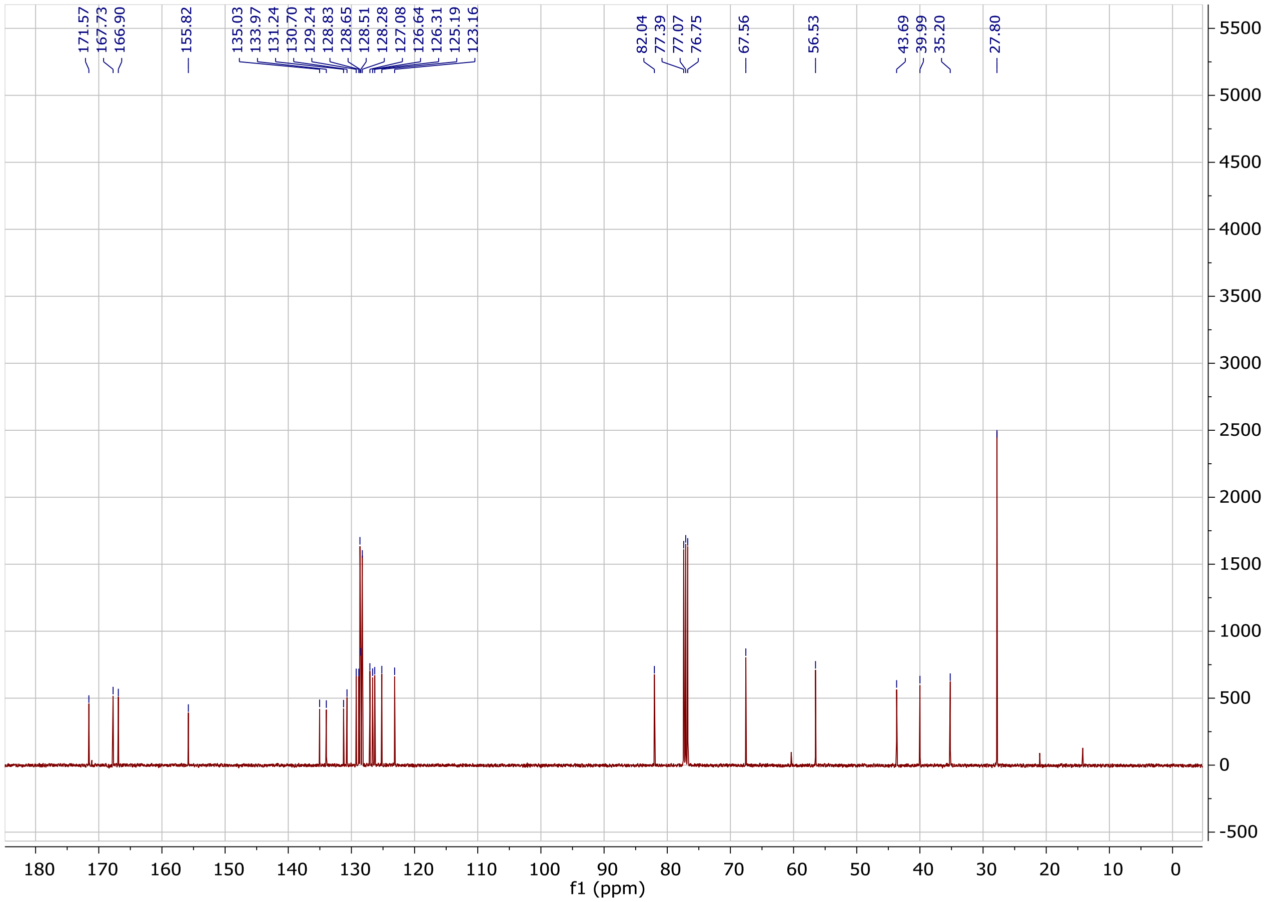

-L.G.S. - Laboratorio Grandi Strumenti - Display Report

Analysis Name	av ac067.d	Acquisition Date	07/24/20 16:24:44	Operator	Administrator
Sample Name		Method	Copy of _01tmix_posneg lm.MS	Instrument	esquire3000plus
Comment	1mg/ml dil 1:100 MeOH Richiedente: Caramiello				

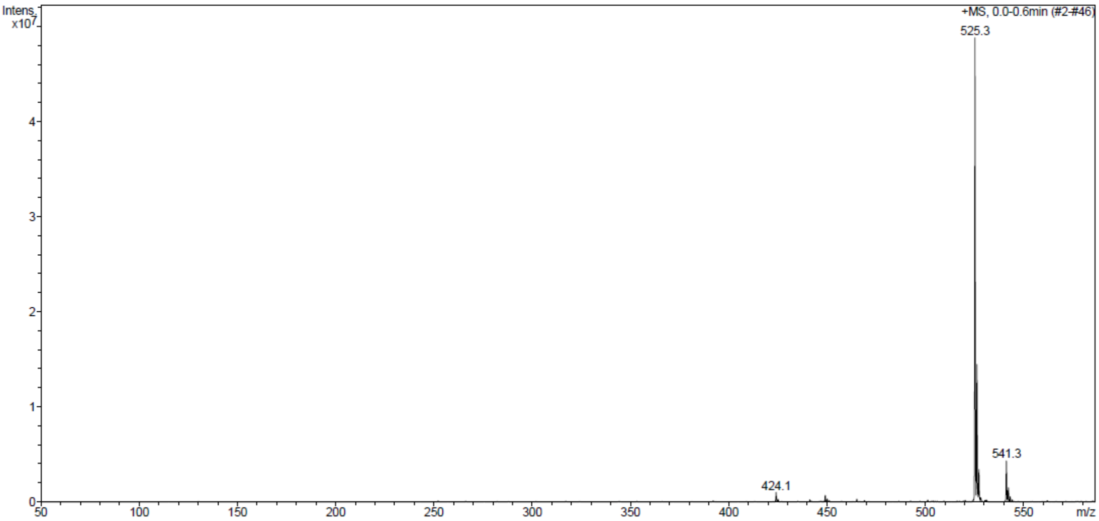

Bruker Daltonics DataAnalysis 3.1 printed: 07/24/20 16:25:33 Page 1 of 1

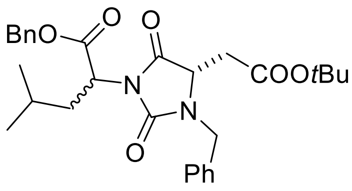

5h

^{1}H-NMR (400 MHz, CDCl$_3$)

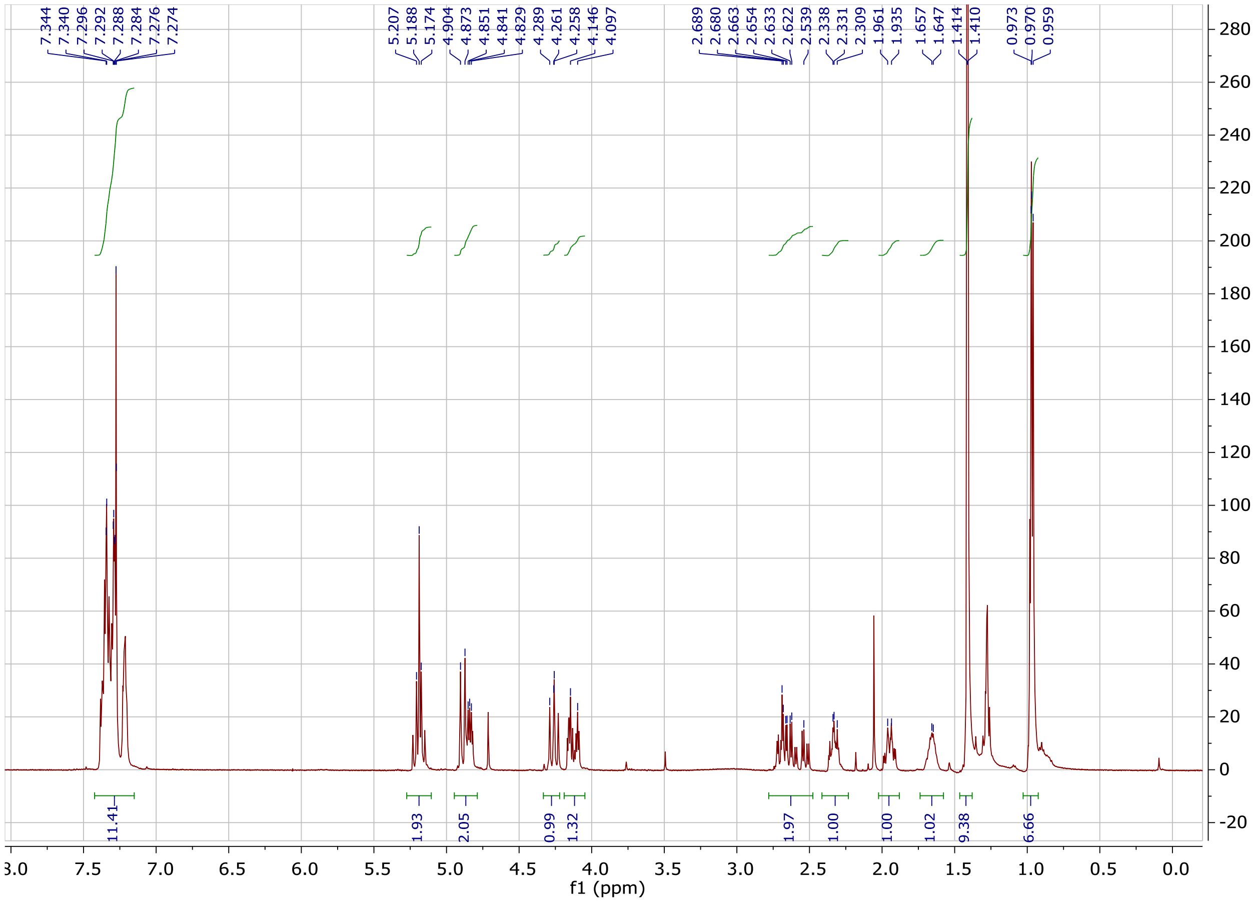

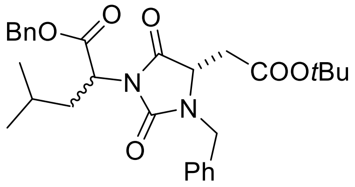

5h

$^{13}C\{^1H\}$-NMR (101 MHz, $CDCl_3$)

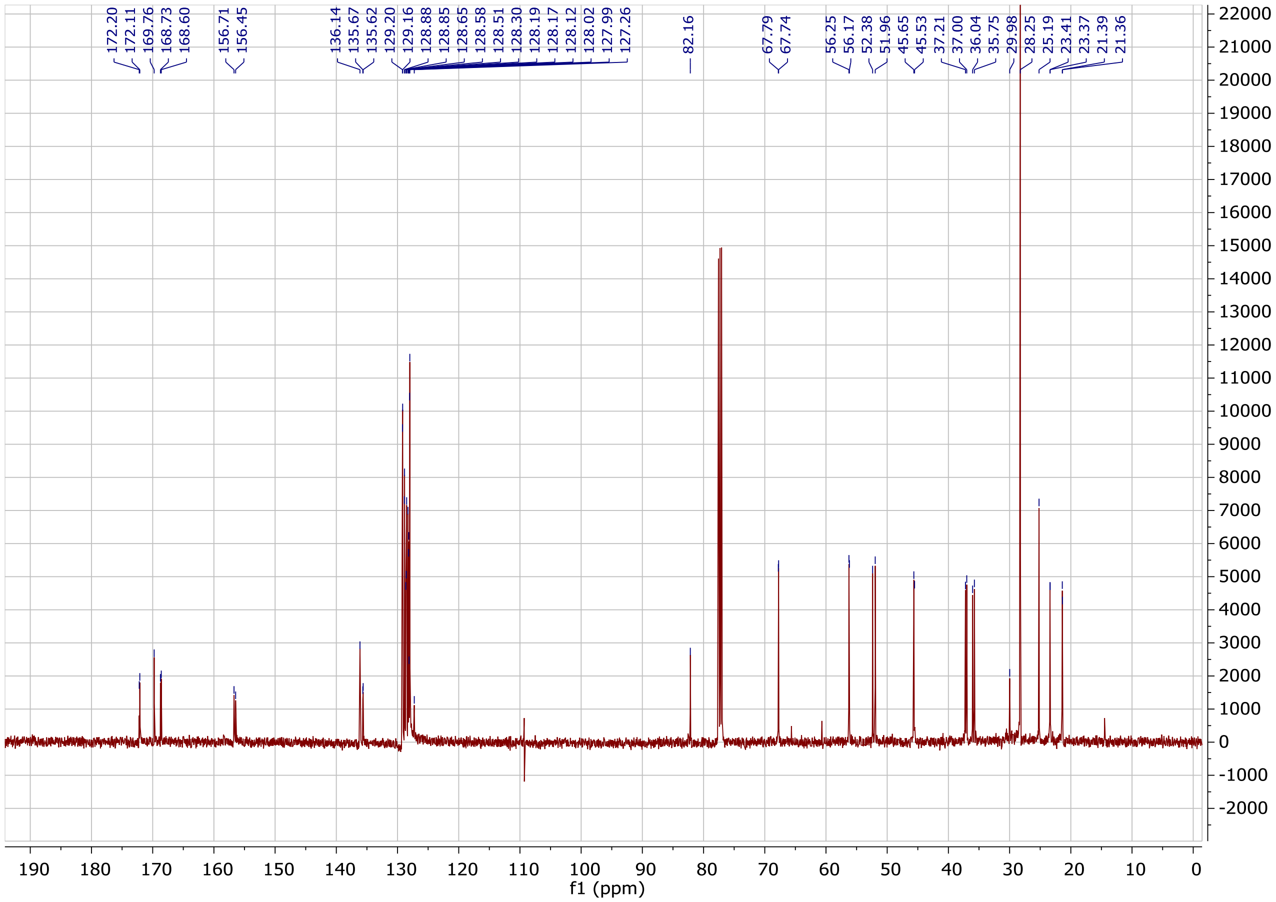

-L.G.S. - Laboratorio Grandi Strumenti - Display Report

Analysis Name	av gc91.d	Acquisition Date	10/26/18 15:28:52	Operator	Walter Panzeri
Sample Name		Method	Copy of _01tmix_posneg lm.MS	Instrument	esquire3000plus
Comment	1 mg/ml dil 1:100 MeOH<br>Richiedente: Cristina				

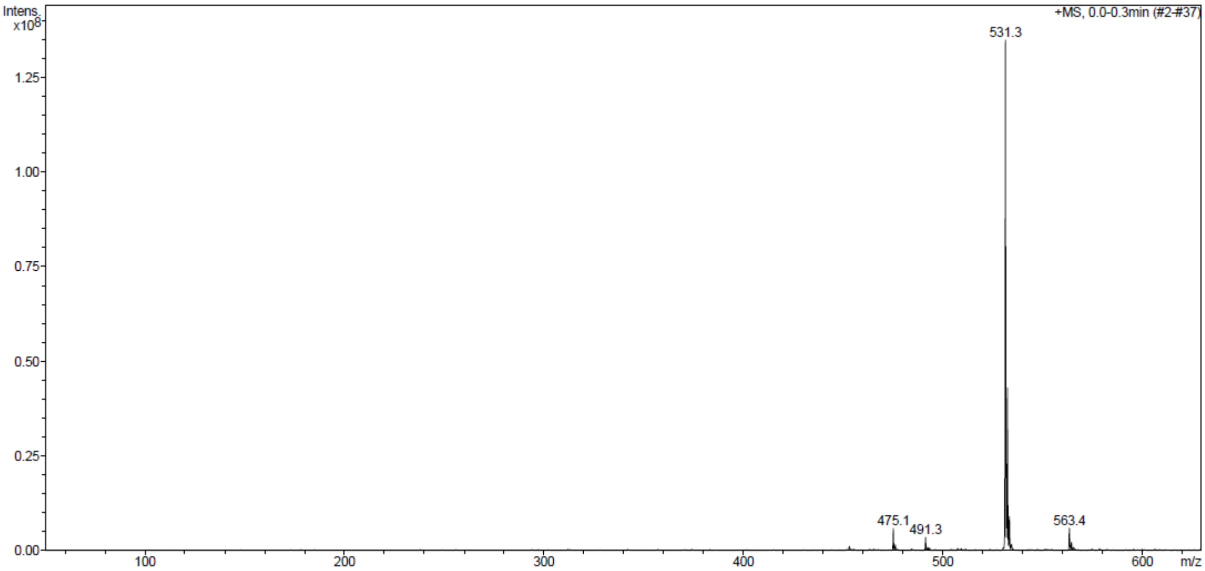

Bruker Daltonics DataAnalysis 3.1 printed: 10/26/18 15:34:14 Page 1 of 1

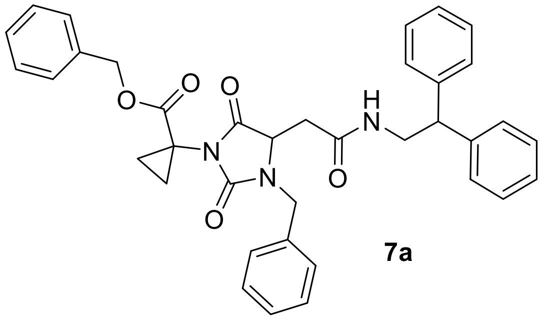

7a

^{1}H-NMR (400 MHz, $CDCl_3$)

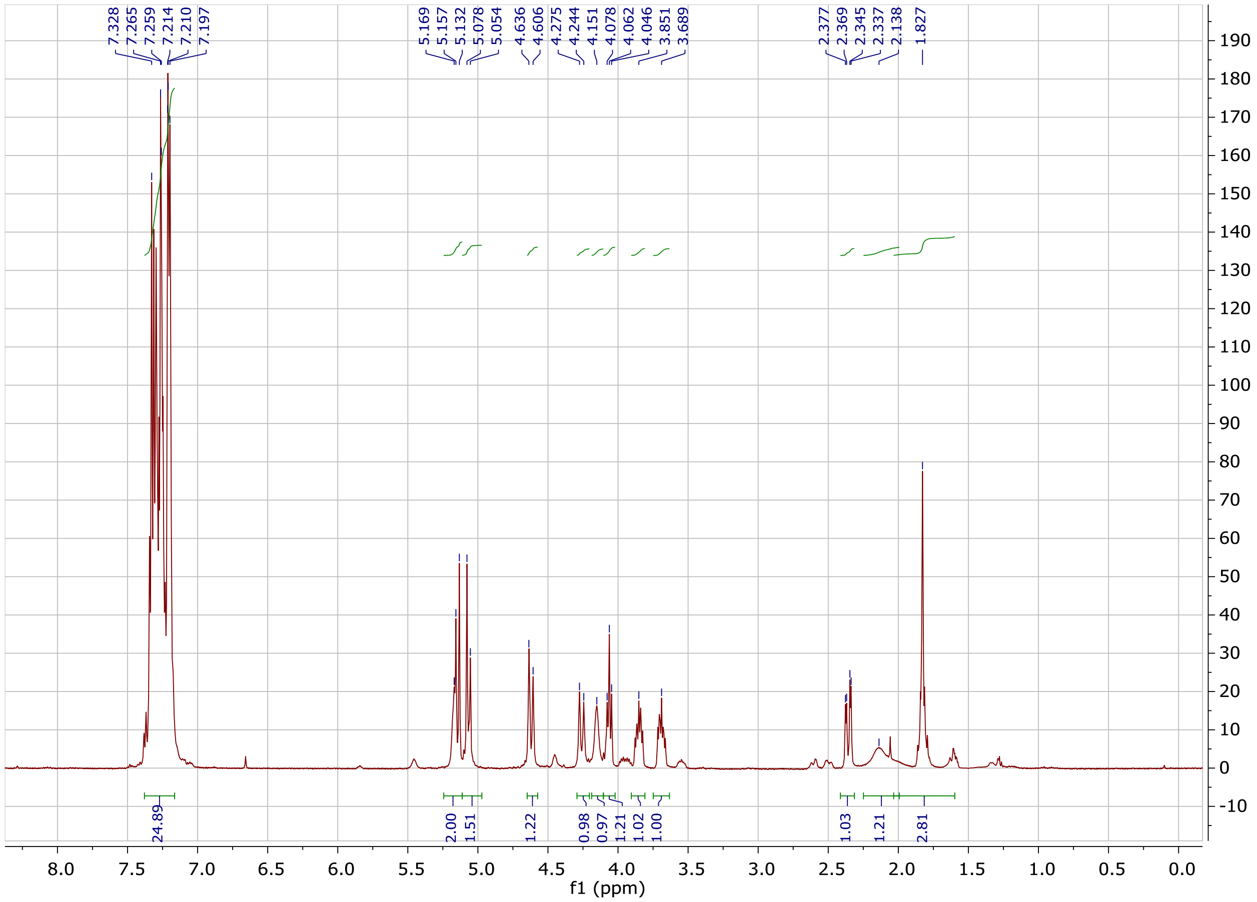

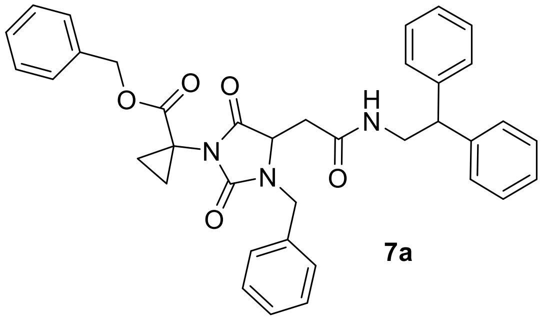

7a

$^{13}C\{^{1}H\}$-NMR (101 MHz, $CDCl_3$)

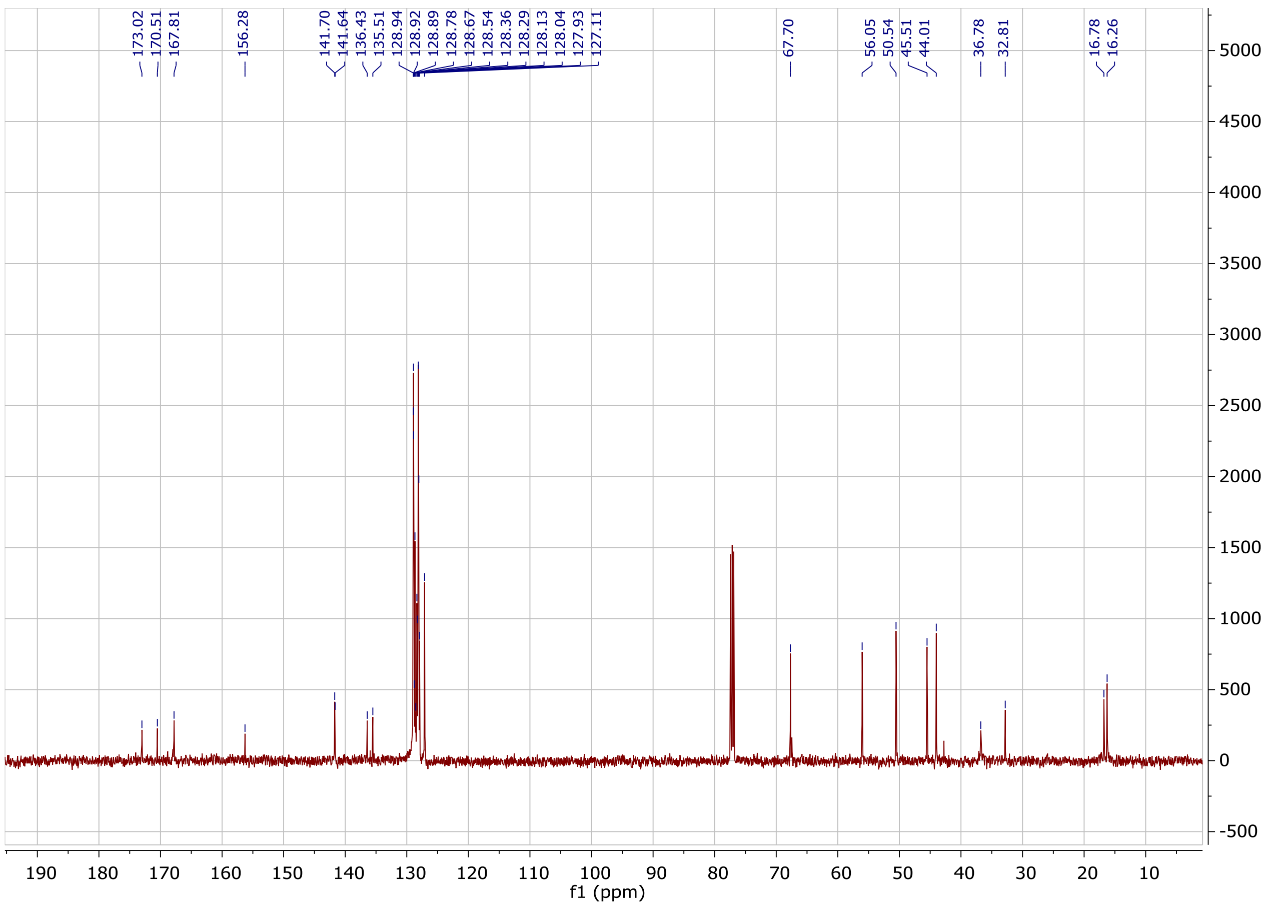

-L.G.S. - Laboratorio Grandi Strumenti - Display Report

Analysis Name	av fm2840.d	Acquisition Date	06/19/17 12:09:26	Operator	Administrator
Sample Name		Method	Copy of _01tmix_posneg lm.MS	Instrument	esquire3000plus
Comment	1 mg/ml dil 1:100 MeOH<br>Richiedente: Frigerio				

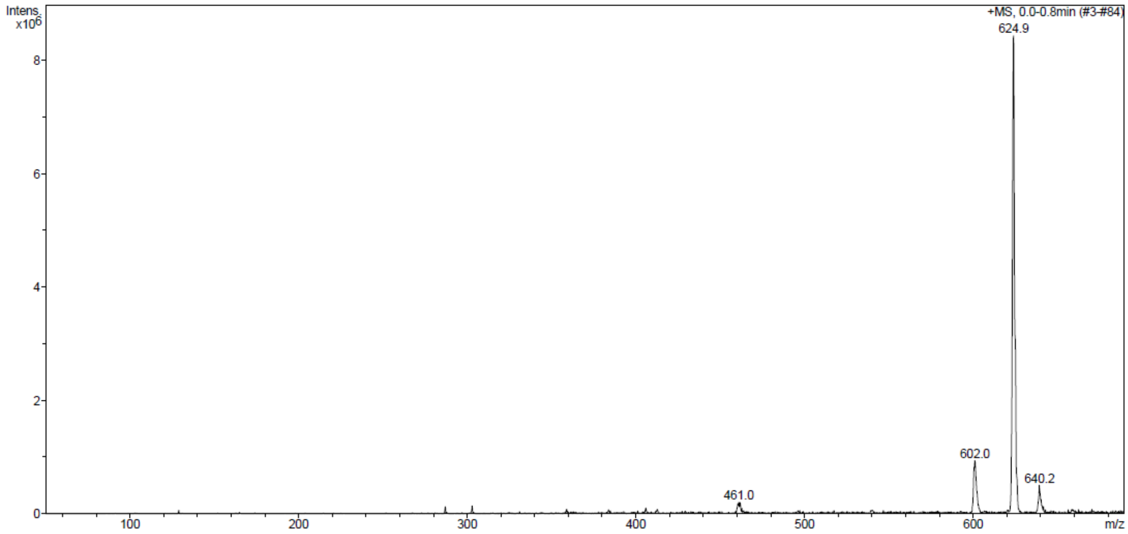

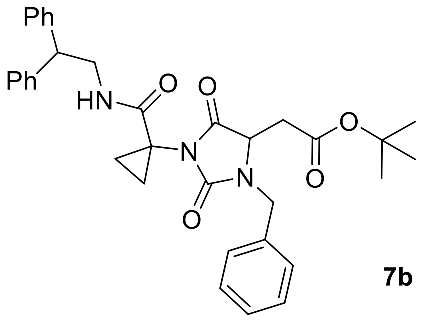

7b

^{1}H-NMR (400 MHz, $CDCl_3$)

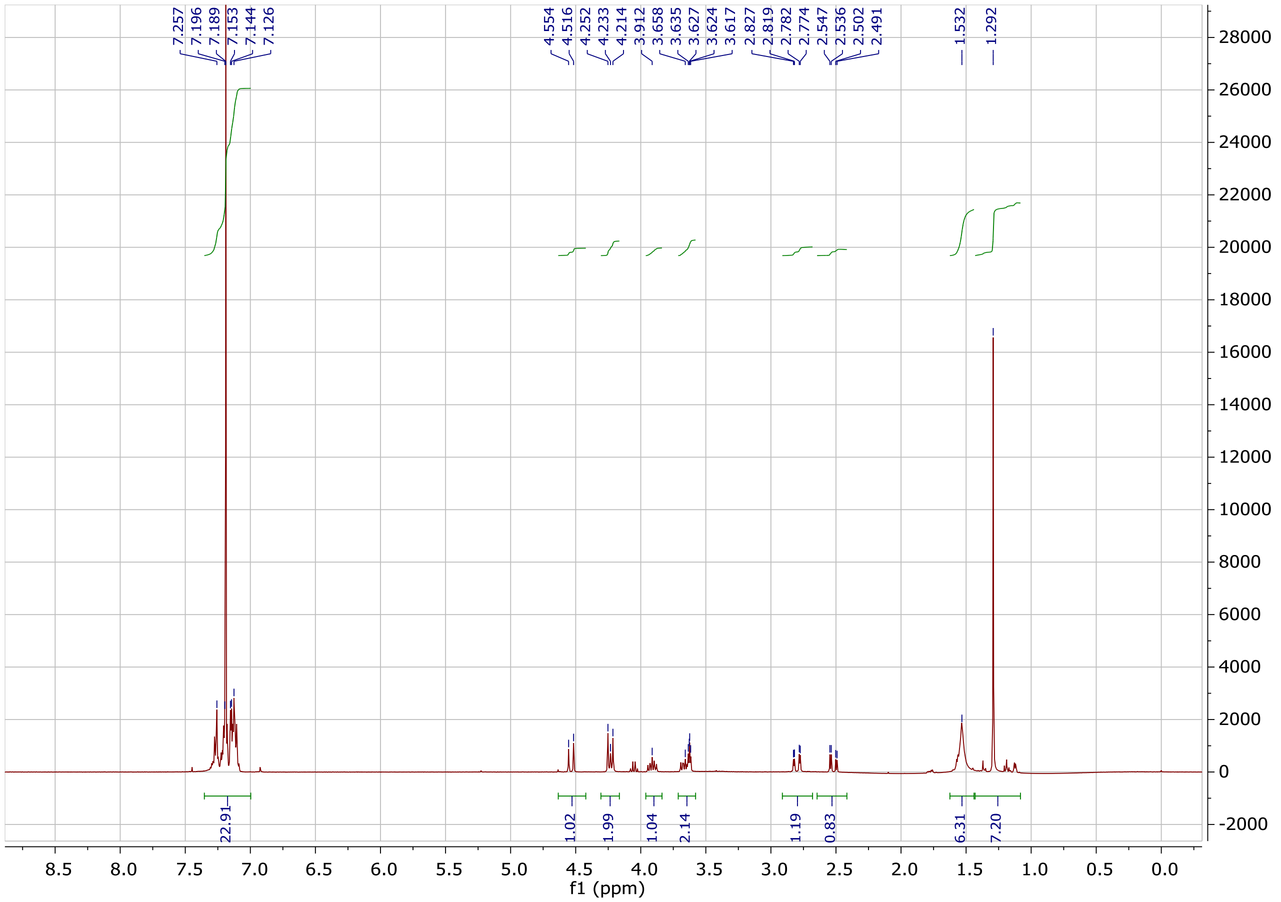

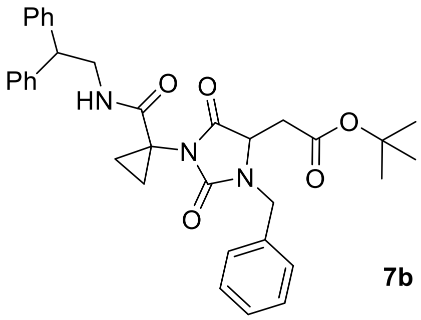

7b

$^{13}C\{^{1}H\}$-NMR (101 MHz, $CDCl_3$)

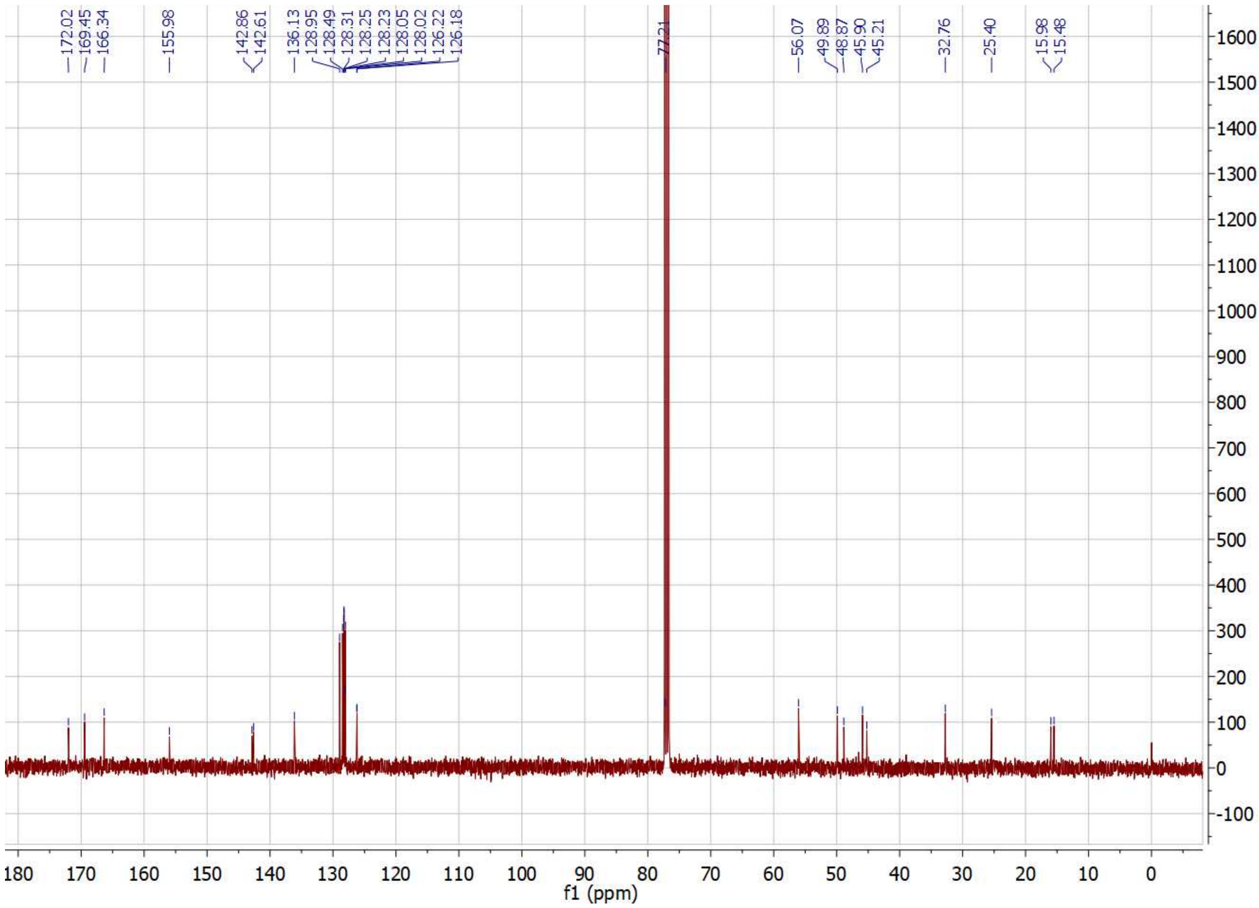

-L.G.S. - Laboratorio Grandi Strumenti - Display Report

Analysis Name	av gc132.d	Acquisition Date	07/01/19 14:41:30	Operator	Walter Panzeri
Sample Name		Method	Copy of _01tmix_posneg lm.MS	Instrument	esquire3000plus
Comment	1mg/ml dil 1:100 MeOH<br>Richiedente: Cristina				

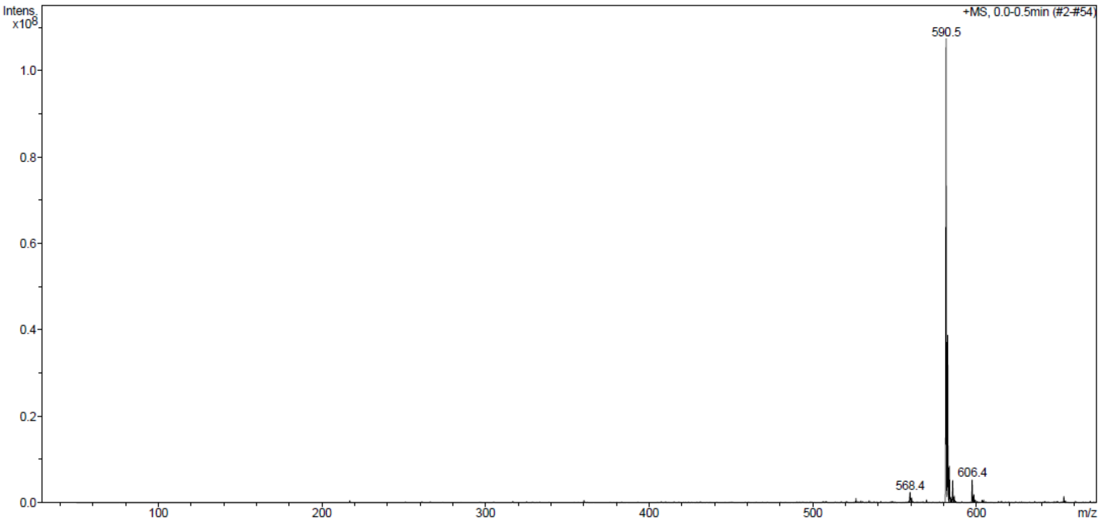

Bruker Daltonics DataAnalysis 3.1 printed: 07/01/19 14:44:00 Page 1 of 1

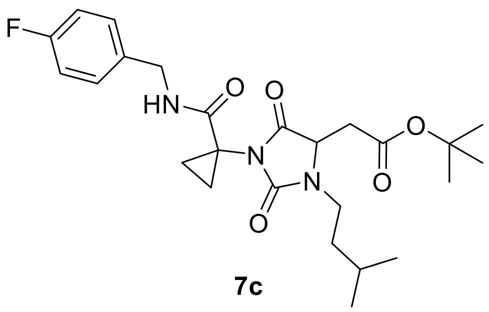

7c

^{1}H-NMR (400 MHz, $CDCl_3$)

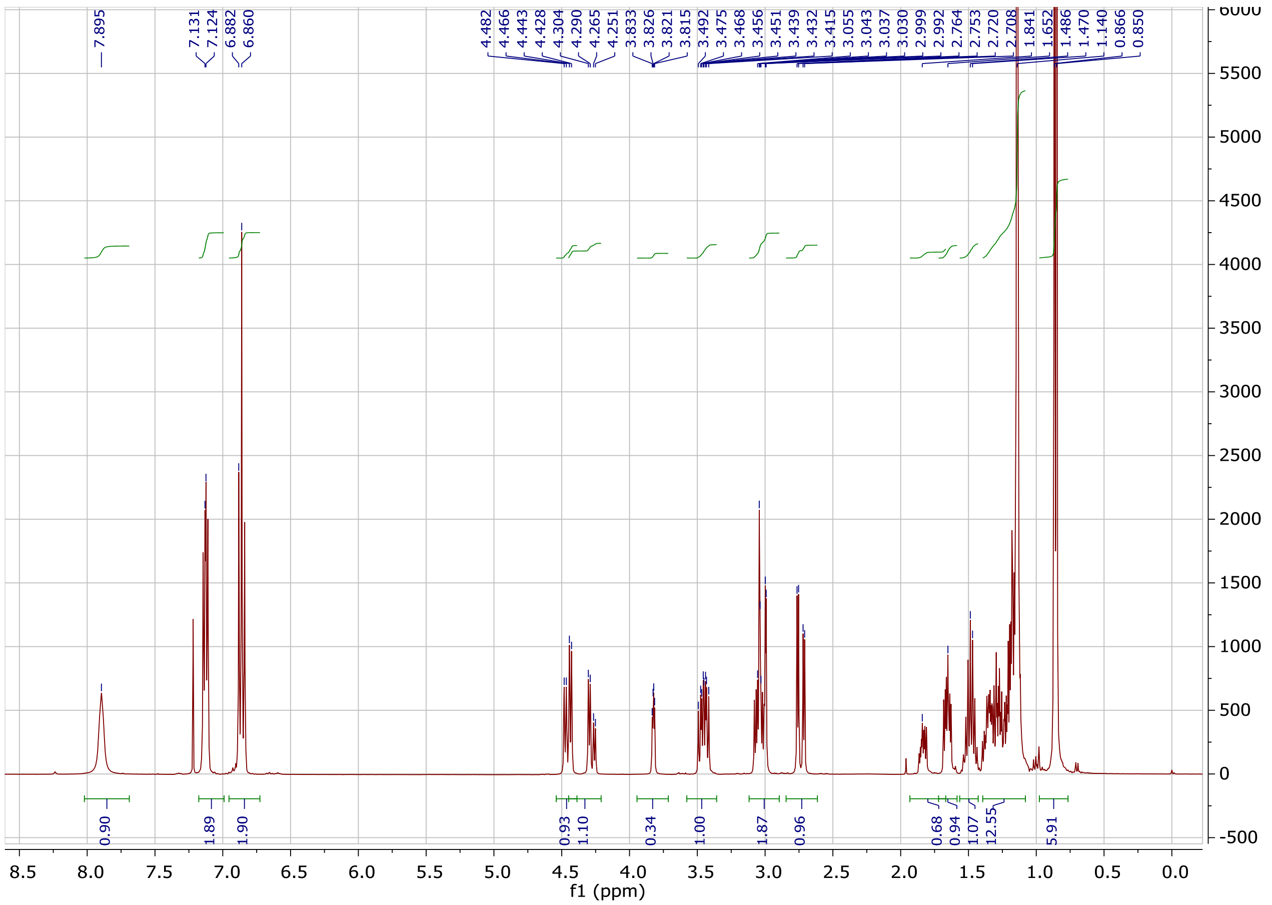

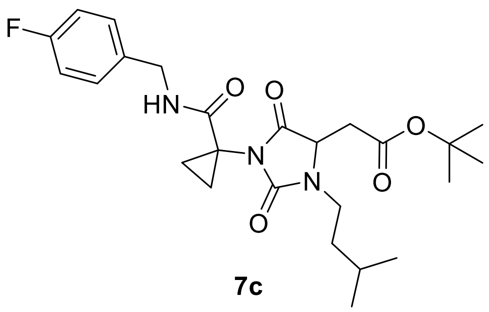

7c

$^{13}C\{^{1}H\}$-NMR (101 MHz, $CDCl_3$)

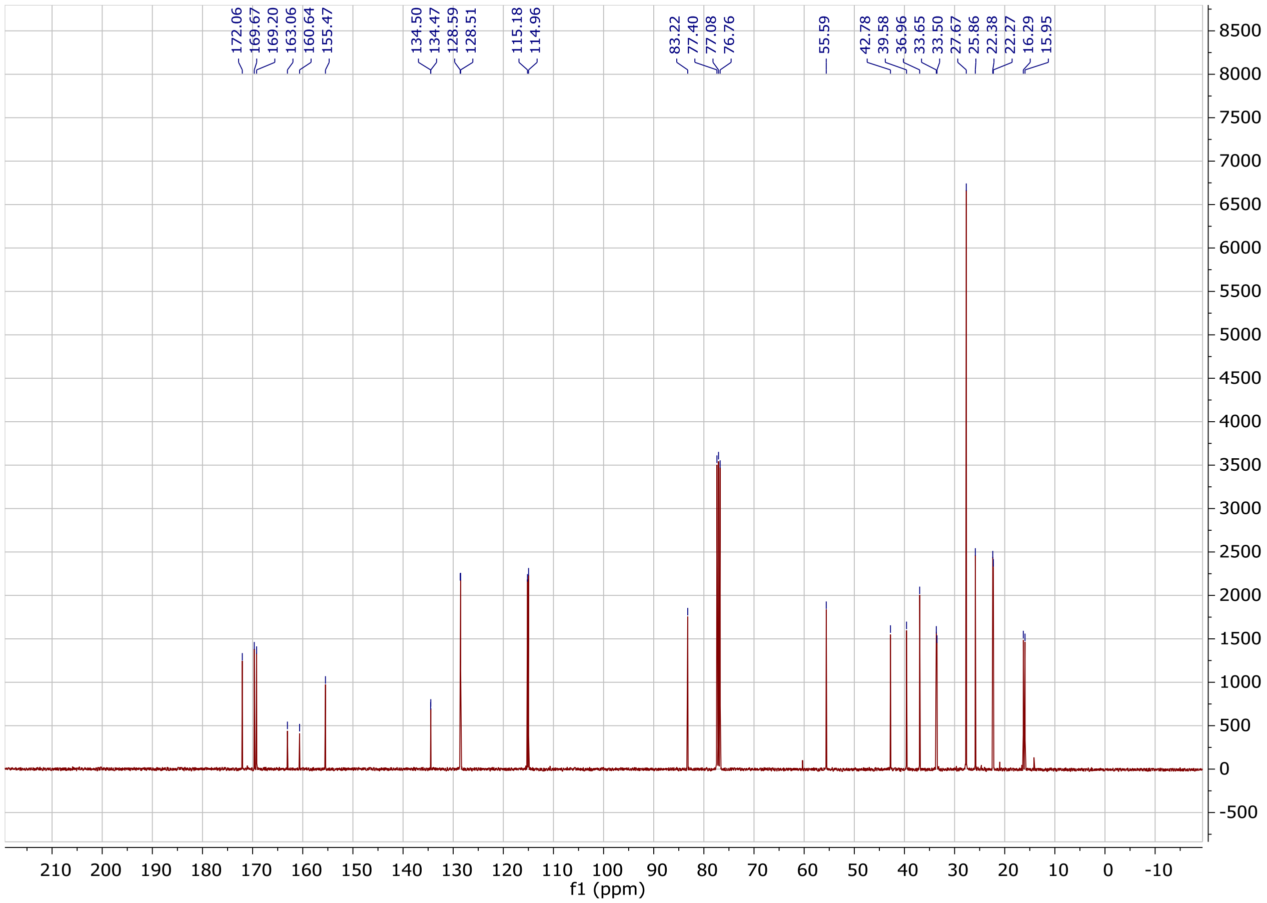

-L.G.S. - Laboratorio Grandi Strumenti - Display Report

Analysis Name	av gc120.d	Acquisition Date	04/05/19 13:14:49	Operator	Walter Panzeri
Sample Name		Method	Copy of _01tmix_posneg lm.MS	Instrument	esquire3000plus
Comment	1 mg/ml dil 1:100 MeOH Richiedente: Cristina				

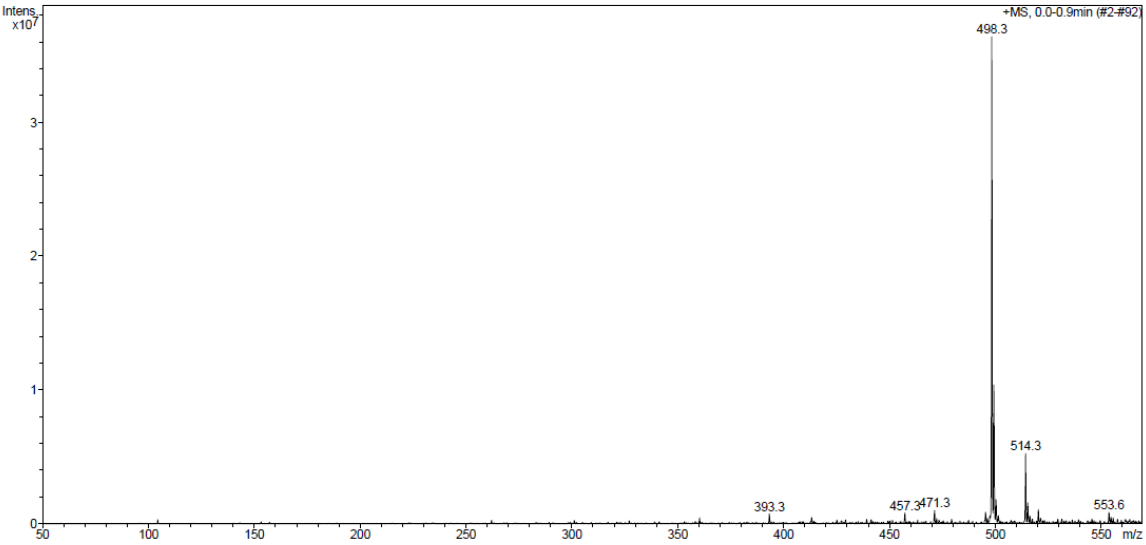

Bruker Daltonics DataAnalysis 3.1 printed: 04/05/19 13:21:00 Page 1 of 1

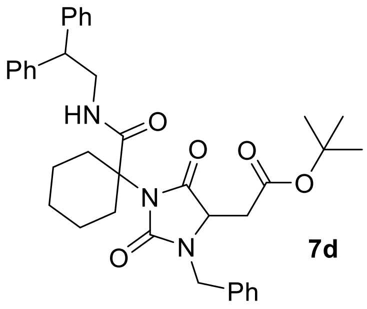

7d

^{1}H-NMR (400 MHz, $CDCl_3$)

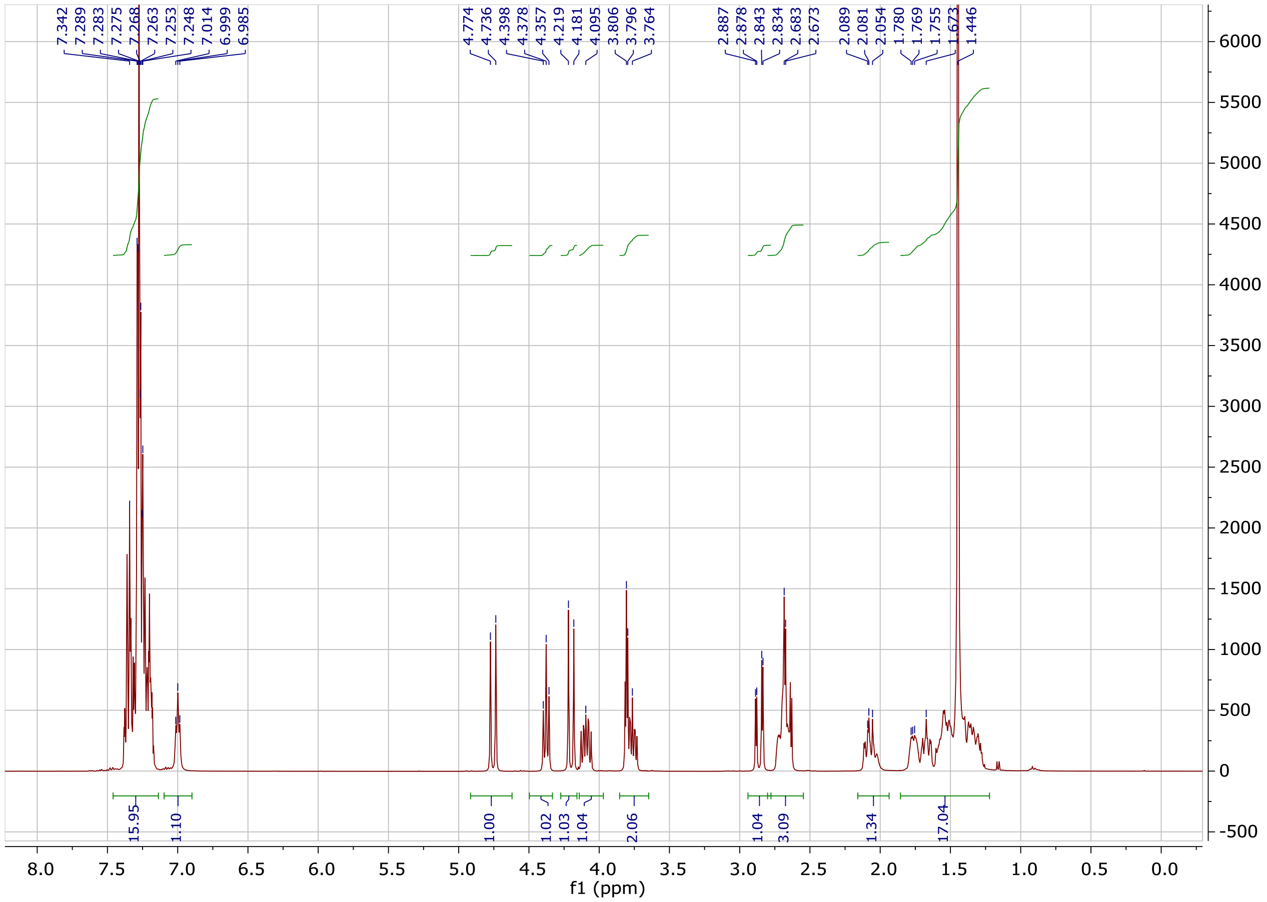

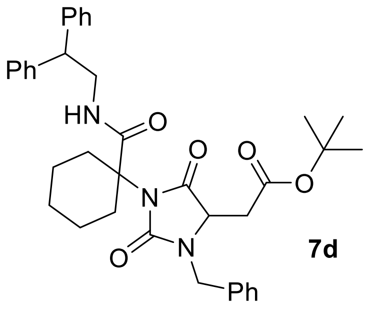

7d

$^{13}C\{^1H\}$-NMR (101 MHz, $CDCl_3$)

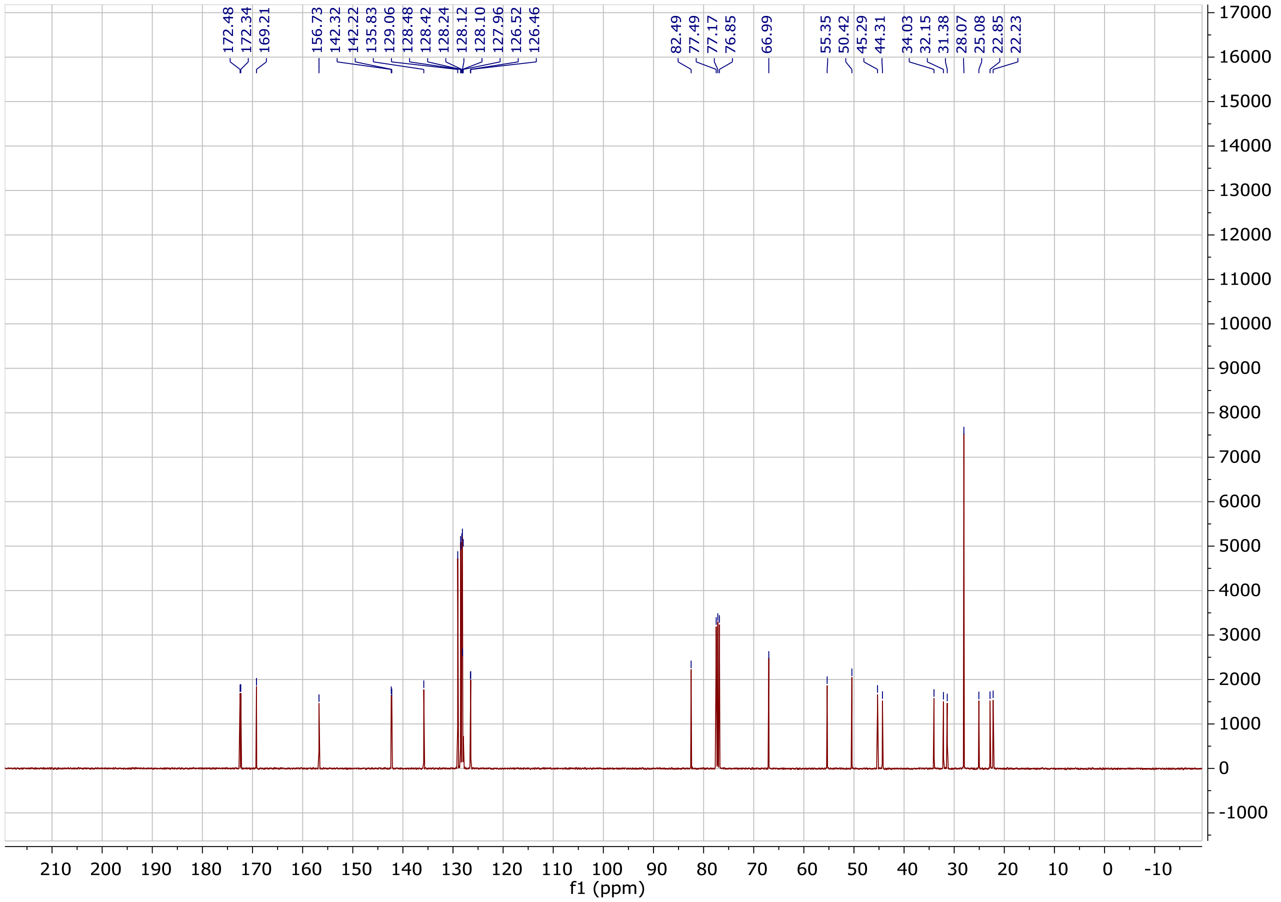

-L.G.S. - Laboratorio Grandi Strumenti - Display Report

Analysis Name	av gc78.d	Acquisition Date	07/25/18 15:35:31	Operator	Walter Panzeri
Sample Name		Method	Copy of _01tmix_posneg lm.MS	Instrument	esquire3000plus
Comment	1 mg/ml dil 1:100 MeOH<br>Richiedente: Cristina				

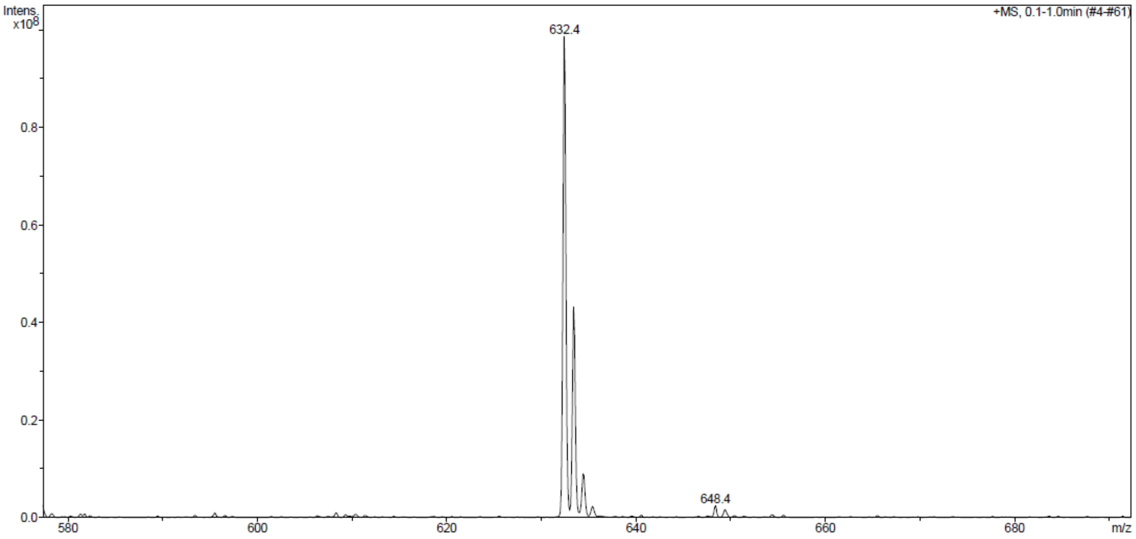

Bruker Daltonics DataAnalysis 3.1 printed: 07/25/18 15:37:11 Page 1 of 1

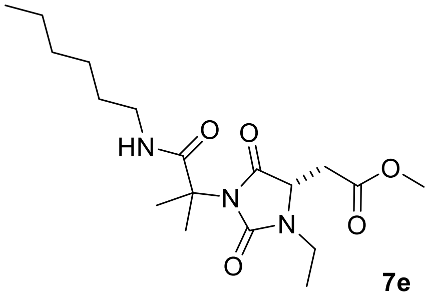

7e

^{1}H-NMR (400 MHz, $CDCl_3$)

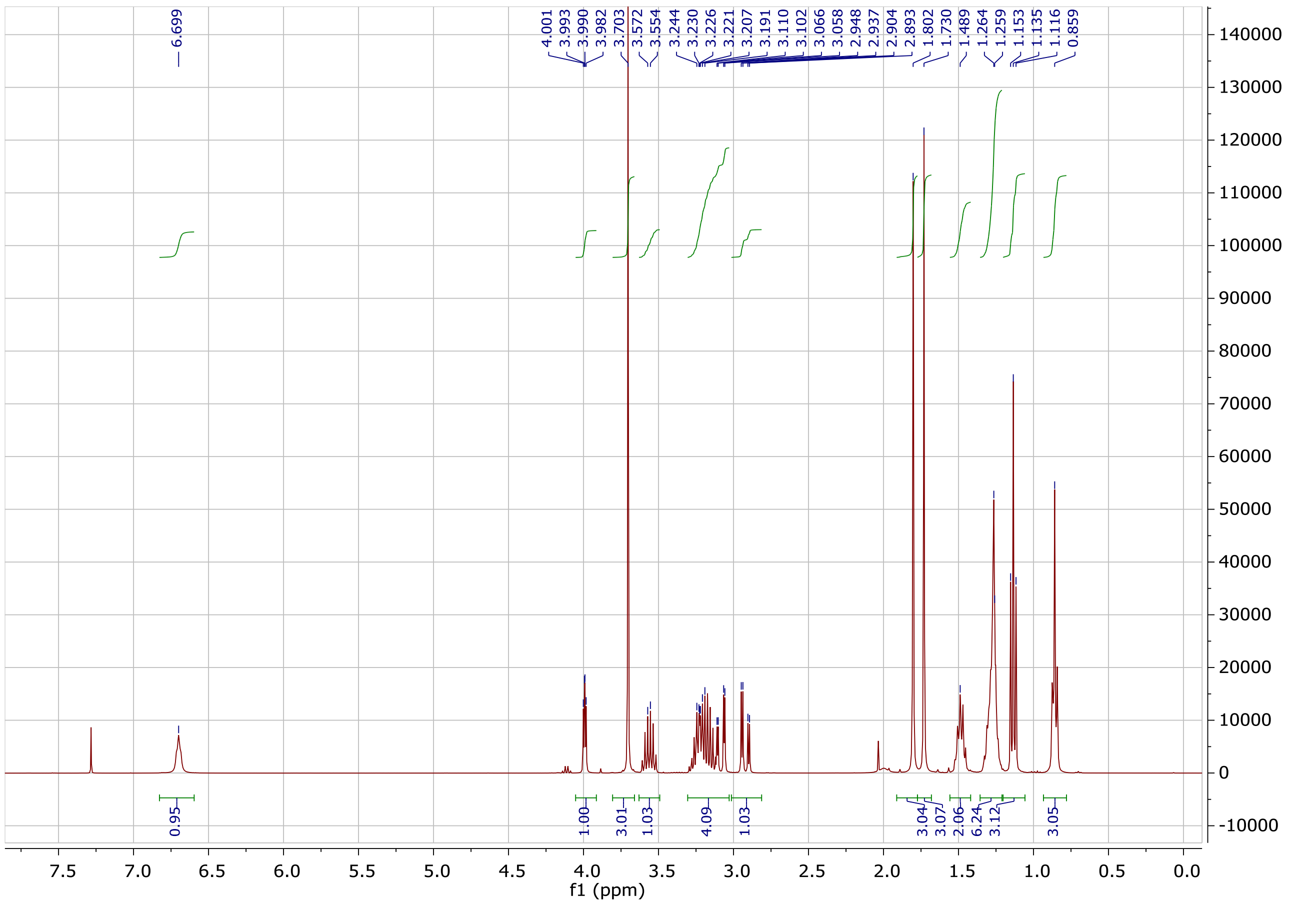

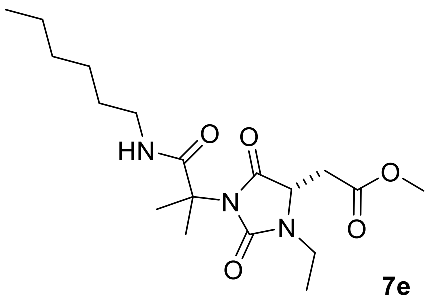

7e

$^{13}C\{^{1}H\}$-NMR (101 MHz, $CDCl_3$)

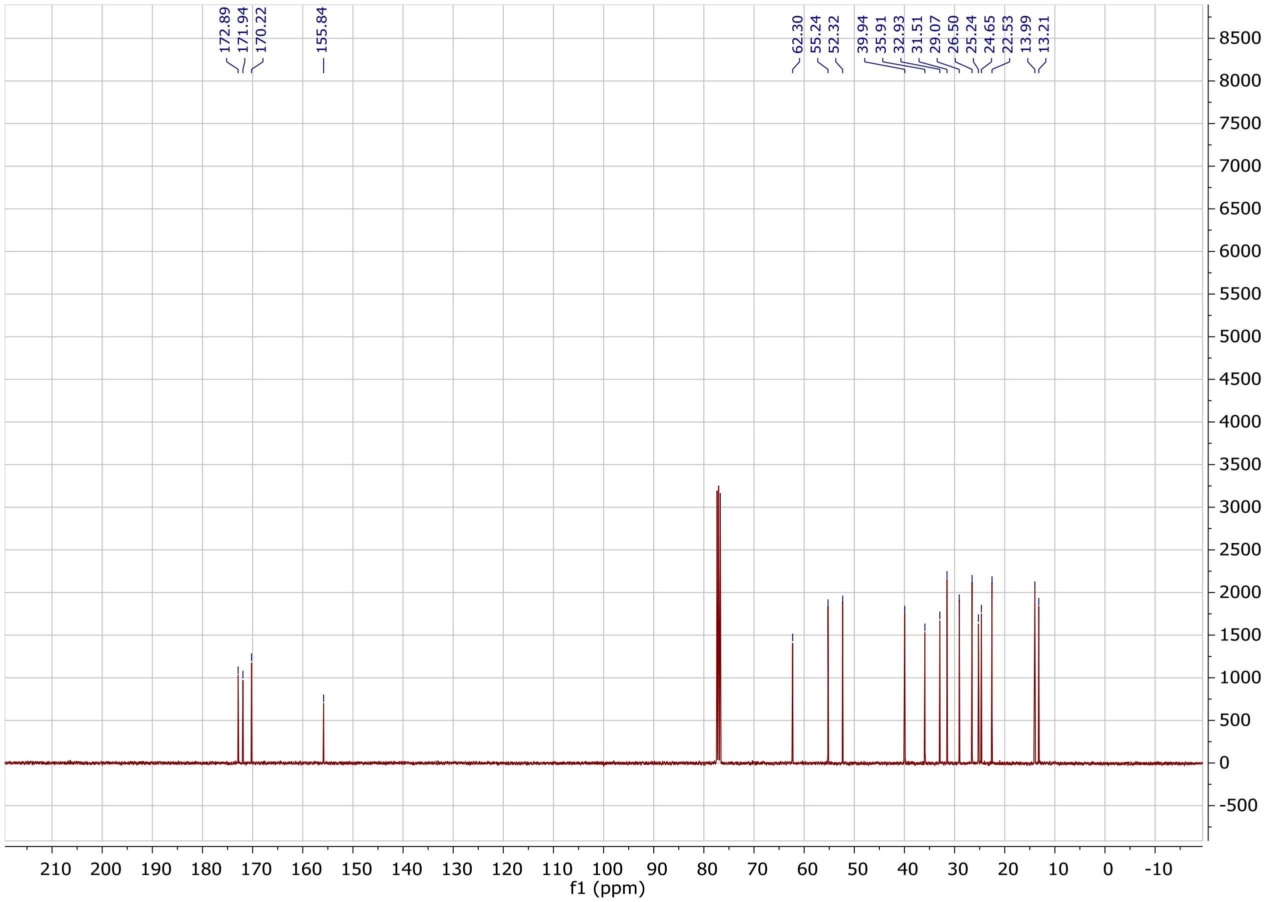

-L.G.S. - Laboratorio Grandi Strumenti - Display Report

Analysis Name	av gc20_c.d	Acquisition Date	01/26/18 14:43:10	Operator	Walter Panzeri
Sample Name		Method	Copy of _01tmix_posneg lm.MS	Instrument	esquire3000plus
Comment	1 mg/ml H2O<br>Richiedente: Cristina				

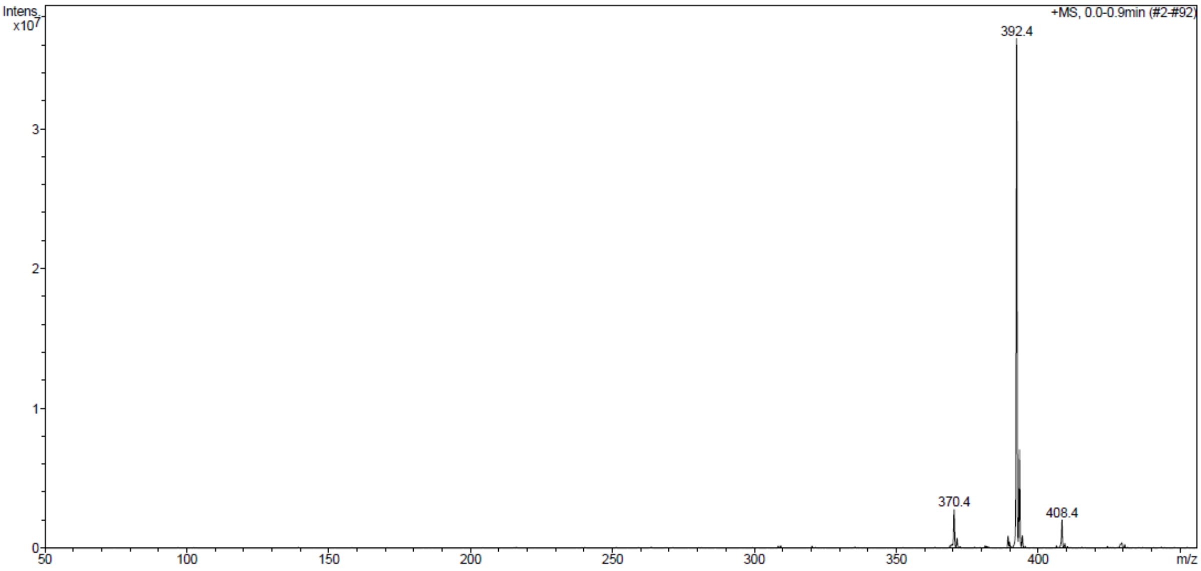

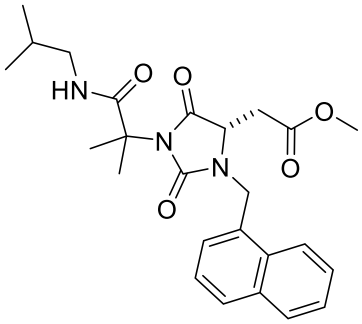

7f

^{1}H-NMR (400 MHz, $CDCl_3$)

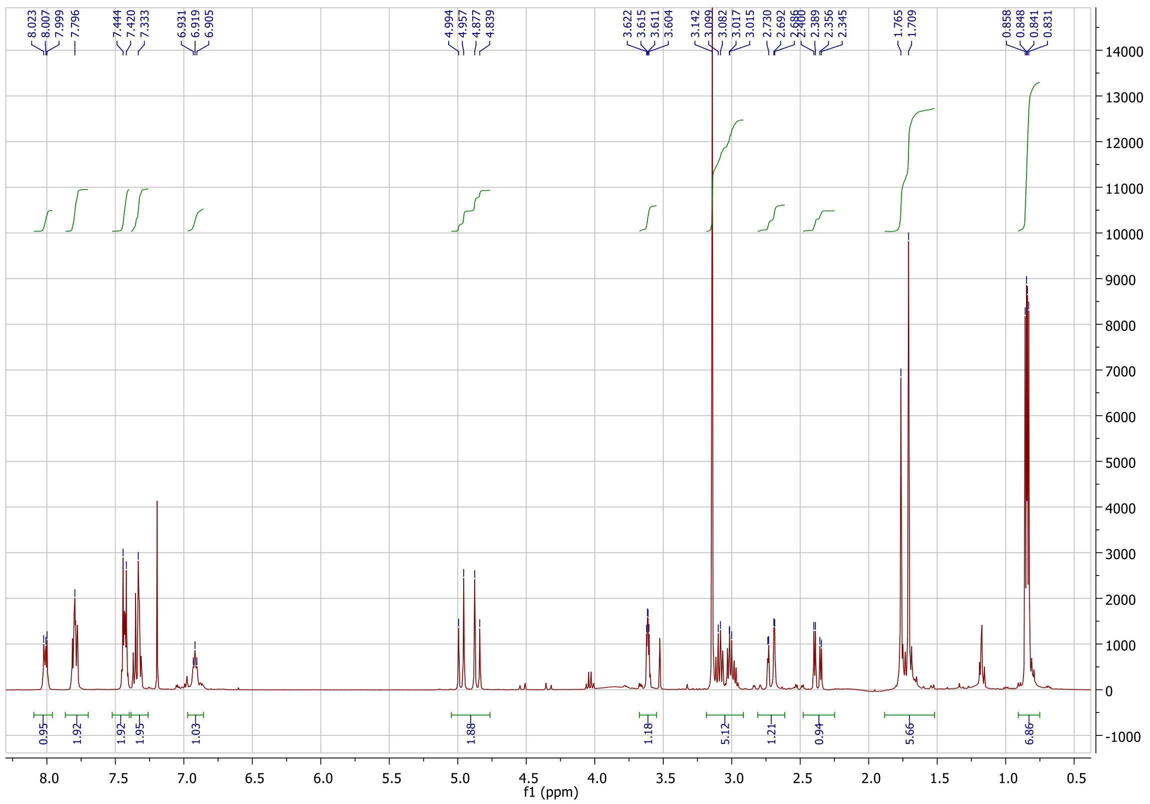

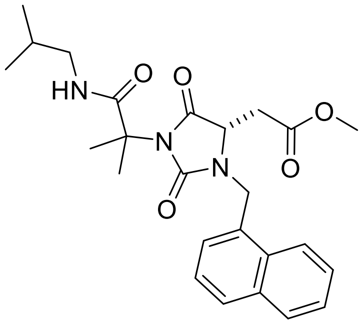

7f

$^{13}C\{^{1}H\}$-NMR (101 MHz, $CDCl_3$)

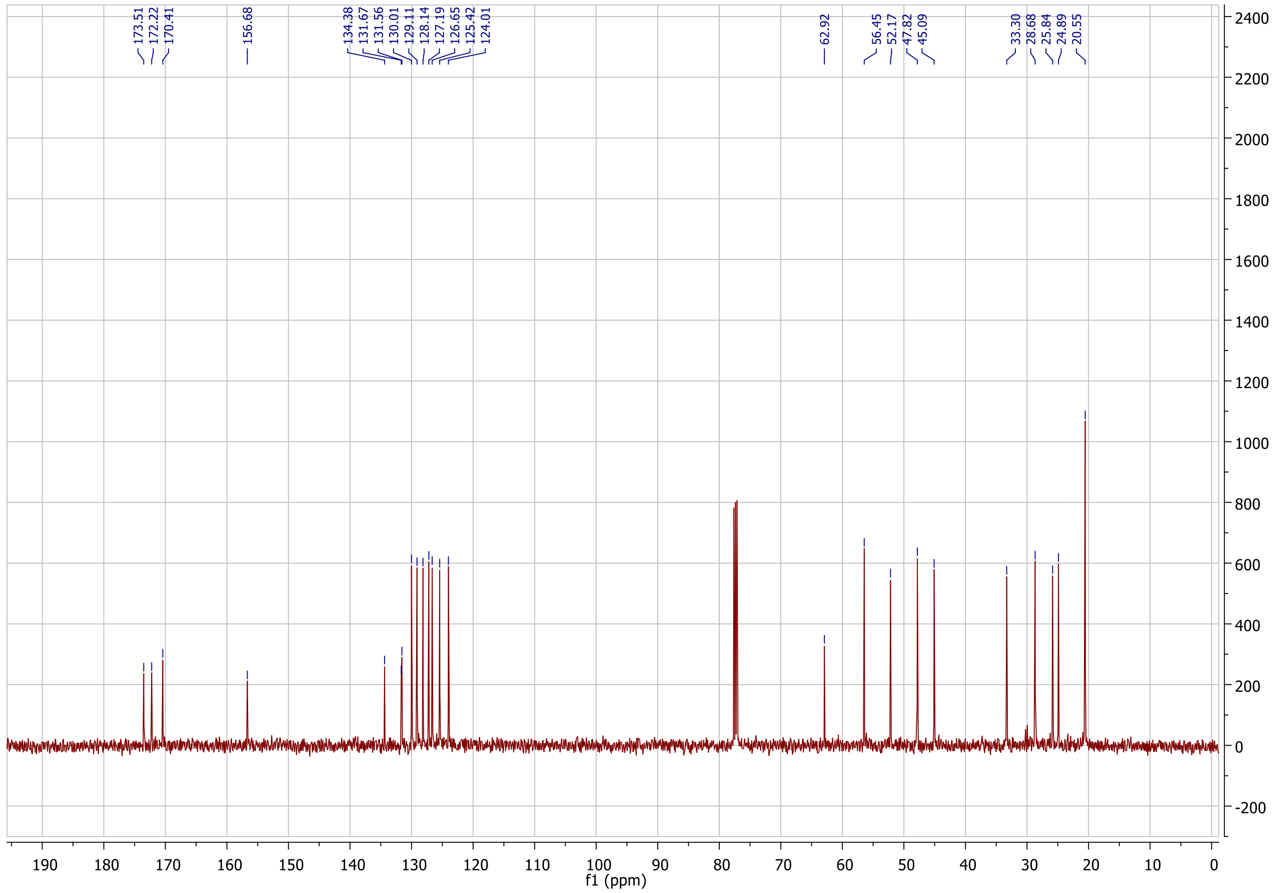

-L.G.S. - Laboratorio Grandi Strumenti - Display Report

Analysis Name	av gc50.d	Acquisition Date	05/22/18 12:26:44	Operator	Walter Panzeri
Sample Name		Method	Copy of _01tmix_posneg lm.MS	Instrument	esquire3000plus
Comment	1 mg/ml dil 1:100 MeOH<br>Richiedente: Cristina				

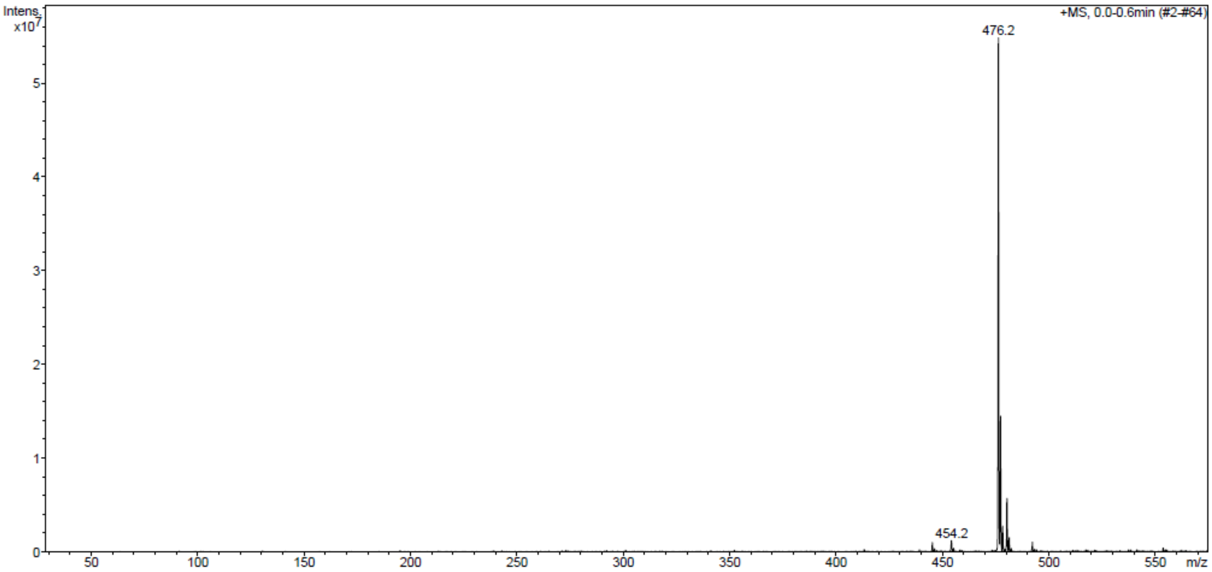

Bruker Daltonics DataAnalysis 3.1 printed: 05/22/18 12:28:40 Page 1 of 1

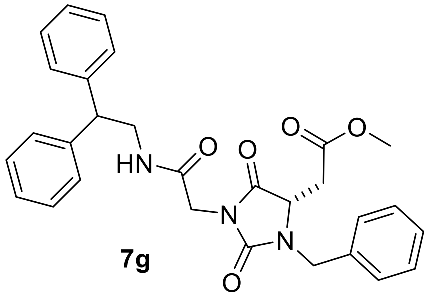

7g

^{1}H-NMR (400 MHz, $CDCl_3$)

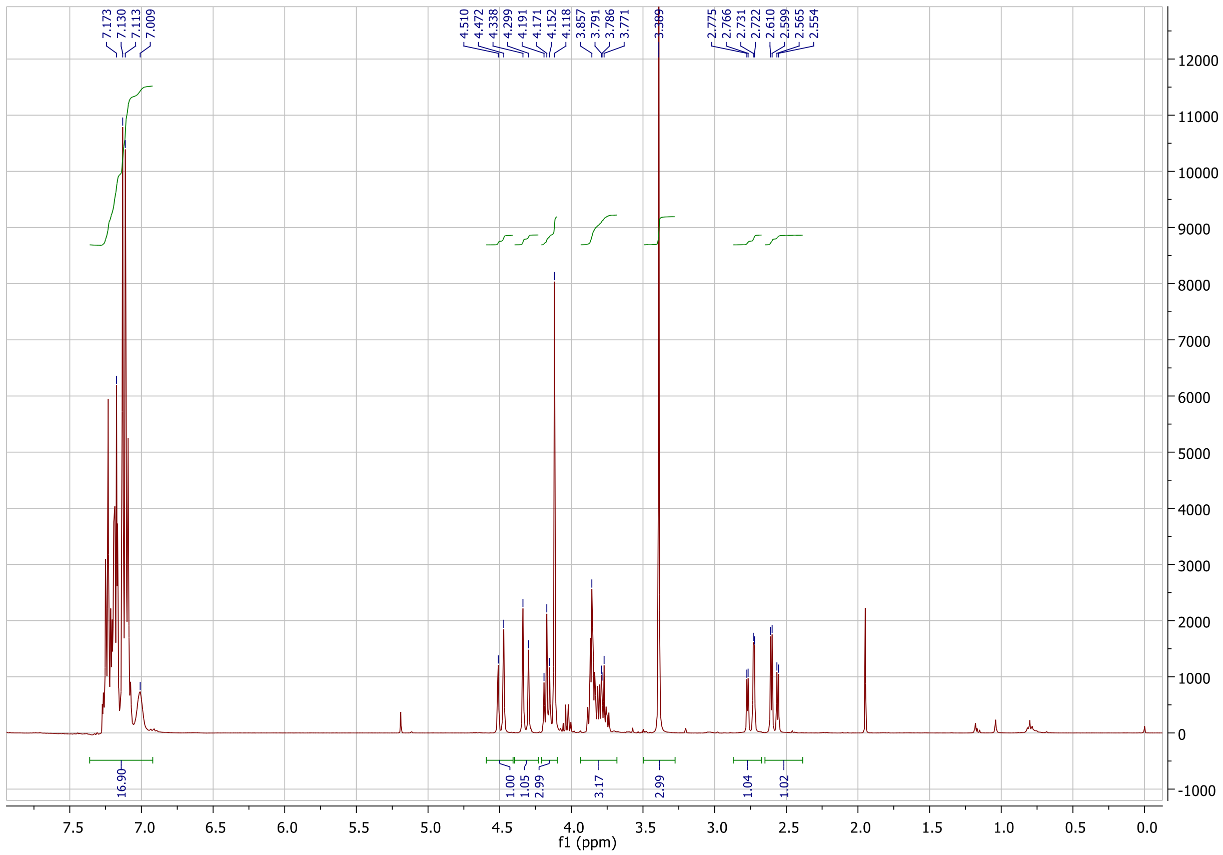

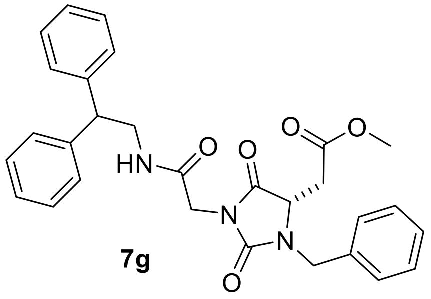

7g

$^{13}C\{^1H\}$-NMR (101 MHz, $CDCl_3$)

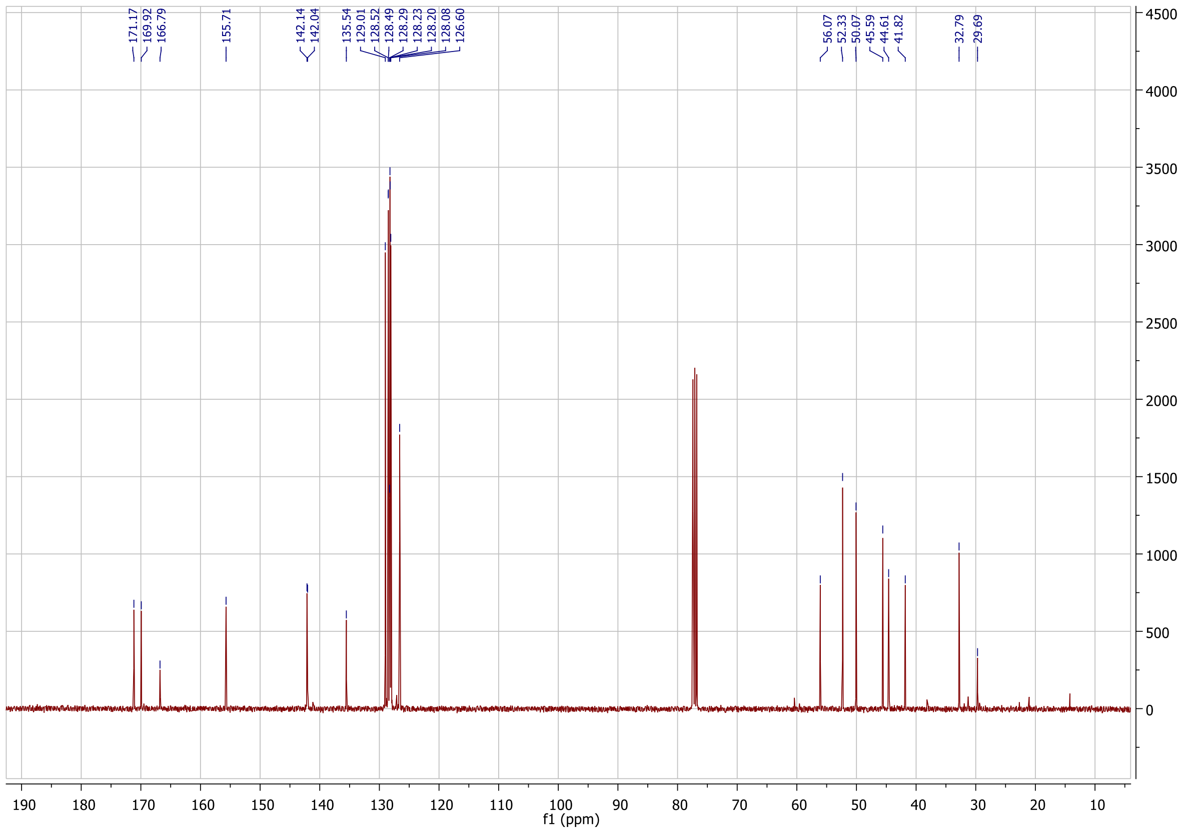

-L.G.S. - Laboratorio Grandi Strumenti - Display Report

Analysis Name	av gc38.d	Acquisition Date	04/16/18 09:30:16	Operator	Walter Panzeri
Sample Name		Method	Copy of _01tmix_posneg lm.MS	Instrument	esquire3000plus
Comment	1 mg/ml dil 1:100 MeOH Richiedente: Cristina				

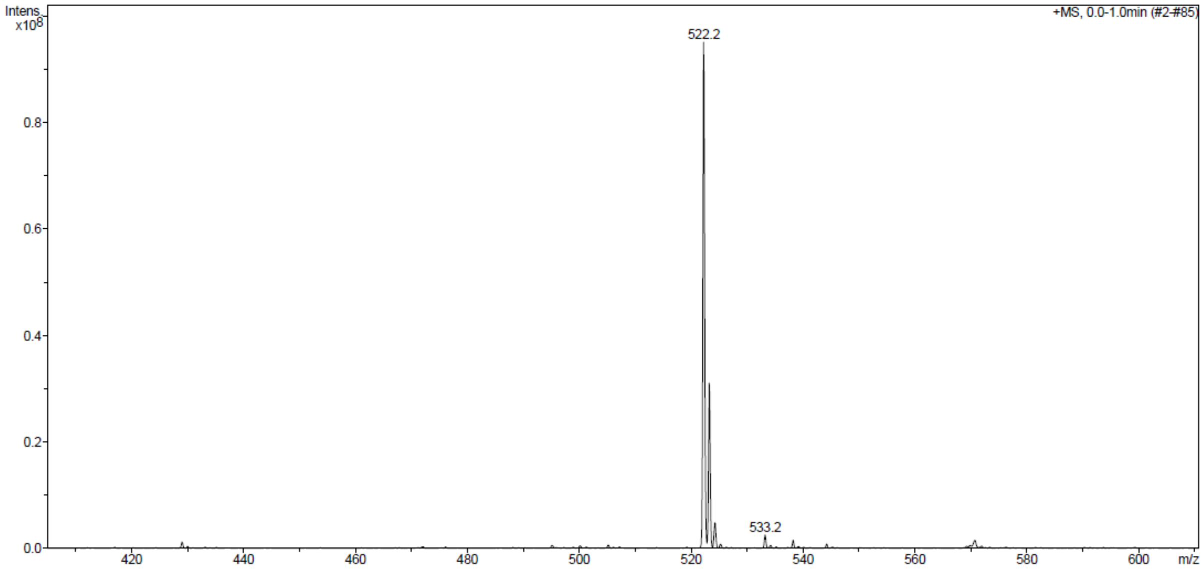

Bruker Daltonics DataAnalysis 3.1 printed: 04/16/18 09:32:00 Page 1 of 1

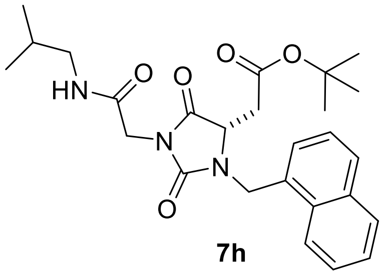

7h

^{1}H-NMR (400 MHz, CDCl$_3$)

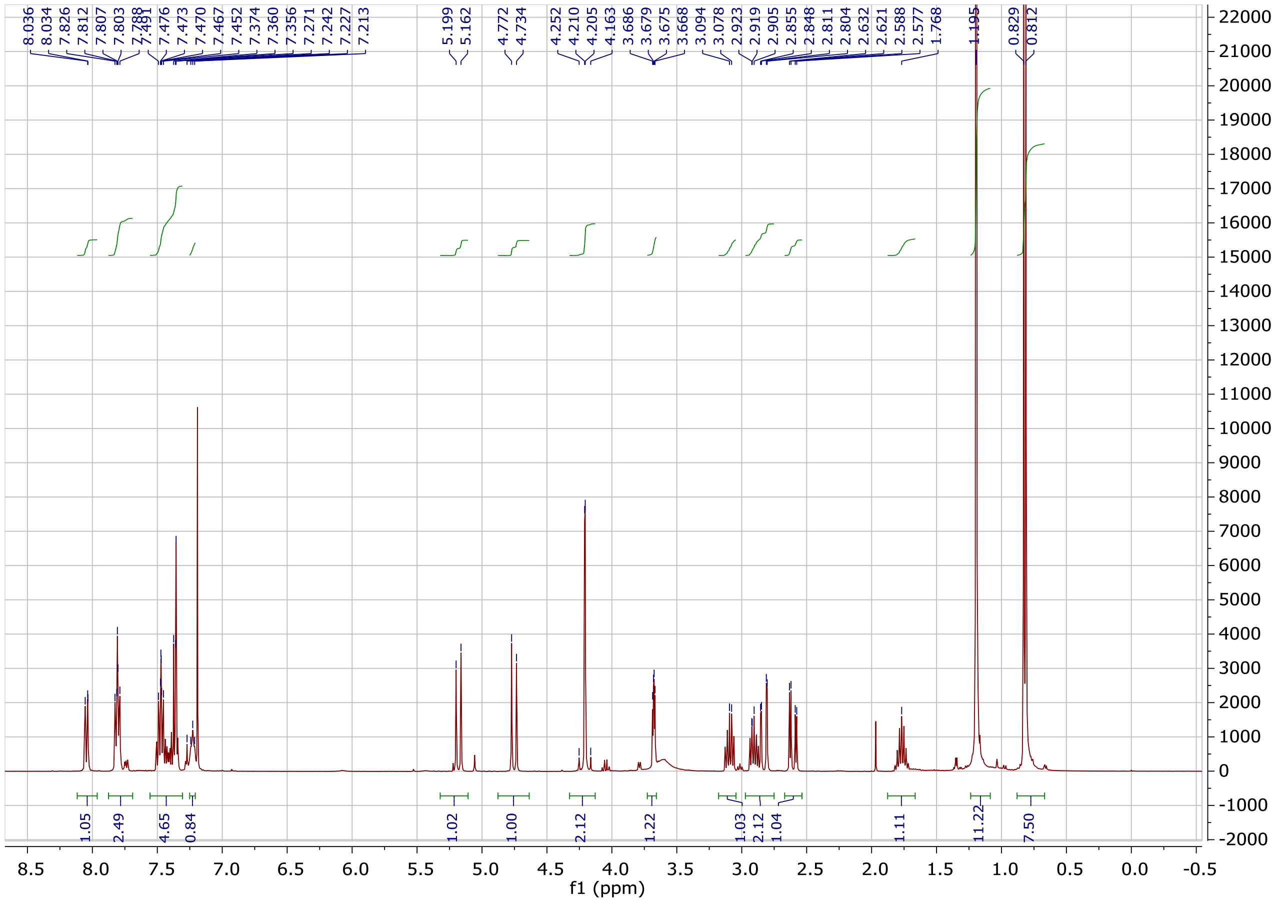

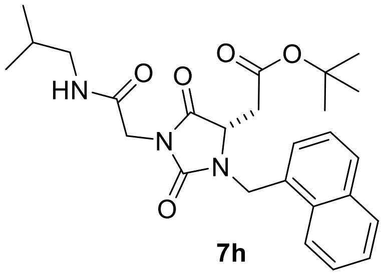

7h

$^{13}C\{^1H\}$-NMR (101 MHz, $CDCl_3$)

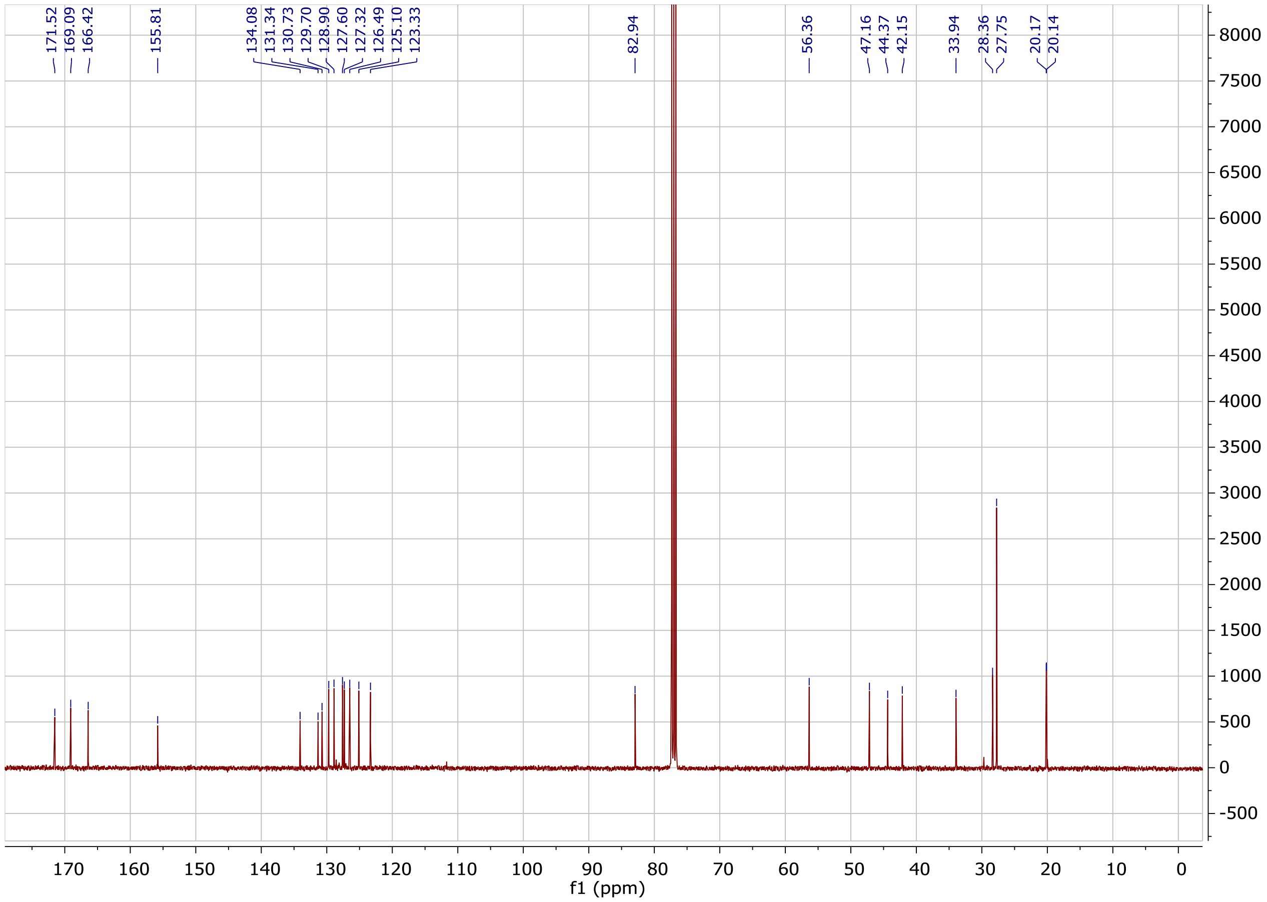

-L.G.S. - Laboratorio Grandi Strumenti - Display Report

Analysis Name	av ac069.d	Acquisition Date	08/31/20 13:39:32	Operator	Walter Panzeri
Sample Name		Method	Copy of $PW_TBLM.MS	Instrument	esquire3000plus
Comment	1 mg/ml dil 1:100 MeOH<br>Richiedente: Caramiello				

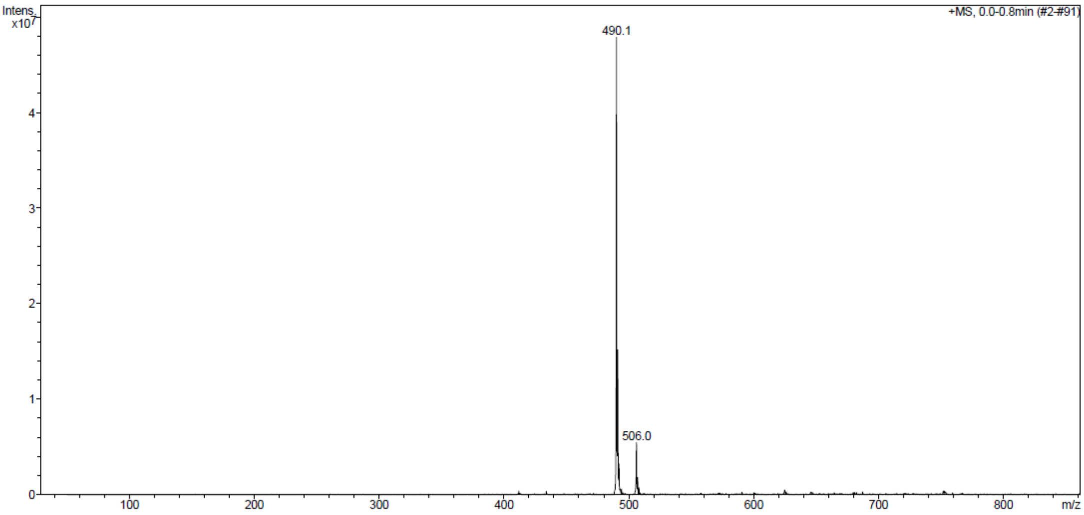

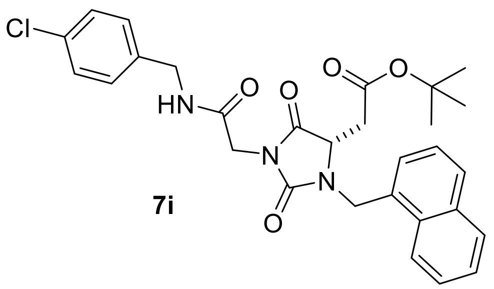

7i

^{1}H-NMR (400 MHz, $CDCl_3$)

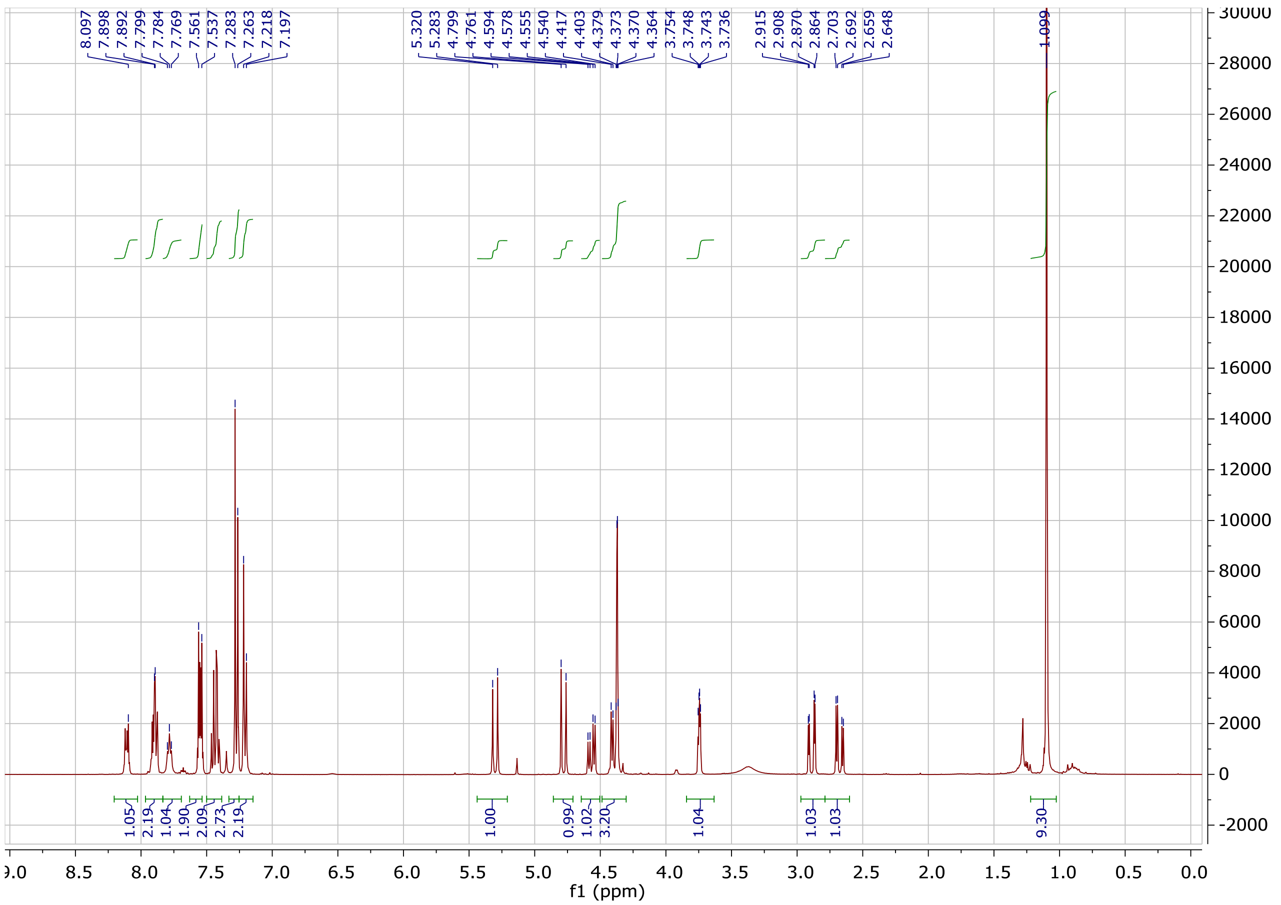

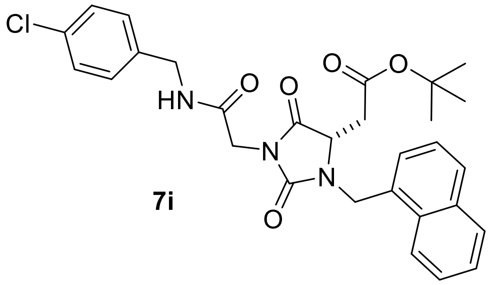

7i

$^{13}C\{^1H\}$-NMR (101 MHz, $CDCl_3$)

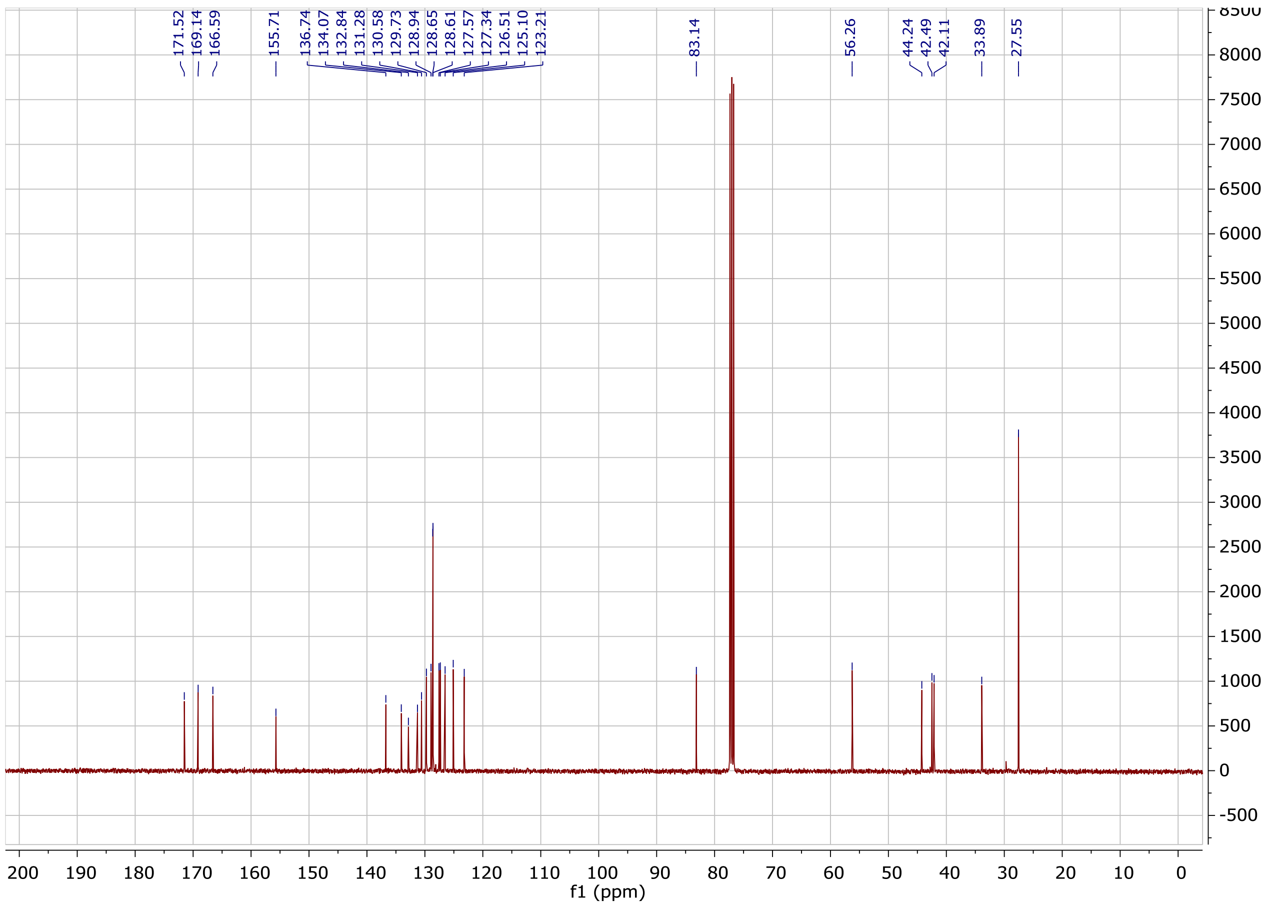

-L.G.S. - Laboratorio Grandi Strumenti - Display Report

Analysis Name	av ac194.d	Acquisition Date	03/18/22 11:26:34	Operator	Walter Panzeri
Sample Name		Method	Copy of $opt0622.MS	Instrument	esquire3000plus
Comment	1 mg/ml dil 1:100 MeOH<br>Richiedente: Mapanesi Alessia				

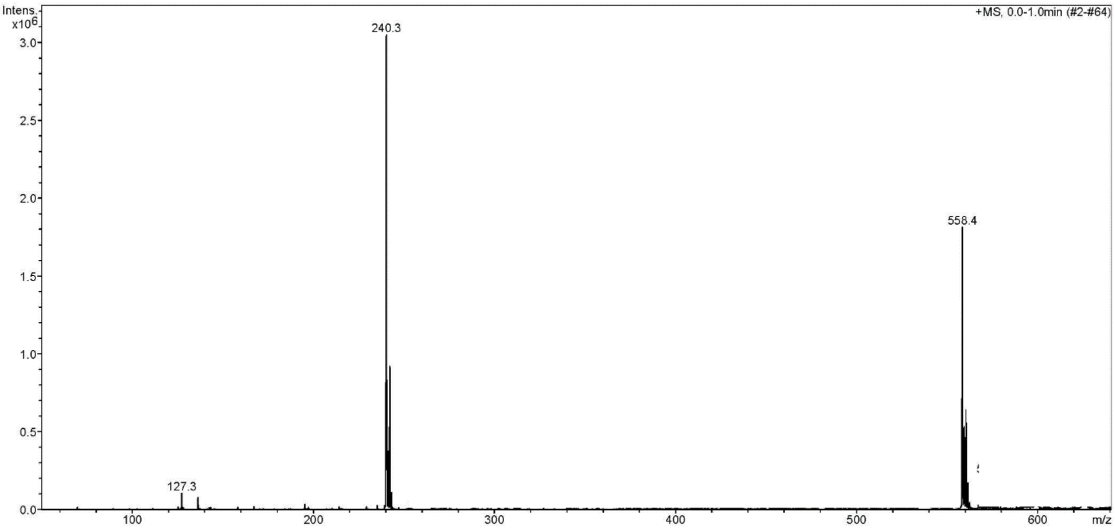

Bruker Daltonics DataAnalysis 3.1 printed: 03/18/22 11:29:14 Page 1 of 1

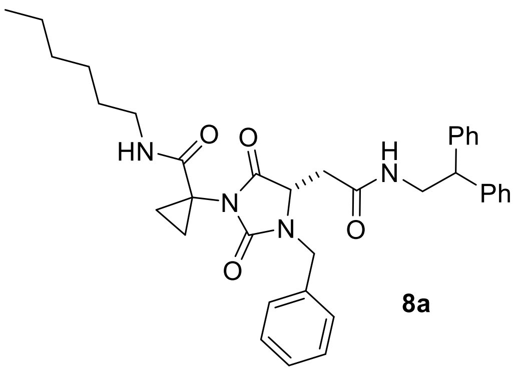

8a

^{1}H-NMR (400 MHz, $CDCl_3$)

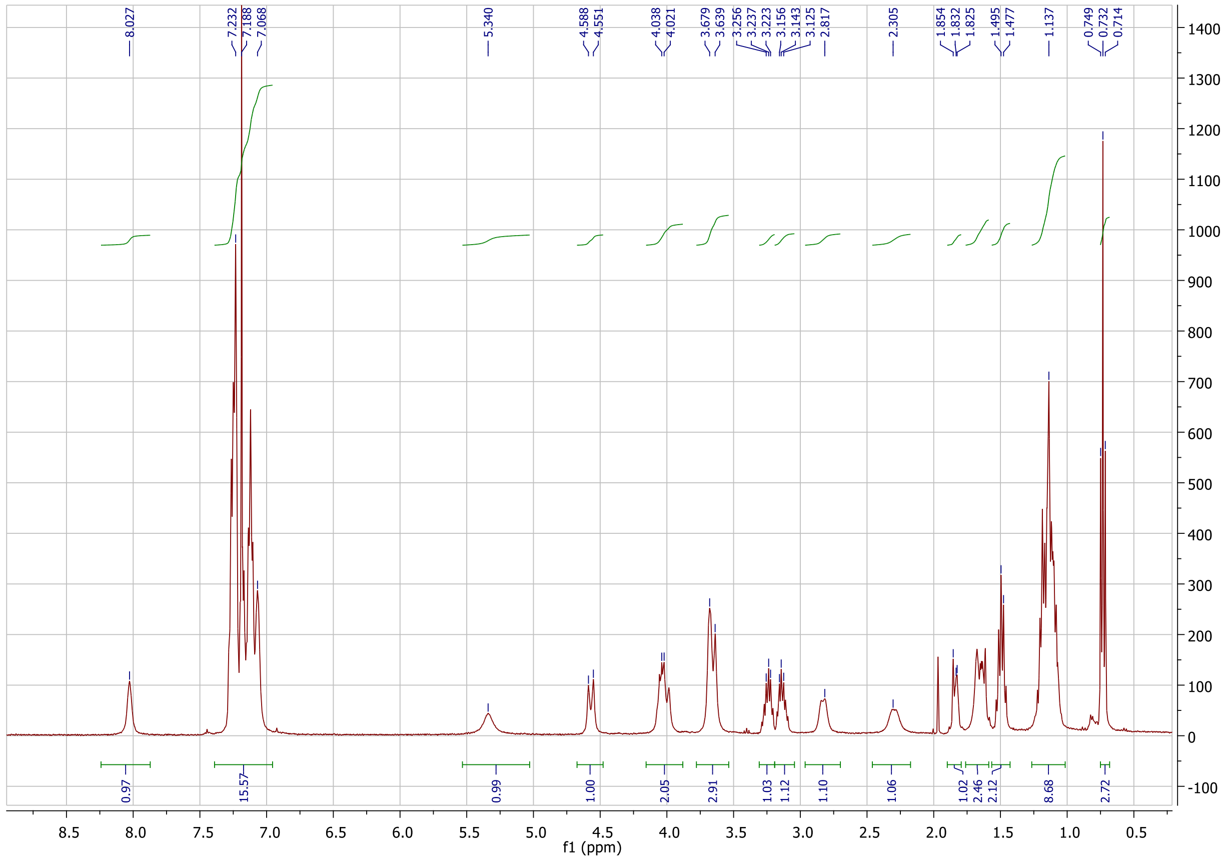

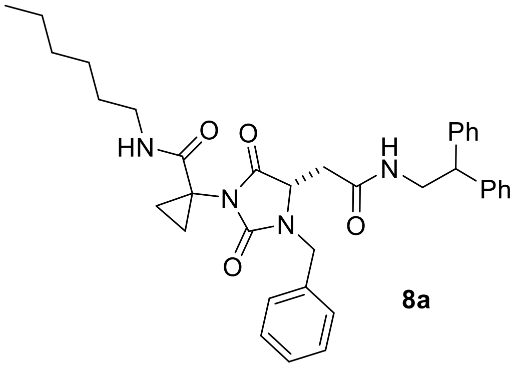

8a

$^{13}C\{^1H\}$-NMR (101 MHz, $CDCl_3$)

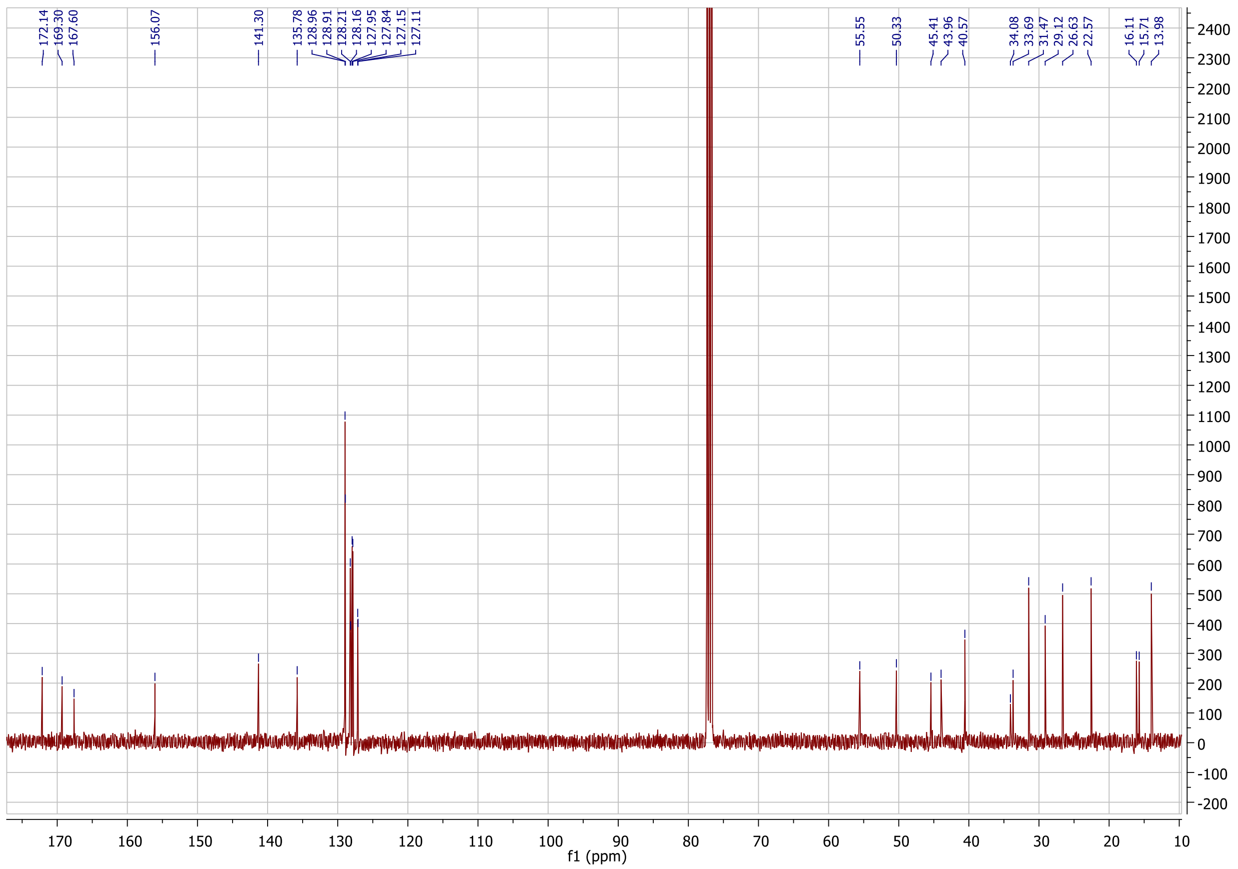

-L.G.S. - Laboratorio Grandi Strumenti - Display Report

Analysis Name	av fm2856.d	Acquisition Date	07/13/17 13:23:26	Operator	Walter Panzeri
Sample Name		Method	Copy of _01tmix_posneg lm.MS	Instrument	esquire3000plus
Comment	1 mg/ml dil 1:100 MeOH<br>Richiedente: Frigerio				

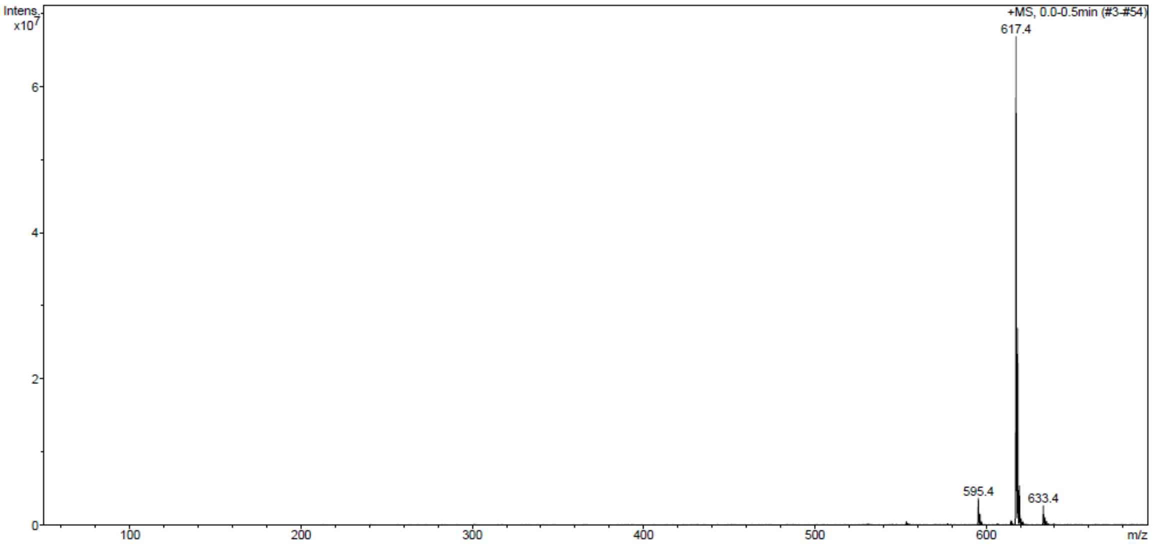

Bruker Daltonics DataAnalysis 3.1 printed: 07/13/17 13:24:17 Page 1 of 1

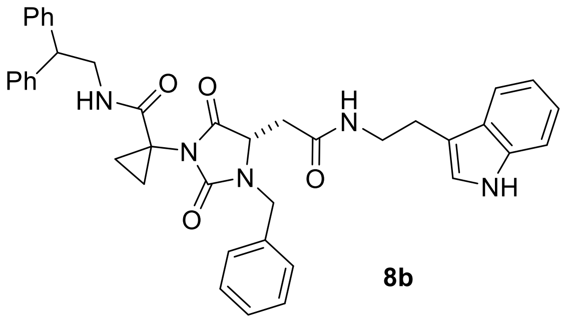

8b

^{1}H-NMR (101 MHz, dmso-d_6)

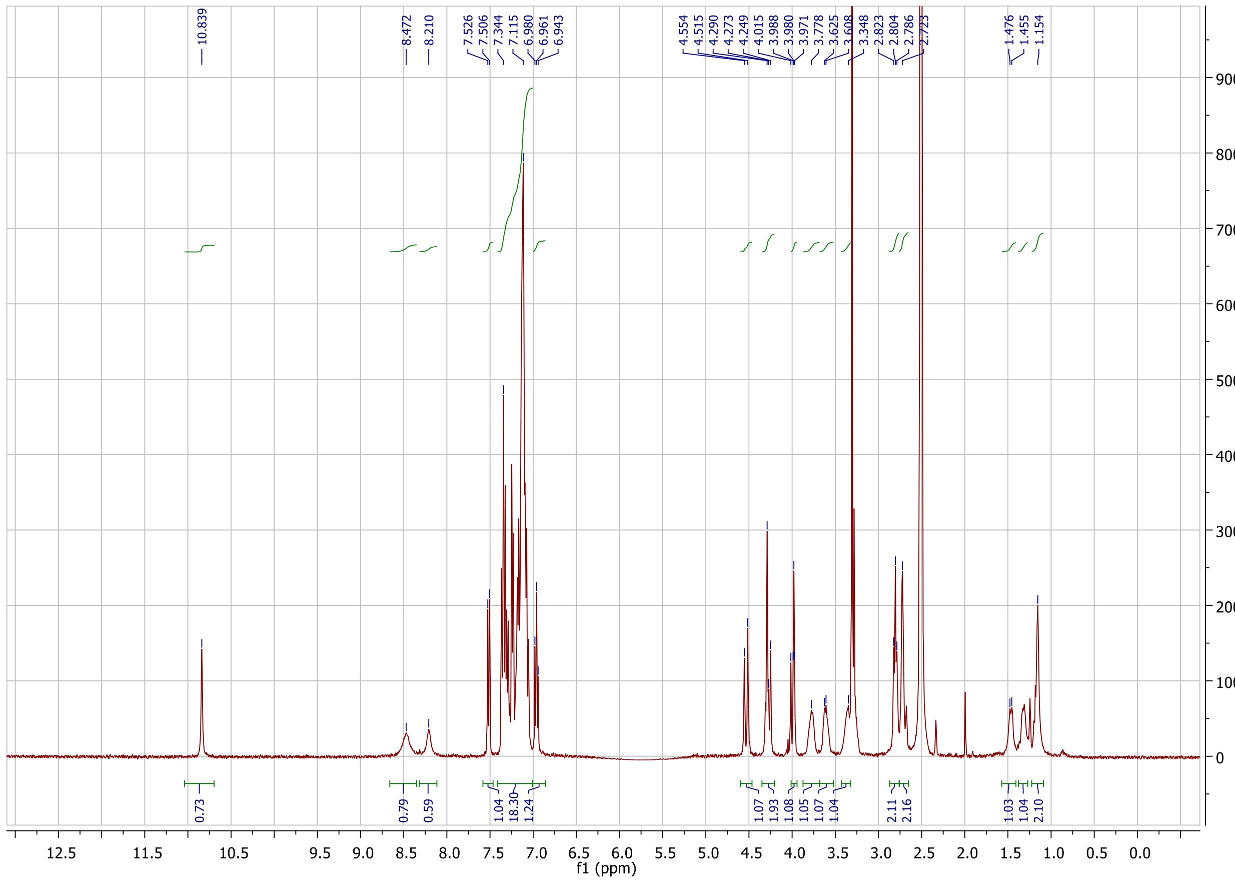

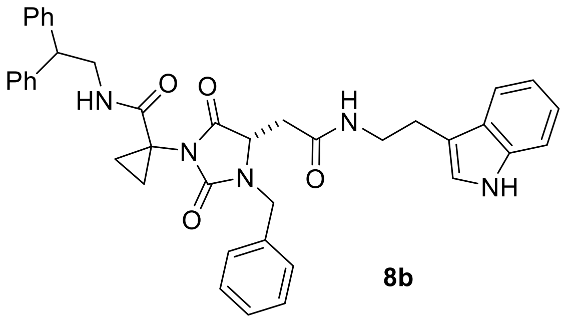

8b

$^{13}C\{^{1}H\}$-NMR (101 MHz, $CDCl_3$)

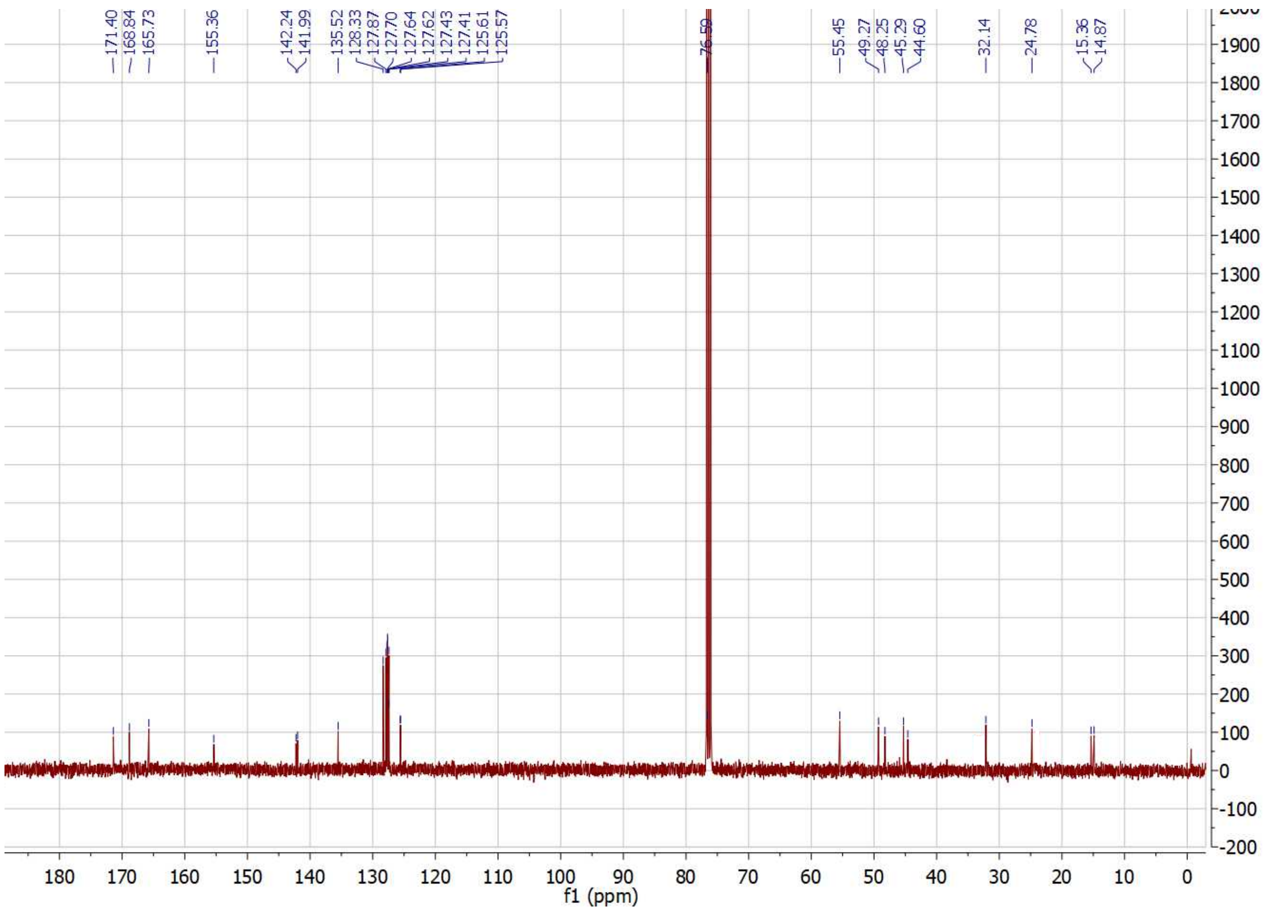

-L.G.S. - Laboratorio Grandi Strumenti - Display Report

Analysis Name	av gc111.d	Acquisition Date	02/08/19 14:15:22	Operator	Walter Panzeri
Sample Name		Method	Copy of _01tmix_posneg lm.MS	Instrument	esquire3000plus
Comment	1 mg/ml dil 1:100 MeOH<br>Richiedente: Cristina				

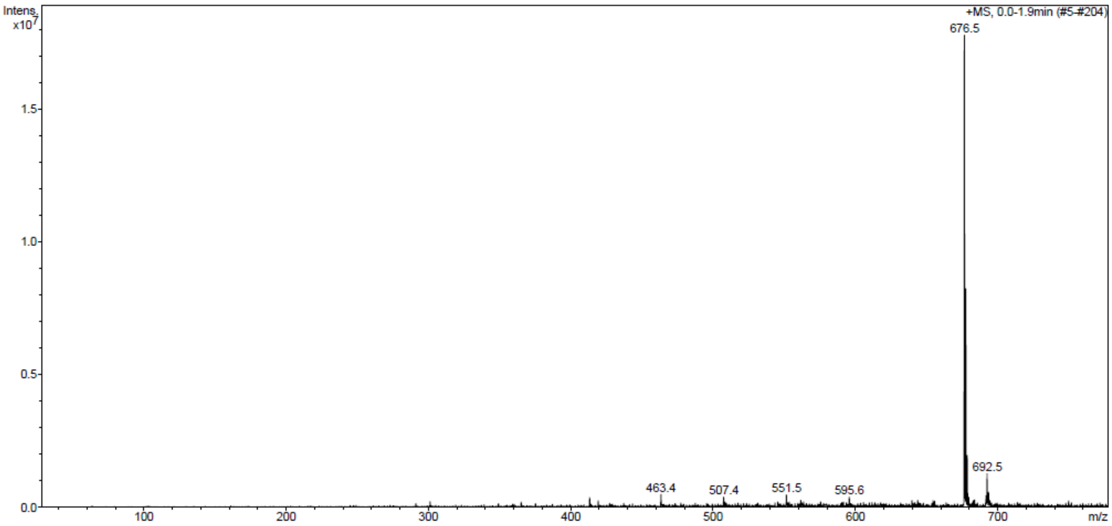

Bruker Daltonics DataAnalysis 3.1 printed: 02/08/19 14:25:49 Page 1 of 1

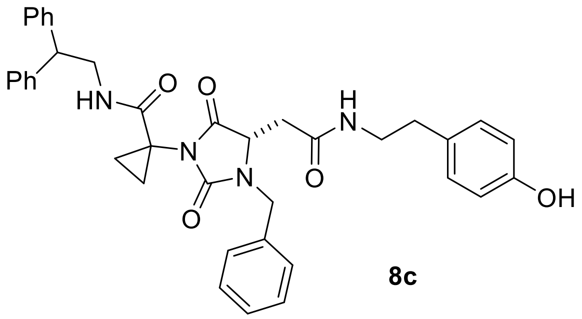

8c

^{1}H-NMR (400 MHz, dmso-d$_6$)

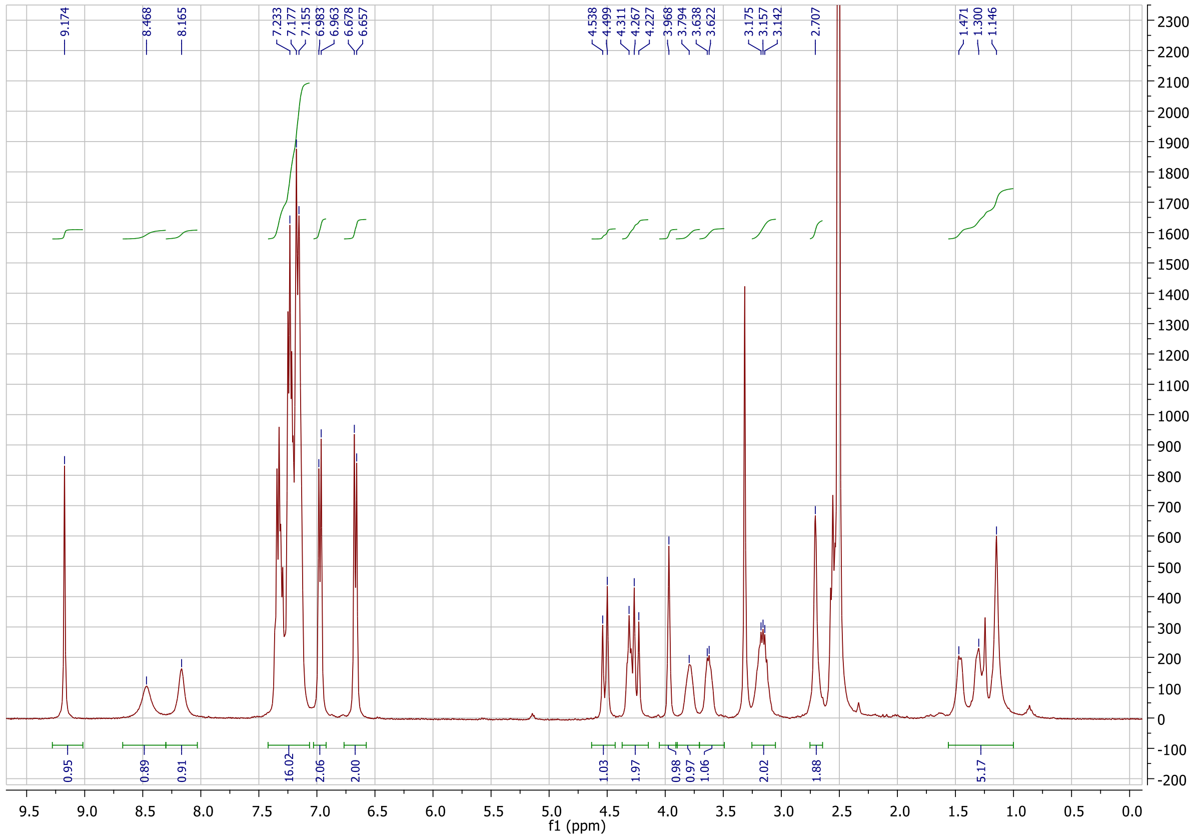

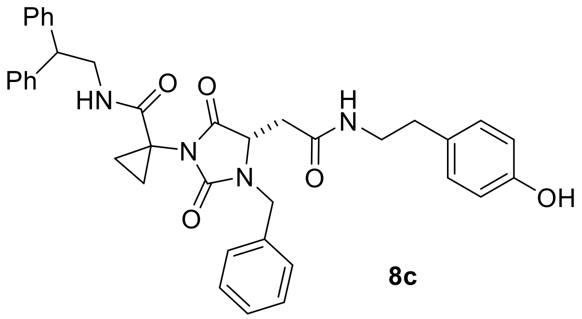

8c

$^{13}C\{^{1}H\}$-NMR (101 MHz, dmso-d_6)

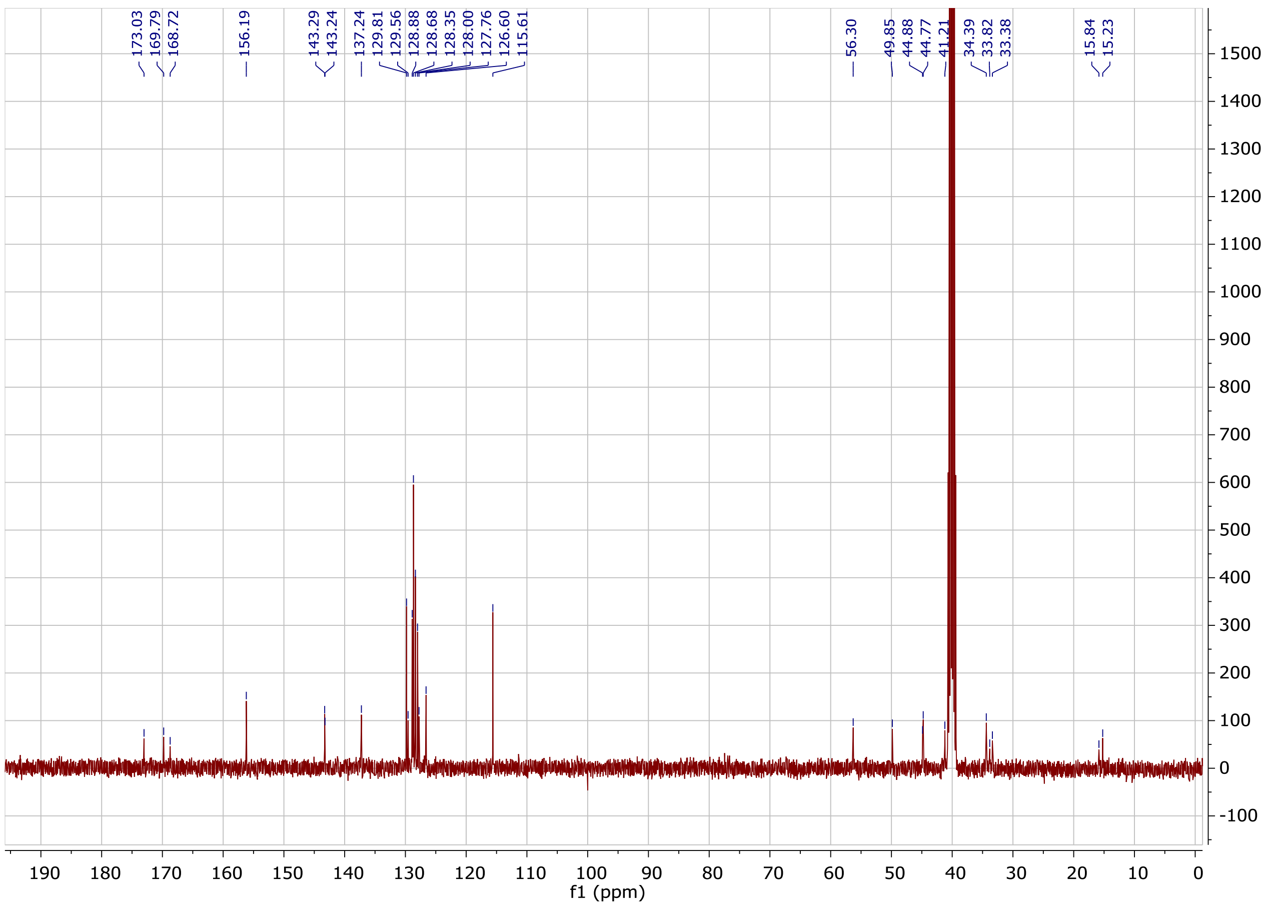

-L.G.S. - Laboratorio Grandi Strumenti - Display Report

Analysis Name	av ac 172.d	Acquisition Date	11/10/21 10:44:30	Operator	Walter Panzeri
Sample Name		Method	Copy of $opt0622.MS	Instrument	esquire3000plus
Comment	1 mg/ml dil 1:100 MeOH<br>Richiedente: Caramiello				

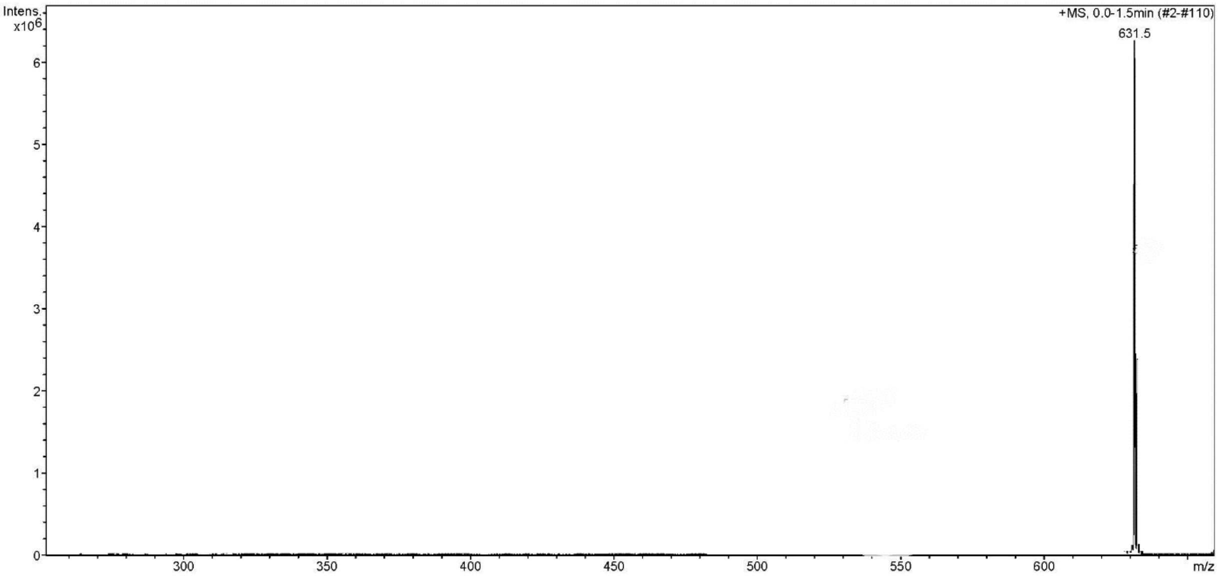

Bruker Daltonics DataAnalysis 3.1 printed: 11/10/21 10:50:17 Page 1 of 1

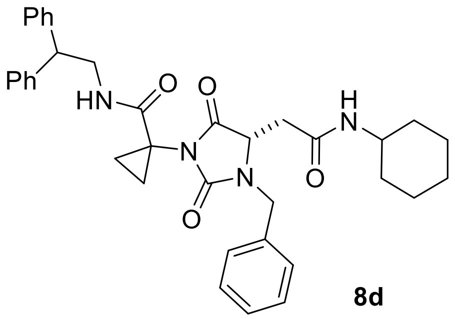

8d

^{1}H-NMR (400 MHz, $CDCl_3$)

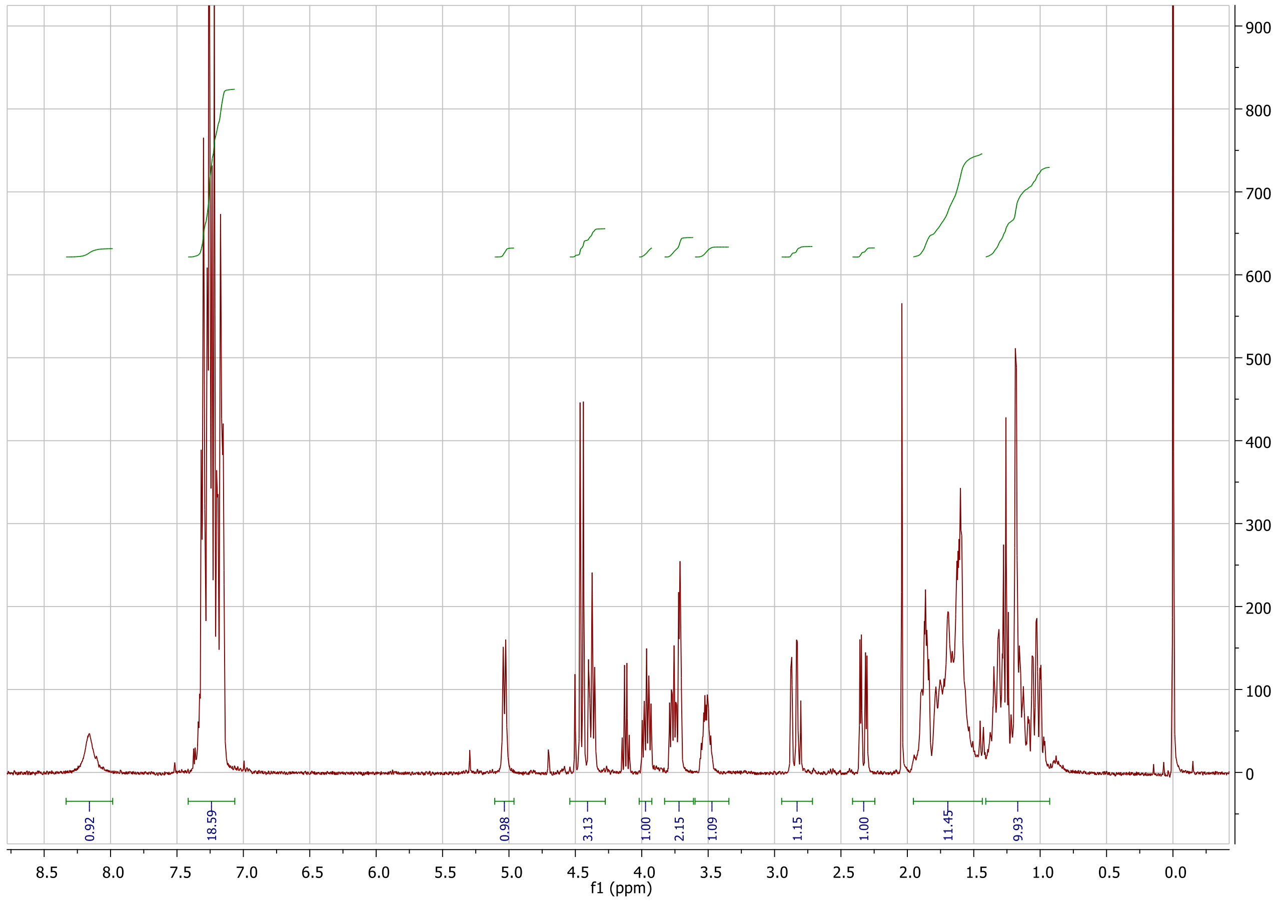

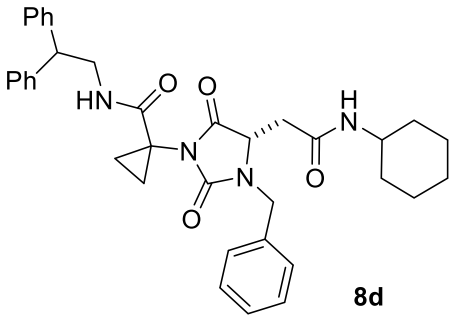

8d

$^{13}C\{^{1}H\}$-NMR (101 MHz, $CDCl_3$)

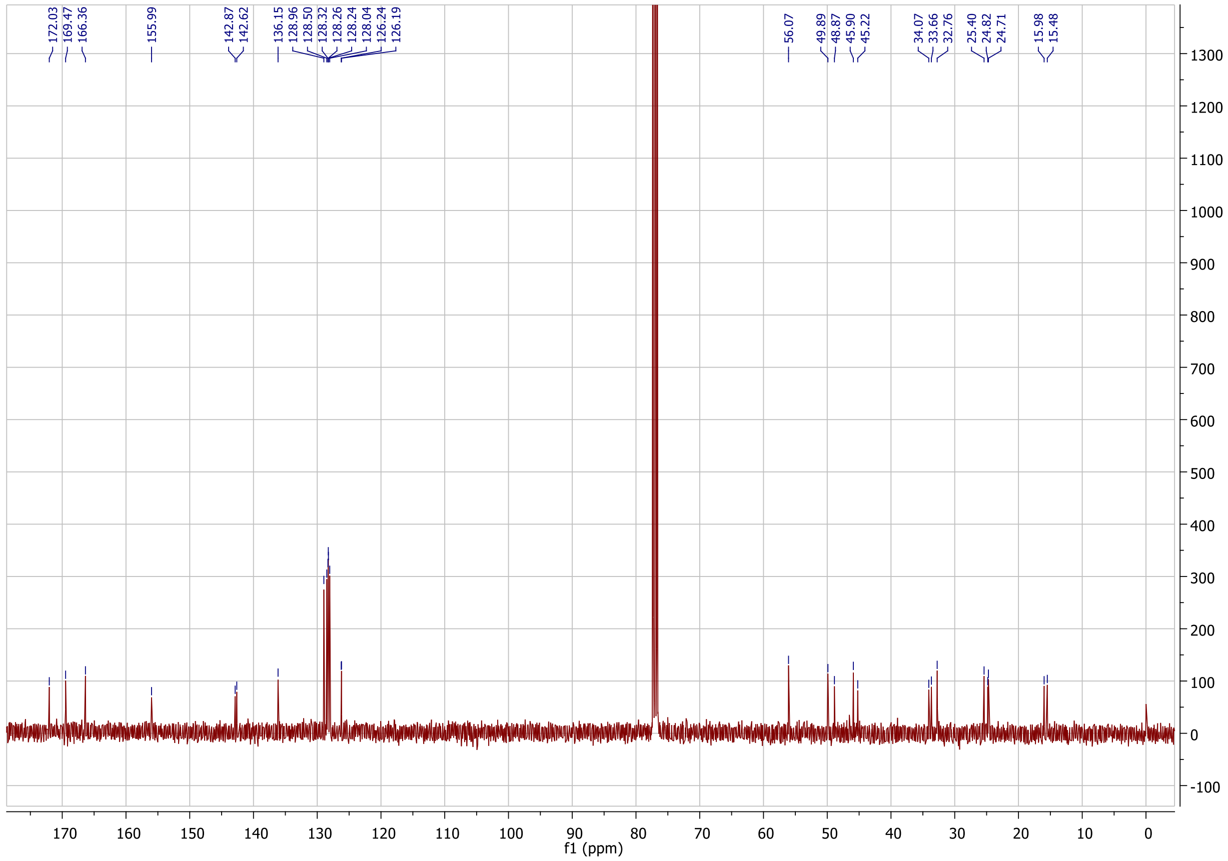

-L.G.S. - Laboratorio Grandi Strumenti - Display Report

Analysis Name	av fv2063.d	Acquisition Date	02/04/19 14:42:37	Operator	Walter Panzeri
Sample Name		Method	Copy of _01tmix_posneg lm.MS	Instrument	esquire3000plus
Comment	1 mg/ml dil 1:100 MeOH<br>Richiedente: Viani				

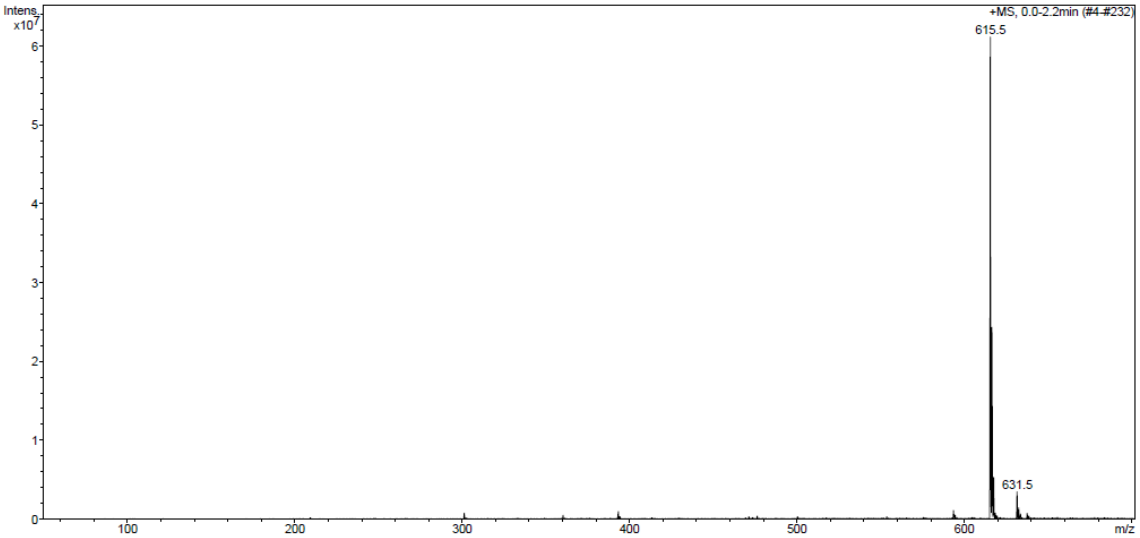

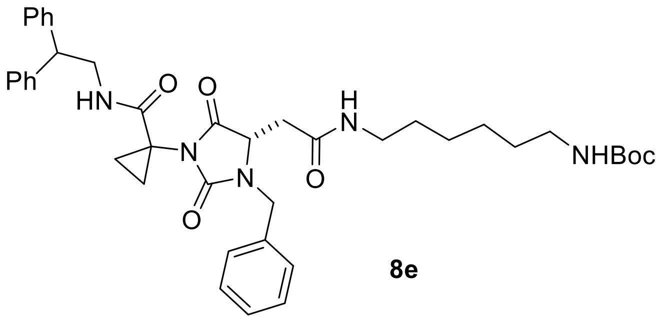

8e

^{1}H-NMR (400 MHz, CD_3OD)

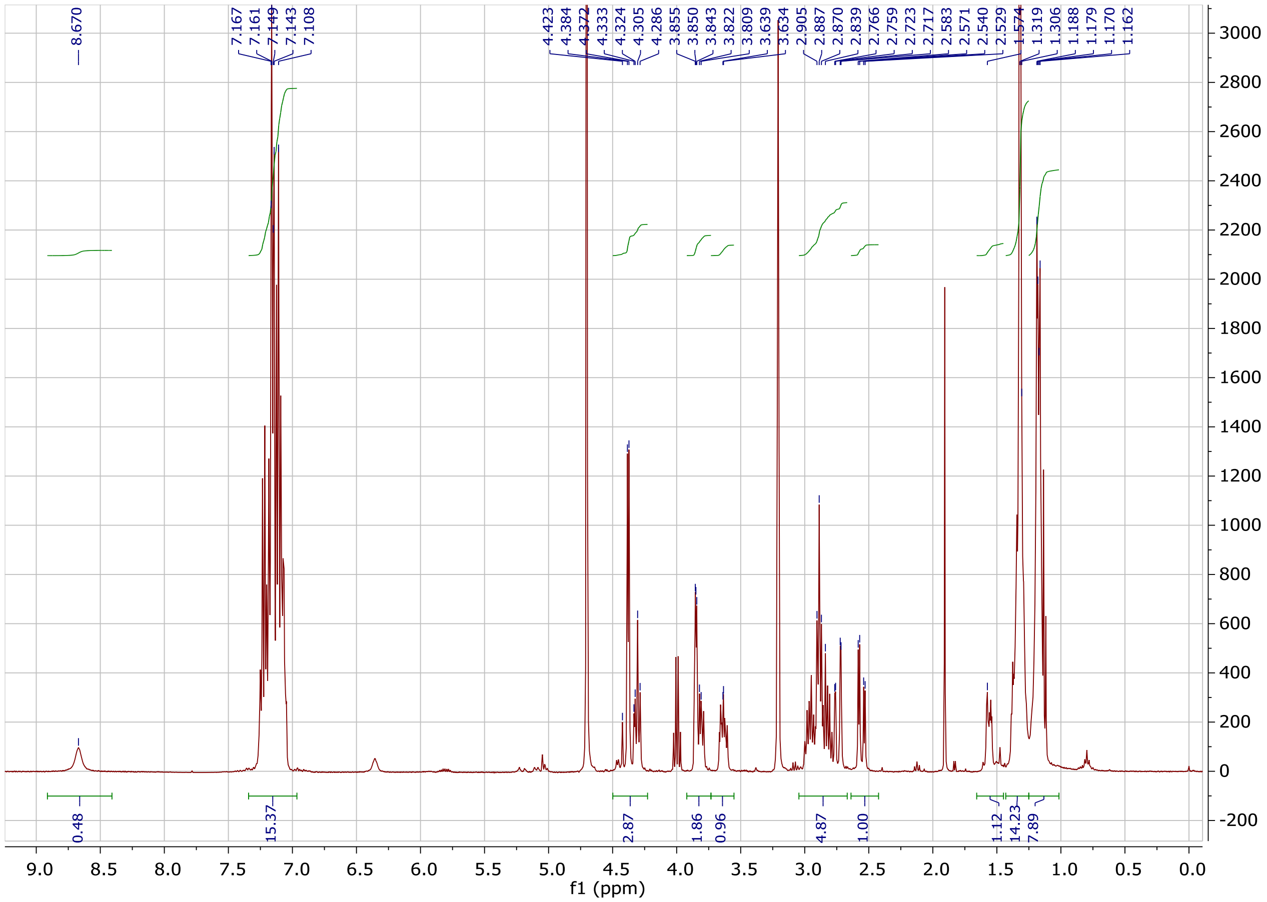

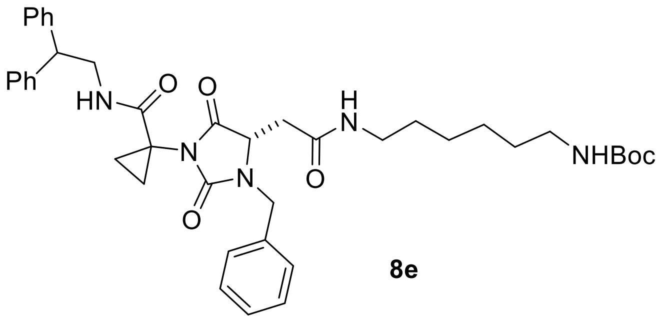

8e

$^{13}C\{^{1}H\}$-NMR (101 MHz, CD_3OD)

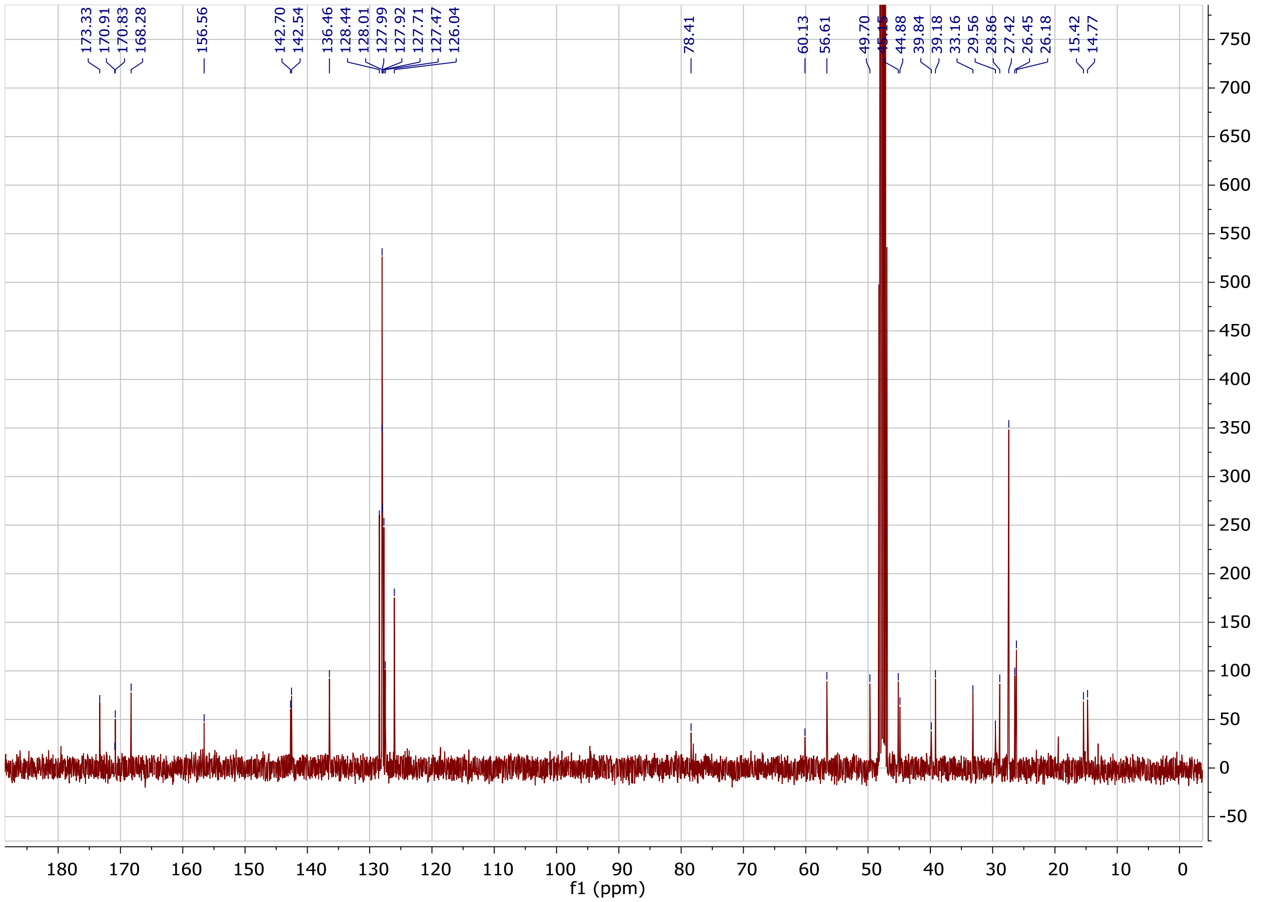

-L.G.S. - Laboratorio Grandi Strumenti - Display Report

Analysis Name	av gc113.d	Acquisition Date	03/06/19 14:49:10	Operator	Walter Panzeri
Sample Name		Method	Copy of _01tmix_posneg lm.MS	Instrument	esquire3000plus
Comment	1 mg/ml dil 1:100 MeOH<br>Richiedente: Cristina				

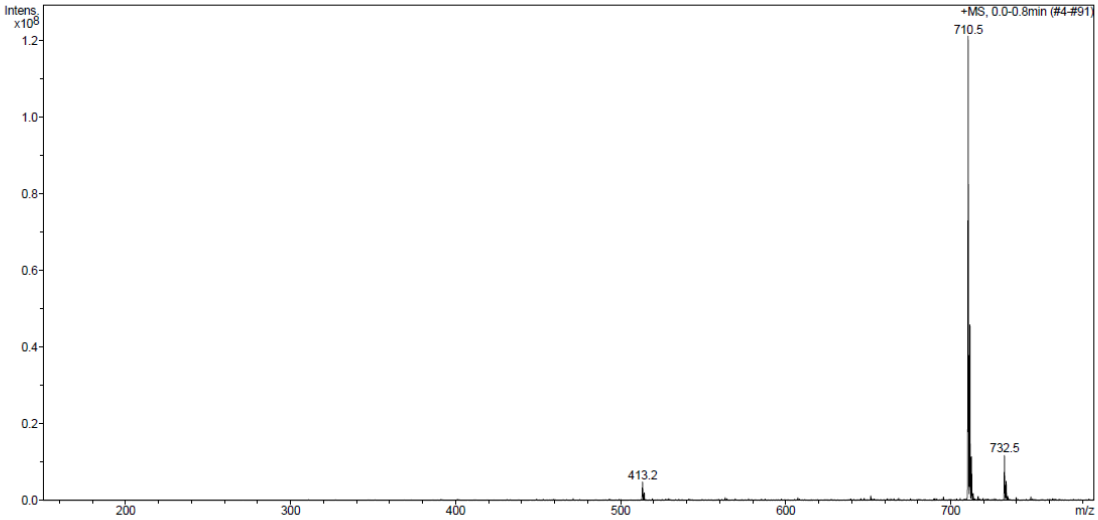

Bruker Daltonics DataAnalysis 3.1 printed: 03/06/19 15:05:46 Page 1 of 1

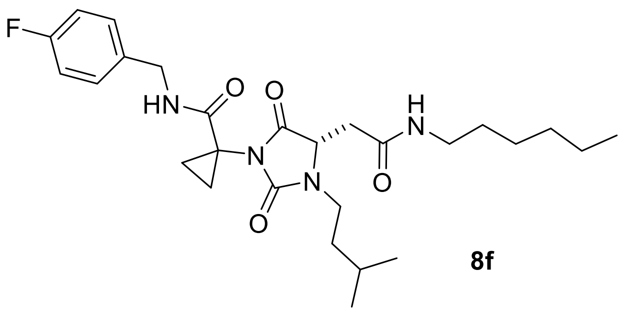

8f

^{1}H-NMR (400 MHz, CD_3OD)

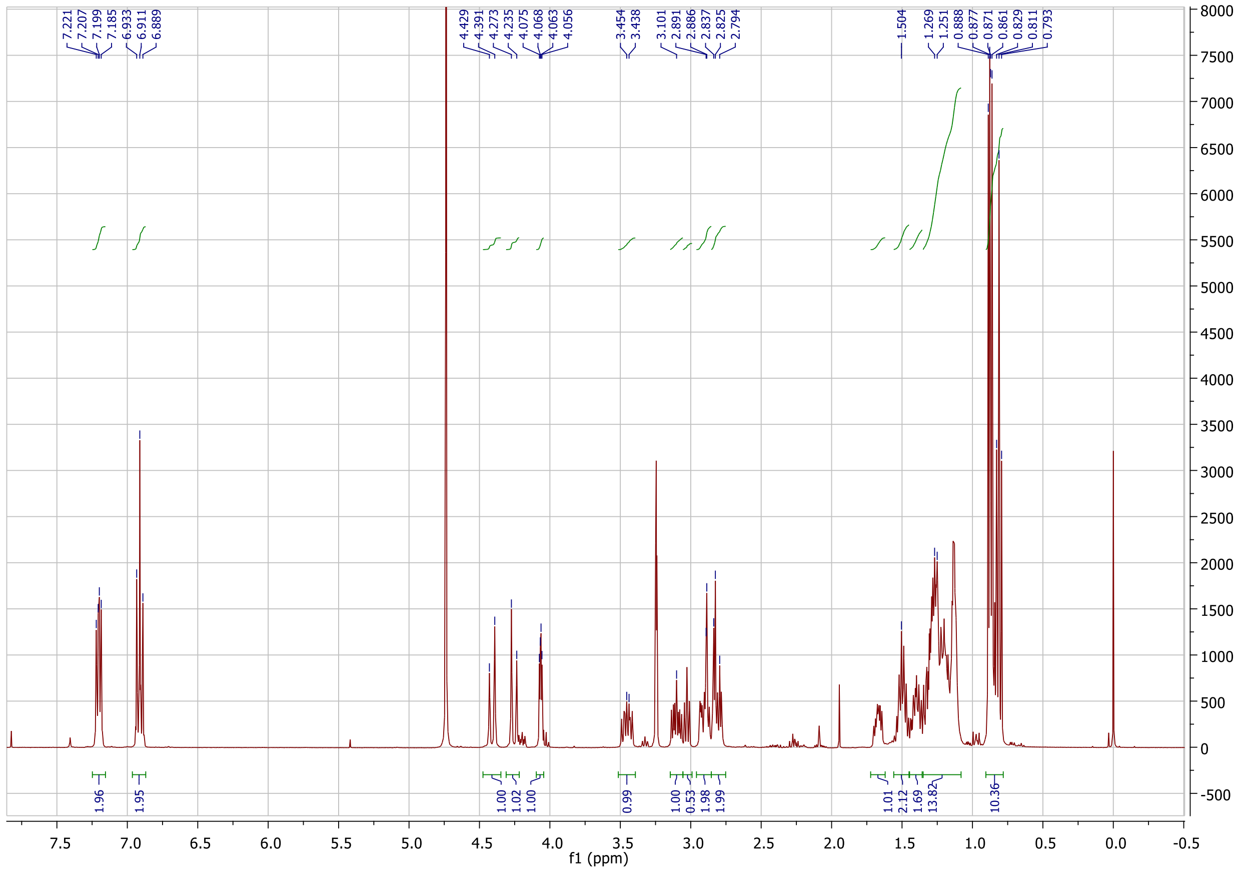

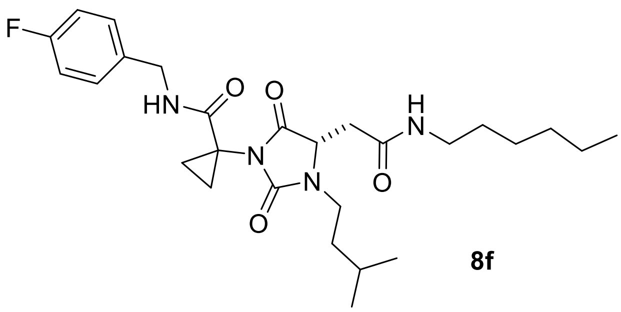

8f

$^{13}C\{^{1}H\}$-NMR (101 MHz, CD_3OD)

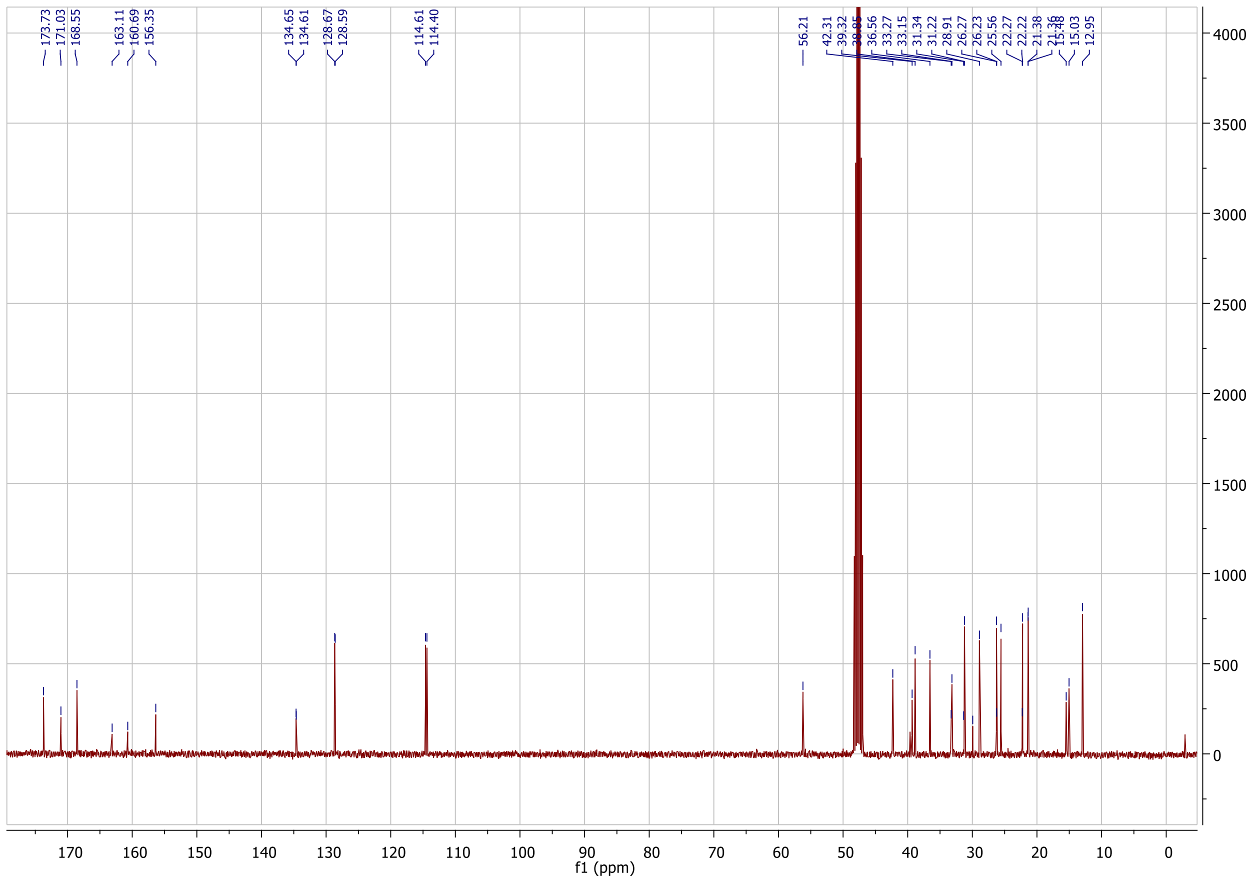

-L.G.S. - Laboratorio Grandi Strumenti - Display Report

Analysis Name	av ac132c.d	Acquisition Date	04/13/21 15:57:54	Operator	Walter Panzeri
Sample Name		Method	Copy of $PW_TBLM.MS	Instrument	esquire3000plus
Comment	1 mg/ml dil 1:100 MeOH<br>Richiedente: Caramiello				

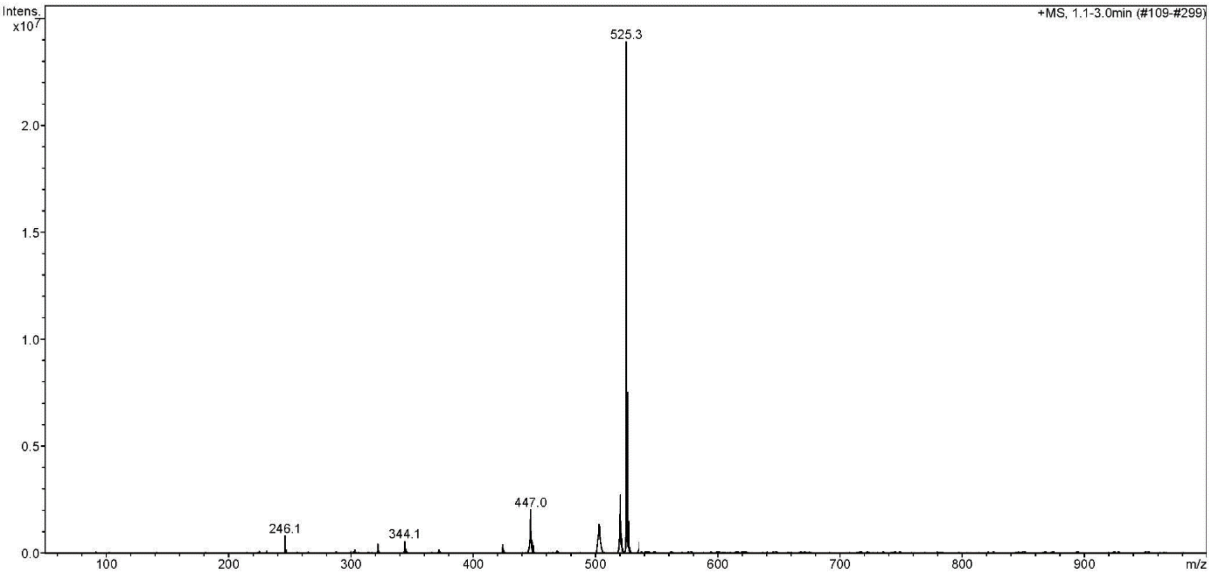

Bruker Daltonics DataAnalysis 3.1 printed: 04/13/21 16:01:57 Page 1 of 1

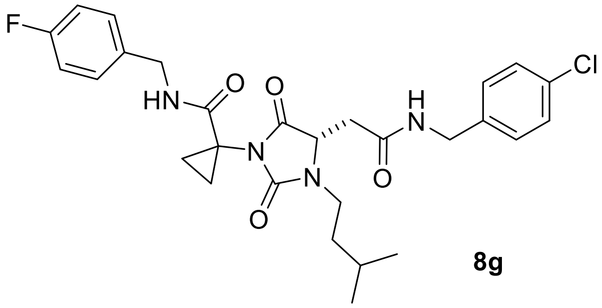

8g

^{1}H-NMR (101 MHz, CD_3OD)

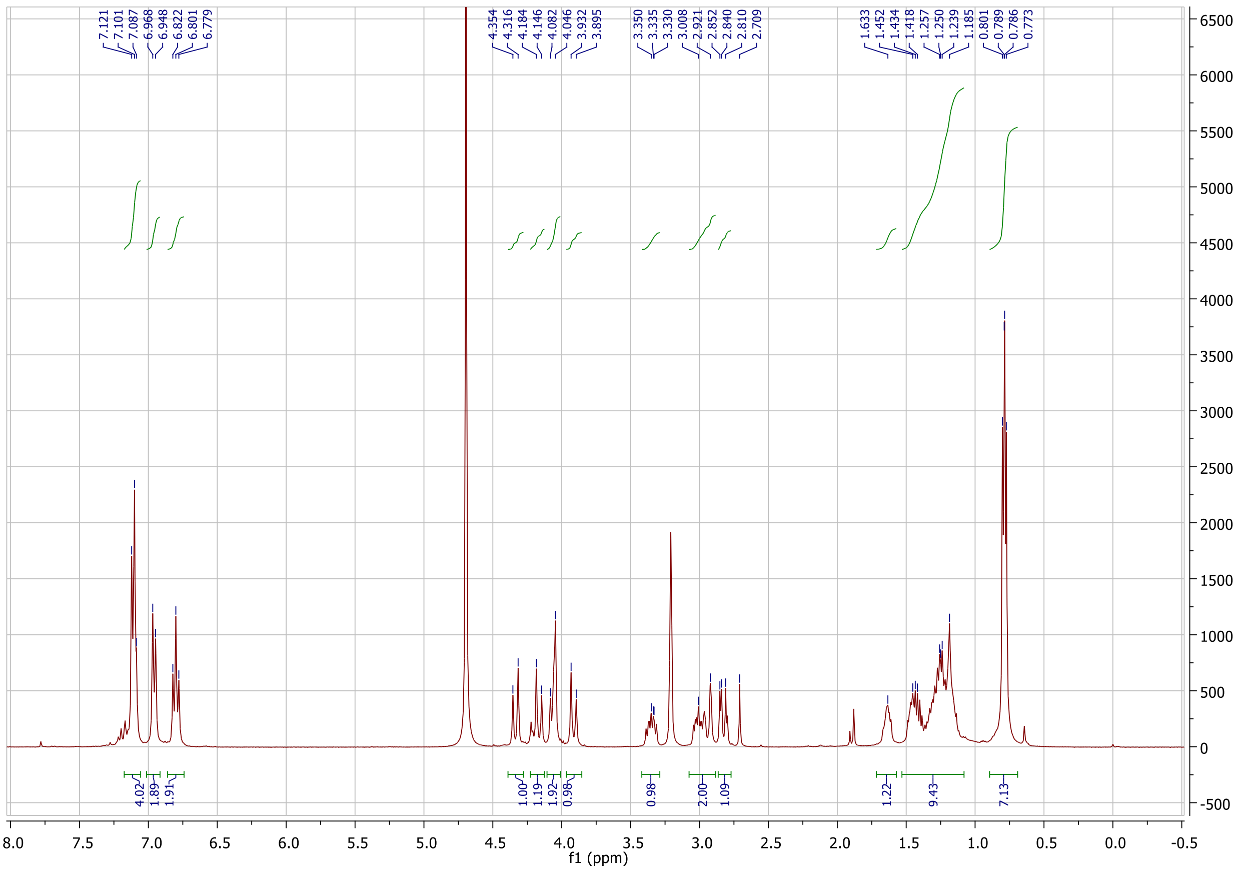

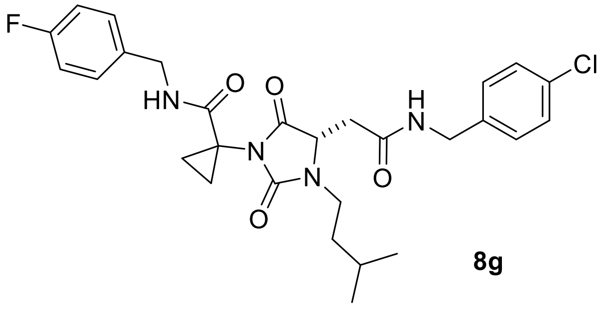

8g

$^{13}C\{^{1}H\}$-NMR (101 MHz, CD_3OD)

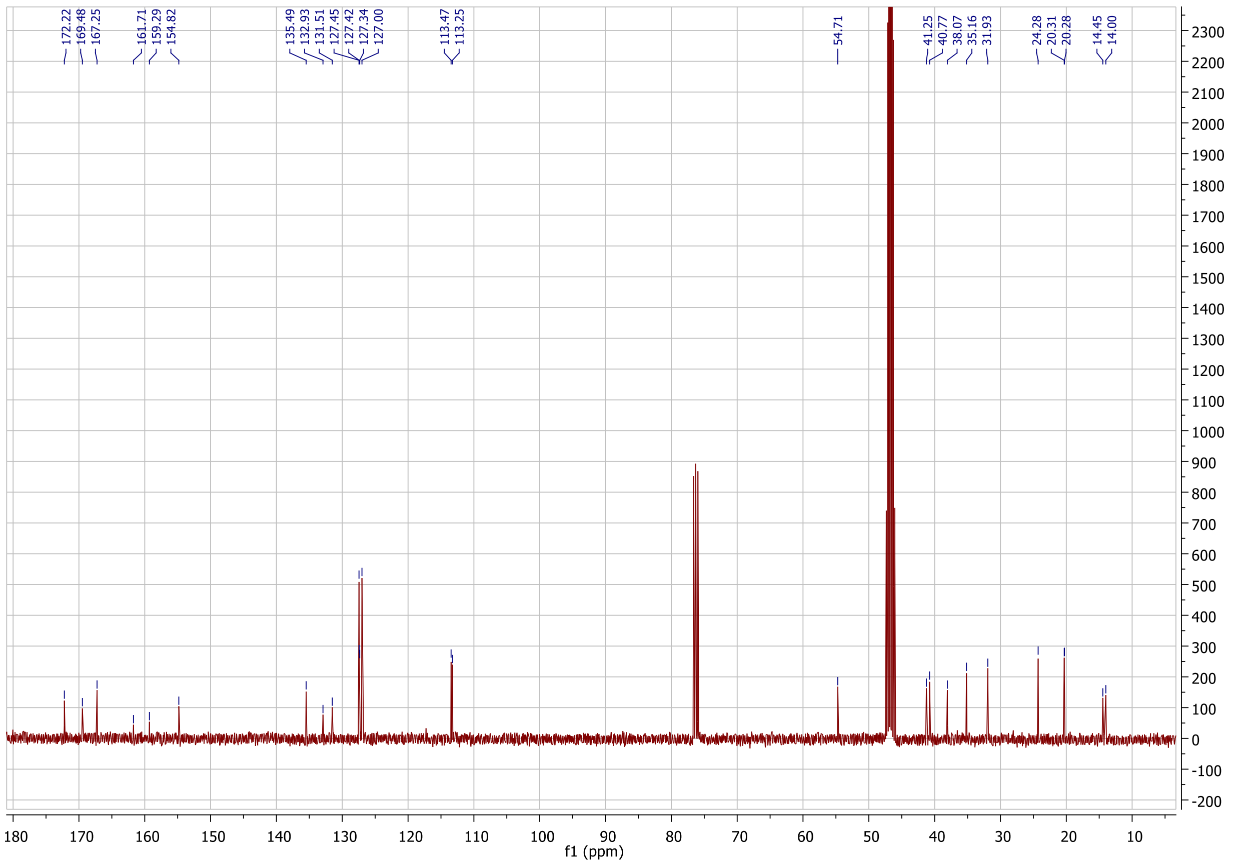

-L.G.S. - Laboratorio Grandi Strumenti - Display Report

Analysis Name	av ms2057.d	Acquisition Date	07/08/22 09:17:41	Operator	Walter Panzeri
Sample Name		Method	Copy of $opt0622.MS	Instrument	esquire3000plus
Comment	1 mg/mL dil 1:100 MeOH<br>Richiedente: Sani				

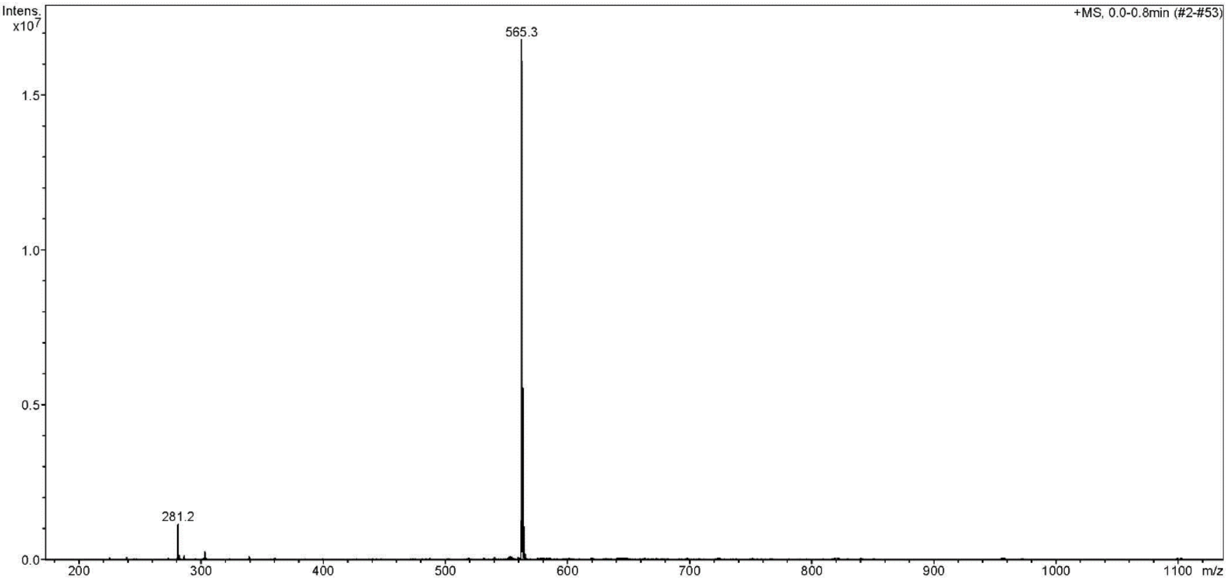

Bruker Daltonics DataAnalysis 3.1 printed: 07/08/22 09:19:05 Page 1 of 1

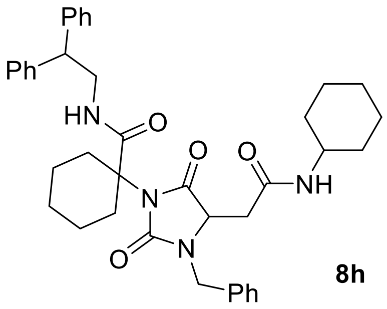

8h

^{1}H-NMR (400 MHz, $CDCl_3$)

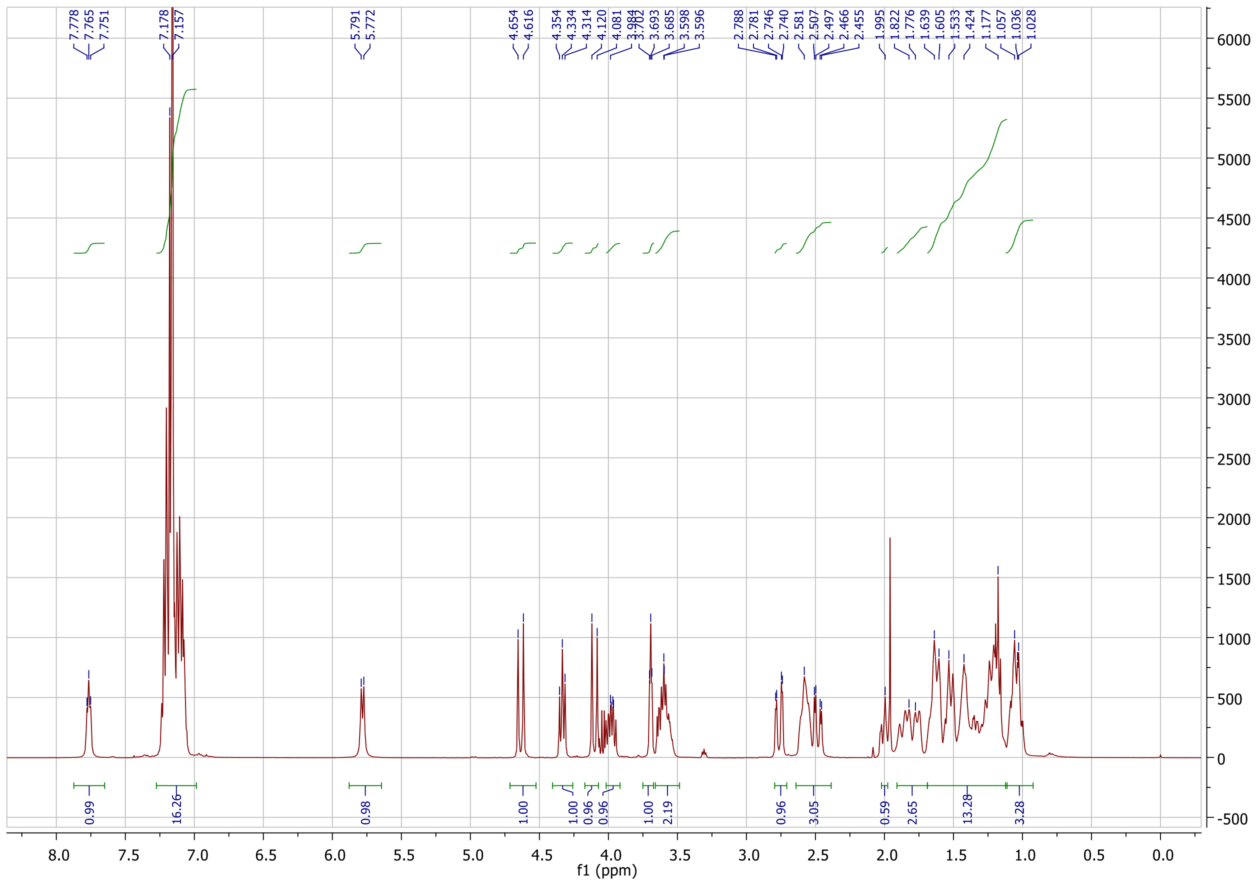

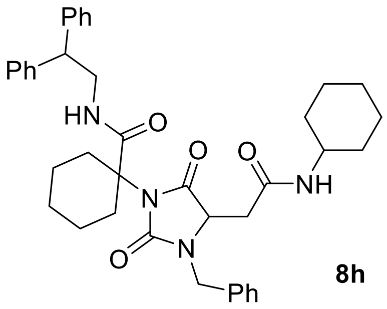

8h

$^{13}C\{^{1}H\}$-NMR (101 MHz, $CDCl_3$)

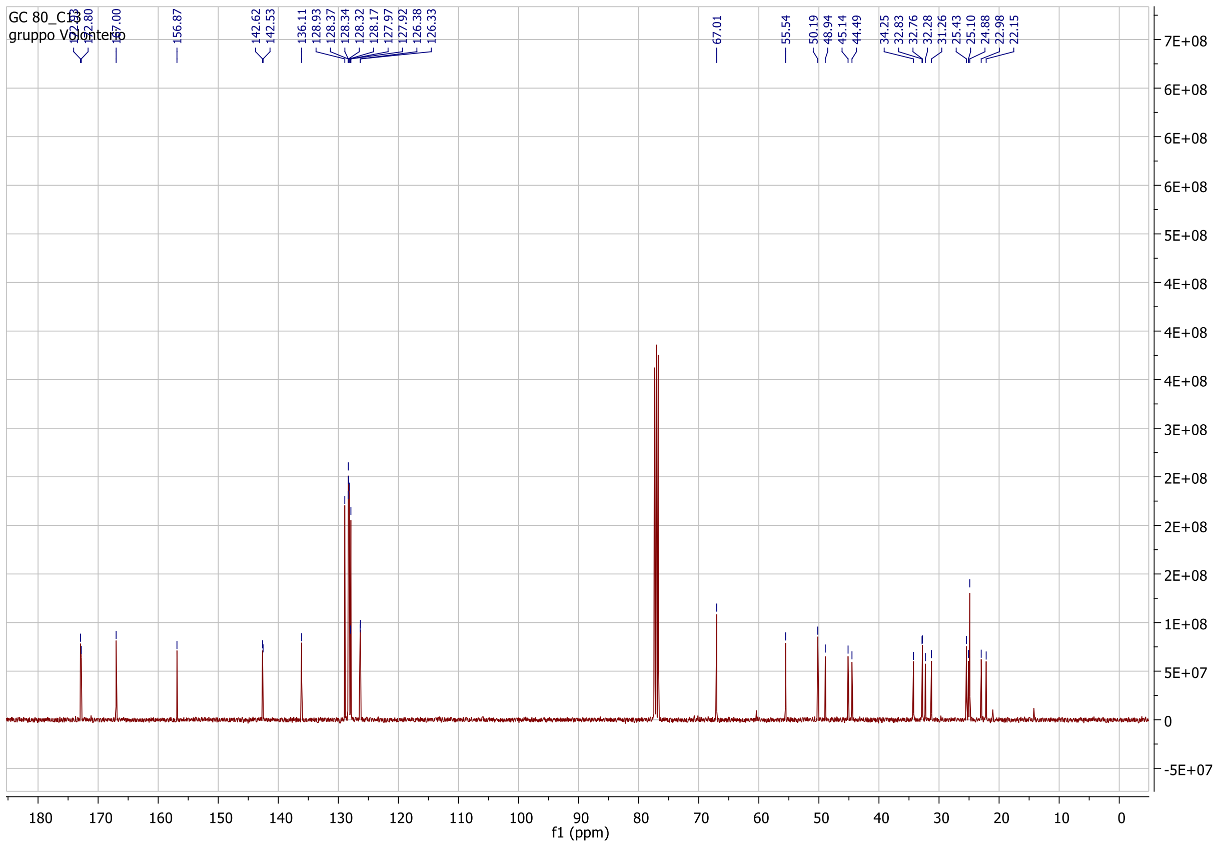

-L.G.S. - Laboratorio Grandi Strumenti - Display Report

Analysis Name	av gc80.d	Acquisition Date	09/14/18 10:53:50	Operator	Walter Panzeri
Sample Name		Method	Copy of _01tmix_posneg lm.MS	Instrument	esquire3000plus
Comment	1 mg/ml dil 1:100 MeOH<br>Richiedente: Cristina				

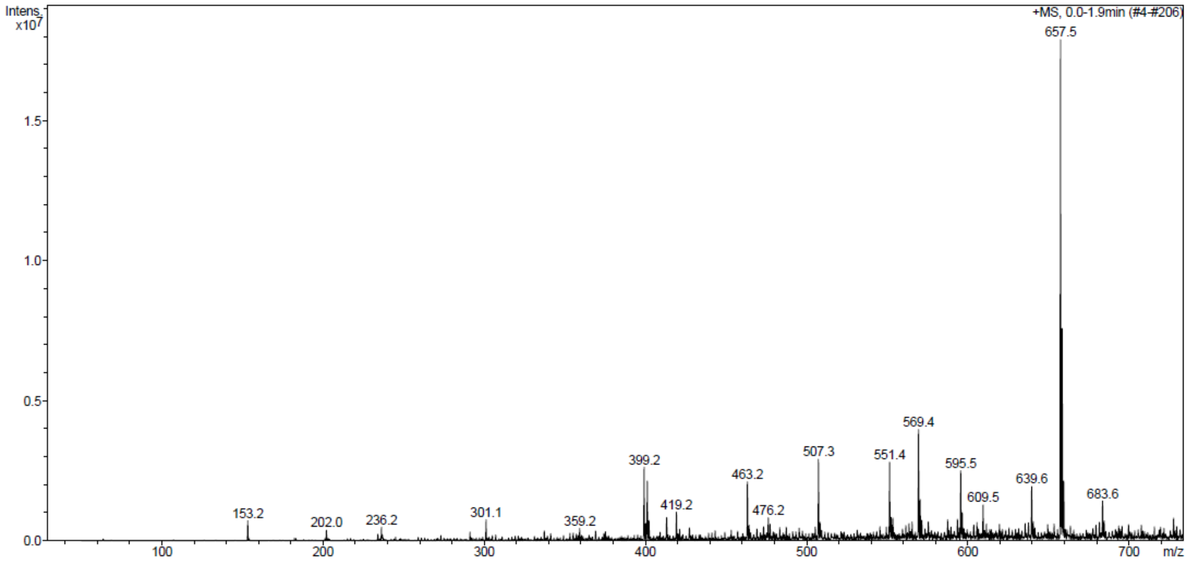

Bruker Daltonics DataAnalysis 3.1 printed: 09/14/18 10:56:40 Page 1 of 1

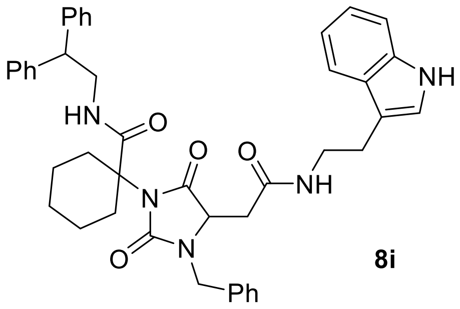

8i

^{1}H-NMR (400 MHz, $CDCl_3$)

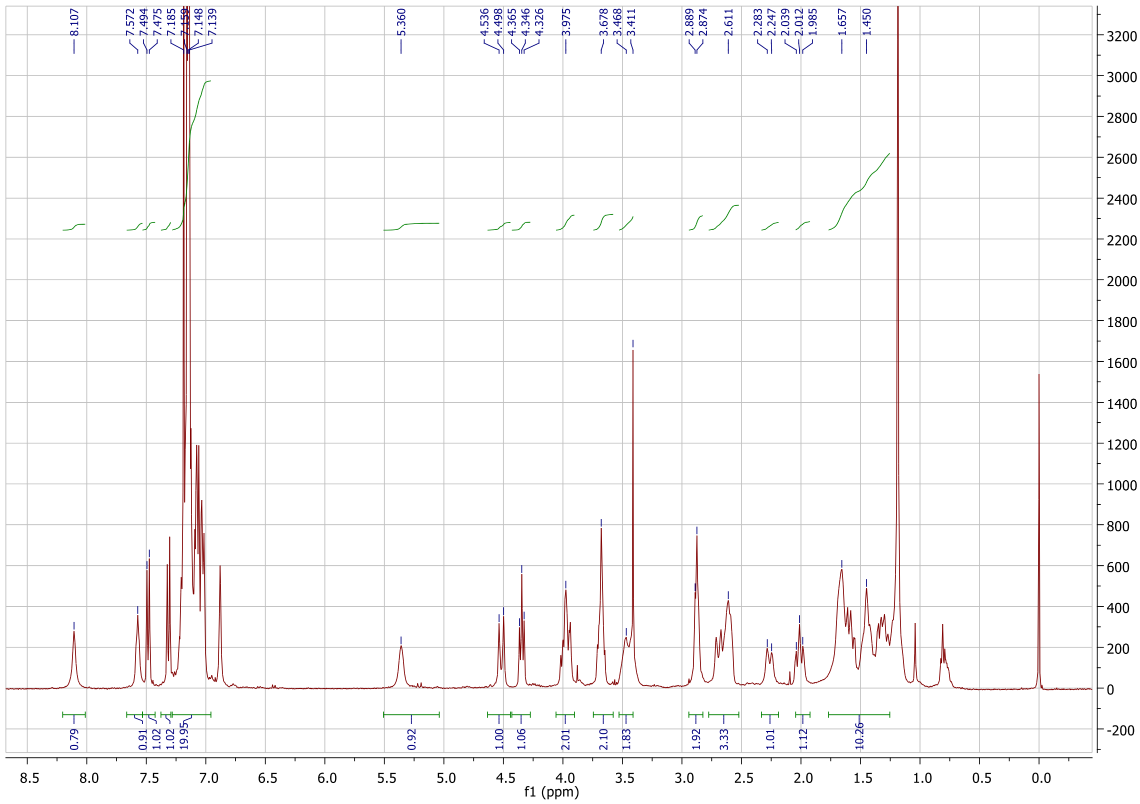

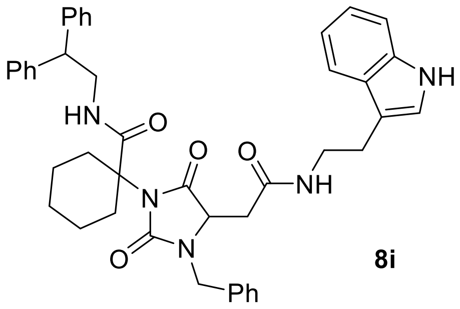

8i

$^{13}C\{^{1}H\}$-NMR (101 MHz, $CDCl_3$)

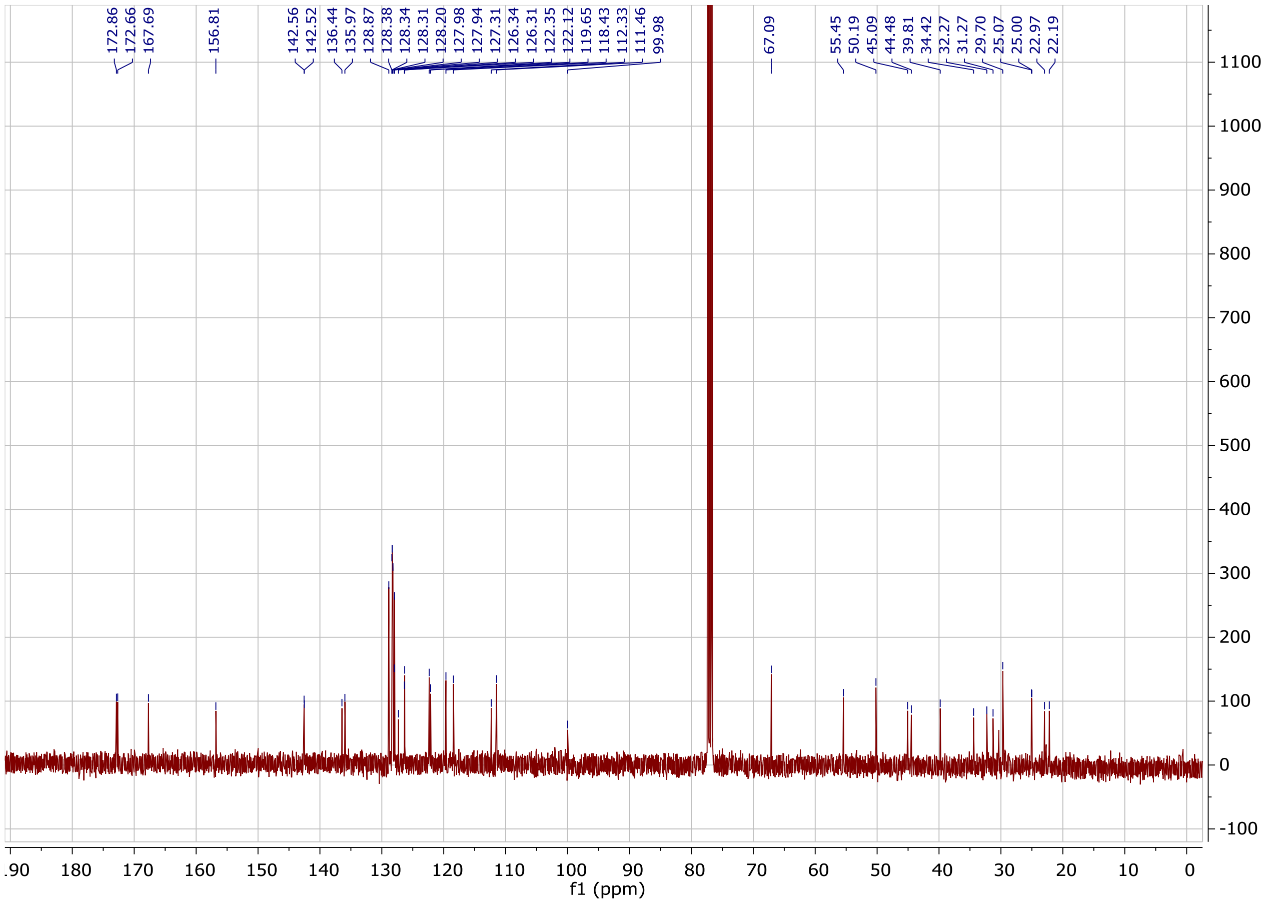

-L.G.S. - Laboratorio Grandi Strumenti - Display Report

Analysis Name	av md20g_f.d	Acquisition Date	04/24/19 14:19:55	Operator	Walter Panzeri
Sample Name		Method	Copy of _01tmix_posneg lm.MS	Instrument	esquire3000plus
Comment	1 mg/ml dil 1:100 CH3CN<br>Richiedente: Viani				

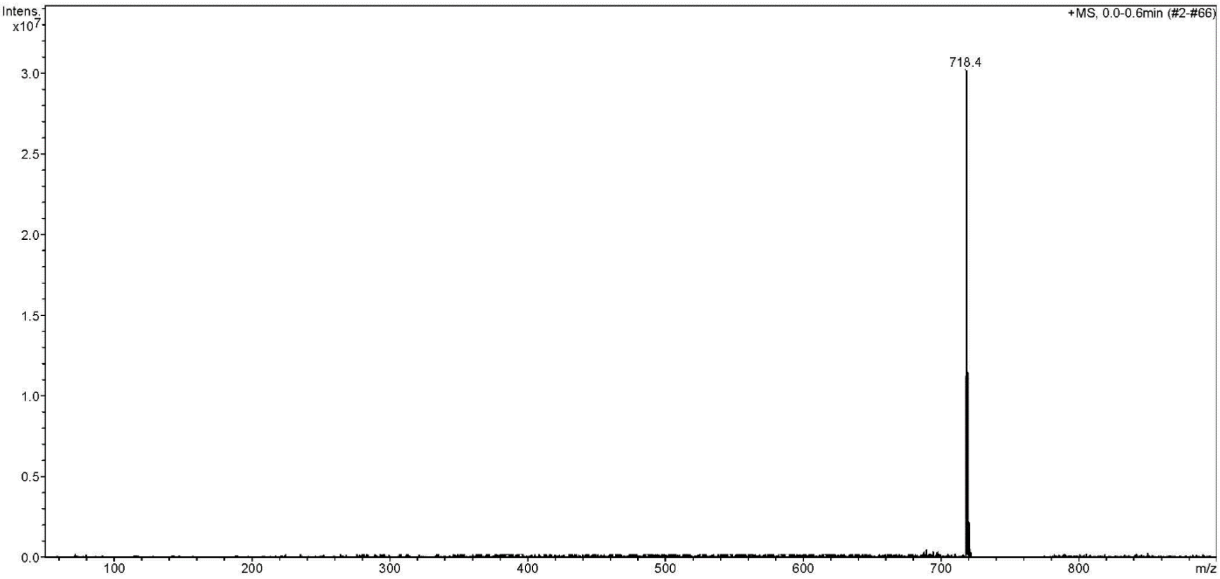

Bruker Daltonics DataAnalysis 3.1 printed: 04/24/19 14:50:02 Page 1 of 1

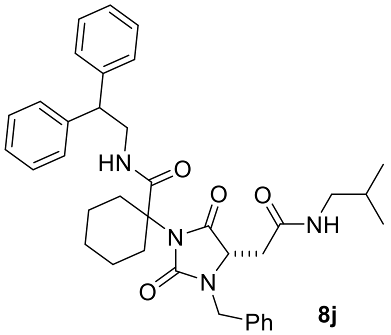

8j

^{1}H-NMR (400 MHz, $CDCl_3$)

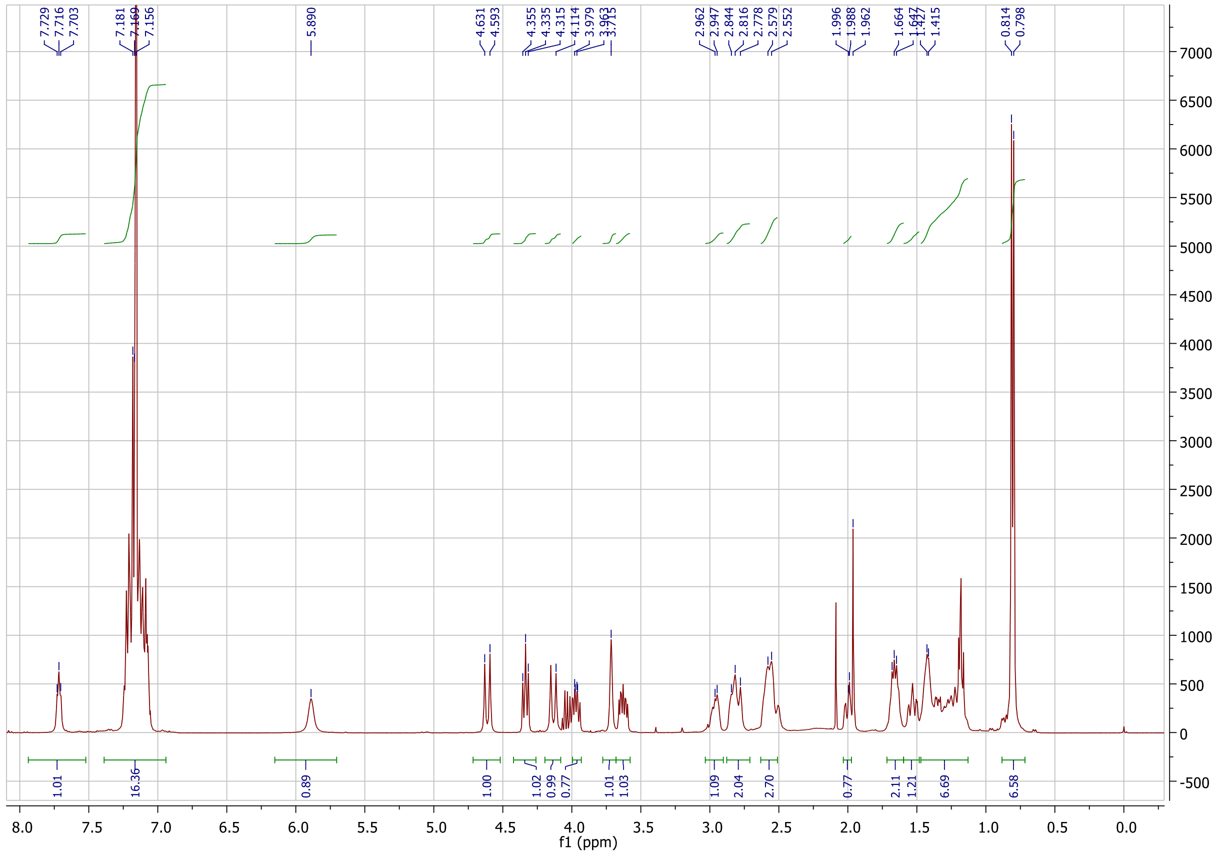

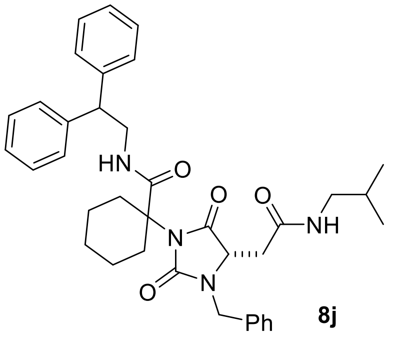

8j

$^{13}C\{^{1}H\}$-NMR (101 MHz, $CDCl_3$)

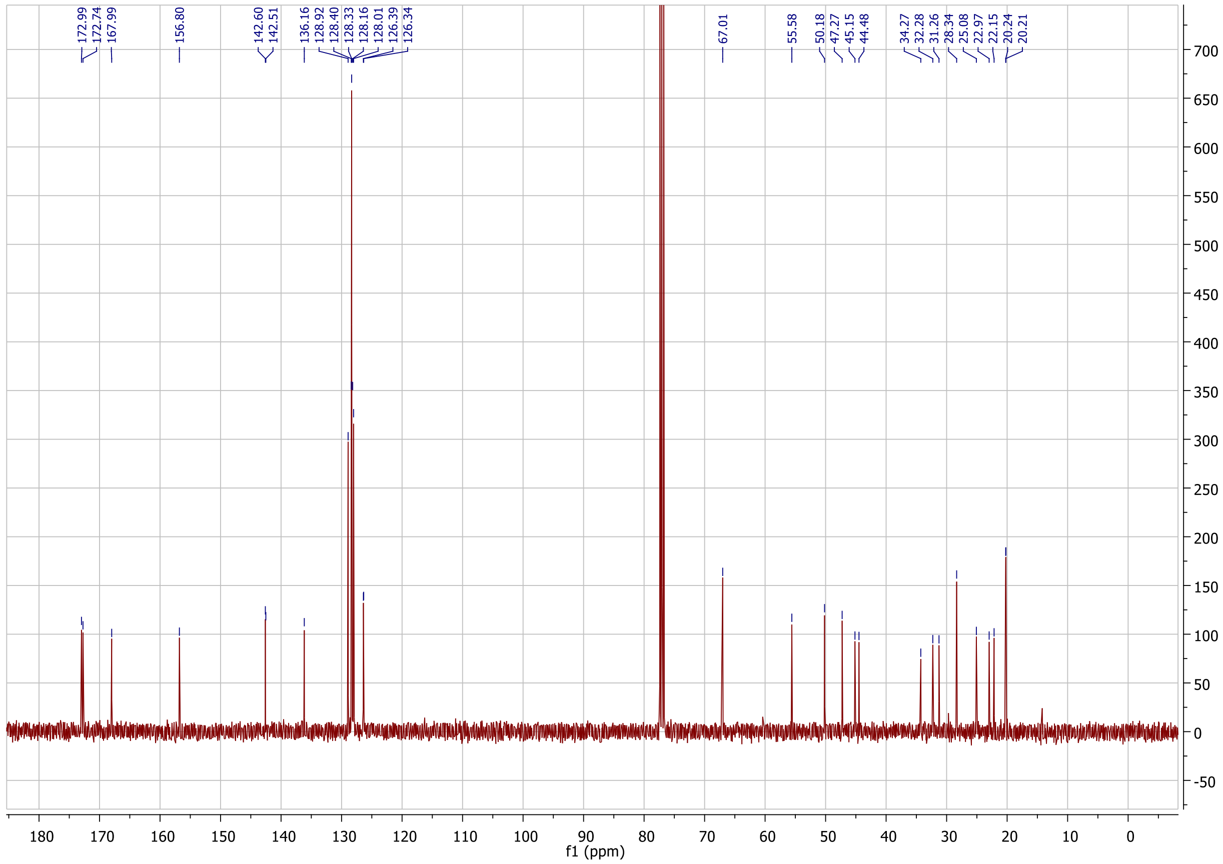

-L.G.S. - Laboratorio Grandi Strumenti - Display Report

Analysis Name	av ac176a.d	Acquisition Date	02/02/22 14:07:38	Operator	Walter Panzeri
Sample Name		Method	Copy of $opt0922.MS	Instrument	esquire3000plus
Comment	1 mg/ml dil 1:100 MeOH<br>Richiedente: Caramiello				

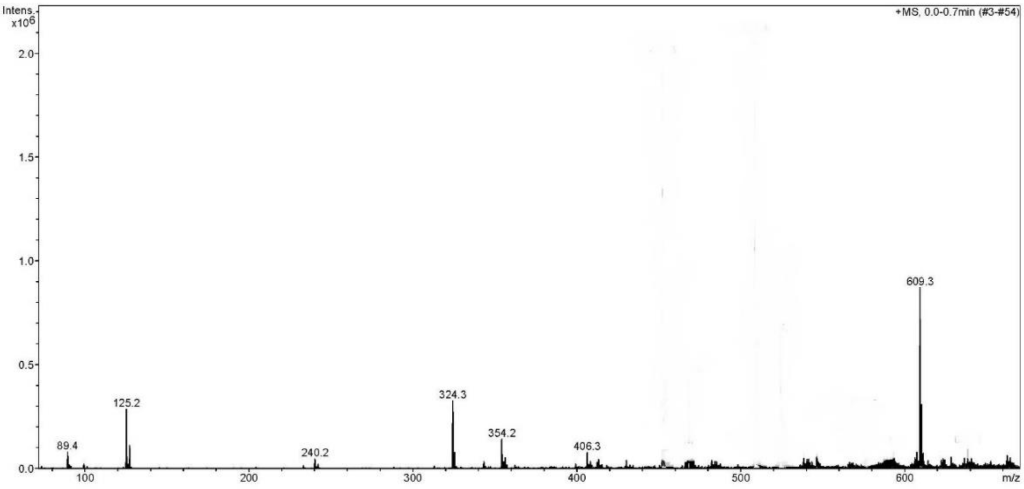

Bruker Daltonics DataAnalysis 3.1 printed: 02/02/22 14:09:42 Page 1 of 1

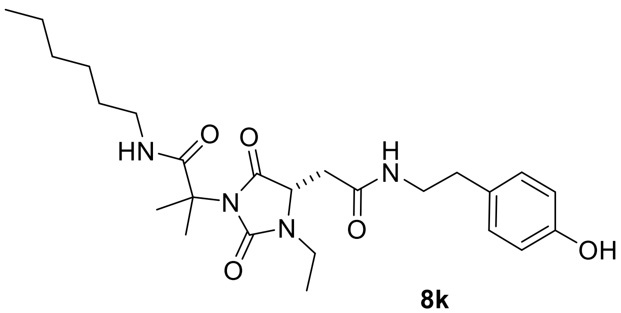

8k

^{1}H-NMR (400 MHz, CD_3OD)

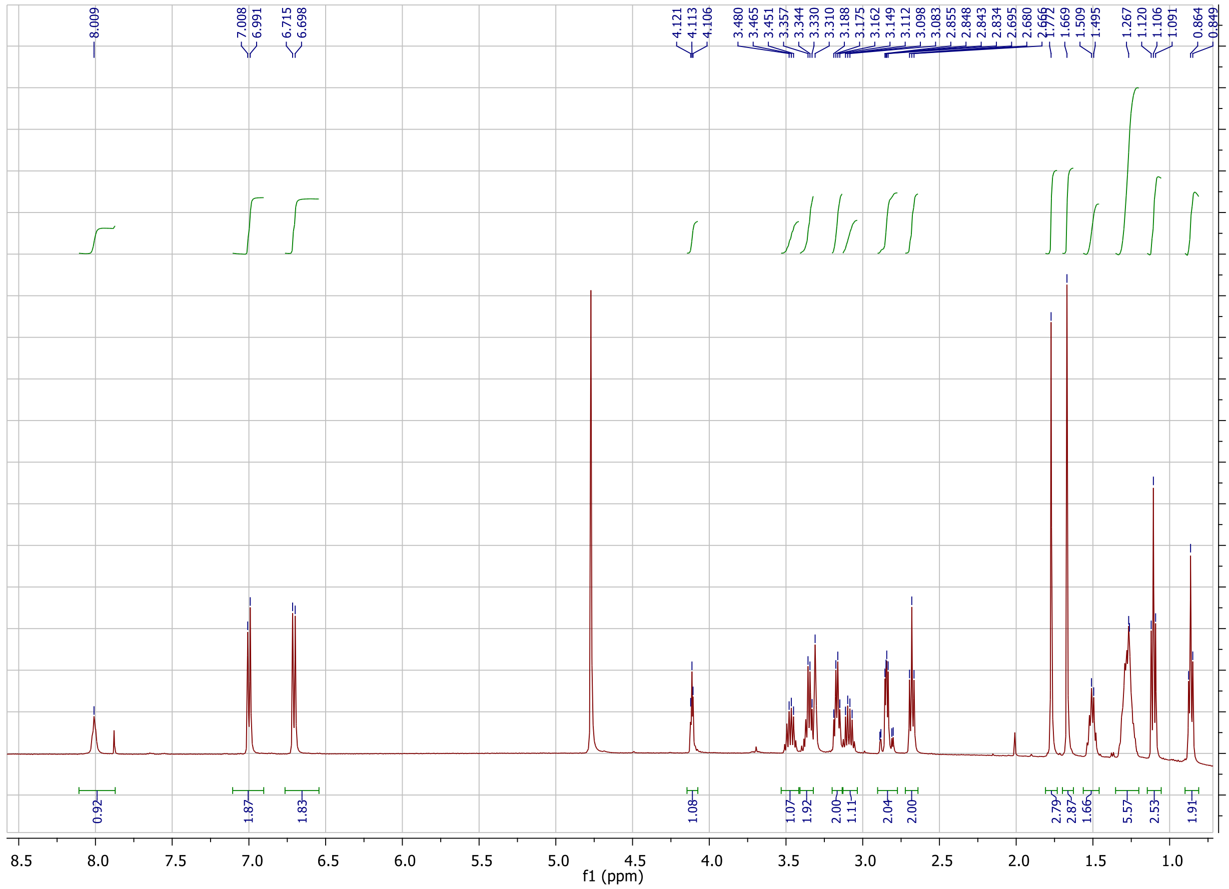

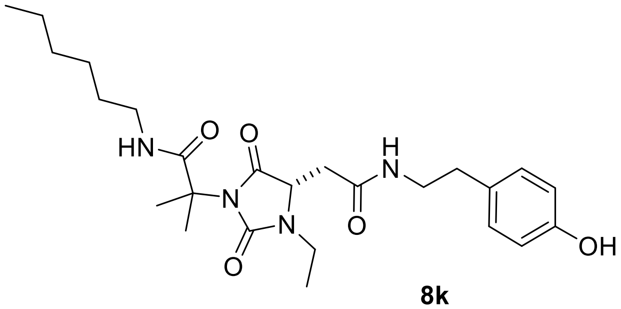

8k

$^{13}C\{^{1}H\}$-NMR (101 MHz, CD_3OD)

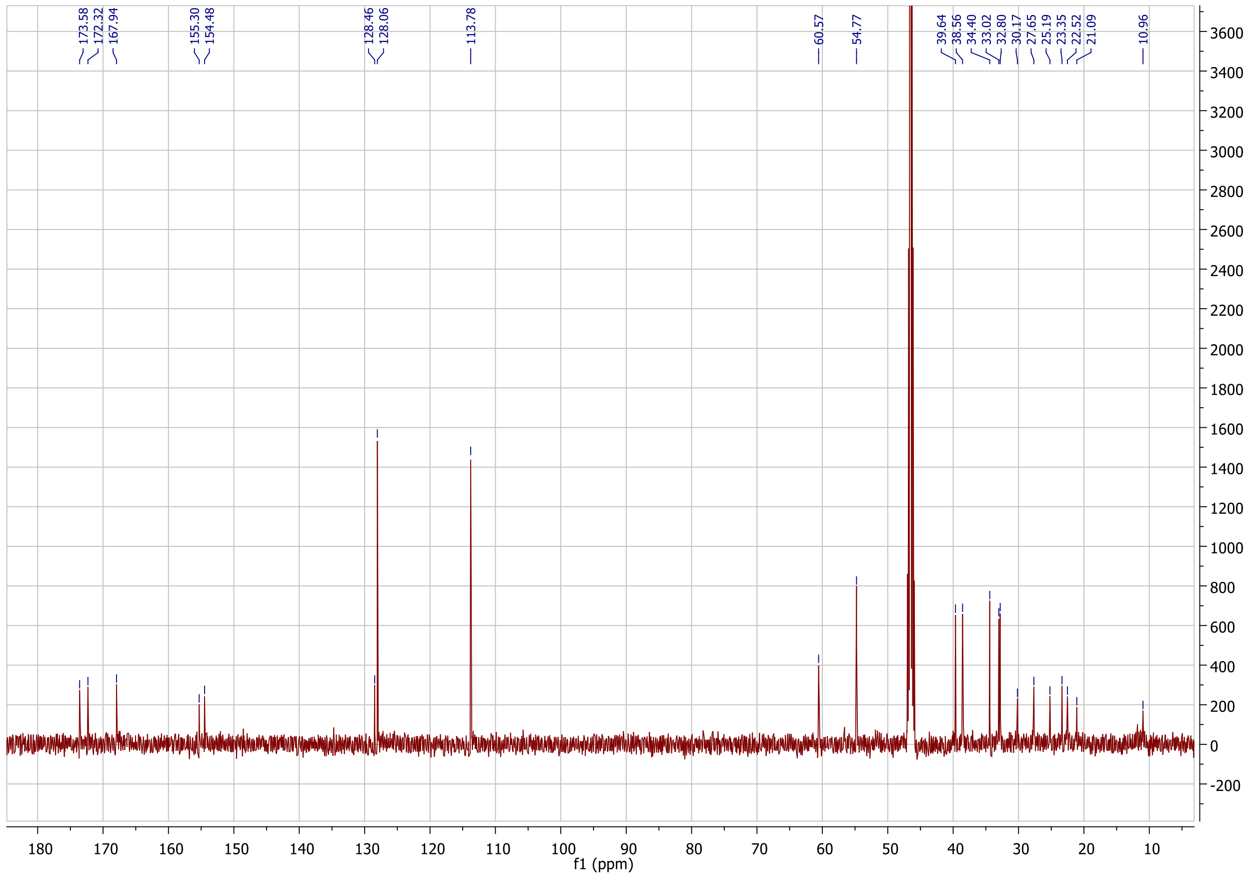

-L.G.S. - Laboratorio Grandi Strumenti - Display Report

Analysis Name	av gc22b.d	Acquisition Date	02/08/18 14:19:50	Operator	Walter Panzeri
Sample Name		Method	Copy of _01tmix_posneg lm.MS	Instrument	esquire3000plus
Comment	1 mg/ml dil 1:100 CH3CN<br>Richiedente: Cristina				

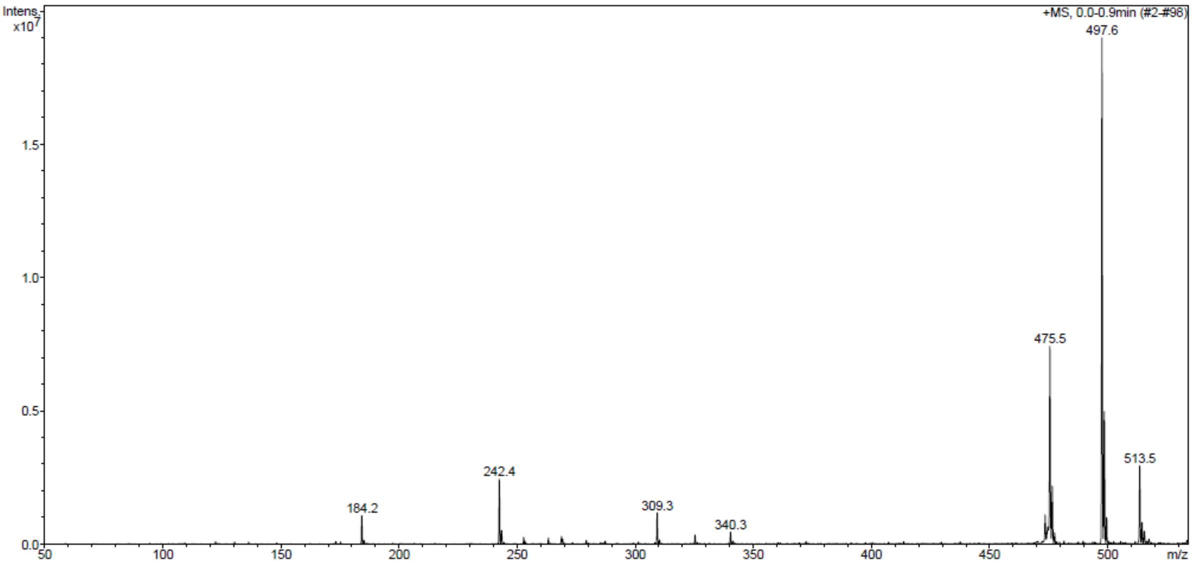

Bruker Daltonics DataAnalysis 3.1 printed: 02/08/18 14:21:50 Page 1 of 1

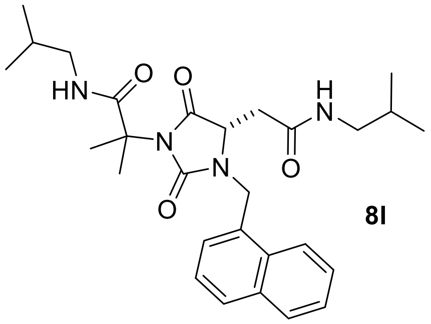

8l

^{1}H-NMR (400 MHz, CD_3OD)

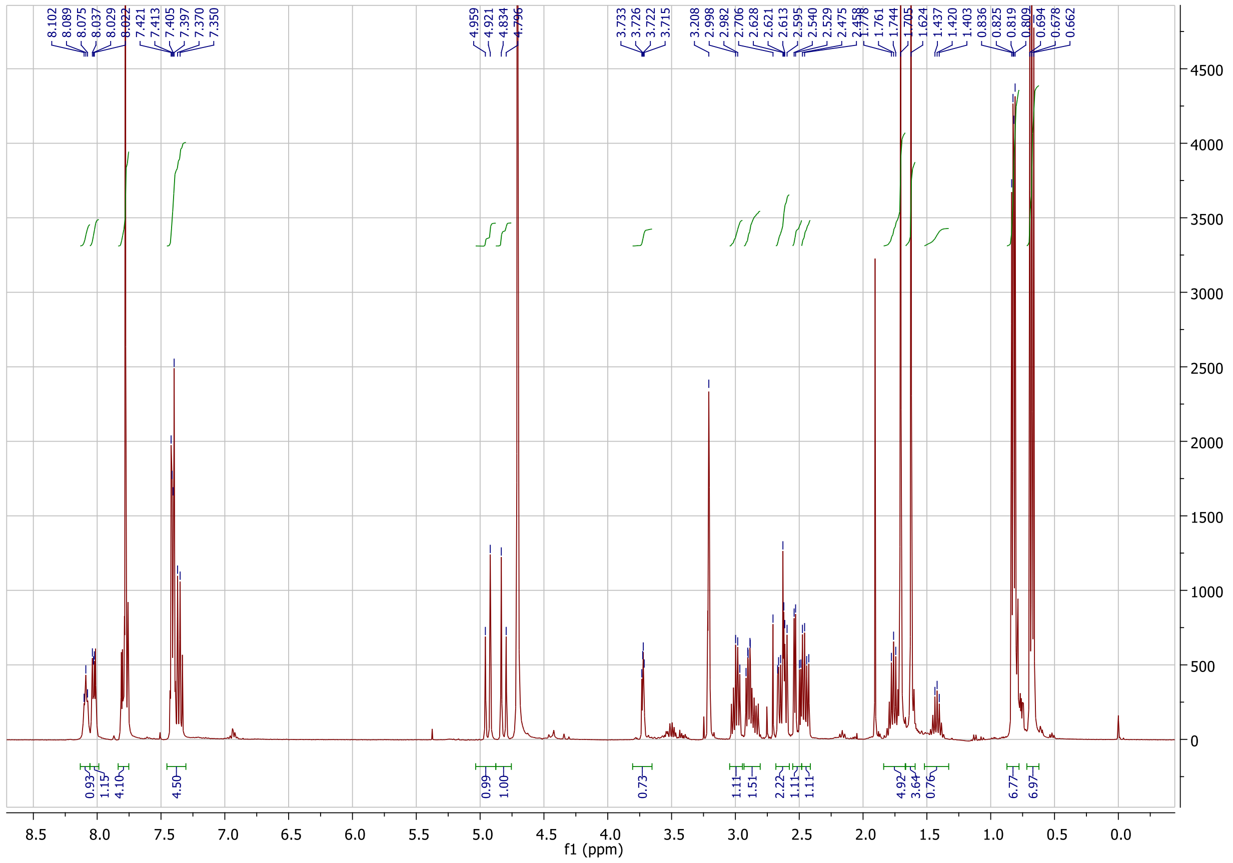

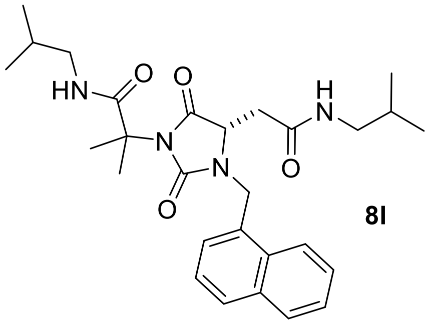

8l

$^{13}C\{^{1}H\}$-NMR (101 MHz, CD_3OD)

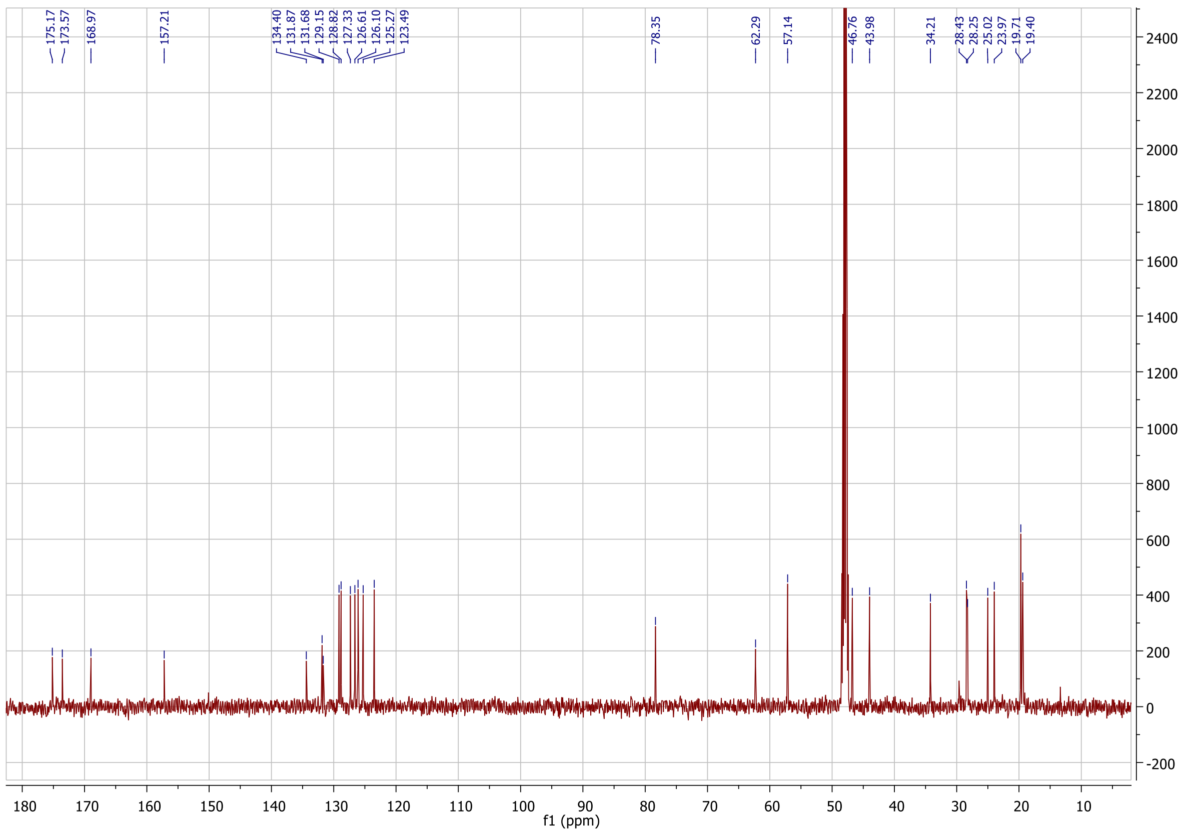

-L.G.S. - Laboratorio Grandi Strumenti - Display Report

Analysis Name	av gc62.d	Acquisition Date	06/13/18 10:46:23	Operator	Walter Panzeri
Sample Name		Method	Copy of _01tmix_posneg lm.MS	Instrument	esquire3000plus
Comment	1 mg/ml dil 1:100 MeOH<br>Richiedente: Cristina				

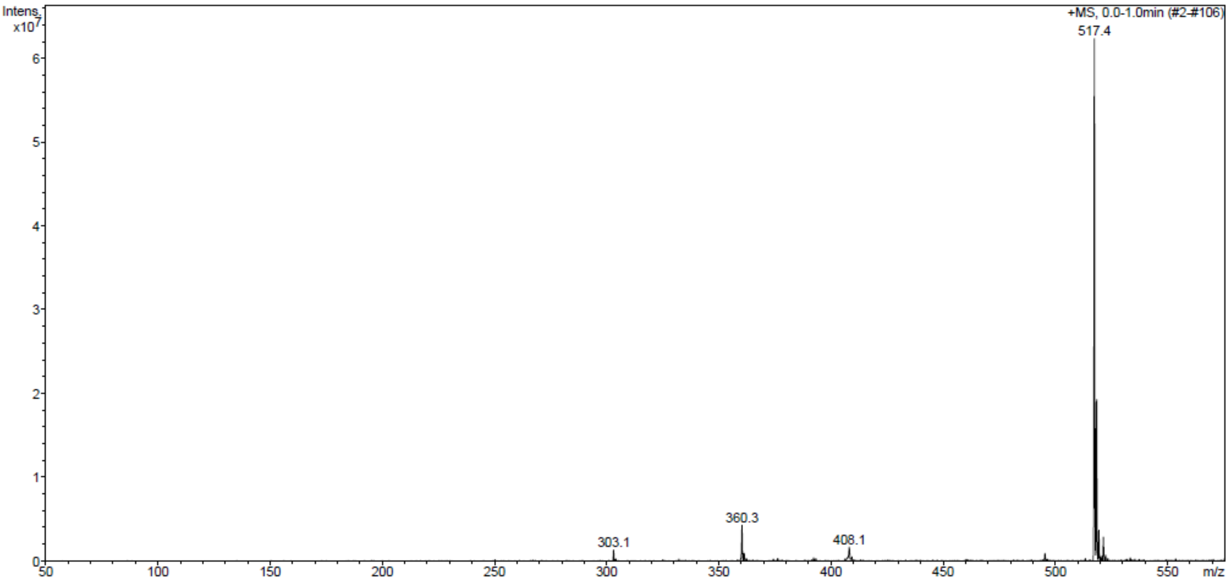

Bruker Daltonics DataAnalysis 3.1 printed: 06/13/18 10:47:56 Page 1 of 1

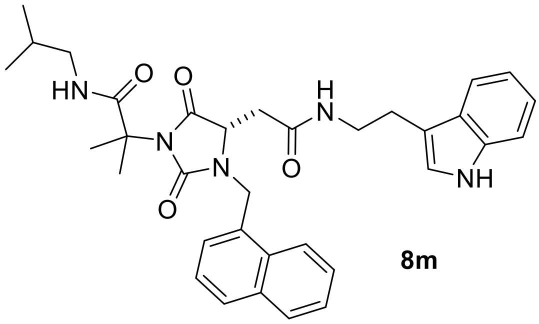

8m

^{1}H-NMR (400 MHz, CD_3OD)

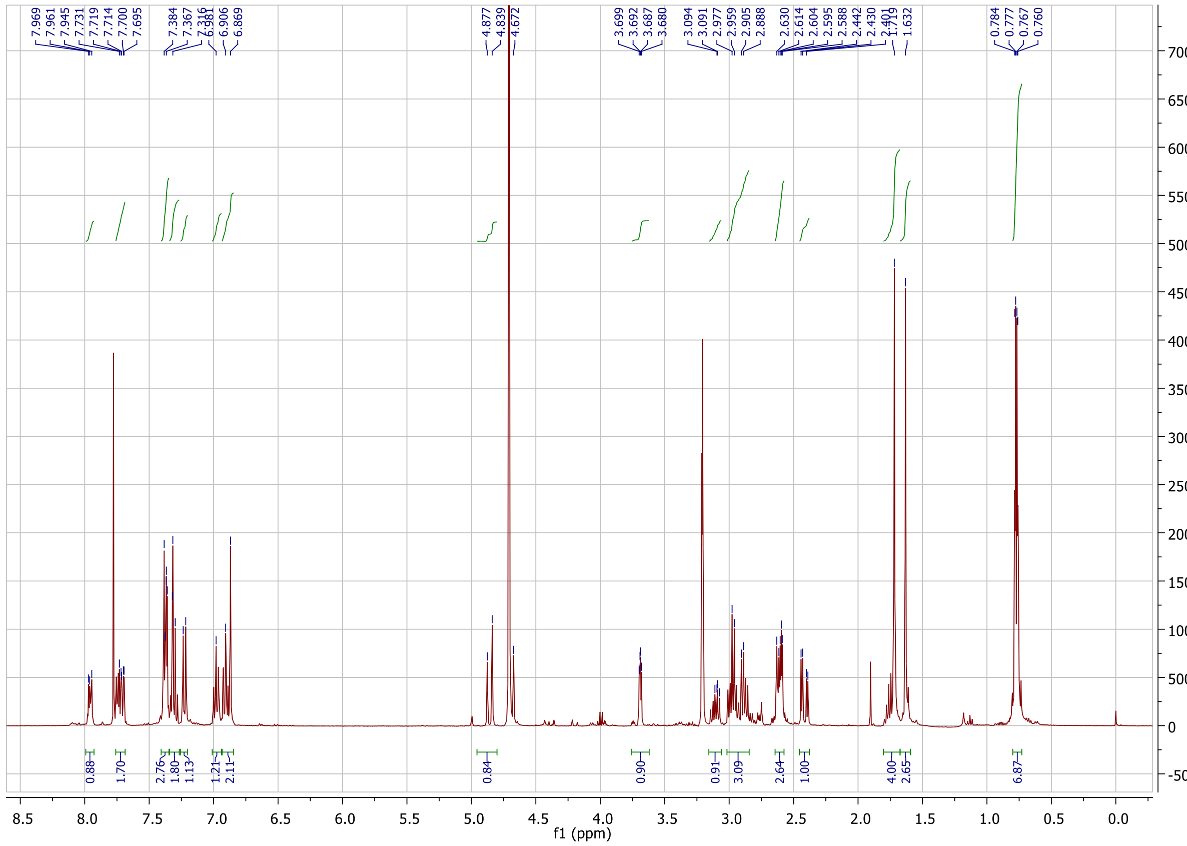

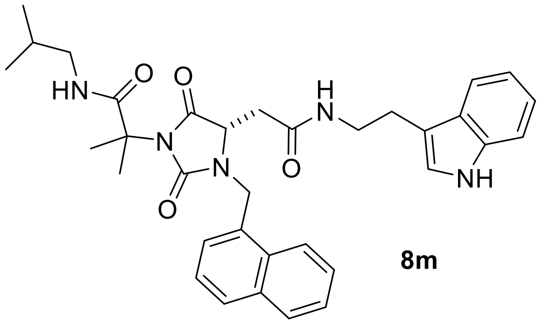

8m

$^{13}C\{^1H\}$-NMR (101 MHz, CD_3OD)

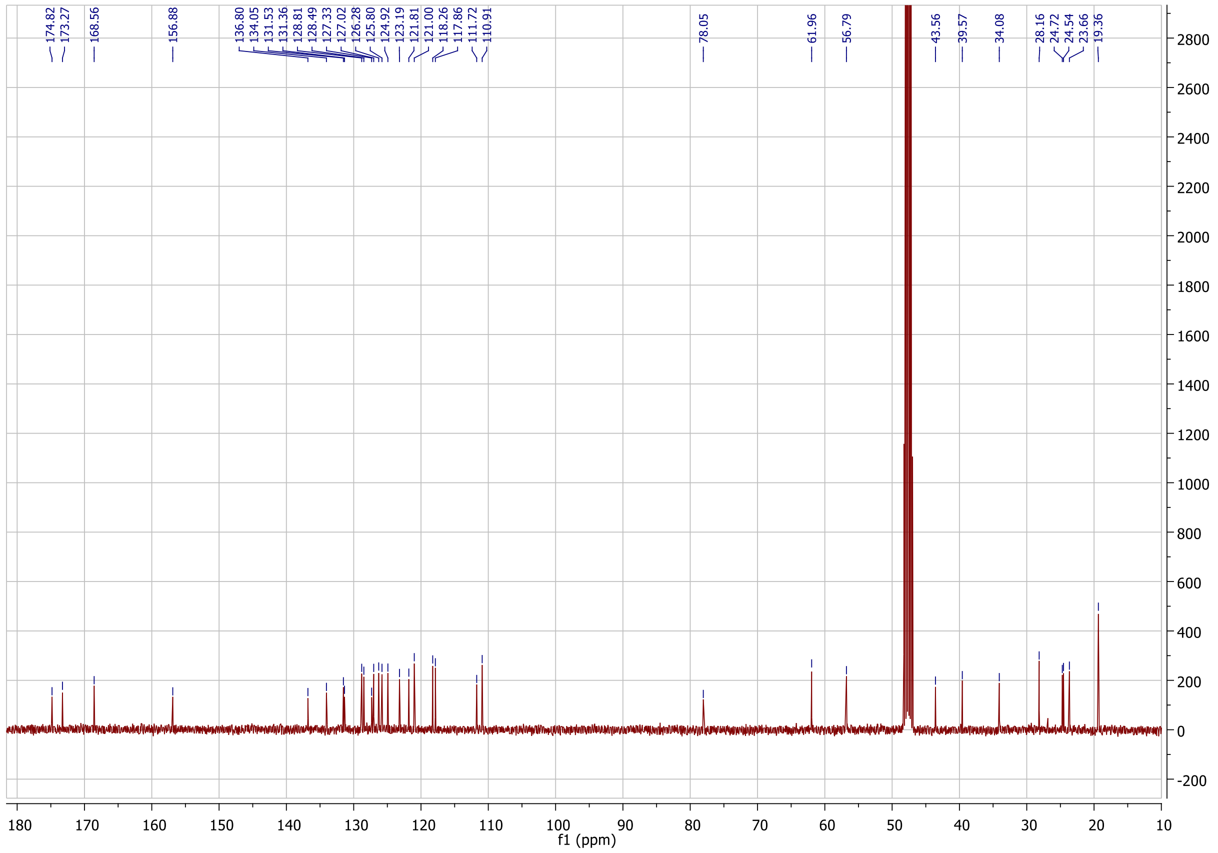

-L.G.S. - Laboratorio Grandi Strumenti - Display Report

Analysis Name	k ms1671.d	Acquisition Date	03/28/18 11:55:09	Operator	Walter Panzeri
Sample Name		Method	Copy of _01tmix_posneg Im.MS	Instrument	esquire3000plus
Comment	1 mg/ml dil 1:100 MeOH Richiedente: Sani				

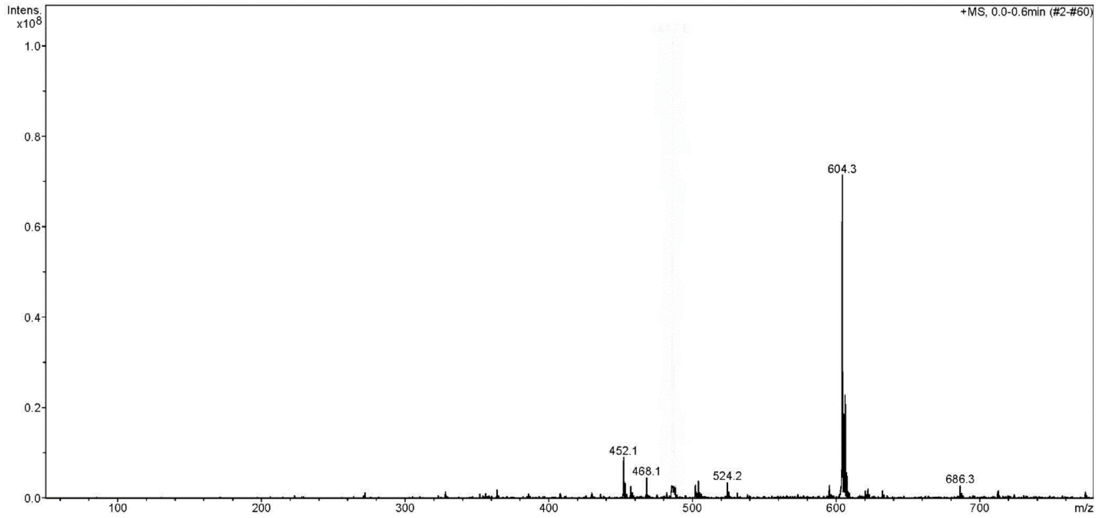

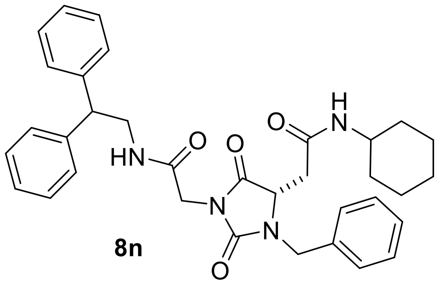

8n

^{1}H-NMR (400 MHz, $CDCl_3$)

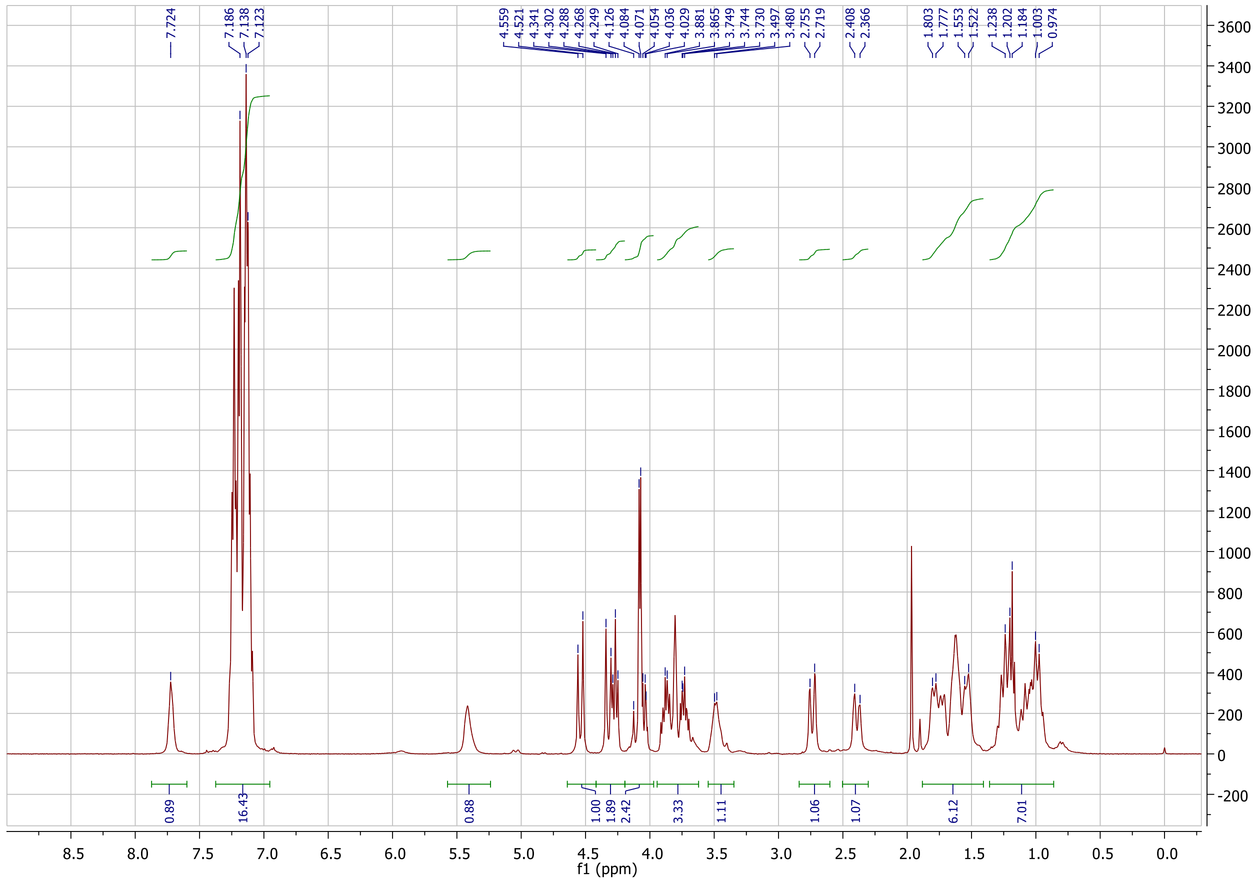

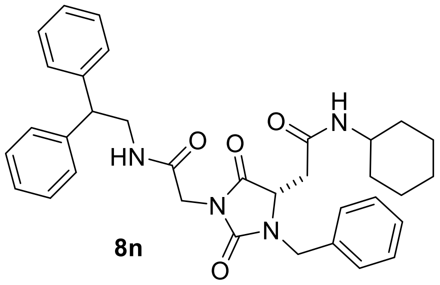

8n

$^{13}C\{^{1}H\}$-NMR (101 MHz, $CDCl_3$)

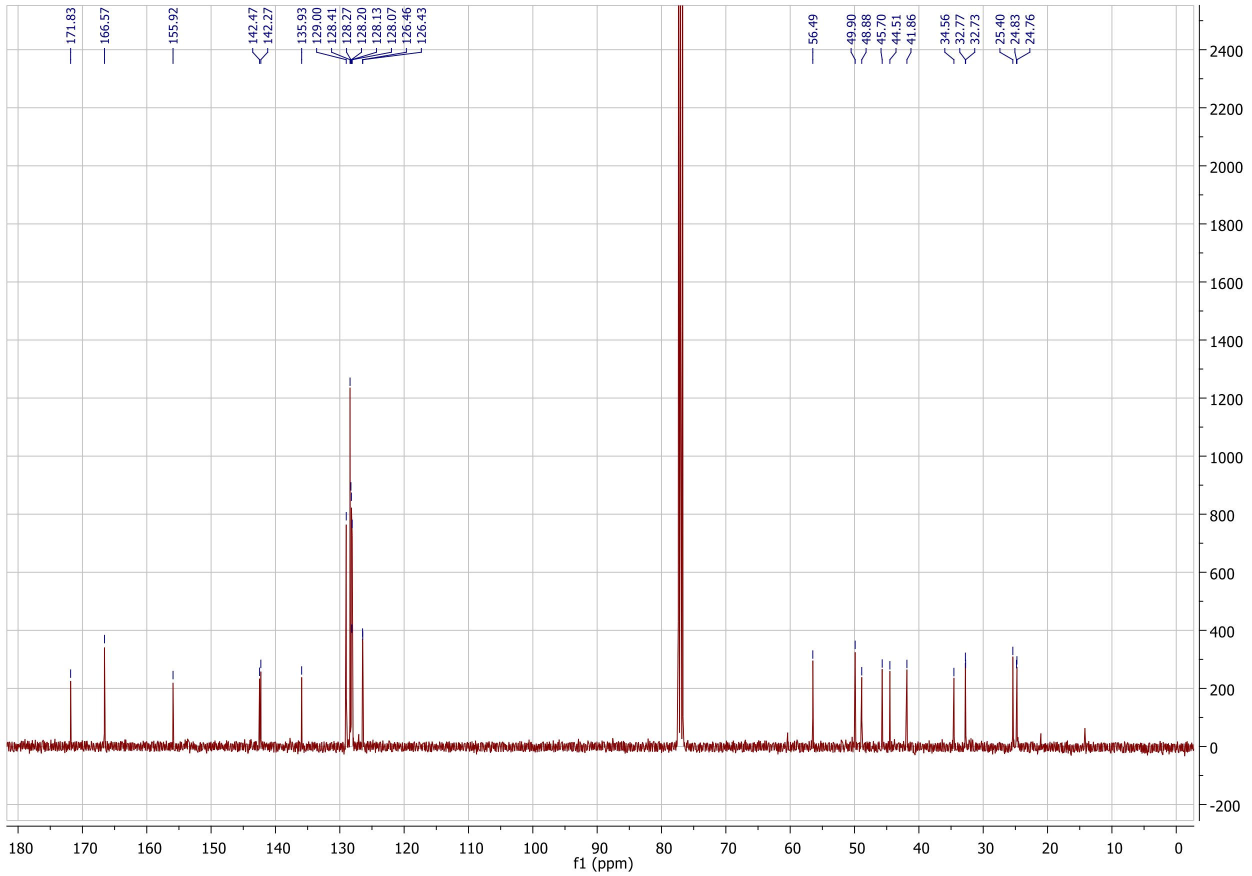

-L.G.S. - Laboratorio Grandi Strumenti - Display Report

Analysis Name	av gc40.d	Acquisition Date	04/19/18 09:25:10	Operator	Walter Panzeri
Sample Name		Method	Copy of _01tmix_posneg lm.MS	Instrument	esquire3000plus
Comment	1 mg/ml dil 1:100 MeOH<br>Richiedente: Cristina				

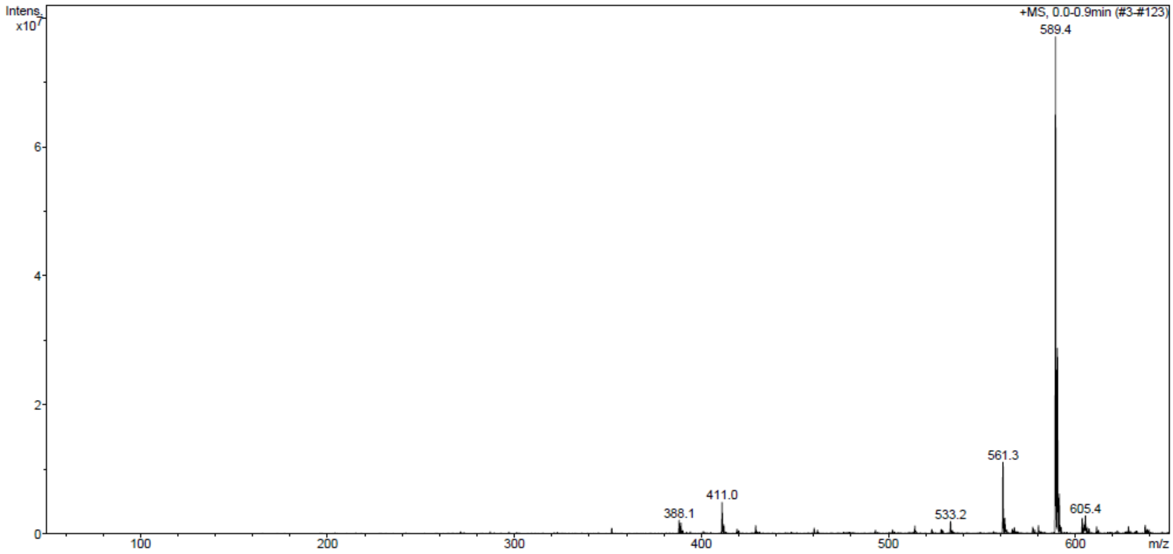

Bruker Daltonics DataAnalysis 3.1 printed: 04/19/18 09:26:20 Page 1 of 1

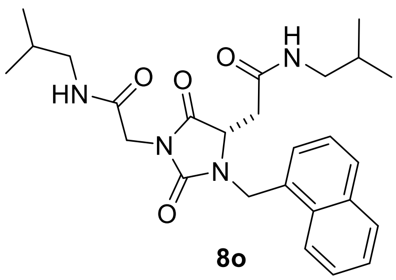

8o

^{1}H-NMR (101 MHz, CD_3OD)

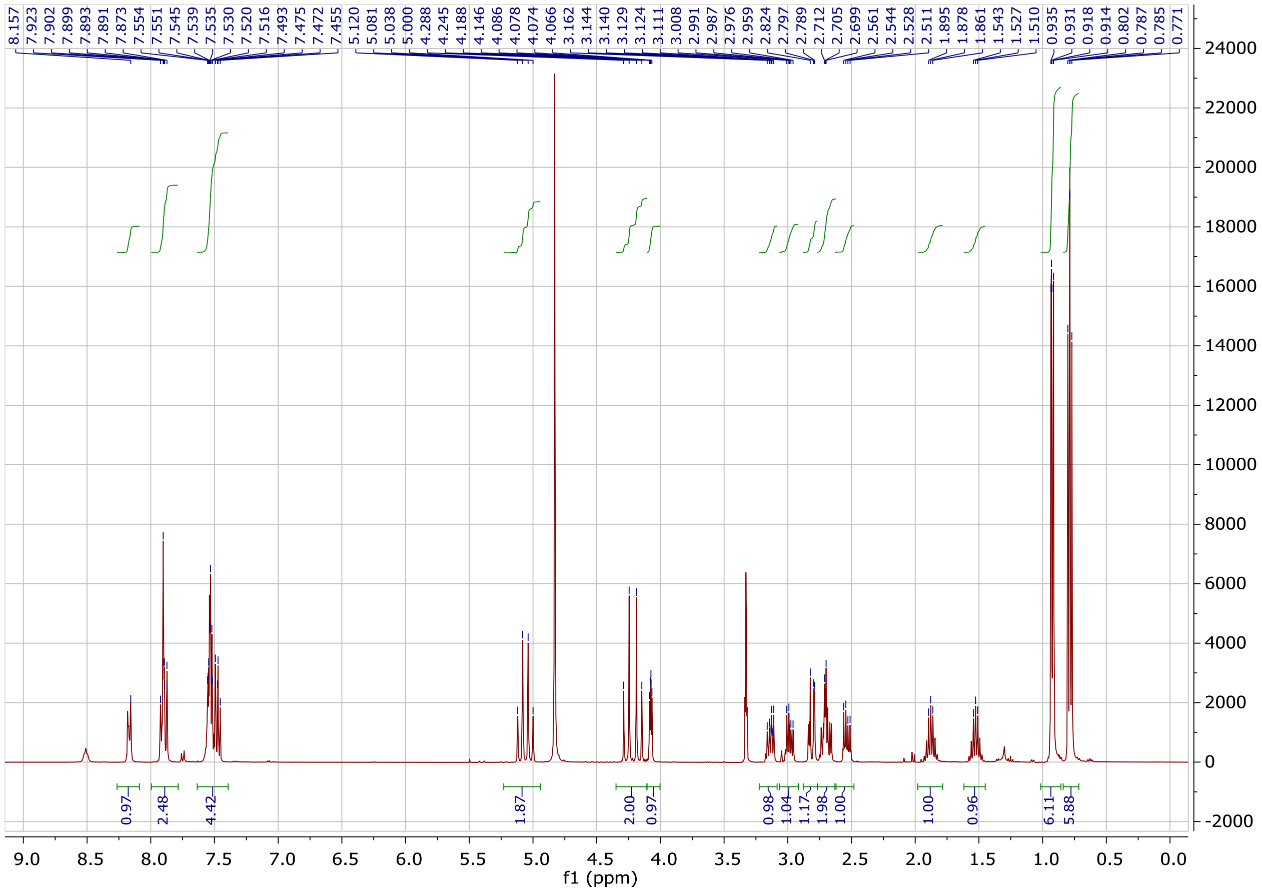

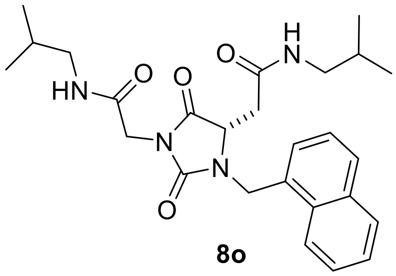

8o

$^{13}C\{^{1}H\}$-NMR (101 MHz, CD_3OD)

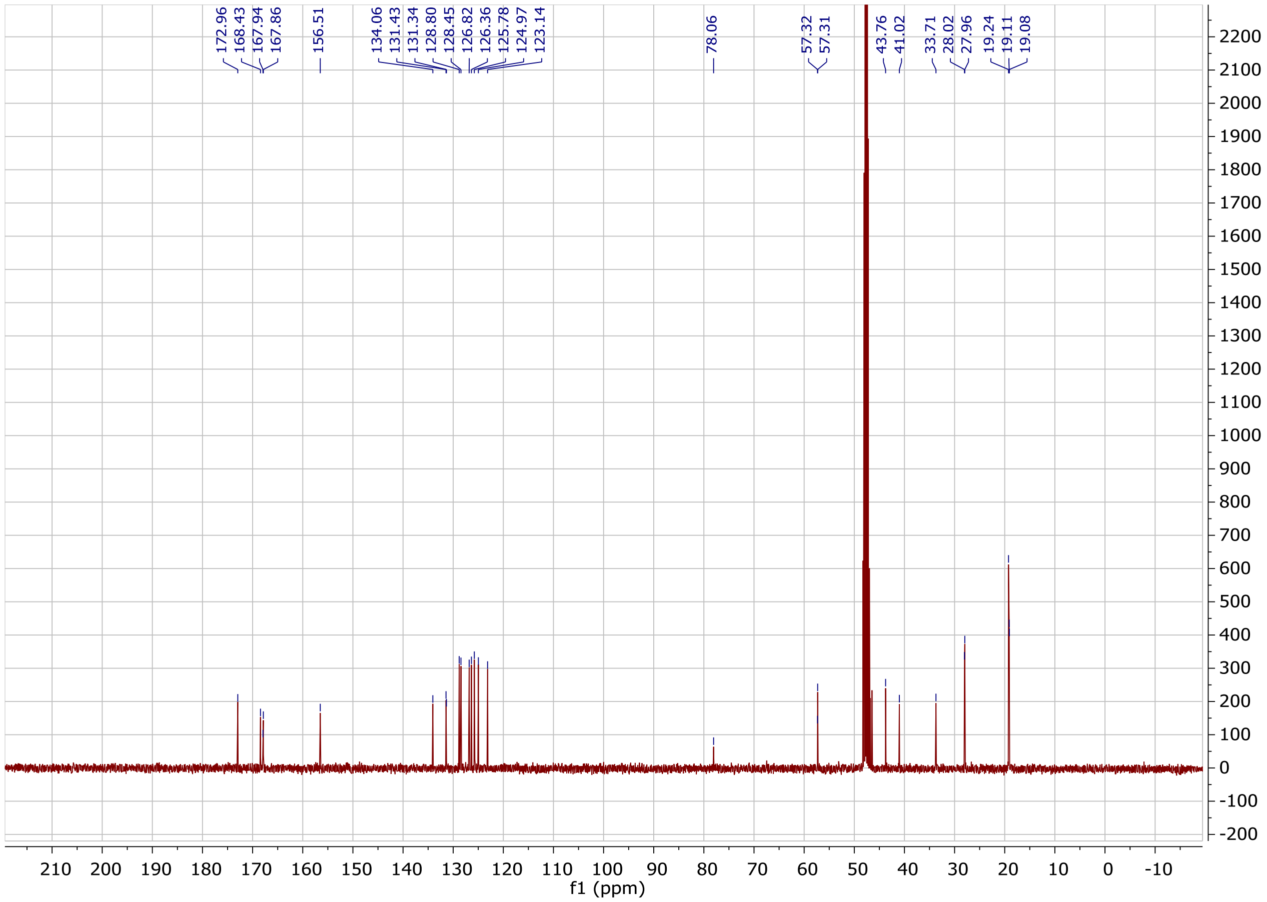

Analysis Name	av ac065.d	Acquisition Date	07/20/20 14:27:00	Operator	Walter Panzeri
Sample Name		Method	Copy of _01tmix_posneg lm.MS	Instrument	esquire3000plus
Comment	1mg/ml dil 1:100 MeOH<br>Richiedente: Caramiello				

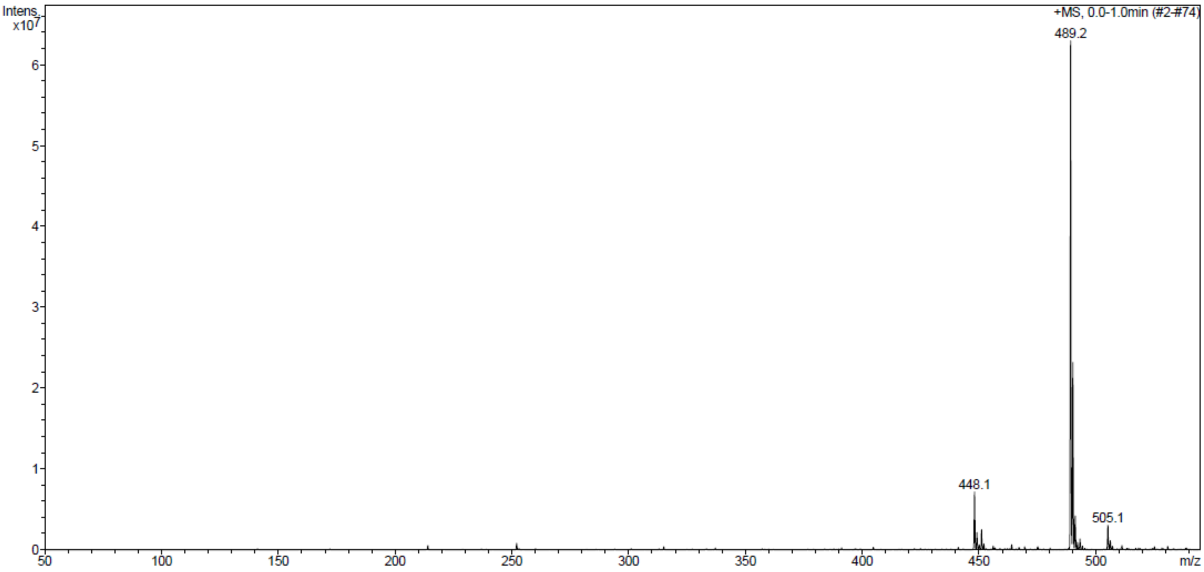

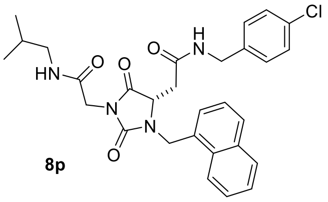

8p

^{1}H NMR and COSY in $CDCl_3$ (400 MHz)

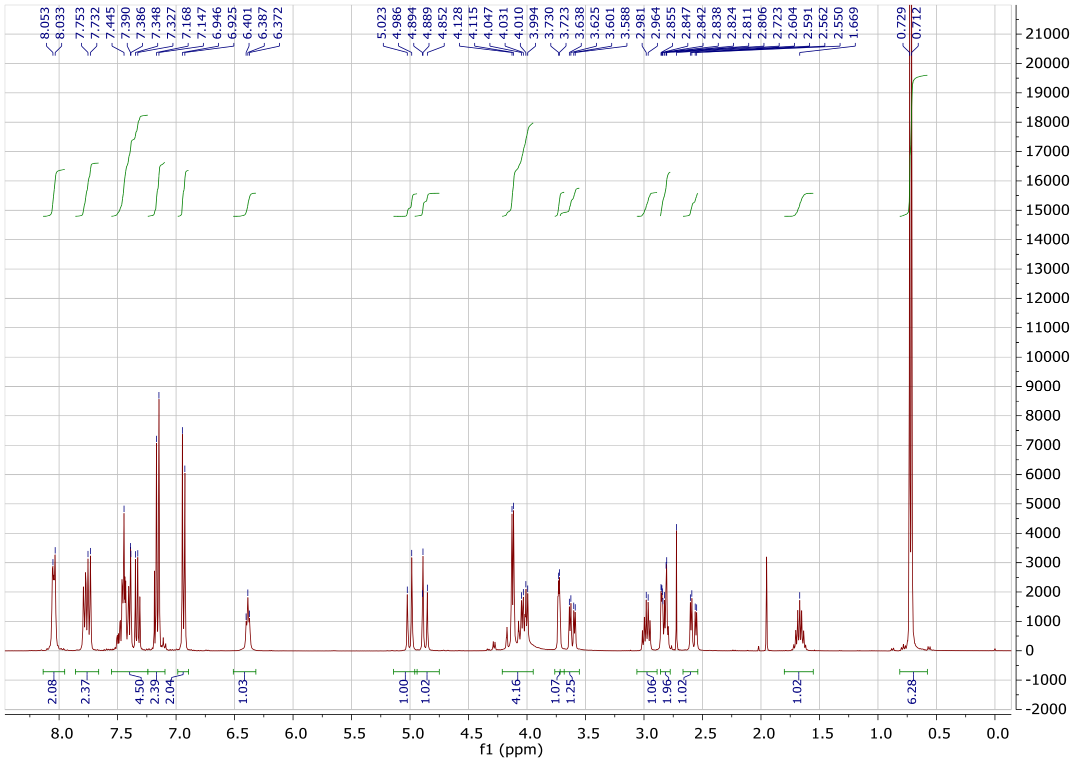

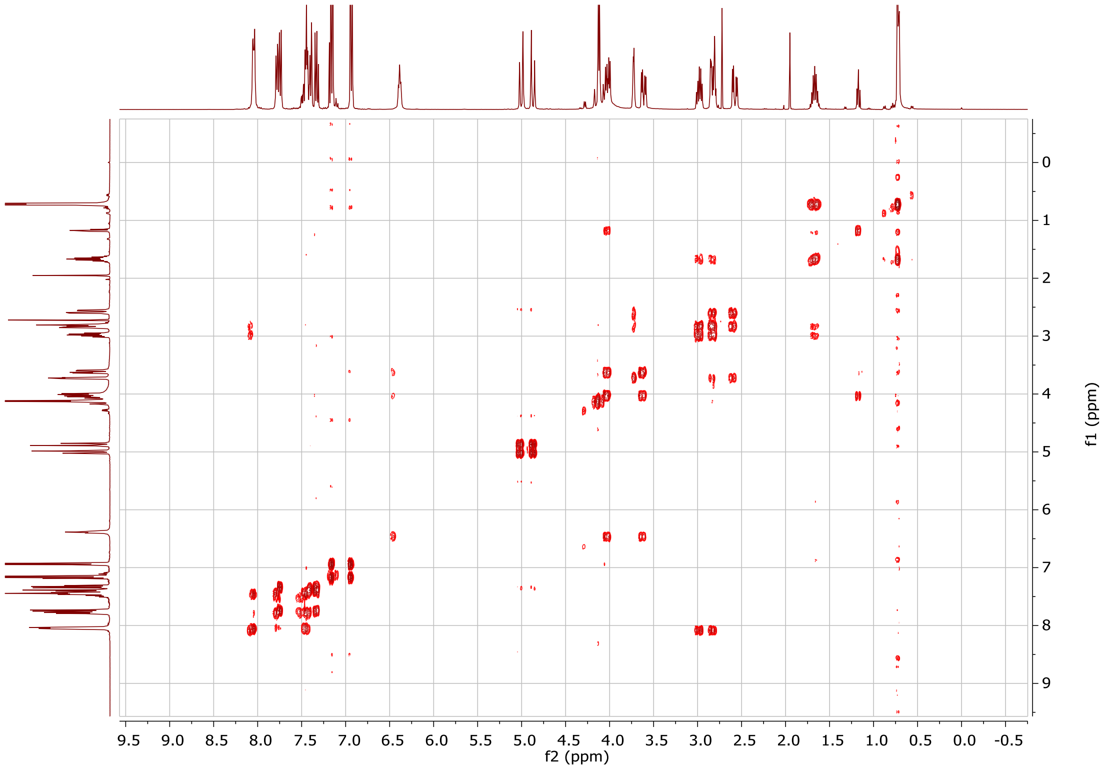

^{1}H NMR (400 MH, dmso-d_6)

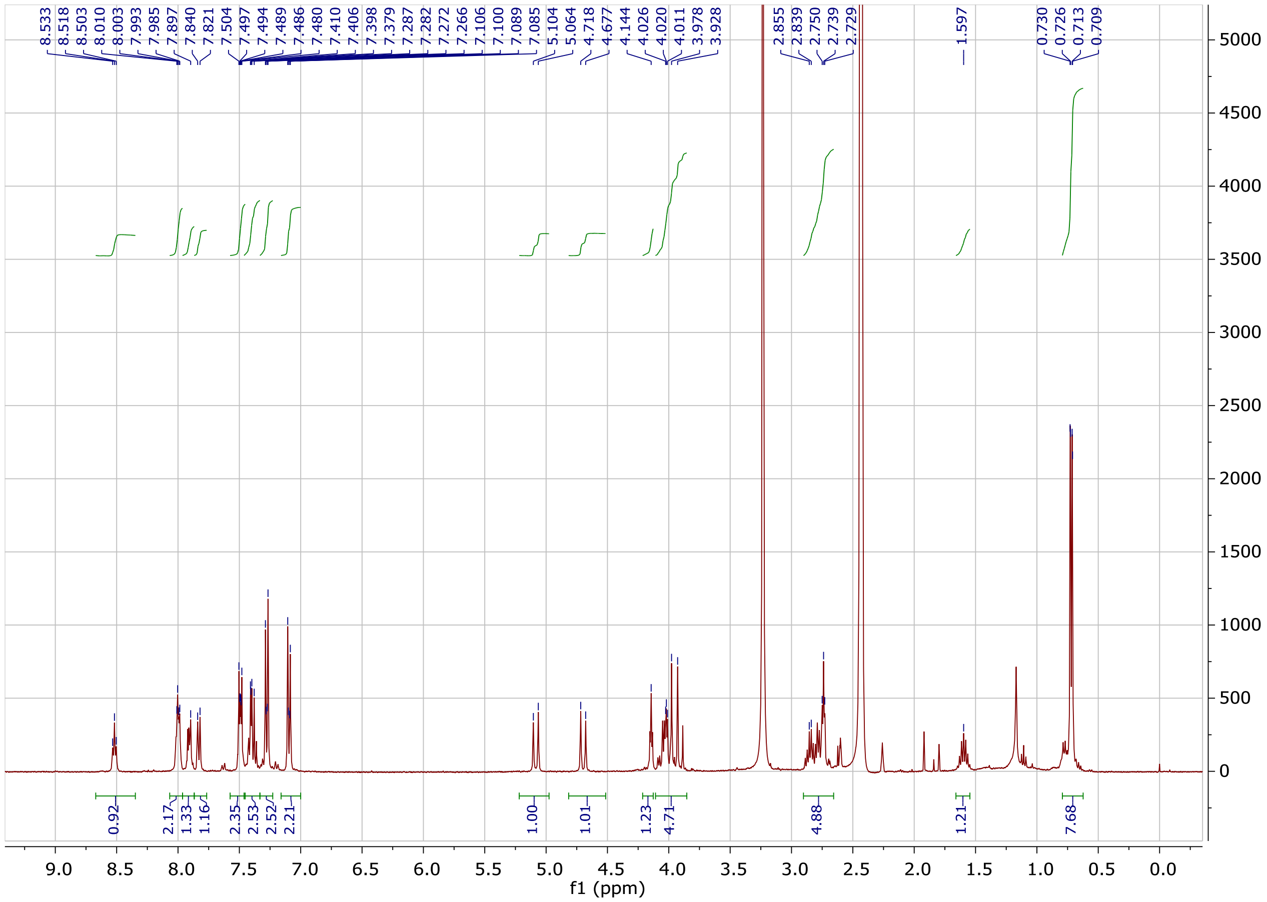

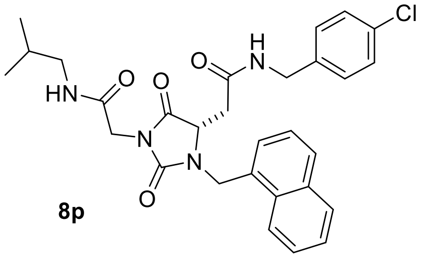

8p

$^{13}C\{^{1}H\}$-NMR (101 MHz, $CDCl_3$)

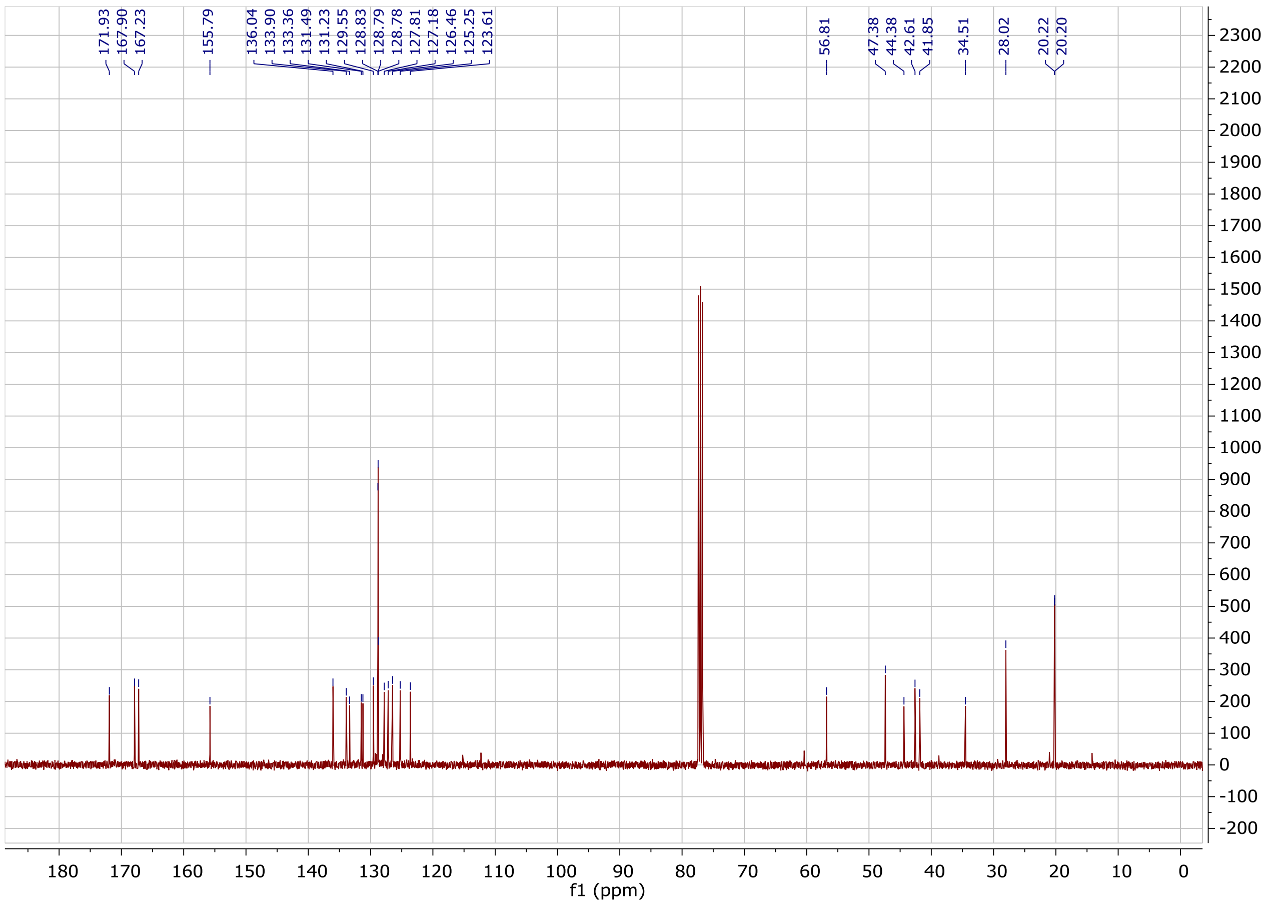

-L.G.S. - Laboratorio Grandi Strumenti - Display Report

Analysis Name	av ac073.d	Acquisition Date	09/08/20 10:52:09	Operator	Walter Panzeri
Sample Name		Method	Copy of $PW_TBLM.MS	Instrument	esquire3000plus
Comment	1 mg/ml dil 1:100 MeOH<br>Richiedente: Caramiello				

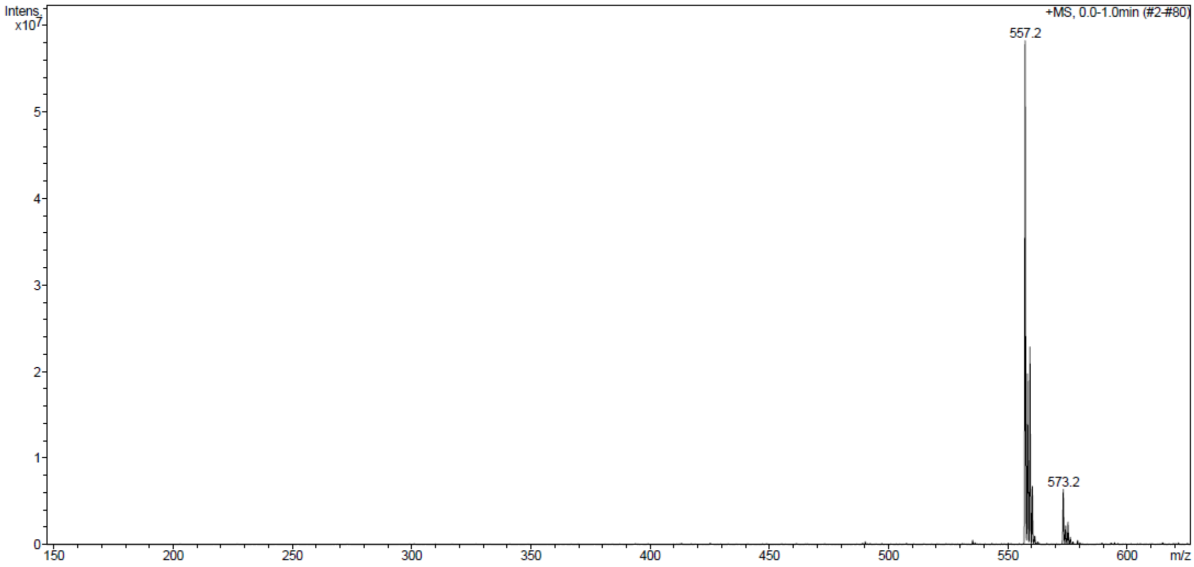

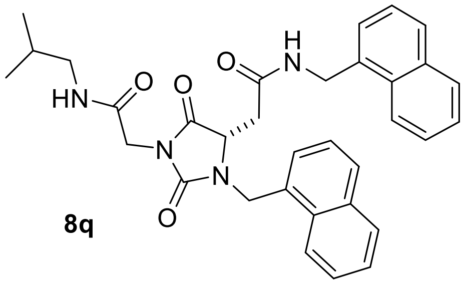

8q

^{1}H-NMR (400 MHz, CD_3OD)

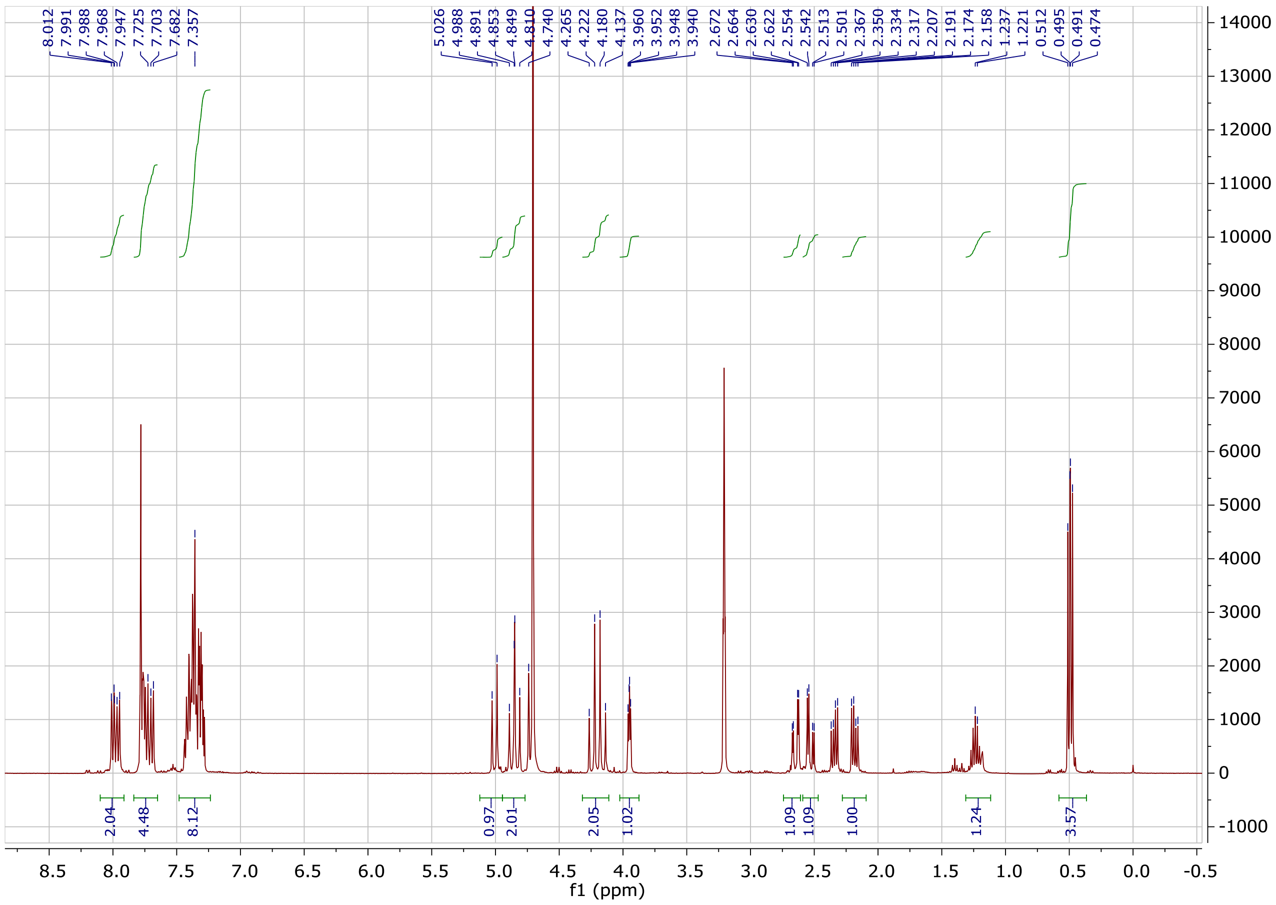

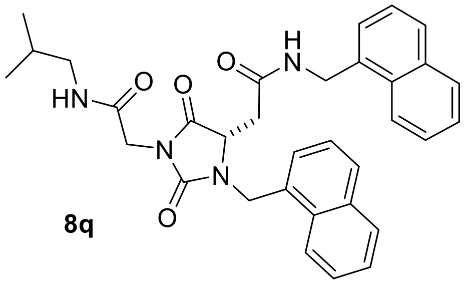

8q

$^{13}C\{^1H\}$-NMR (101 MHz, CD_3OD)

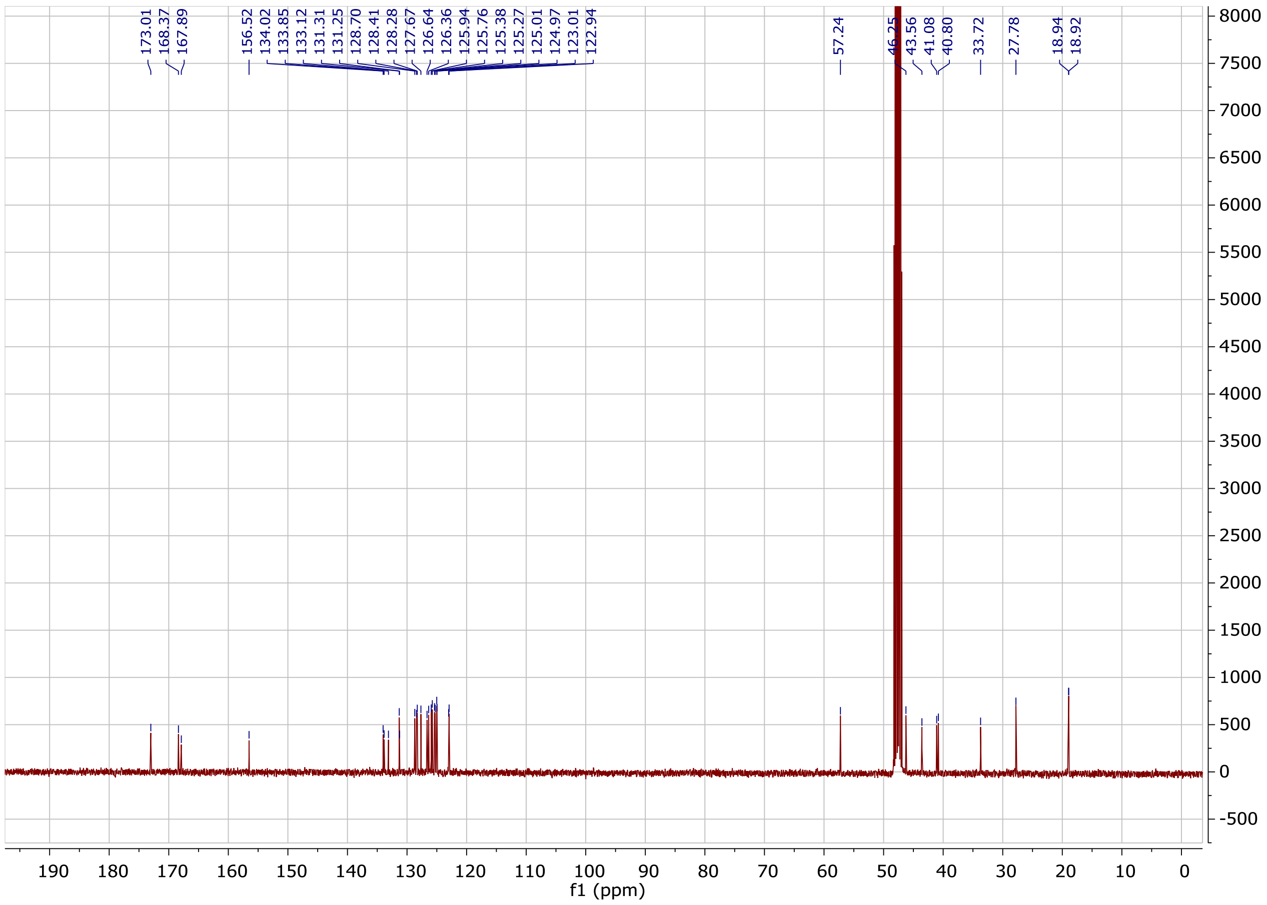

-L.G.S. - Laboratorio Grandi Strumenti - Display Report

Analysis Name	av ac074.d	Acquisition Date	09/08/20 10:45:32	Operator	Walter Panzeri
Sample Name		Method	Copy of $PW_TBLM.MS	Instrument	esquire3000plus
Comment	1 mg/ml dil 1:100 MeOH<br>Richiedente: Caramiello				

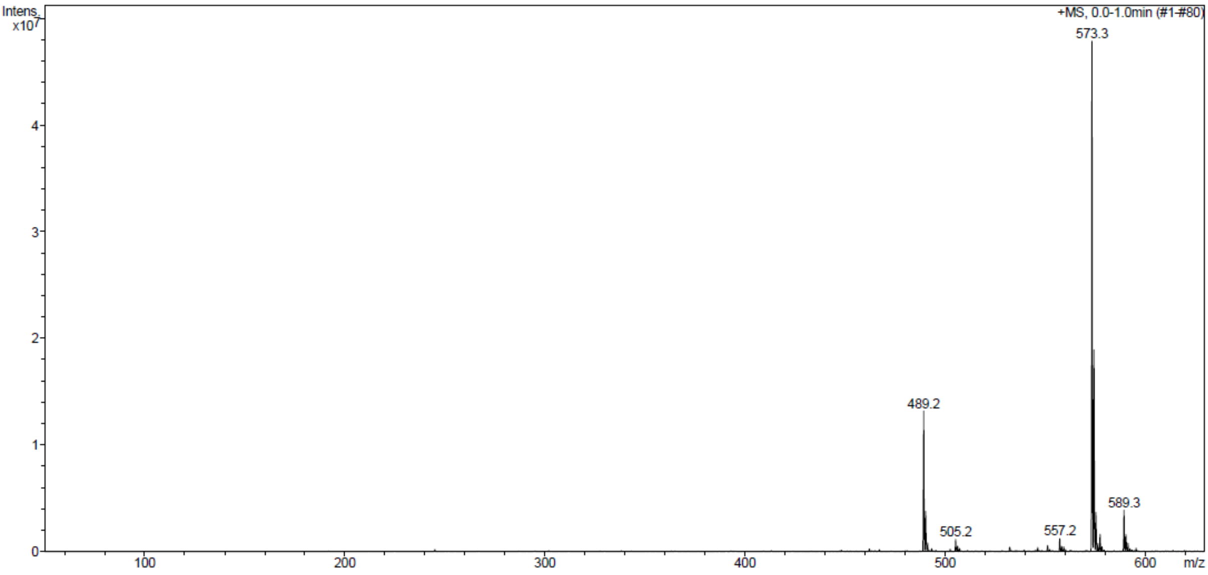

Bruker Daltonics DataAnalysis 3.1 printed: 09/08/20 10:47:17 Page 1 of 1

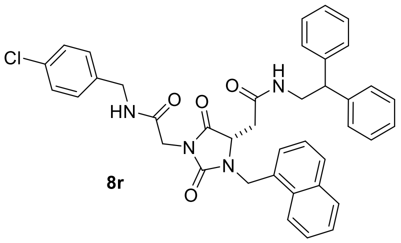

8r

^{1}H NMR and COSY in $CDCl_3$ (400 MHz)

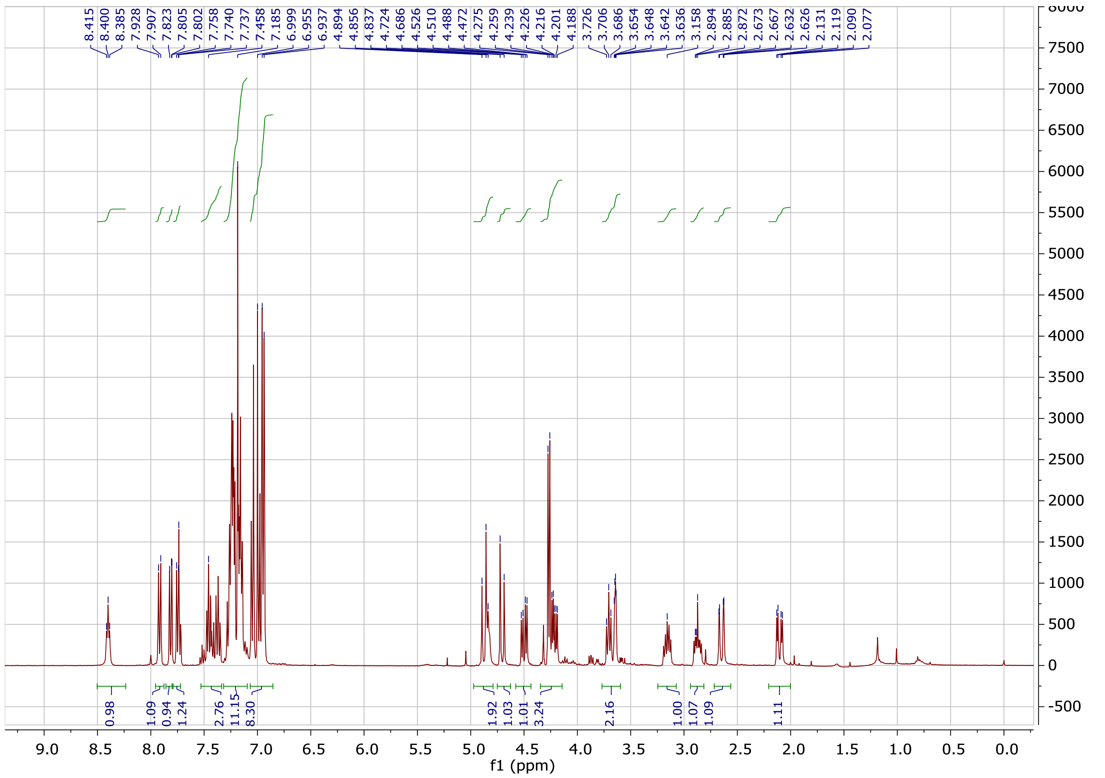

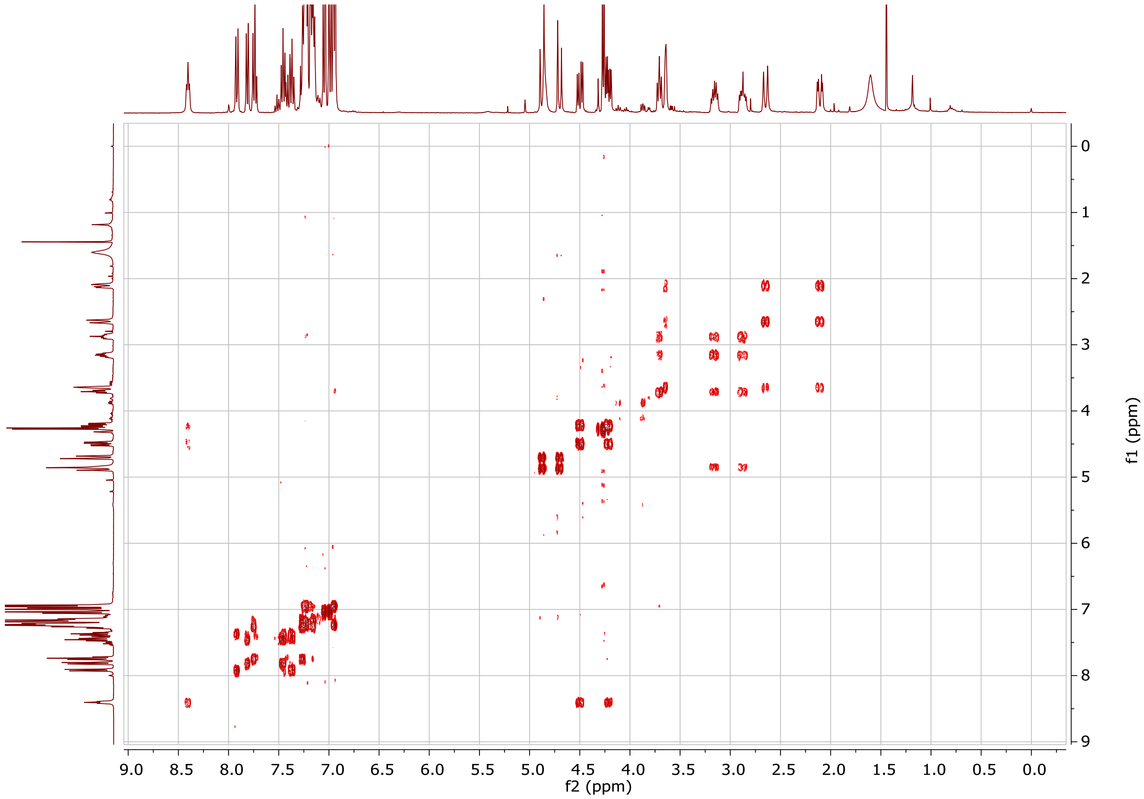

^{1}H NMR and COSY in dmso-d$_6$ (400 MHz)

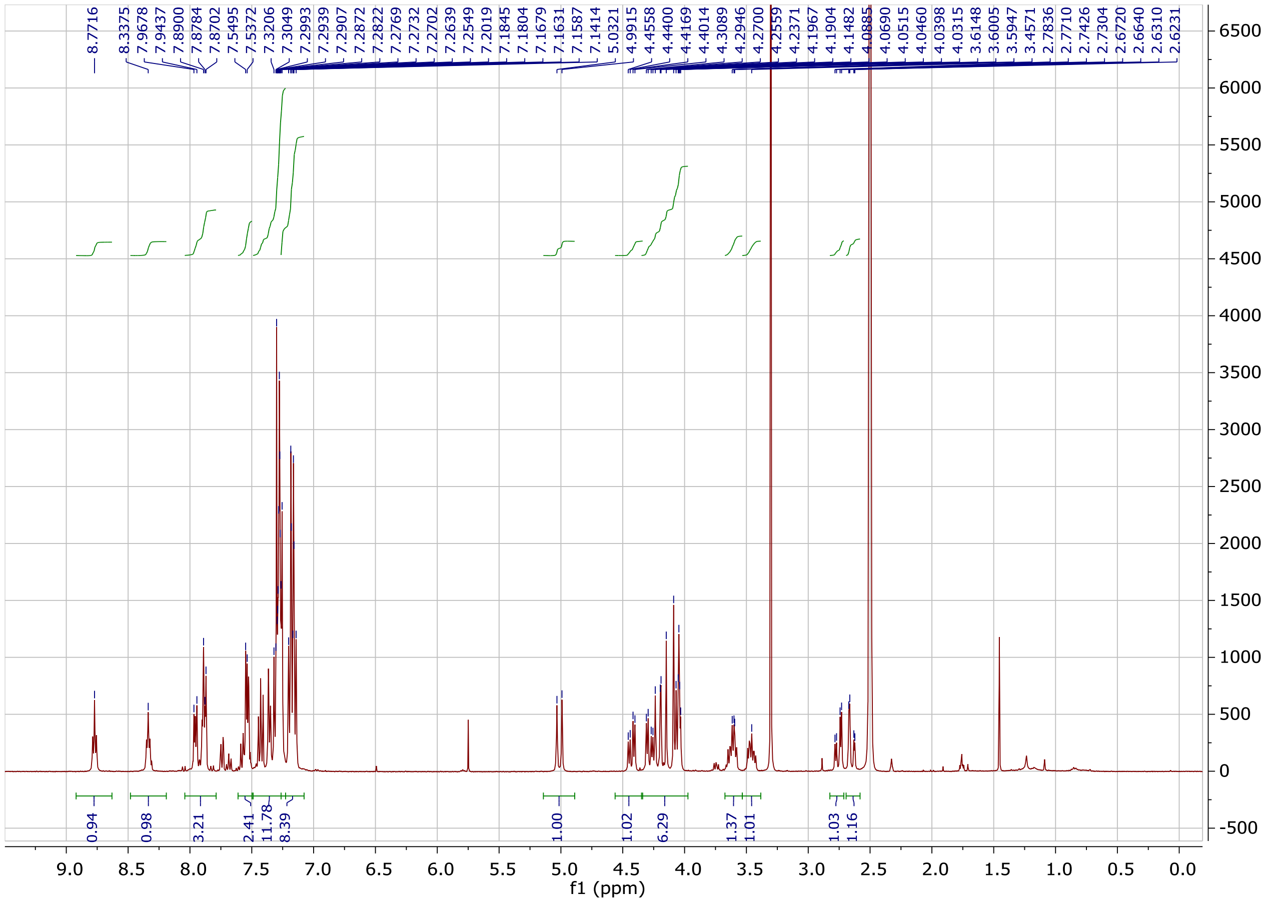

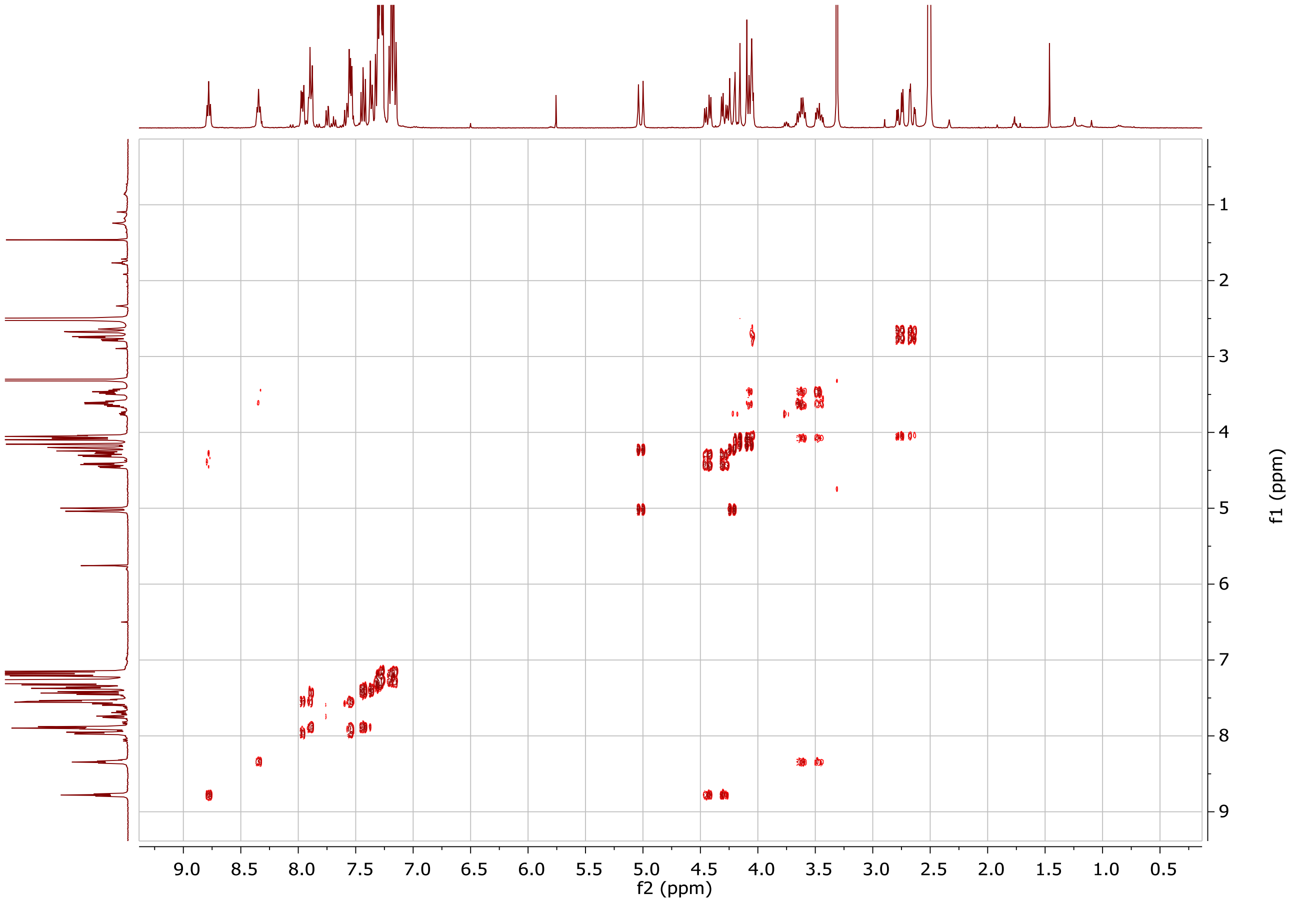

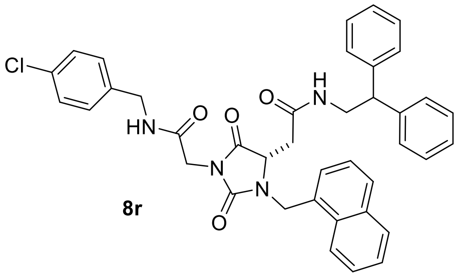

8r

$^{13}C\{^{1}H\}$-NMR (101 MHz, $CDCl_3$)

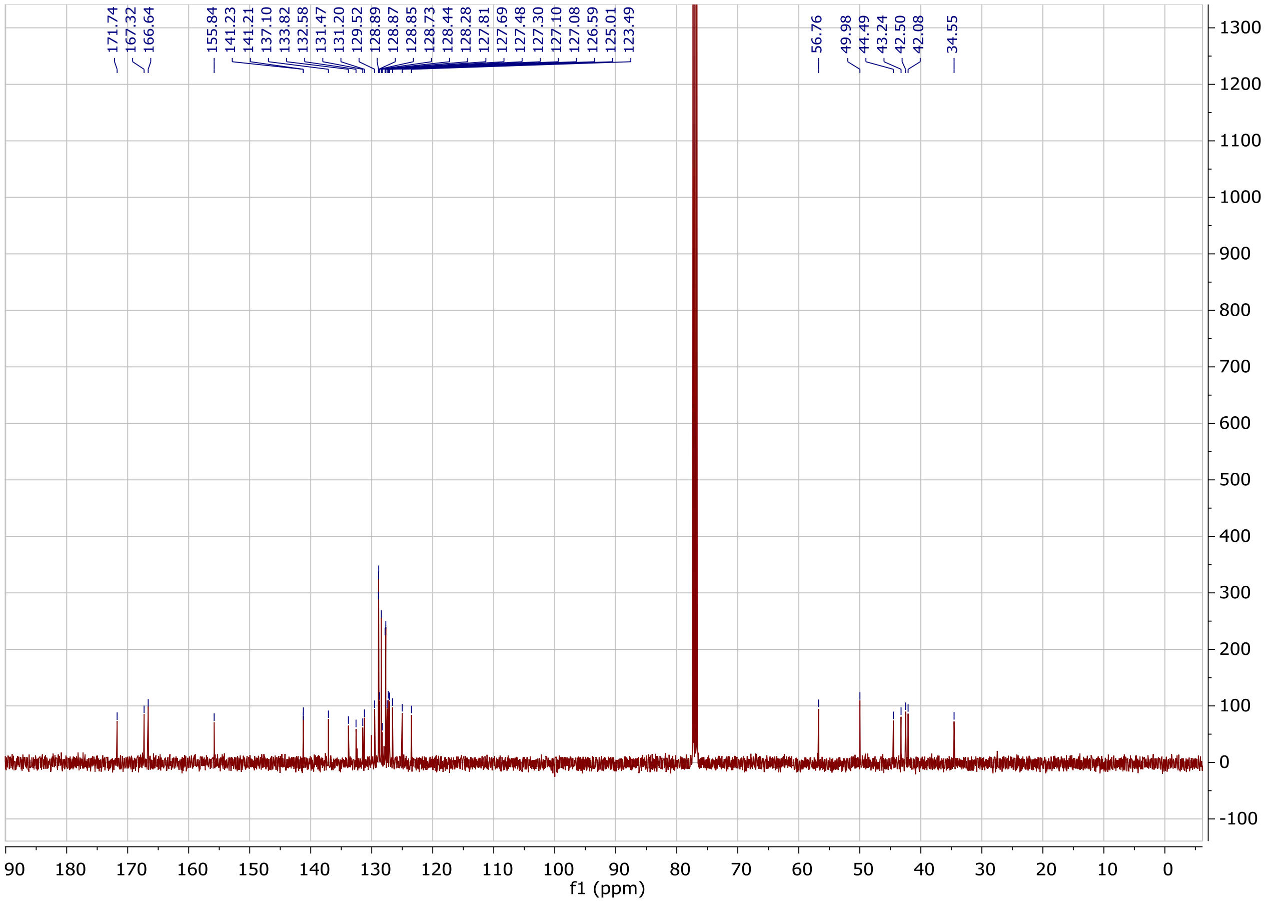

-L.G.S. - Laboratorio Grandi Strumenti - Display Report

Analysis Name	av ac192.d	Acquisition Date	03/09/22 10:19:08	Operator	Walter Panzeri
Sample Name		Method	Copy of $opt0622.MS	Instrument	esquire3000plus
Comment	1 mg/ml dil 1:100 MeOH<br>Richiedente: Caramiello				

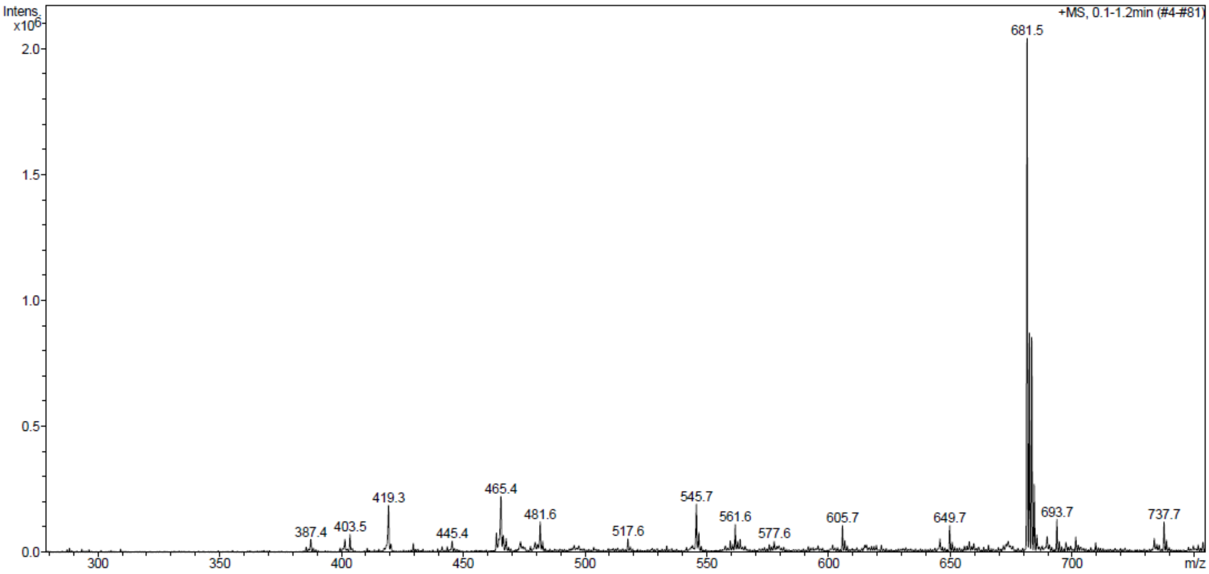

Bruker Daltonics DataAnalysis 3.1 printed: 03/09/22 10:24:41 Page 1 of 1

Figure S1. Results from Monte Carlo/MM conformational analysis. For each compounds the percentage of conformers meeting the geometrical requirements for β-turn, α-helix and 3_{10} -helix are reported.

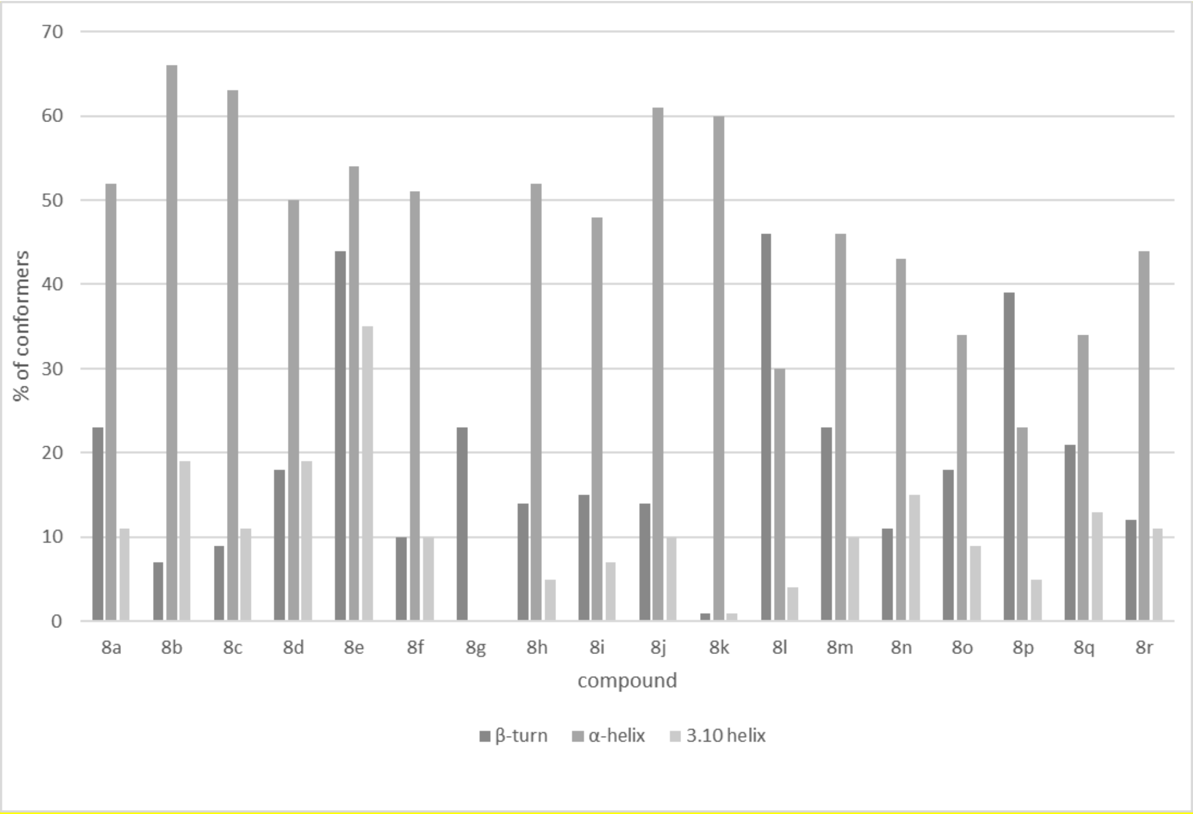

Figure S2. VT 1H NMR spectra of **8o** (400 MHz)

From the bottom to the top: a) ^{1}H NMR in $CDCl_3$ at 302 K, b) ^{1}H NMR in $CDCl_3$ at 306 K, c) ^{1}H NMR in $CDCl_3$ at 310 K, d) ^{1}H NMR in $CDCl_3$ at 314 K, e) ^{1}H NMR in $CDCl_3$ at 318 K.

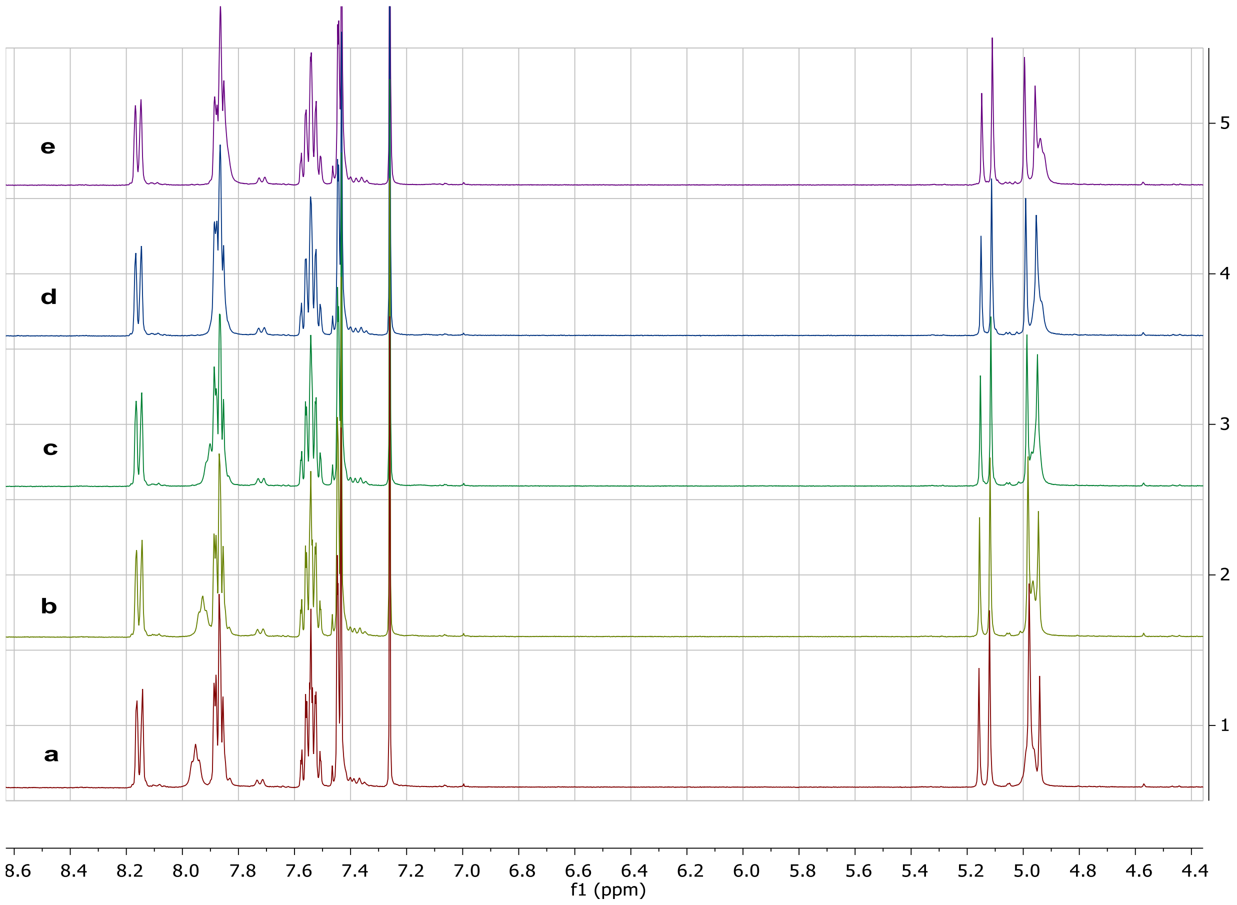

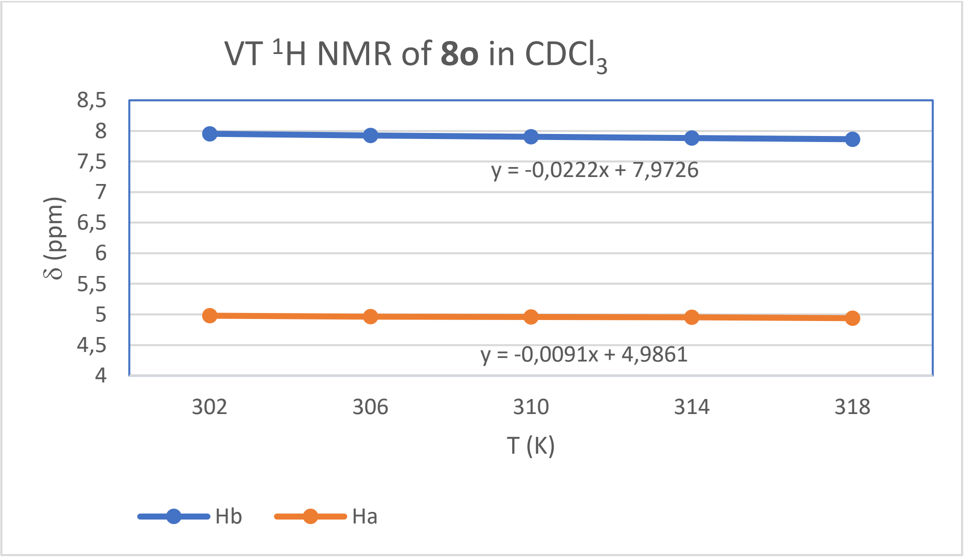

Figure S3. VT 1H NMR spectra of **8p** (400 MHz)

From the bottom to the top: a) ^{1}H NMR in $CDCl_3$ at 302 K, b) ^{1}H NMR in $CDCl_3$ at 306 K, c) ^{1}H NMR in $CDCl_3$ at 310 K, d) ^{1}H NMR in $CDCl_3$ at 314 K, e) ^{1}H NMR in $CDCl_3$ at 318 K.

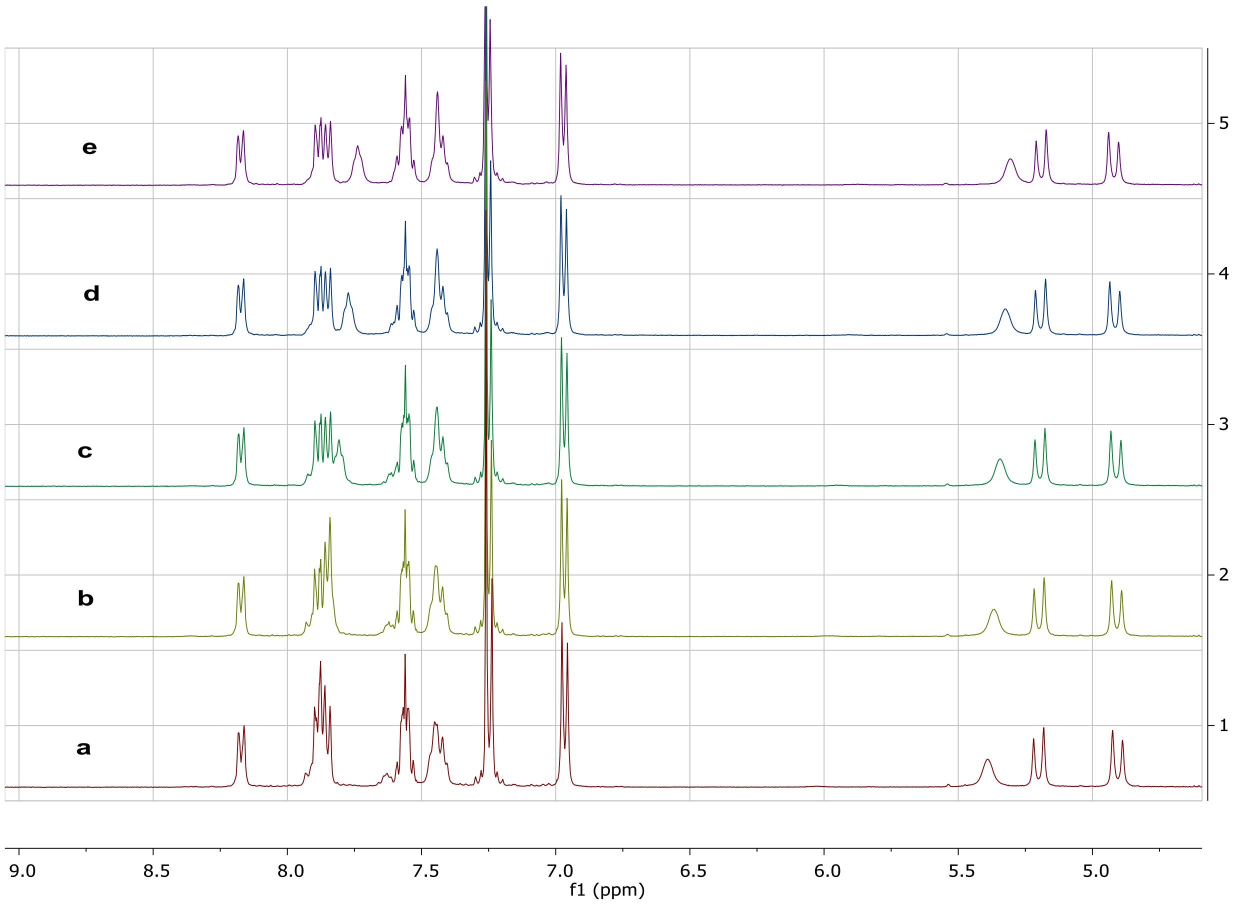

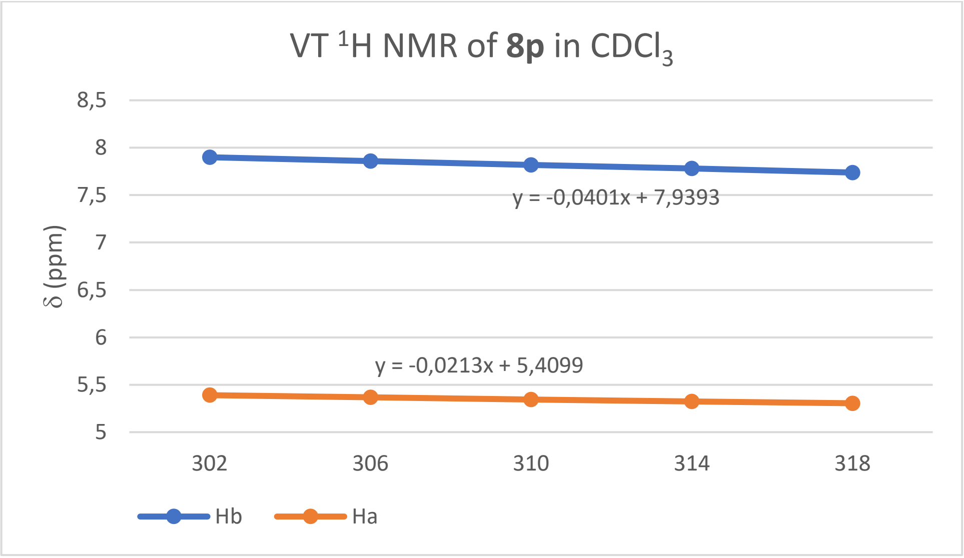

Figure S4. VT ^{1}H NMR spectra of **8r** in $CDCl_3$ (400 MHz)

From the bottom to the top: a) ^{1}H NMR in $CDCl_3$ at 302 K, b) ^{1}H NMR in $CDCl_3$ at 306 K, c) ^{1}H NMR in $CDCl_3$ at 310 K, d) ^{1}H NMR in $CDCl_3$ at 314 K, e) ^{1}H NMR in $CDCl_3$ at 318 K.

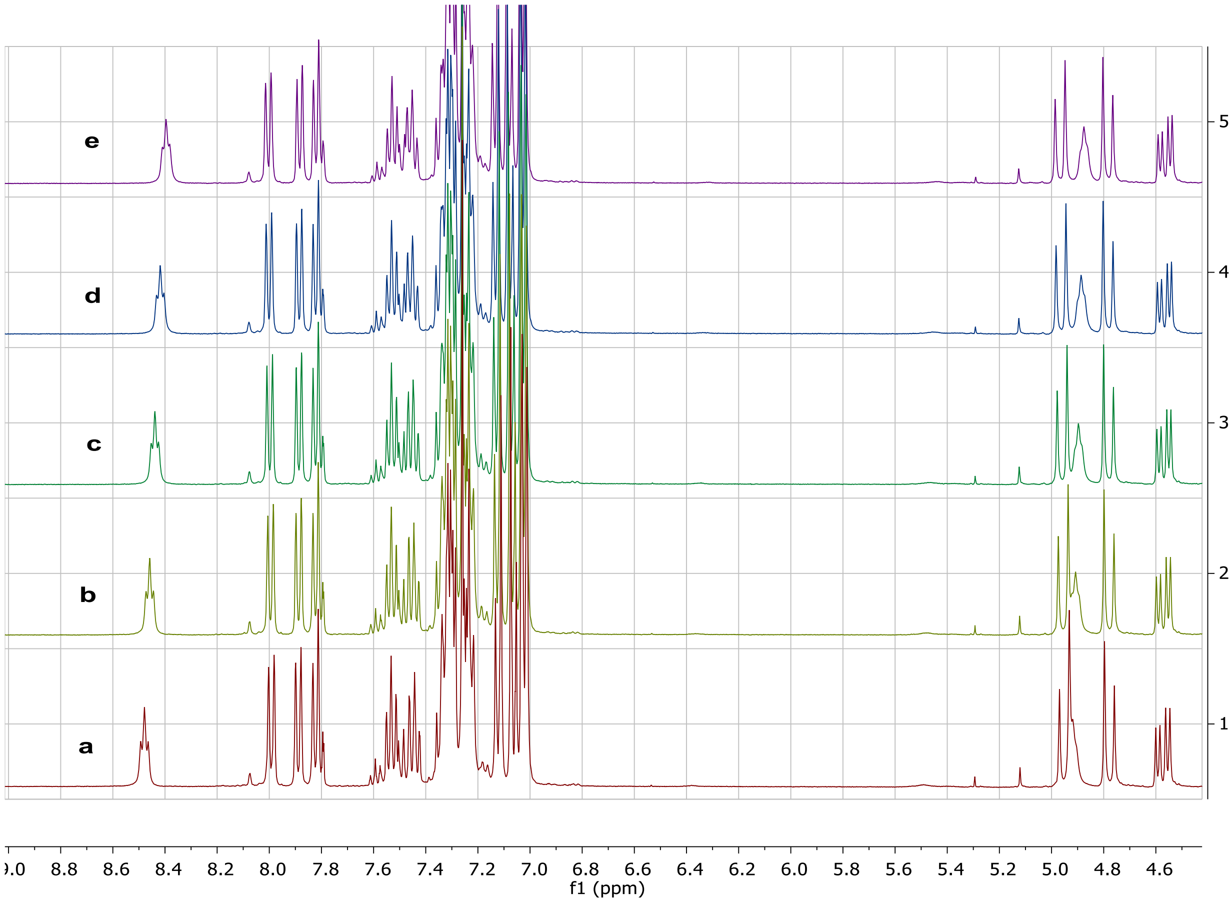

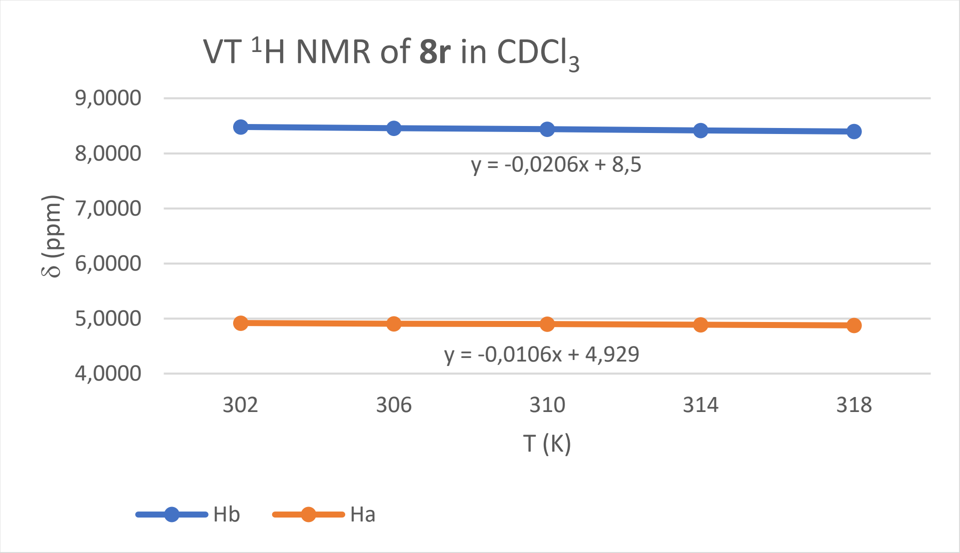

Figure S5. VT ^{1}H NMR spectra of **8p** in dmso-d_6 (400 MHz)

From the bottom to the top: a) ^{1}H NMR in dmso-d_6 at 302 K, b) ^{1}H NMR in dmso-d_6 at 306 K, c) ^{1}H NMR in dmso-d_6 at 310 K, d) ^{1}H NMR in dmso-d_6 at 314 K, e) ^{1}H NMR in dmso-d_6 at 318 K.

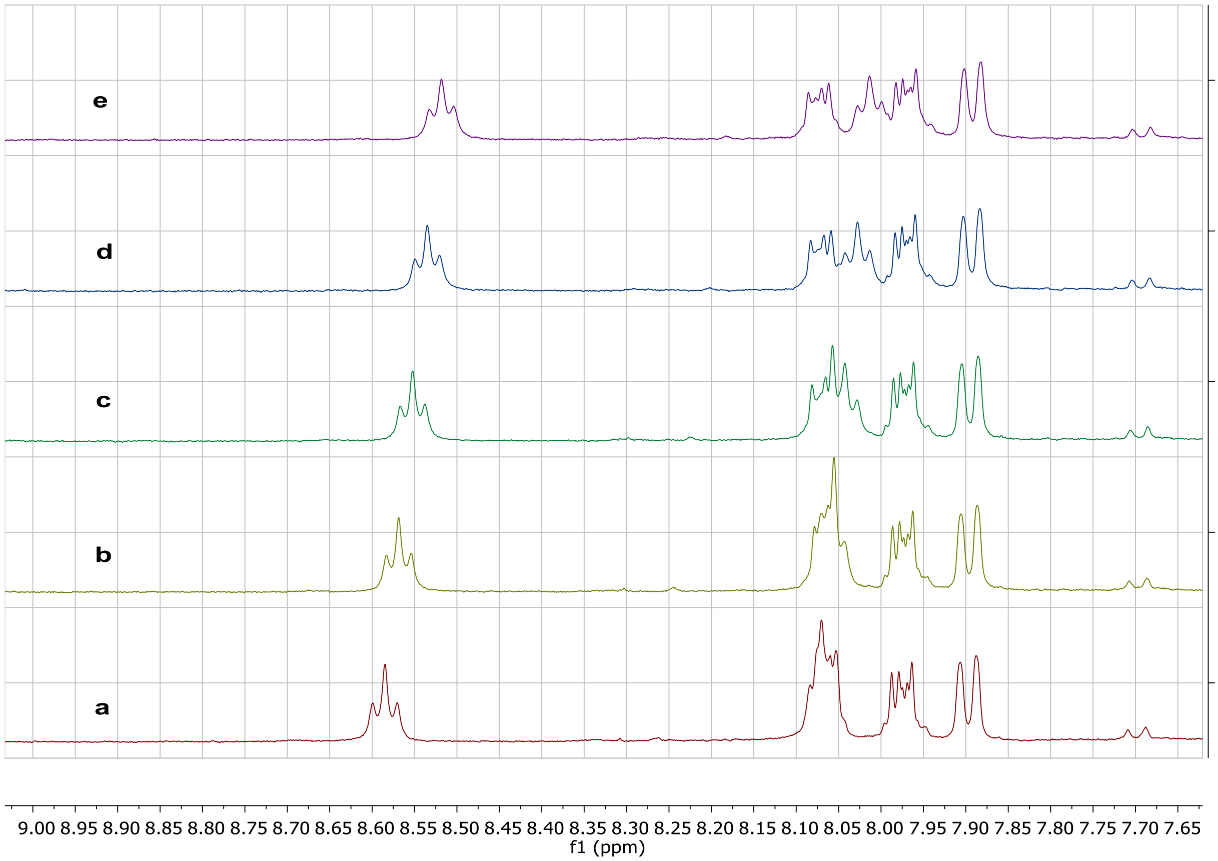

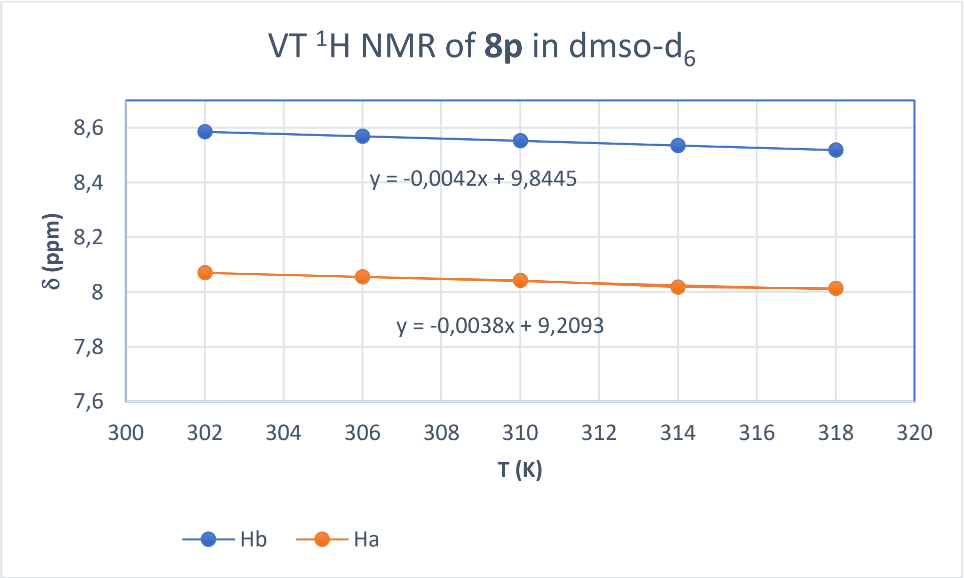

Figure S6. VT ^{1}H NMR spectra of **8r** in dmso-d_6 (400 MHz)

From the bottom to the top: a) ^{1}H NMR in dmso-d_6 at 302 K, b) ^{1}H NMR in dmso-d_6 at 304 K, c) ^{1}H NMR in dmso-d_6 at 306 K, d) ^{1}H NMR in dmso-d_6 at 310 K, e) ^{1}H NMR in dmso-d_6 at 314 K, f) ^{1}H NMR in dmso-d_6 at 318 K.

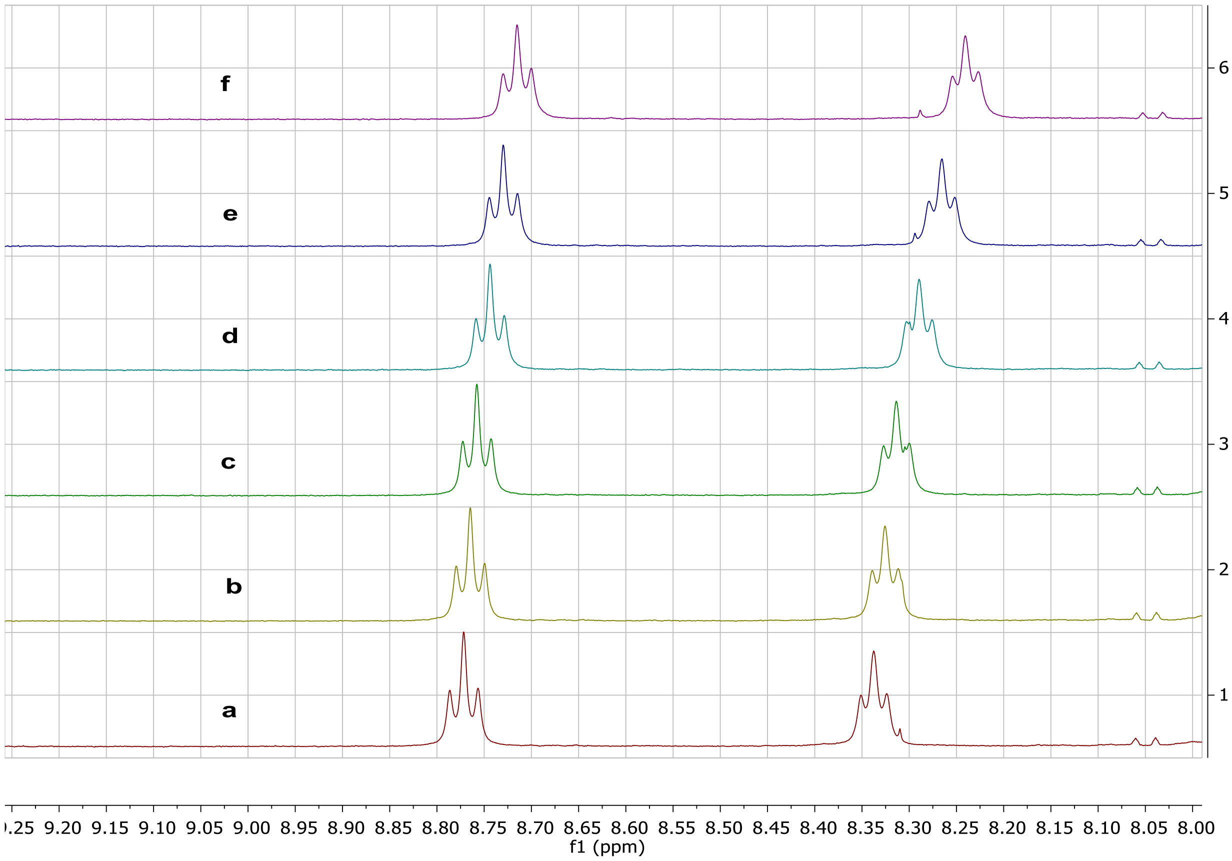

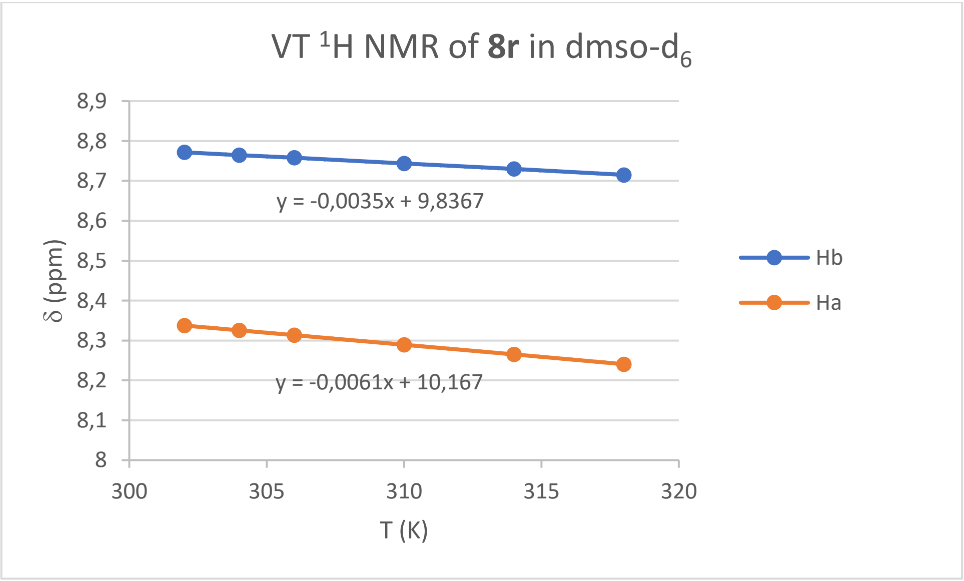

Figure S7. DMSO titration ^{1}H NMR spectra of **8p** (500 MHz)

From the bottom to the top: a) ^{1}H NMR in $CDCl_3$, b) ^{1}H NMR in $CDCl_3$ + 5 μL of DMSO, c) ^{1}H NMR in $CDCl_3$ + 10 μL of DMSO, d) ^{1}H NMR in $CDCl_3$ + 15 μL of DMSO, e) ^{1}H NMR in $CDCl_3$ + 20 μL of DMSO, f) ^{1}H NMR in $CDCl_3$ + 25 μL of DMSO; g) ^{1}H NMR in $CDCl_3$ + 30 μL of DMSO; h) ^{1}H NMR in $CDCl_3$ + 35 μL of DMSO, i) ^{1}H NMR in $CDCl_3$ + 40 μL of DMSO.

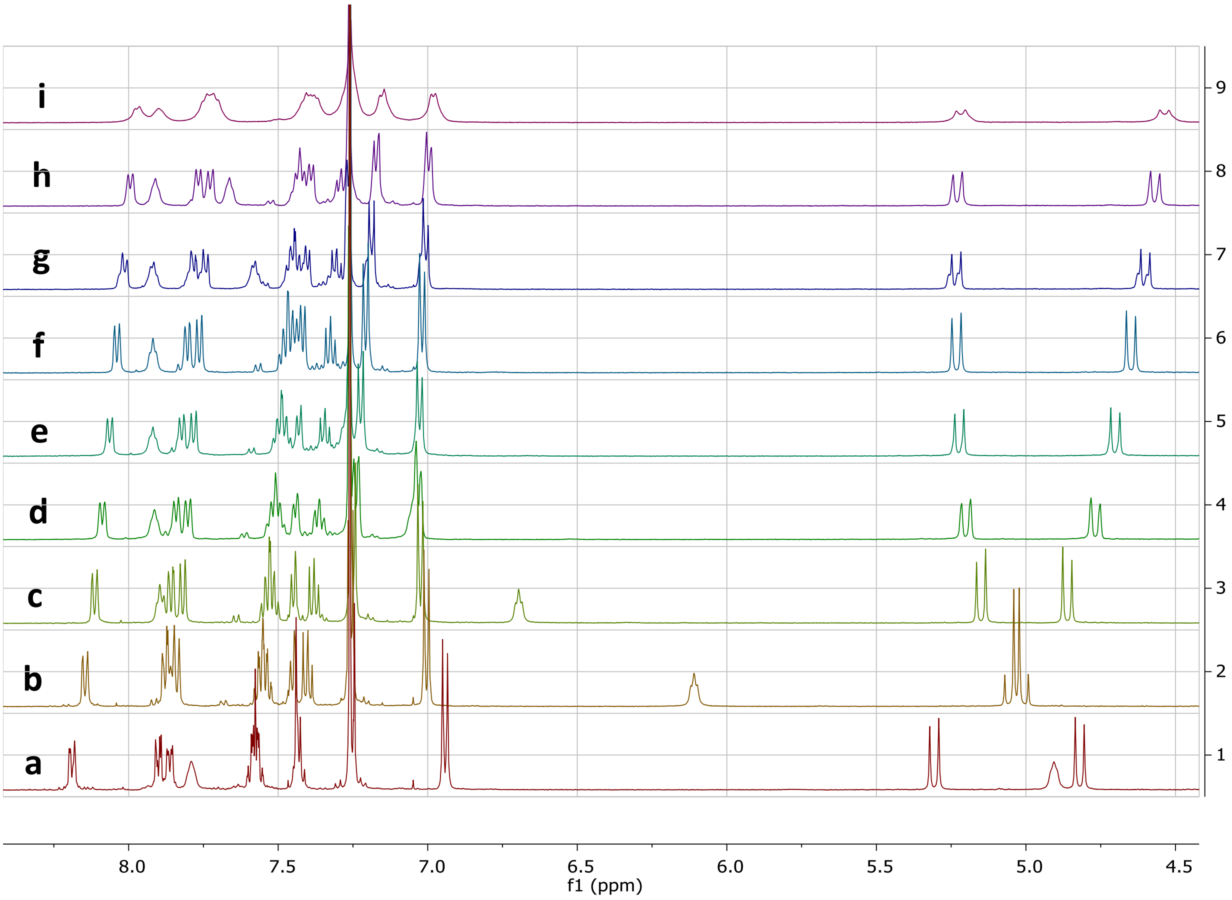

Figure S8. DMSO titration ^{1}H NMR spectra of **8r** (500 MHz)

From the bottom to the top: a) ^{1}H NMR in $CDCl_3$, b) ^{1}H NMR in $CDCl_3$ + 5 μL of DMSO, c) ^{1}H NMR in $CDCl_3$ + 10 μL of DMSO, d) ^{1}H NMR in $CDCl_3$ + 15 μL of DMSO, e) ^{1}H NMR in $CDCl_3$ + 20 μL of DMSO, f) ^{1}H NMR in $CDCl_3$ + 25 μL of DMSO; g) ^{1}H NMR in $CDCl_3$ + 30 μL of DMSO; h) ^{1}H NMR in $CDCl_3$ + 35 μL of DMSO, i) ^{1}H NMR in $CDCl_3$ + 40 μL of DMSO, j) ^{1}H NMR in $CDCl_3$ + 45 μL of DMSO, k) ^{1}H NMR in $CDCl_3$ + 50 μL of DMSO

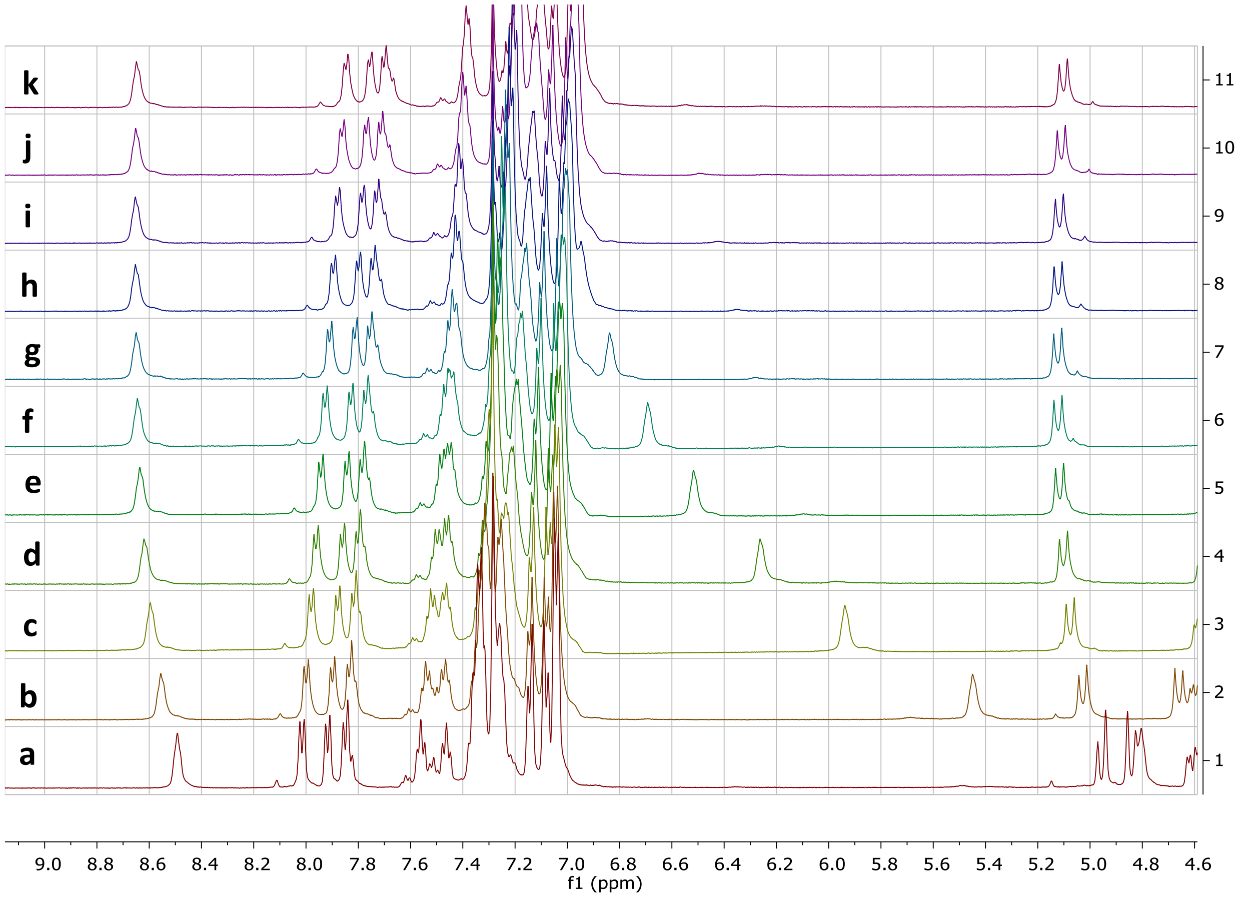

Table S1. Selected H-bond distances and angles.

Bond	D···A (Å)	D-H···A (Å)	D-H···A (°)	Equivalent positions
N3-H3N···O4	2.727(9)	1.965(6)	147.2(5)	x,y,z
N4-H4N···O3	2.843(9)	2.001(6)	166.1(5)	x,y-1,z
C38-H38···O1	3.651(12)	2.738(6)	167.5(6)	x,y,z
C31-H31···O3	3.751(11)	2.947(6)	145.5(6)	x,y-1,z
C32-H32···O1	3.784(13)	2.894(6)	160.5(7)	x,y-1,z
C25-H25···Cl1	3.775(20)	2.895(5)	158(1)	1-x,y,1-z
